# Supplementary material for: Modeling the Impact of Management Changes on the Infection Dynamics of Extended-Spectrum Beta-Lactamase-Producing Escherichia coli in the Broiler Production
Source: Microorganisms. 2022 May 7;10(5):981. doi: 10.3390/microorganisms10050981 (PMC9144090; doi:10.3390/microorganisms10050981)
Supplement: Supplementary file 1 [file microorganisms-10-00981-s001.zip › microorganisms-1705345-supplementary.pdf]

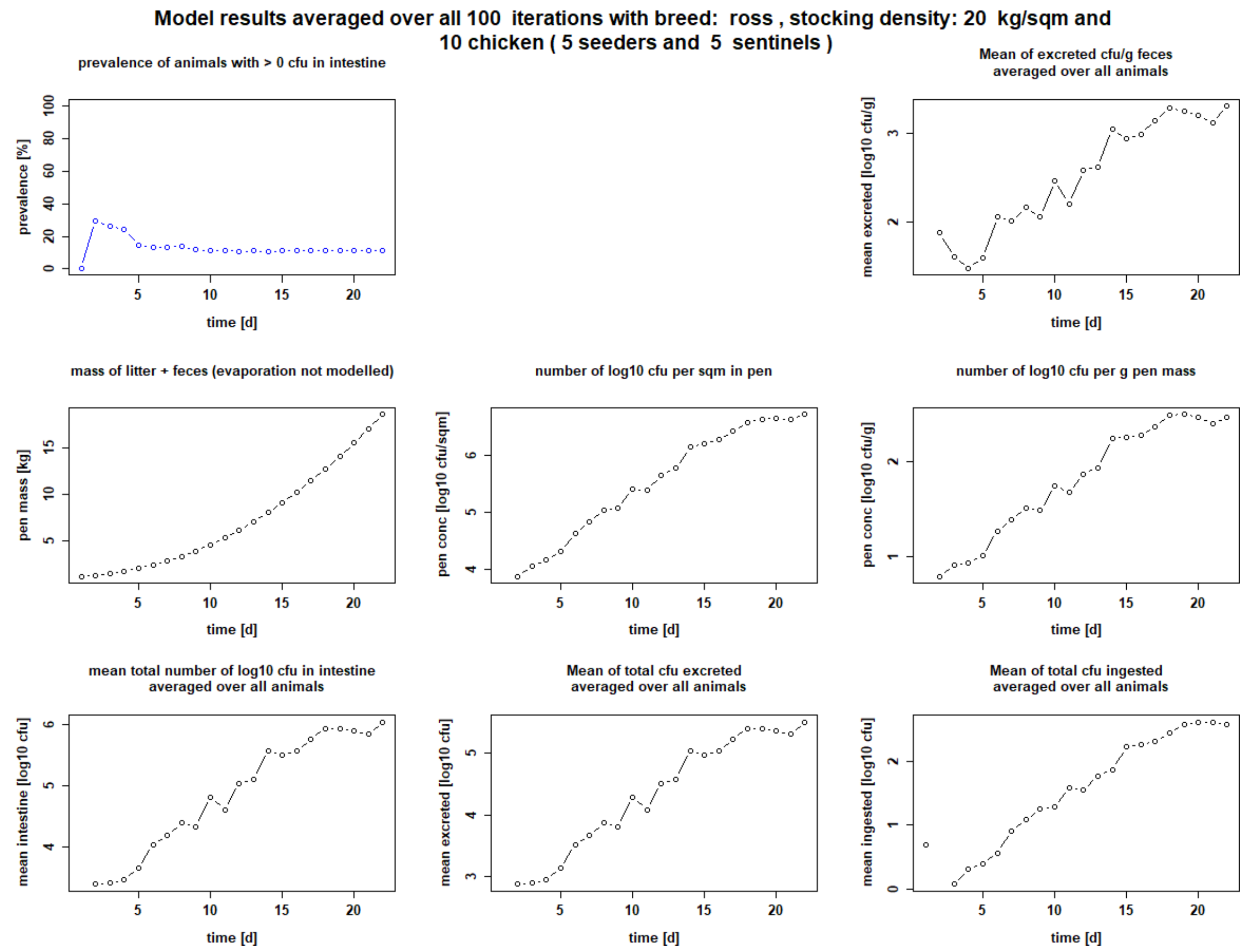

Figure S1. Calculated infection dynamic for the scenario when day-old chicks are set positive (10^1 CFU bacteria per chick on day 1). Dotplots with results for 10 chicks, 5 seeder, 5 sentinels, Ross 308, target weight 2 kg, feeding duration 21 days, stocking density 20 kg/m², 1000 g litter/m²

Table S1a (CFU). Calculated numeric values for the scenario when day-old chicks are set positive (10^1 CFU bacteria per chick on day 1). Setting: 5 seeder, 5 sentinels, Ross 308, target weight 2 kg, feeding duration 21 days, stocking density 20 kg/m^2, 1000 g litter/m^2.

day	prevalence in %	cfu/sqm	cfu/g manure	cfu in intestine	cfu/g feces	cfu ingested per animal
	(mean ± sd)	(mean ± sd)	(mean ± sd)	(mean ± sd)	(mean ± sd)	(mean ± sd)
1	0 ± 0	0e0 ± 0	0e0 ± 0	0e0 ± 0	0e0 ± 0	5 ± 0
2	29.2 ± 10.61	7.5e3 ± 10.8e3	6.2 ± 9	2.5e3 ± 3.6e3	7.5e1 ± 10.8e1	0e0 ± 0
3	26.3 ± 20.48	1.1e4 ± 1.8e4	8.2 ± 12.9	2.6e3 ± 4.7e3	4e1 ± 7.3e1	1.18 ± 2
4	24.1 ± 27.75	1.5e4 ± 3.8e4	8.6 ± 22.2	2.9e3 ± 10e3	2.9e1 ± 10e1	2.03 ± 3.8
5	14.6 ± 30.73	2.1e4 ± 8.2e4	1e1 ± 4e1	4.6e3 ± 21.2e3	3.9e1 ± 18.1e1	2.51 ± 7.4
6	13.3 ± 31.97	4.3e4 ± 27.5e4	1.9e1 ± 11.8e1	1.1e4 ± 7.9e4	1.2e2 ± 8.3e2	3.69 ± 15.6
7	13.5 ± 31.79	6.8e4 ± 44e4	2.4e1 ± 15.8e1	1.5e4 ± 10.1e4	1e2 ± 6.7e2	8.09 ± 52.8
8	13.6 ± 33.5	1.1e5 ± 6.3e5	3.3e1 ± 19.2e1	2.5e4 ± 13.8e4	1.4e2 ± 8.2e2	1.22e1 ± 8e1
9	11.7 ± 30.82	1.2e5 ± 5.9e5	3.1e1 ± 15.3e1	2.2e4 ± 10.5e4	1.1e2 ± 5.6e2	1.82e1 ± 10.7e1
10	11 ± 30.7	2.5e5 ± 16.1e5	5.6e1 ± 35.5e1	6.5e4 ± 44.2e4	2.9e2 ± 19.9e2	1.92e1 ± 9.6e1
11	11.4 ± 31.4	2.5e5 ± 12.2e5	4.7e1 ± 23.1e1	4.1e4 ± 17.2e4	1.6e2 ± 6.8e2	3.84e1 ± 24.3e1
12	10.6 ± 30.45	4.5e5 ± 21.3e5	7.3e1 ± 34.8e1	1.1e5 ± 5.5e5	3.9e2 ± 19.7e2	3.56e1 ± 17.4e1
13	11 ± 31.45	6e5 ± 31e5	8.6e1 ± 44.1e1	1.3e5 ± 7e5	4.2e2 ± 23.4e2	5.95e1 ± 28.3e1
14	10.8 ± 30.93	1.4e6 ± 7.4e6	1.8e2 ± 9.2e2	3.7e5 ± 19.5e5	1.1e3 ± 5.9e3	7.58e1 ± 38.9e1
15	11 ± 31.45	1.6e6 ± 8.7e6	1.8e2 ± 9.6e2	3.1e5 ± 17e5	8.7e2 ± 47.6e2	1.7e2 ± 8.9e2
16	11 ± 31.45	1.9e6 ± 9.3e6	1.9e2 ± 9.1e2	3.6e5 ± 19.1e5	9.9e2 ± 51.8e2	1.87e2 ± 9.9e2
17	11 ± 31.45	2.7e6 ± 12.9e6	2.3e2 ± 11.3e2	5.7e5 ± 28.7e5	1.4e3 ± 7.1e3	2.08e2 ± 10.1e2
18	11 ± 31.45	3.9e6 ± 20.7e6	3.1e2 ± 16.3e2	8.5e5 ± 49.7e5	2e3 ± 11.4e3	2.79e2 ± 13.4e2
19	11 ± 31.45	4.4e6 ± 23e6	3.1e2 ± 16.3e2	8.2e5 ± 42.7e5	1.8e3 ± 9.3e3	3.86e2 ± 20.5e2
20	11 ± 31.45	4.5e6 ± 20.9e6	2.9e2 ± 13.5e2	7.6e5 ± 33.1e5	1.6e3 ± 7e3	4.22e2 ± 21.9e2
21	11 ± 31.45	4.3e6 ± 19e6	2.5e2 ± 11.2e2	6.8e5 ± 29.3e5	1.3e3 ± 5.8e3	4.15e2 ± 19.3e2
22	11 ± 31.45	5.4e6 ± 23.1e6	2.9e2 ± 12.4e2	1.1e6 ± 4.7e6	2e3 ± 9e3	3.81e2 ± 16.9e2

The average of the prevalence, the CFU-values per square meter (cfu/sqm) and per gram manure are calculated over all iterations. The CFU values in the intestine as well as the excreted and ingested quantities are obtained by calculating the average over all animals in one iteration at first and then over all iterations.

Table S1b (log_{10} CFU). Calculated numeric values for the scenario when day-old chicks are set positive (10^1 CFU bacteria per chick on day 1). Setting: 5 seeder, 5 sentinels, Ross 308, target weight 2 kg, feeding duration 21 days, stocking density 20 kg/m^2, 1000 g litter/m^2

day	prevalence in %	cfu/sqm	cfu/g manure	cfu in intestine	cfu/g feces	cfu ingested per animal
	(% mean ± sd)	(log10 mean ± sd)	(log10 mean ± sd)	(log10 mean ± sd)	(log10 mean ± sd)	(log10 mean ± sd)
1	0 ± 0	-Inf ± -Inf	-Inf ± -Inf	-Inf ± -Inf	-Inf ± -Inf	0.7 ± 0
2	29.2 ± 10.61	3.87 ± 4.03	0.79 ± 0.95	3.4 ± 3.56	1.87 ± 2.03	-Inf ± 0
3	26.3 ± 20.48	4.06 ± 4.25	0.92 ± 1.11	3.41 ± 3.67	1.6 ± 1.86	0.07 ± 0.31
4	24.1 ± 27.75	4.16 ± 4.58	0.93 ± 1.35	3.47 ± 4	1.47 ± 2	0.31 ± 0.58
5	14.6 ± 30.73	4.32 ± 4.91	1.01 ± 1.6	3.66 ± 4.33	1.59 ± 2.26	0.4 ± 0.87
6	13.3 ± 31.97	4.64 ± 5.44	1.27 ± 2.07	4.04 ± 4.9	2.06 ± 2.92	0.57 ± 1.19
7	13.5 ± 31.79	4.83 ± 5.64	1.39 ± 2.2	4.19 ± 5	2.01 ± 2.83	0.91 ± 1.72
8	13.6 ± 33.5	5.03 ± 5.8	1.51 ± 2.28	4.39 ± 5.14	2.16 ± 2.91	1.08 ± 1.9
9	11.7 ± 30.82	5.07 ± 5.77	1.49 ± 2.18	4.33 ± 5.02	2.06 ± 2.74	1.26 ± 2.03
10	11 ± 30.7	5.4 ± 6.21	1.75 ± 2.55	4.81 ± 5.65	2.46 ± 3.3	1.28 ± 1.98
11	11.4 ± 31.4	5.4 ± 6.09	1.67 ± 2.36	4.61 ± 5.24	2.21 ± 2.83	1.58 ± 2.39
12	10.6 ± 30.45	5.65 ± 6.33	1.87 ± 2.54	5.03 ± 5.74	2.59 ± 3.29	1.55 ± 2.24
13	11 ± 31.45	5.78 ± 6.49	1.93 ± 2.64	5.1 ± 5.85	2.63 ± 3.37	1.77 ± 2.45
14	10.8 ± 30.93	6.15 ± 6.87	2.24 ± 2.96	5.57 ± 6.29	3.05 ± 3.77	1.88 ± 2.59
15	11 ± 31.45	6.21 ± 6.94	2.26 ± 2.98	5.49 ± 6.23	2.94 ± 3.68	2.23 ± 2.95
16	11 ± 31.45	6.28 ± 6.97	2.27 ± 2.96	5.56 ± 6.28	3 ± 3.71	2.27 ± 3
17	11 ± 31.45	6.43 ± 7.11	2.37 ± 3.05	5.76 ± 6.46	3.15 ± 3.85	2.32 ± 3.01
18	11 ± 31.45	6.59 ± 7.32	2.49 ± 3.21	5.93 ± 6.7	3.29 ± 4.06	2.45 ± 3.13
19	11 ± 31.45	6.65 ± 7.36	2.5 ± 3.21	5.92 ± 6.63	3.26 ± 3.97	2.59 ± 3.31
20	11 ± 31.45	6.65 ± 7.32	2.46 ± 3.13	5.88 ± 6.52	3.21 ± 3.84	2.63 ± 3.34
21	11 ± 31.45	6.63 ± 7.28	2.4 ± 3.05	5.83 ± 6.47	3.13 ± 3.76	2.62 ± 3.28
22	11 ± 31.45	6.73 ± 7.36	2.46 ± 3.09	6.03 ± 6.67	3.31 ± 3.95	2.58 ± 3.23

The average of the prevalence, the CFU-values per square meter (cfu/sqm) and per gram manure are calculated over all iterations. The CFU values in the intestine as well as the excreted and ingested quantities are obtained by calculating the average over all animals in one iteration at first and then over all iterations. The decadic logarithm of zero or negative values is negative infinite (-Inf).

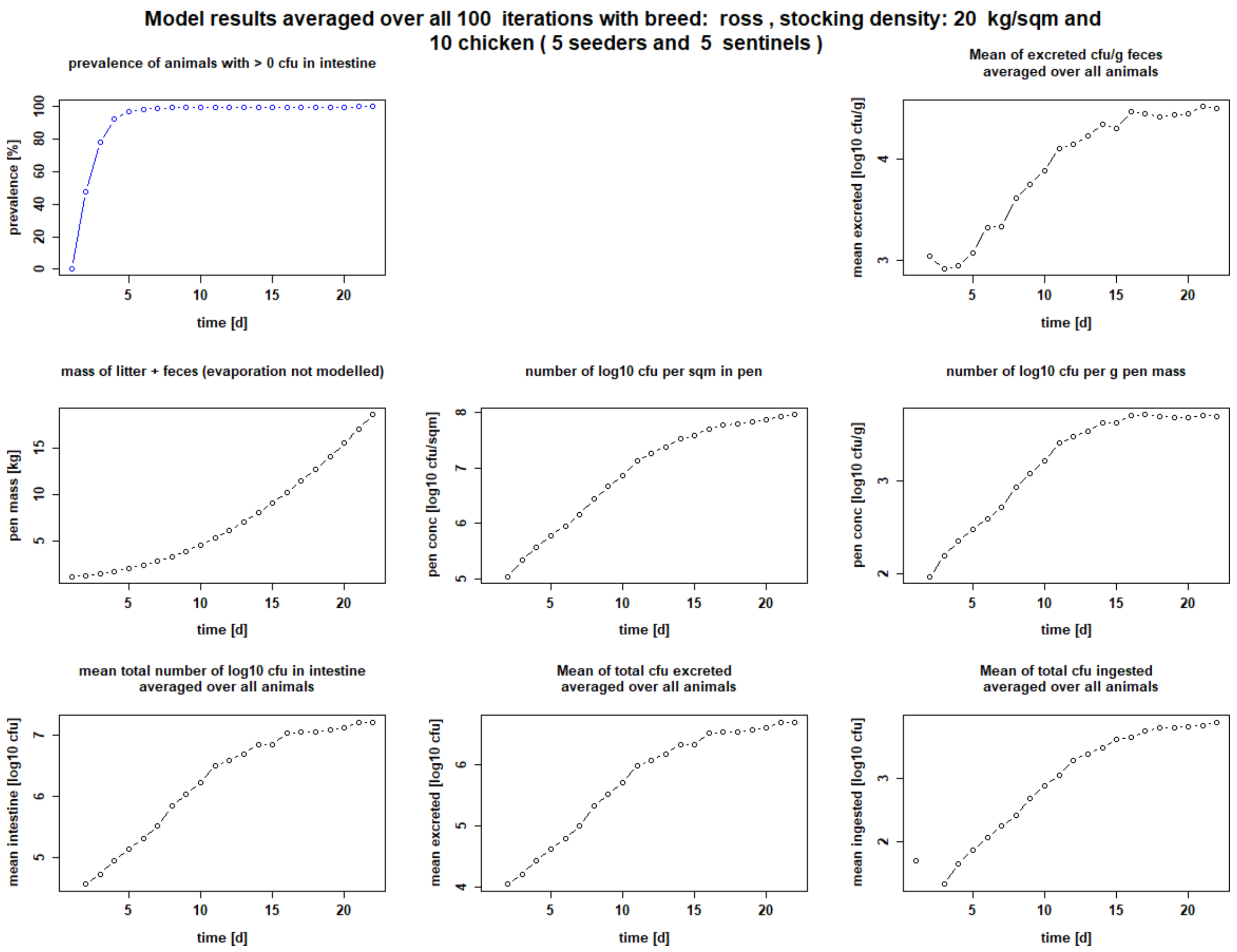

Figure S2. Calculated infection dynamic for the scenario when day-old chicks are set positive (10^2 CFU bacteria per chick on day 1). Dotplots with results for 10 chicks, 5 seeder, 5 sentinels, Ross 308, target weight 2 kg, feeding duration 21 days, stocking density 20 kg/m^2, 1000 g litter/m^2

Table S2a (CFU). Calculated numeric values for the scenario when day-old chicks are set positive (10^2 CFU bacteria per chick on day 1). Setting: 5 seeder, 5 sentinels, Ross 308, target weight 2 kg, feeding duration 21 days, stocking density 20 kg/m^2, 1000 g litter/m^2

day	prevalence in %	cfu/sqm	cfu/g manure	cfu in intestine	cfu/g feces	cfu ingested per animal
	(mean ± sd)	(mean ± sd)	(mean ± sd)	(mean ± sd)	(mean ± sd)	(mean ± sd)
1	0 ± 0	0e0 ± 0	0e0 ± 0	0e0 ± 0	0e0 ± 0	5e1 ± 0e1
2	47.6 ± 4.74	1.1e5 ± 1.3e5	9.2e1 ± 10.8e1	3.7e4 ± 4.3e4	1.1e3 ± 1.3e3	0e0 ± 0
3	78 ± 21.79	2.2e5 ± 2.7e5	1.5e2 ± 2e2	5.4e4 ± 7.5e4	8.3e2 ± 11.7e2	2.13e1 ± 2.6e1
4	92.4 ± 13.86	3.8e5 ± 4.5e5	2.2e2 ± 2.7e2	9e4 ± 11.3e4	8.9e2 ± 11.3e2	4.5e1 ± 5.8e1
5	97.1 ± 9.13	6.1e5 ± 10.4e5	3e2 ± 5.1e2	1.4e5 ± 2.9e5	1.2e3 ± 2.5e3	7.42e1 ± 9e1
6	98.5 ± 5.92	9.1e5 ± 11.2e5	3.9e2 ± 4.8e2	2e5 ± 2.6e5	2.1e3 ± 2.8e3	1.16e2 ± 2e2
7	99.2 ± 4.42	1.4e6 ± 1.9e6	5.2e2 ± 6.7e2	3.3e5 ± 4.8e5	2.2e3 ± 3.2e3	1.74e2 ± 2.1e2
8	99.4 ± 5.09	2.8e6 ± 4.1e6	8.5e2 ± 12.4e2	6.9e5 ± 11.5e5	4.1e3 ± 6.8e3	2.6e2 ± 3.4e2
9	99.4 ± 4.45	4.6e6 ± 7.1e6	1.2e3 ± 1.8e3	1.1e6 ± 1.8e6	5.6e3 ± 9.3e3	4.73e2 ± 6.9e2
10	99.6 ± 3.15	7.4e6 ± 11.9e6	1.6e3 ± 2.6e3	1.7e6 ± 3.1e6	7.6e3 ± 13.9e3	7.49e2 ± 11.5e2
11	99.5 ± 5	1.3e7 ± 2.6e7	2.5e3 ± 4.9e3	3.2e6 ± 6.9e6	1.3e4 ± 2.7e4	1.12e3 ± 1.8e3
12	99.7 ± 2.23	1.8e7 ± 3e7	3e3 ± 4.9e3	3.8e6 ± 7.1e6	1.4e4 ± 2.5e4	1.91e3 ± 3.7e3
13	99.7 ± 3	2.4e7 ± 3.3e7	3.4e3 ± 4.7e3	5e6 ± 7e6	1.7e4 ± 2.3e4	2.41e3 ± 4e3
14	99.6 ± 4	3.3e7 ± 4.1e7	4.1e3 ± 5.1e3	7.1e6 ± 8.9e6	2.1e4 ± 2.7e4	3.01e3 ± 4.2e3
15	99.8 ± 2	3.8e7 ± 4.1e7	4.2e3 ± 4.5e3	7.1e6 ± 8.4e6	2e4 ± 2.3e4	4e3 ± 4.9e3
16	99.7 ± 3	5.1e7 ± 4.8e7	5e3 ± 4.8e3	1.1e7 ± 1.1e7	2.9e4 ± 2.9e4	4.32e3 ± 4.7e3
17	99.9 ± 1	5.9e7 ± 5.2e7	5.2e3 ± 4.5e3	1.1e7 ± 1.1e7	2.8e4 ± 2.6e4	5.51e3 ± 5.3e3
18	99.8 ± 2	6.3e7 ± 5.5e7	4.9e3 ± 4.3e3	1.1e7 ± 1.2e7	2.5e4 ± 2.7e4	6.13e3 ± 5.4e3
19	99.9 ± 1	6.8e7 ± 5.3e7	4.8e3 ± 3.8e3	1.2e7 ± 1.1e7	2.7e4 ± 2.4e4	6.2e3 ± 5.5e3
20	99.9 ± 1	7.3e7 ± 5.4e7	4.7e3 ± 3.5e3	1.3e7 ± 1.1e7	2.7e4 ± 2.3e4	6.48e3 ± 5.1e3
21	100 ± 0	8.5e7 ± 6.1e7	5e3 ± 3.6e3	1.6e7 ± 1.3e7	3.2e4 ± 2.6e4	6.73e3 ± 5e3
22	100 ± 0	9.2e7 ± 6e7	4.9e3 ± 3.2e3	1.6e7 ± 1.2e7	3.1e4 ± 2.3e4	7.59e3 ± 5.4e3

The average of the prevalence, the CFU-values per square meter (cfu/sqm) and per gram manure are calculated over all iterations. The CFU values in the intestine as well as the excreted and ingested quantities are obtained by calculating the average over all animals in one iteration at first and then over all iterations.

Table S2b (log_{10} CFU). Calculated numeric values for the scenario when day-old chicks are set positive (10^2 CFU bacteria per chick on day 1). Setting: 5 seeder, 5 sentinels, Ross 308, target weight 2 kg, feeding duration 21 days, stocking density 20 kg/m^2, 1000 g litter/m^2

day	prevalence in %	cfu/sqm	cfu/g manure	cfu in intestine	cfu/g feces	cfu ingested per animal
	(% mean ± sd)	(log10 mean ± sd)	(log10 mean ± sd)	(log10 mean ± sd)	(log10 mean ± sd)	(log10 mean ± sd)
1	0 ± 0	-Inf ± -Inf	-Inf ± -Inf	-Inf ± -Inf	-Inf ± -Inf	1.7 ± 0
2	47.6 ± 4.74	5.04 ± 5.11	1.96 ± 2.03	4.56 ± 4.64	3.04 ± 3.11	-Inf ± 0
3	78 ± 21.79	5.33 ± 5.44	2.19 ± 2.29	4.73 ± 4.88	2.92 ± 3.07	1.33 ± 1.41
4	92.4 ± 13.86	5.58 ± 5.66	2.35 ± 2.43	4.95 ± 5.05	2.95 ± 3.05	1.65 ± 1.76
5	97.1 ± 9.13	5.78 ± 6.02	2.47 ± 2.71	5.15 ± 5.46	3.08 ± 3.39	1.87 ± 1.95
6	98.5 ± 5.92	5.96 ± 6.05	2.59 ± 2.68	5.3 ± 5.42	3.33 ± 3.44	2.06 ± 2.3
7	99.2 ± 4.42	6.16 ± 6.27	2.71 ± 2.82	5.52 ± 5.68	3.34 ± 3.5	2.24 ± 2.33
8	99.4 ± 5.09	6.44 ± 6.61	2.93 ± 3.09	5.84 ± 6.06	3.61 ± 3.83	2.41 ± 2.53
9	99.4 ± 4.45	6.66 ± 6.85	3.08 ± 3.26	6.03 ± 6.25	3.75 ± 3.97	2.67 ± 2.84
10	99.6 ± 3.15	6.87 ± 7.08	3.21 ± 3.42	6.23 ± 6.49	3.88 ± 4.14	2.87 ± 3.06
11	99.5 ± 5	7.13 ± 7.41	3.4 ± 3.69	6.51 ± 6.84	4.1 ± 4.43	3.05 ± 3.26
12	99.7 ± 2.23	7.26 ± 7.48	3.47 ± 3.69	6.58 ± 6.85	4.14 ± 4.4	3.28 ± 3.57
13	99.7 ± 3	7.38 ± 7.52	3.53 ± 3.68	6.7 ± 6.85	4.22 ± 4.37	3.38 ± 3.6
14	99.6 ± 4	7.52 ± 7.61	3.62 ± 3.7	6.85 ± 6.95	4.33 ± 4.43	3.48 ± 3.62
15	99.8 ± 2	7.58 ± 7.61	3.62 ± 3.65	6.85 ± 6.92	4.3 ± 4.37	3.6 ± 3.69
16	99.7 ± 3	7.71 ± 7.69	3.7 ± 3.68	7.03 ± 7.03	4.46 ± 4.46	3.64 ± 3.67
17	99.9 ± 1	7.77 ± 7.71	3.71 ± 3.66	7.05 ± 7.02	4.44 ± 4.42	3.74 ± 3.72
18	99.8 ± 2	7.8 ± 7.74	3.69 ± 3.64	7.04 ± 7.07	4.41 ± 4.43	3.79 ± 3.73
19	99.9 ± 1	7.83 ± 7.73	3.68 ± 3.58	7.09 ± 7.03	4.43 ± 4.37	3.79 ± 3.74
20	99.9 ± 1	7.86 ± 7.74	3.67 ± 3.54	7.12 ± 7.05	4.44 ± 4.37	3.81 ± 3.71
21	100 ± 0	7.93 ± 7.79	3.7 ± 3.56	7.21 ± 7.13	4.51 ± 4.42	3.83 ± 3.7
22	100 ± 0	7.96 ± 7.78	3.69 ± 3.51	7.21 ± 7.09	4.49 ± 4.37	3.88 ± 3.74

The average of the prevalence, the CFU-values per square meter (cfu/sqm) and per gram manure are calculated over all iterations. The CFU values in the intestine as well as the excreted and ingested quantities are obtained by calculating the average over all animals in one iteration at first and then over all iterations. The decadic logarithm of zero or negative values is negative infinite (-Inf).

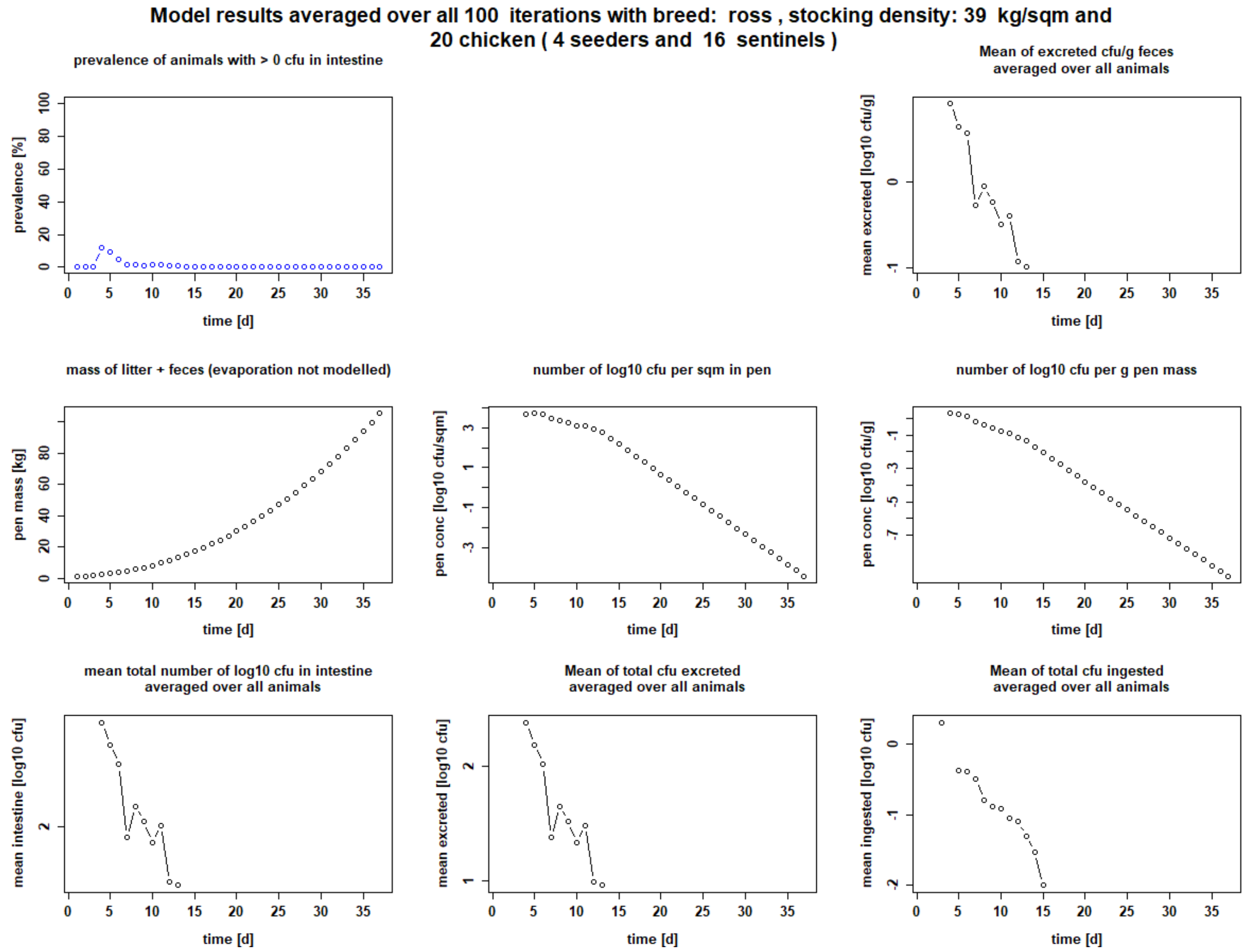

Figure S3. Calculated infection dynamic for the scenario when chicks are set positive with 10^1 CFU bacteria per chick on day 3. Dotplots with results for 20 chicks, 4 seeder, 16 sentinels, Ross 308, target weight 2 kg, feeding duration 36 days, stocking density 39 kg/m^2, 1000 g litter/m^2

Table S3a (CFU). Calculated numeric values for the scenario when chicks are set positive with 10^1 CFU bacteria per chick on day 3. Setting: 20 chicks, 4 seeder, 16 sentinels, Ross 308, target weight 2 kg, feeding duration 36 days, stocking density 39 kg/m2, 1000 g litter/m2

day	prevalence in %	cfu/sqm	cfu/g manure	cfu in intestine	cfu/g feces	cfu ingested per animal
	(mean ± sd)	(mean ± sd)	(mean ± sd)	(mean ± sd)	(mean ± sd)	(mean ± sd)
1	0 ± 0	0e0 ± 0	0e0 ± 0	0e0 ± 0	0e0 ± 0	0e0 ± 0
2	0 ± 0	0e0 ± 0	0e0 ± 0	0e0 ± 0	0e0 ± 0	0e0 ± 0
3	0 ± 0	0e0 ± 0	0e0 ± 0	0e0 ± 0	0e0 ± 0	2 ± 0
4	11.65 ± 4.66	5.4e3 ± 8.8e3	2.3 ± 3.7	9.3e2 ± 15.1e2	9.2 ± 15	0e0 ± 0
5	11.15 ± 13.16	6.3e3 ± 9.5e3	2.1 ± 3.1	6.1e2 ± 11.1e2	5.3 ± 9.5	5.3e-1 ± 11.1e-1
6	5.85 ± 11.24	5.1e3 ± 9.5e3	1.4 ± 2.7	3.3e2 ± 10.2e2	3.5 ± 10.7	5.2e-1 ± 11.4e-1
7	2.15 ± 12.62	3.8e3 ± 13.6e3	8.6e-1 ± 30.4e-1	2.2e2 ± 16.2e2	1.5 ± 10.7	3.7e-1 ± 11.3e-1
8	2.1 ± 12.8	3.9e3 ± 20.4e3	7.2e-1 ± 37.3e-1	3.5e2 ± 23.4e2	2 ± 13.8	2.3e-1 ± 14.8e-1
9	2 ± 13.08	5.4e3 ± 36.7e3	8.1e-1 ± 55.9e-1	5.8e2 ± 45.9e2	3.1 ± 24.3	3e-1 ± 20.2e-1
10	2.35 ± 13.92	6.1e3 ± 43.2e3	7.8e-1 ± 54.9e-1	5.9e2 ± 42.8e2	2.6 ± 19.2	4.5e-1 ± 34.1e-1
11	1.9 ± 13.39	6.9e3 ± 48.6e3	7.3e-1 ± 51.9e-1	6.5e2 ± 46.7e2	2.6 ± 18.4	4.9e-1 ± 36.9e-1
12	1.95 ± 13.72	5e3 ± 36.2e3	4.5e-1 ± 32.9e-1	2.7e2 ± 20.4e2	9.6e-1 ± 72.9e-1	5.4e-1 ± 39.2e-1
13	1.9 ± 13.39	5.7e3 ± 42.1e3	4.4e-1 ± 33.1e-1	5.4e2 ± 41.1e2	1.8 ± 13.7	3.5e-1 ± 26.3e-1
14	2 ± 14.07	1e4 ± 7.2e4	7e-1 ± 48.9e-1	1.3e3 ± 8.9e3	3.8 ± 27	3.8e-1 ± 28.5e-1
15	2 ± 14.07	1.1e4 ± 7.8e4	6.6e-1 ± 46.4e-1	1e3 ± 7.2e3	2.8 ± 20.1	6.6e-1 ± 46.5e-1
16	2 ± 14.07	9.9e3 ± 70.7e3	5.2e-1 ± 37.4e-1	7.5e2 ± 54.9e2	2 ± 14.9	6.7e-1 ± 47.3e-1
17	2 ± 14.07	2.1e4 ± 15.2e4	1 ± 7.1	2.8e3 ± 20.3e3	7 ± 50.2	5.7e-1 ± 40.6e-1
18	2 ± 14.07	2.7e4 ± 19.5e4	1.1 ± 8.2	2.8e3 ± 20.5e3	6.5 ± 47	1.18 ± 8.4
19	2 ± 14.07	4.8e4 ± 33.9e4	1.8 ± 12.8	5.9e3 ± 41.6e3	1.3e1 ± 9.1e1	1.43 ± 10.2
20	2 ± 14.07	6.9e4 ± 48.7e4	2.4 ± 16.6	7.7e3 ± 54.4e3	1.6e1 ± 11.4e1	2.44 ± 17.2
21	2 ± 14.07	7.4e4 ± 52.4e4	2.3 ± 16.2	6.8e3 ± 48.3e3	1.3e1 ± 9.5e1	3.36 ± 23.7
22	2 ± 14.07	7.9e4 ± 55.7e4	2.2 ± 15.8	7.1e3 ± 50.9e3	1.4e1 ± 9.7e1	3.48 ± 24.5
23	2 ± 14.07	1e5 ± 7.1e5	2.6 ± 18.4	1e4 ± 7.5e4	1.9e1 ± 13.5e1	3.56 ± 25.1
24	2 ± 14.07	1.4e5 ± 9.8e5	3.3 ± 23.2	1.5e4 ± 10.7e4	2.5e1 ± 17.7e1	4.35 ± 30.8
25	2 ± 14.07	1.9e5 ± 13.2e5	4.1 ± 28.8	2e4 ± 14.4e4	3.2e1 ± 23e1	5.75 ± 40.5
26	2 ± 14.07	1.6e5 ± 11.6e5	3.2 ± 23.4	1.1e4 ± 10.6e4	1.8e1 ± 16.7e1	7.43 ± 52.9
27	2 ± 14.07	2.9e5 ± 21.1e5	5.4 ± 39.5	3.6e4 ± 26.2e4	5.3e1 ± 38.6e1	6.14 ± 44.9
28	2 ± 14.07	3.3e5 ± 23.2e5	5.7 ± 40.2	3.1e4 ± 21.8e4	4.3e1 ± 30.6e1	1.09e1 ± 7.9e1
29	2 ± 14.07	2.9e5 ± 20.9e5	4.7 ± 33.7	2.2e4 ± 15.9e4	2.9e1 ± 21.1e1	1.18e1 ± 8.4e1
30	2 ± 14.07	4.5e5 ± 33.5e5	6.7 ± 50.3	5.1e4 ± 39.9e4	6.7e1 ± 52.1e1	1.02e1 ± 7.2e1
31	2 ± 14.07	4.9e5 ± 36.5e5	6.9 ± 51.2	4.6e4 ± 33.7e4	5.7e1 ± 42e1	1.5e1 ± 11.3e1
32	2 ± 14.07	5.3e5 ± 38e5	6.9 ± 49.8	4.9e4 ± 34.3e4	5.8e1 ± 40.8e1	1.59e1 ± 11.8e1
33	2 ± 14.07	6.2e5 ± 44.5e5	7.6 ± 54.7	6e4 ± 43.6e4	6.9e1 ± 50.1e1	1.65e1 ± 11.9e1
34	2 ± 14.07	5.2e5 ± 36.5e5	6 ± 42.2	3.6e4 ± 28e4	4.1e1 ± 31.7e1	1.86e1 ± 13.4e1
35	2 ± 14.07	5.1e5 ± 36.2e5	5.6 ± 39.4	4.4e4 ± 30.7e4	4.7e1 ± 33.3e1	1.51e1 ± 10.6e1
36	2 ± 14.07	4.8e5 ± 34.3e5	4.9 ± 35.3	3.8e4 ± 29.9e4	4e1 ± 31.8e1	1.45e1 ± 10.2e1
37	2 ± 14.07	8.1e5 ± 57.3e5	7.9 ± 55.7	9.8e4 ± 70e4	1e2 ± 7.3e2	1.29e1 ± 9.3e1

The average of the prevalence, the CFU-values per square meter (cfu/sqm) and per gram manure are calculated over all iterations. The CFU values in the intestine as well as the excreted and ingested quantities are obtained by calculating the average over all animals in one iteration at first and then over all iterations.

Table S3b (log_{10} CFU). Calculated numeric values for the scenario when chicks are set positive with 10^1 CFU bacteria per chick on day 3. Setting. 20 chicks, 4 seeder, 16 sentinels, Ross 308, target weight 2 kg, feeding duration 36 days, stocking density 39 kg/m2, 1000 g litter/m2

day	prevalence in %	cfu/sqm	cfu/g manure	cfu in intestine	cfu/g feces	cfu ingested per animal
	(% mean ± sd)	(log10 mean ± sd)	(log10 mean ± sd)	(log10 mean ± sd)	(log10 mean ± sd)	(log10 mean ± sd)
1	0 ± 0	-Inf ± -Inf	-Inf ± -Inf	-Inf ± -Inf	-Inf ± -Inf	-Inf ± 0
2	0 ± 0	-Inf ± -Inf	-Inf ± -Inf	-Inf ± -Inf	-Inf ± -Inf	-Inf ± 0
3	0 ± 0	-Inf ± -Inf	-Inf ± -Inf	-Inf ± -Inf	-Inf ± -Inf	0.3 ± 0
4	11.65 ± 4.66	3.73 ± 3.95	0.36 ± 0.57	2.97 ± 3.18	0.97 ± 1.18	-Inf ± 0
5	11.15 ± 13.16	3.8 ± 3.98	0.32 ± 0.49	2.79 ± 3.04	0.72 ± 0.98	-0.28 ± 0.04
6	5.85 ± 11.24	3.71 ± 3.98	0.15 ± 0.42	2.52 ± 3.01	0.54 ± 1.03	-0.28 ± 0.06
7	2.15 ± 12.62	3.58 ± 4.13	-0.07 ± 0.48	2.35 ± 3.21	0.17 ± 1.03	-0.43 ± 0.05
8	2.1 ± 12.8	3.6 ± 4.31	-0.14 ± 0.57	2.54 ± 3.37	0.31 ± 1.14	-0.64 ± 0.17
9	2 ± 13.08	3.73 ± 4.57	-0.09 ± 0.75	2.76 ± 3.66	0.49 ± 1.38	-0.52 ± 0.31
10	2.35 ± 13.92	3.79 ± 4.64	-0.11 ± 0.74	2.77 ± 3.63	0.42 ± 1.28	-0.35 ± 0.53
11	1.9 ± 13.39	3.84 ± 4.69	-0.14 ± 0.72	2.81 ± 3.67	0.41 ± 1.26	-0.31 ± 0.57
12	1.95 ± 13.72	3.7 ± 4.56	-0.34 ± 0.52	2.43 ± 3.31	-0.02 ± 0.86	-0.27 ± 0.59
13	1.9 ± 13.39	3.75 ± 4.62	-0.35 ± 0.52	2.73 ± 3.61	0.26 ± 1.14	-0.46 ± 0.42
14	2 ± 14.07	4.01 ± 4.86	-0.16 ± 0.69	3.1 ± 3.95	0.58 ± 1.43	-0.42 ± 0.46
15	2 ± 14.07	4.04 ± 4.89	-0.18 ± 0.67	3.01 ± 3.86	0.45 ± 1.3	-0.18 ± 0.67
16	2 ± 14.07	4 ± 4.85	-0.28 ± 0.57	2.88 ± 3.74	0.31 ± 1.17	-0.17 ± 0.67
17	2 ± 14.07	4.33 ± 5.18	0 ± 0.85	3.45 ± 4.31	0.84 ± 1.7	-0.24 ± 0.61
18	2 ± 14.07	4.44 ± 5.29	0.06 ± 0.91	3.45 ± 4.31	0.81 ± 1.67	0.07 ± 0.92
19	2 ± 14.07	4.68 ± 5.53	0.26 ± 1.11	3.77 ± 4.62	1.11 ± 1.96	0.16 ± 1.01
20	2 ± 14.07	4.84 ± 5.69	0.37 ± 1.22	3.89 ± 4.74	1.21 ± 2.06	0.39 ± 1.23
21	2 ± 14.07	4.87 ± 5.72	0.36 ± 1.21	3.83 ± 4.68	1.13 ± 1.98	0.53 ± 1.38
22	2 ± 14.07	4.9 ± 5.75	0.35 ± 1.2	3.85 ± 4.71	1.13 ± 1.98	0.54 ± 1.39
23	2 ± 14.07	5 ± 5.85	0.41 ± 1.26	4.02 ± 4.88	1.27 ± 2.13	0.55 ± 1.4
24	2 ± 14.07	5.14 ± 5.99	0.52 ± 1.36	4.18 ± 5.03	1.4 ± 2.25	0.64 ± 1.49
25	2 ± 14.07	5.27 ± 6.12	0.61 ± 1.46	4.3 ± 5.16	1.5 ± 2.36	0.76 ± 1.61
26	2 ± 14.07	5.2 ± 6.06	0.51 ± 1.37	4.05 ± 5.03	1.25 ± 2.22	0.87 ± 1.72
27	2 ± 14.07	5.46 ± 6.32	0.74 ± 1.6	4.56 ± 5.42	1.73 ± 2.59	0.79 ± 1.65
28	2 ± 14.07	5.51 ± 6.37	0.75 ± 1.6	4.49 ± 5.34	1.64 ± 2.49	1.04 ± 1.9
29	2 ± 14.07	5.47 ± 6.32	0.67 ± 1.53	4.35 ± 5.2	1.47 ± 2.32	1.07 ± 1.92
30	2 ± 14.07	5.65 ± 6.53	0.83 ± 1.7	4.71 ± 5.6	1.82 ± 2.72	1.01 ± 1.86
31	2 ± 14.07	5.69 ± 6.56	0.84 ± 1.71	4.66 ± 5.53	1.75 ± 2.62	1.17 ± 2.05
32	2 ± 14.07	5.72 ± 6.58	0.84 ± 1.7	4.69 ± 5.54	1.76 ± 2.61	1.2 ± 2.07
33	2 ± 14.07	5.79 ± 6.65	0.88 ± 1.74	4.78 ± 5.64	1.84 ± 2.7	1.22 ± 2.07
34	2 ± 14.07	5.71 ± 6.56	0.78 ± 1.63	4.55 ± 5.45	1.61 ± 2.5	1.27 ± 2.13
35	2 ± 14.07	5.71 ± 6.56	0.75 ± 1.6	4.64 ± 5.49	1.68 ± 2.52	1.18 ± 2.03
36	2 ± 14.07	5.68 ± 6.54	0.69 ± 1.55	4.57 ± 5.48	1.6 ± 2.5	1.16 ± 2.01
37	2 ± 14.07	5.91 ± 6.76	0.9 ± 1.75	4.99 ± 5.84	2.01 ± 2.86	1.11 ± 1.97

The average of the prevalence, the CFU-values per square meter (cfu/sqm) and per gram manure are calculated over all iterations. The CFU values in the intestine as well as the excreted and ingested quantities are obtained by calculating the average over all animals in one iteration at first and then over all iterations. The decadic logarithm of zero or negative values is negative infinite (-Inf).

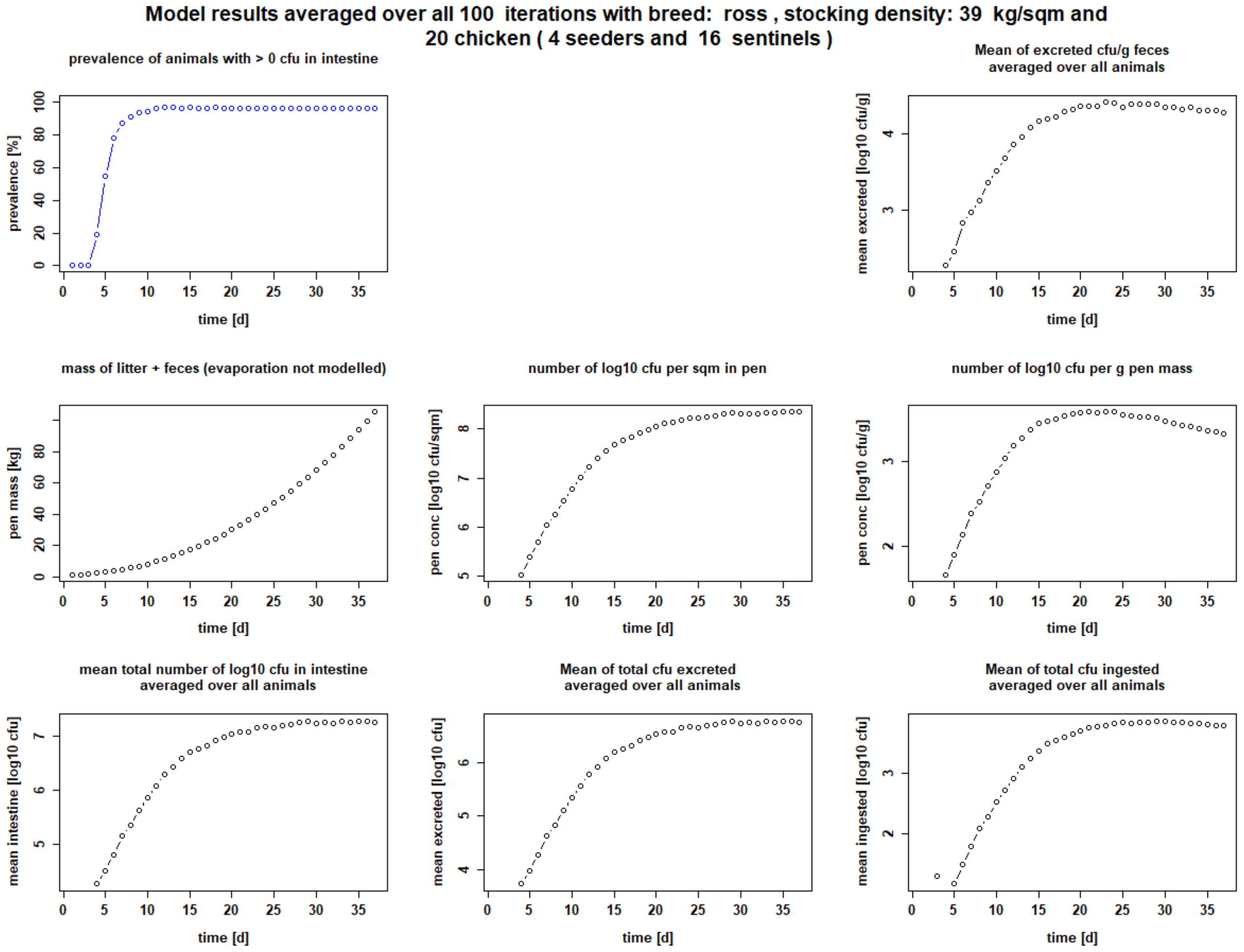

Figure S4. Calculated infection dynamic for the scenario when chicks are set positive with 10^2 CFU bacteria per chick on day 3. Dotplots with results for 90 chicks, 18 seeder, 72 sentinels, Ross 308, target weight 2.332 kg, feeding duration 36 days, stocking density 39 kg/m^2, 1000 g litter/m^2

Table S4a (CFU). Calculated numeric values for the scenario when chicks are set positive with 10^2 CFU bacteria per chick on day 3. Setting: 20 chicks, 4 seeder, 16 sentinels, Ross 308, target weight 2 kg, feeding duration 21 days, stocking density 39 kg/m2, 1000 g litter/m2

day	prevalence in %	cfu/sqm	cfu/g manure	cfu in intestine	cfu/g feces	cfu ingested per animal
	(mean ± sd)	(mean ± sd)	(mean ± sd)	(mean ± sd)	(mean ± sd)	(mean ± sd)
1	0 ± 0	0e0 ± 0	0e0 ± 0	0e0 ± 0	0e0 ± 0	0e0 ± 0
2	0 ± 0	0e0 ± 0	0e0 ± 0	0e0 ± 0	0e0 ± 0	0e0 ± 0
3	0 ± 0	0e0 ± 0	0e0 ± 0	0e0 ± 0	0e0 ± 0	2e1 ± 0e1
4	19.25 ± 1.93	1.1e5 ± 1.4e5	4.6e1 ± 6.1e1	1.8e4 ± 2.4e4	1.8e2 ± 2.4e2	0e0 ± 0
5	54.6 ± 35.63	2.4e5 ± 2.9e5	8e1 ± 9.4e1	3.2e4 ± 3.9e4	2.8e2 ± 3.4e2	1.49e1 ± 2e1
6	78.25 ± 28.84	5e5 ± 6.1e5	1.4e2 ± 1.7e2	6.4e4 ± 8.3e4	6.8e2 ± 8.7e2	3.09e1 ± 3.7e1
7	87.55 ± 24.96	1.1e6 ± 1.5e6	2.4e2 ± 3.4e2	1.4e5 ± 2.1e5	9.4e2 ± 14.2e2	6.16e1 ± 7.6e1
8	90.9 ± 23.17	1.8e6 ± 2.8e6	3.4e2 ± 5.1e2	2.2e5 ± 3.6e5	1.3e3 ± 2.1e3	1.21e2 ± 1.7e2
9	93.6 ± 18.96	3.4e6 ± 5.1e6	5.2e2 ± 7.8e2	4.3e5 ± 6.7e5	2.3e3 ± 3.5e3	1.89e2 ± 2.8e2
10	94.75 ± 19.02	6e6 ± 9.2e6	7.6e2 ± 11.6e2	7.3e5 ± 11.7e5	3.3e3 ± 5.3e3	3.29e2 ± 4.9e2
11	96.1 ± 16.29	1e7 ± 1.8e7	1.1e3 ± 1.9e3	1.2e6 ± 2.3e6	4.8e3 ± 9.1e3	5.2e2 ± 8e2
12	96.95 ± 14.67	1.7e7 ± 3e7	1.5e3 ± 2.7e3	2e6 ± 3.8e6	7.3e3 ± 13.6e3	8.21e2 ± 14.2e2
13	96.8 ± 14.69	2.5e7 ± 4.1e7	1.9e3 ± 3.2e3	2.8e6 ± 4.6e6	9.2e3 ± 15.2e3	1.25e3 ± 2.2e3
14	96.7 ± 15.94	3.6e7 ± 5.6e7	2.4e3 ± 3.8e3	4e6 ± 6.3e6	1.2e4 ± 1.9e4	1.7e3 ± 2.8e3
15	97.15 ± 14.67	4.8e7 ± 6.6e7	2.9e3 ± 3.9e3	5.2e6 ± 6.8e6	1.5e4 ± 1.9e4	2.34e3 ± 3.7e3
16	96.6 ± 16.76	5.8e7 ± 7.3e7	3.1e3 ± 3.9e3	5.7e6 ± 7.6e6	1.6e4 ± 2.1e4	2.99e3 ± 4.1e3
17	96.35 ± 18.02	6.8e7 ± 7.8e7	3.2e3 ± 3.7e3	6.7e6 ± 7.6e6	1.7e4 ± 1.9e4	3.38e3 ± 4.3e3
18	97 ± 15.08	8.4e7 ± 8.9e7	3.5e3 ± 3.8e3	8.5e6 ± 9.3e6	1.9e4 ± 2.1e4	3.82e3 ± 4.4e3
19	96.65 ± 16.58	9.8e7 ± 9.3e7	3.7e3 ± 3.5e3	9.6e6 ± 8.9e6	2.1e4 ± 2e4	4.42e3 ± 4.7e3
20	96.6 ± 16.76	1.1e8 ± 1e8	3.9e3 ± 3.5e3	1.1e7 ± 1e7	2.3e4 ± 2.2e4	4.96e3 ± 4.7e3
21	96.5 ± 17.27	1.3e8 ± 1e8	3.9e3 ± 3.1e3	1.2e7 ± 0.9e7	2.3e4 ± 1.9e4	5.52e3 ± 5e3
22	96.55 ± 17.02	1.3e8 ± 1e8	3.8e3 ± 2.8e3	1.2e7 ± 0.9e7	2.3e4 ± 1.8e4	5.9e3 ± 4.7e3
23	96.45 ± 17.51	1.5e8 ± 1e8	3.9e3 ± 2.7e3	1.4e7 ± 1e7	2.6e4 ± 1.8e4	6.08e3 ± 4.5e3
24	96.5 ± 17.25	1.6e8 ± 1e8	3.9e3 ± 2.4e3	1.5e7 ± 1e7	2.5e4 ± 1.6e4	6.61e3 ± 4.5e3
25	96.4 ± 17.75	1.7e8 ± 1e8	3.6e3 ± 2.2e3	1.4e7 ± 1e7	2.3e4 ± 1.5e4	6.84e3 ± 4.2e3
26	96.55 ± 17.02	1.7e8 ± 1e8	3.5e3 ± 2e3	1.6e7 ± 0.9e7	2.5e4 ± 1.5e4	6.63e3 ± 4e3
27	96.35 ± 18	1.8e8 ± 1e8	3.5e3 ± 1.8e3	1.7e7 ± 1e7	2.5e4 ± 1.4e4	6.74e3 ± 3.7e3
28	96.4 ± 17.75	2e8 ± 1e8	3.4e3 ± 1.7e3	1.8e7 ± 1e7	2.5e4 ± 1.4e4	6.92e3 ± 3.6e3
29	96.4 ± 17.74	2.1e8 ± 1e8	3.3e3 ± 1.6e3	1.9e7 ± 1e7	2.5e4 ± 1.3e4	7.12e3 ± 3.5e3
30	96.25 ± 18.48	2e8 ± 1e8	3.1e3 ± 1.4e3	1.7e7 ± 1e7	2.2e4 ± 1.3e4	7.2e3 ± 3.4e3
31	96.5 ± 17.25	2.1e8 ± 0.9e8	2.9e3 ± 1.3e3	1.8e7 ± 0.9e7	2.2e4 ± 1.1e4	6.85e3 ± 3.2e3
32	96.4 ± 17.74	2.1e8 ± 0.9e8	2.7e3 ± 1.1e3	1.8e7 ± 0.9e7	2.1e4 ± 1.1e4	6.67e3 ± 2.9e3
33	96.4 ± 17.75	2.2e8 ± 0.9e8	2.7e3 ± 1.1e3	1.9e7 ± 1e7	2.2e4 ± 1.1e4	6.45e3 ± 2.7e3
34	96.15 ± 19	2.1e8 ± 0.9e8	2.5e3 ± 1e3	1.8e7 ± 0.9e7	2e4 ± 1e4	6.52e3 ± 2.6e3
35	96.25 ± 18.48	2.2e8 ± 0.9e8	2.4e3 ± 1e3	1.9e7 ± 1e7	2.1e4 ± 1.1e4	6.23e3 ± 2.5e3
36	96.25 ± 18.51	2.2e8 ± 0.9e8	2.3e3 ± 0.9e3	1.9e7 ± 0.9e7	2.1e4 ± 1e4	6.14e3 ± 2.5e3
37	96.3 ± 18.25	2.2e8 ± 0.9e8	2.1e3 ± 0.8e3	1.8e7 ± 0.9e7	1.9e4 ± 1e4	6.04e3 ± 2.4e3

The average of the prevalence, the CFU-values per square meter (cfu/sqm) and per gram manure are calculated over all iterations. The CFU values in the intestine as well as the excreted and ingested quantities are obtained by calculating the average over all animals in one iteration at first and then over all iterations.

Table S4b (log_{10} CFU). Calculated numeric values for the scenario when chicks are set positive with 10^2 CFU bacteria per chick on day 3. Setting: 20 chicks, 4 seeder, 16 sentinels, Ross 308, target weight 2 kg, feeding duration 21 days, stocking density 39 kg/m2, 1000 g litter/m2

day	prevalence in %	cfu/sqm	cfu/g manure	cfu in intestine	cfu/g feces	cfu ingested per animal
	(% mean ± sd)	(log10 mean ± sd)	(log10 mean ± sd)	(log10 mean ± sd)	(log10 mean ± sd)	(log10 mean ± sd)
1	0 ± 0	-Inf ± -Inf	-Inf ± -Inf	-Inf ± -Inf	-Inf ± -Inf	-Inf ± 0
2	0 ± 0	-Inf ± -Inf	-Inf ± -Inf	-Inf ± -Inf	-Inf ± -Inf	-Inf ± 0
3	0 ± 0	-Inf ± -Inf	-Inf ± -Inf	-Inf ± -Inf	-Inf ± -Inf	1.3 ± 0
4	19.25 ± 1.93	5.03 ± 5.16	1.66 ± 1.78	4.27 ± 4.39	2.26 ± 2.39	-Inf ± 0
5	54.6 ± 35.63	5.39 ± 5.46	1.9 ± 1.97	4.51 ± 4.6	2.44 ± 2.53	1.17 ± 1.31
6	78.25 ± 28.84	5.7 ± 5.78	2.14 ± 2.23	4.81 ± 4.92	2.83 ± 2.94	1.49 ± 1.57
7	87.55 ± 24.96	6.03 ± 6.18	2.38 ± 2.53	5.15 ± 5.33	2.97 ± 3.15	1.79 ± 1.88
8	90.9 ± 23.17	6.27 ± 6.44	2.53 ± 2.71	5.35 ± 5.55	3.12 ± 3.32	2.08 ± 2.23
9	93.6 ± 18.96	6.54 ± 6.71	2.72 ± 2.89	5.63 ± 5.83	3.36 ± 3.55	2.28 ± 2.45
10	94.75 ± 19.02	6.78 ± 6.96	2.88 ± 3.07	5.86 ± 6.07	3.52 ± 3.72	2.52 ± 2.69
11	96.1 ± 16.29	7.01 ± 7.24	3.04 ± 3.27	6.09 ± 6.36	3.69 ± 3.96	2.72 ± 2.9
12	96.95 ± 14.67	7.23 ± 7.48	3.19 ± 3.44	6.31 ± 6.58	3.86 ± 4.13	2.91 ± 3.15
13	96.8 ± 14.69	7.39 ± 7.61	3.29 ± 3.51	6.44 ± 6.66	3.96 ± 4.18	3.1 ± 3.35
14	96.7 ± 15.94	7.55 ± 7.74	3.38 ± 3.58	6.6 ± 6.8	4.08 ± 4.28	3.23 ± 3.45
15	97.15 ± 14.67	7.68 ± 7.82	3.46 ± 3.59	6.72 ± 6.83	4.17 ± 4.28	3.37 ± 3.56
16	96.6 ± 16.76	7.76 ± 7.86	3.48 ± 3.59	6.76 ± 6.88	4.19 ± 4.31	3.48 ± 3.61
17	96.35 ± 18.02	7.83 ± 7.89	3.51 ± 3.57	6.83 ± 6.88	4.22 ± 4.28	3.53 ± 3.63
18	97 ± 15.08	7.92 ± 7.95	3.54 ± 3.57	6.93 ± 6.97	4.29 ± 4.33	3.58 ± 3.64
19	96.65 ± 16.58	7.99 ± 7.97	3.57 ± 3.54	6.98 ± 6.95	4.32 ± 4.29	3.65 ± 3.67
20	96.6 ± 16.76	8.05 ± 8.01	3.59 ± 3.54	7.04 ± 7.01	4.36 ± 4.33	3.7 ± 3.67
21	96.5 ± 17.27	8.1 ± 8	3.59 ± 3.49	7.07 ± 6.98	4.37 ± 4.27	3.74 ± 3.7
22	96.55 ± 17.02	8.13 ± 8	3.58 ± 3.45	7.09 ± 6.98	4.37 ± 4.26	3.77 ± 3.67
23	96.45 ± 17.51	8.18 ± 8.01	3.6 ± 3.43	7.16 ± 7	4.41 ± 4.26	3.78 ± 3.66
24	96.5 ± 17.25	8.22 ± 8.01	3.59 ± 3.38	7.18 ± 6.99	4.4 ± 4.21	3.82 ± 3.65
25	96.4 ± 17.75	8.22 ± 8	3.56 ± 3.34	7.15 ± 6.98	4.36 ± 4.18	3.83 ± 3.63
26	96.55 ± 17.02	8.24 ± 7.99	3.55 ± 3.29	7.19 ± 6.97	4.39 ± 4.17	3.82 ± 3.6
27	96.35 ± 18	8.27 ± 7.99	3.54 ± 3.26	7.22 ± 6.98	4.39 ± 4.15	3.83 ± 3.57
28	96.4 ± 17.75	8.29 ± 7.99	3.53 ± 3.22	7.25 ± 6.98	4.4 ± 4.13	3.84 ± 3.56
29	96.4 ± 17.74	8.32 ± 7.99	3.52 ± 3.2	7.27 ± 7.01	4.39 ± 4.13	3.85 ± 3.54
30	96.25 ± 18.48	8.31 ± 7.98	3.49 ± 3.16	7.23 ± 6.99	4.35 ± 4.11	3.86 ± 3.53
31	96.5 ± 17.25	8.31 ± 7.95	3.46 ± 3.1	7.25 ± 6.96	4.35 ± 4.06	3.84 ± 3.51
32	96.4 ± 17.74	8.32 ± 7.94	3.43 ± 3.05	7.25 ± 6.96	4.32 ± 4.04	3.82 ± 3.46
33	96.4 ± 17.75	8.33 ± 7.94	3.42 ± 3.03	7.29 ± 6.99	4.35 ± 4.05	3.81 ± 3.43
34	96.15 ± 19	8.33 ± 7.93	3.39 ± 3	7.26 ± 6.95	4.31 ± 4.01	3.81 ± 3.42
35	96.25 ± 18.48	8.34 ± 7.95	3.37 ± 2.98	7.28 ± 7	4.31 ± 4.03	3.79 ± 3.4
36	96.25 ± 18.51	8.35 ± 7.95	3.36 ± 2.96	7.29 ± 6.97	4.31 ± 4	3.79 ± 3.4
37	96.3 ± 18.25	8.34 ± 7.94	3.33 ± 2.92	7.27 ± 6.96	4.28 ± 3.98	3.78 ± 3.39

The average of the prevalence, the CFU-values per square meter (cfu/sqm) and per gram manure are calculated over all iterations. The CFU values in the intestine as well as the excreted and ingested quantities are obtained by calculating the average over all animals in one iteration at first and then over all iterations. The decadic logarithm of zero or negative values is negative infinite (-Inf).

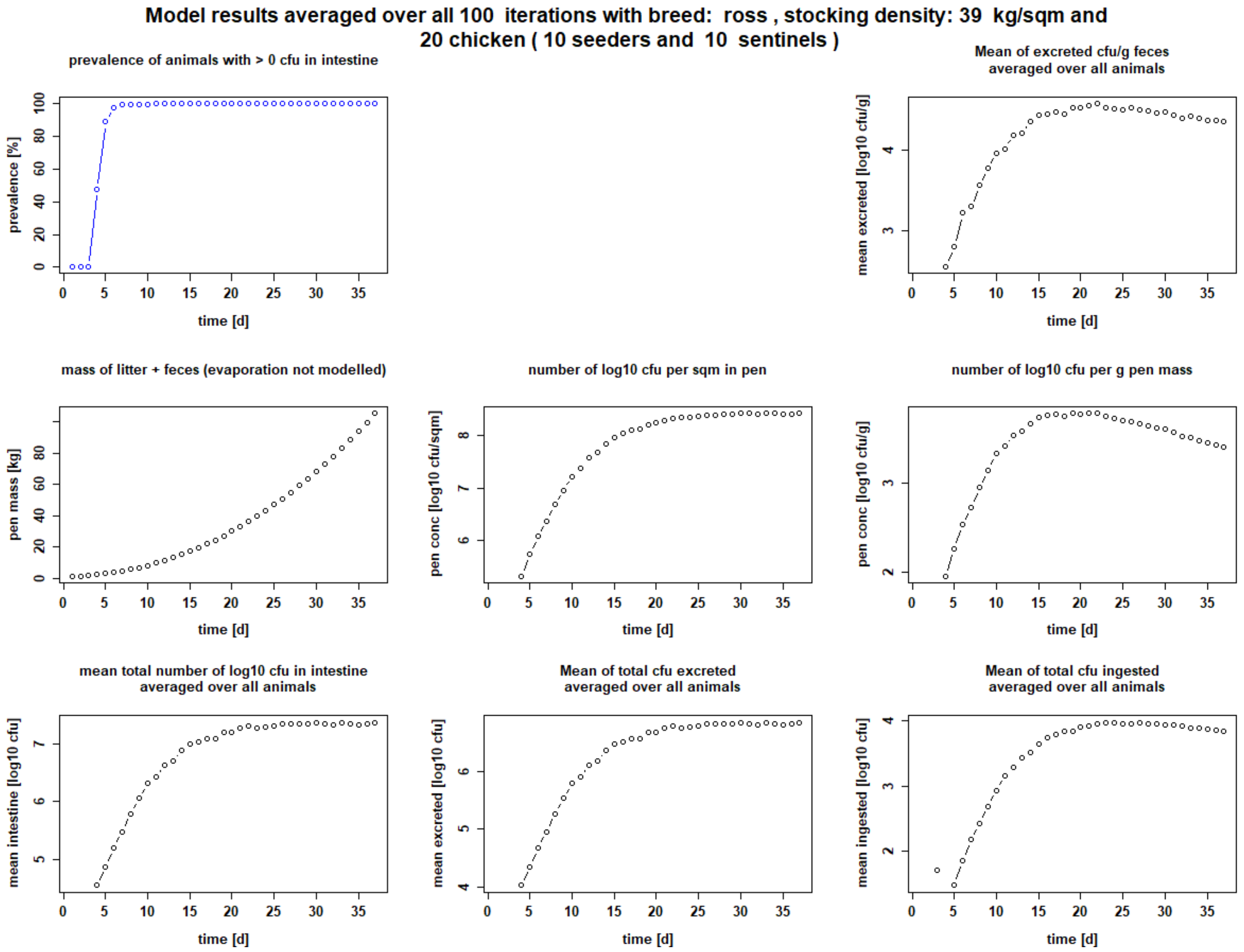

Figure S5. Calculated infection dynamic for the scenario when day-old chicks are set positive (10^2 CFU bacteria per chick on day 3). Dotplots with results for 20 chicks, 10 seeder, 10 sentinels, Ross 308, target weight 2 kg, feeding duration 36 days, stocking density 39 kg/m^2, 1000 g litter/m^2

Table S5a (CFU). Calculated numeric values for the scenario when chicks are set positive with 10^2 CFU bacteria per chick on day 3. Setting: 20 chicks, 10 seeder, 10 sentinels, Ross 308, target weight 2 kg, feeding duration 36 days, stocking density 39 kg/m^2, 1000 g litter/m^2

day	prevalence in %	cfu/sqm	cfu/g manure	cfu in intestine	cfu/g feces	cfu ingested per animal
	(mean ± sd)	(mean ± sd)	(mean ± sd)	(mean ± sd)	(mean ± sd)	(mean ± sd)
1	0 ± 0	0e0 ± 0	0e0 ± 0	0e0 ± 0	0e0 ± 0	0e0 ± 0
2	0 ± 0	0e0 ± 0	0e0 ± 0	0e0 ± 0	0e0 ± 0	0e0 ± 0
3	0 ± 0	0e0 ± 0	0e0 ± 0	0e0 ± 0	0e0 ± 0	5e1 ± 0e1
4	47.75 ± 2.79	2.1e5 ± 1.7e5	8.9e1 ± 7e1	3.6e4 ± 2.8e4	3.6e2 ± 2.8e2	0e0 ± 0
5	89.1 ± 15.46	5.5e5 ± 4.3e5	1.8e2 ± 1.4e2	7.6e4 ± 6.3e4	6.5e2 ± 5.4e2	2.95e1 ± 2.4e1
6	97.75 ± 6.29	1.2e6 ± 1.3e6	3.4e2 ± 3.6e2	1.6e5 ± 1.9e5	1.7e3 ± 2e3	7.03e1 ± 5.6e1
7	99.6 ± 2.09	2.4e6 ± 2.6e6	5.3e2 ± 5.7e2	3e5 ± 3.5e5	2e3 ± 2.3e3	1.5e2 ± 1.6e2
8	99.85 ± 1.11	4.8e6 ± 5.7e6	8.8e2 ± 10.5e2	6.2e5 ± 8e5	3.7e3 ± 4.7e3	2.66e2 ± 2.9e2
9	99.95 ± 0.5	9e6 ± 12.5e6	1.4e3 ± 1.9e3	1.1e6 ± 1.7e6	6e3 ± 9.1e3	4.93e2 ± 5.9e2
10	99.95 ± 0.5	1.7e7 ± 2.4e7	2.1e3 ± 3.1e3	2.1e6 ± 3.2e6	9.2e3 ± 14.5e3	8.64e2 ± 12e2
11	100 ± 0	2.4e7 ± 3.2e7	2.5e3 ± 3.4e3	2.7e6 ± 3.6e6	1e4 ± 1.4e4	1.44e3 ± 2.1e3
12	100 ± 0	3.7e7 ± 4.6e7	3.4e3 ± 4.2e3	4.3e6 ± 5.6e6	1.5e4 ± 2e4	1.92e3 ± 2.6e3
13	100 ± 0	4.8e7 ± 4.9e7	3.7e3 ± 3.9e3	5e6 ± 5.1e6	1.7e4 ± 1.7e4	2.73e3 ± 3.4e3
14	100 ± 0	6.8e7 ± 6.3e7	4.6e3 ± 4.3e3	7.5e6 ± 7.2e6	2.3e4 ± 2.2e4	3.29e3 ± 3.4e3
15	100 ± 0	9.1e7 ± 7.7e7	5.4e3 ± 4.6e3	9.8e6 ± 8.4e6	2.7e4 ± 2.3e4	4.46e3 ± 4.2e3
16	100 ± 0	1.1e8 ± 0.8e8	5.7e3 ± 4.3e3	1.1e7 ± 0.8e7	2.9e4 ± 2.1e4	5.63e3 ± 4.8e3
17	100 ± 0	1.2e8 ± 0.8e8	5.8e3 ± 3.8e3	1.2e7 ± 0.8e7	3e4 ± 2e4	6.26e3 ± 4.7e3
18	100 ± 0	1.3e8 ± 0.8e8	5.6e3 ± 3.3e3	1.2e7 ± 0.8e7	2.8e4 ± 1.9e4	6.9e3 ± 4.5e3
19	100 ± 0	1.6e8 ± 0.9e8	6e3 ± 3.3e3	1.6e7 ± 0.9e7	3.4e4 ± 2e4	7.05e3 ± 4.2e3
20	100 ± 0	1.7e8 ± 0.9e8	5.9e3 ± 3e3	1.6e7 ± 0.9e7	3.3e4 ± 2e4	8.02e3 ± 4.4e3
21	100 ± 0	1.9e8 ± 0.9e8	6e3 ± 2.7e3	1.8e7 ± 0.9e7	3.6e4 ± 1.9e4	8.38e3 ± 4.2e3
22	100 ± 0	2.1e8 ± 1e8	6e3 ± 2.7e3	2e7 ± 1.1e7	3.8e4 ± 2.1e4	9.04e3 ± 4.1e3
23	100 ± 0	2.2e8 ± 0.9e8	5.6e3 ± 2.3e3	1.9e7 ± 1e7	3.4e4 ± 1.7e4	9.58e3 ± 4.4e3
24	100 ± 0	2.2e8 ± 0.8e8	5.3e3 ± 1.9e3	2e7 ± 0.9e7	3.2e4 ± 1.5e4	9.38e3 ± 3.9e3
25	100 ± 0	2.3e8 ± 0.8e8	5e3 ± 1.7e3	2e7 ± 0.9e7	3.2e4 ± 1.4e4	9.23e3 ± 3.3e3
26	100 ± 0	2.4e8 ± 0.8e8	4.8e3 ± 1.5e3	2.1e7 ± 0.9e7	3.4e4 ± 1.4e4	9.16e3 ± 3.1e3
27	100 ± 0	2.4e8 ± 0.7e8	4.6e3 ± 1.3e3	2.1e7 ± 0.9e7	3.1e4 ± 1.3e4	9.28e3 ± 3e3
28	100 ± 0	2.5e8 ± 0.8e8	4.3e3 ± 1.4e3	2.2e7 ± 0.9e7	3.1e4 ± 1.3e4	9.16e3 ± 2.7e3
29	100 ± 0	2.5e8 ± 0.7e8	4.1e3 ± 1.2e3	2.2e7 ± 0.8e7	2.9e4 ± 1.1e4	9.05e3 ± 2.8e3
30	100 ± 0	2.6e8 ± 0.8e8	3.9e3 ± 1.2e3	2.3e7 ± 1e7	3e4 ± 1.2e4	8.82e3 ± 2.5e3
31	100 ± 0	2.6e8 ± 0.7e8	3.6e3 ± 1e3	2.2e7 ± 0.8e7	2.7e4 ± 1e4	8.82e3 ± 2.6e3
32	100 ± 0	2.5e8 ± 0.7e8	3.3e3 ± 0.9e3	2.1e7 ± 0.9e7	2.5e4 ± 1.1e4	8.39e3 ± 2.2e3
33	100 ± 0	2.6e8 ± 0.7e8	3.2e3 ± 0.9e3	2.3e7 ± 1e7	2.6e4 ± 1.2e4	7.91e3 ± 2.2e3
34	100 ± 0	2.6e8 ± 0.7e8	3e3 ± 0.8e3	2.2e7 ± 0.9e7	2.5e4 ± 1e4	7.87e3 ± 2.2e3
35	100 ± 0	2.5e8 ± 0.6e8	2.8e3 ± 0.6e3	2.1e7 ± 0.8e7	2.3e4 ± 0.8e4	7.51e3 ± 2e3
36	100 ± 0	2.6e8 ± 0.6e8	2.6e3 ± 0.7e3	2.2e7 ± 0.9e7	2.3e4 ± 0.9e4	7.15e3 ± 1.6e3
37	100 ± 0	2.6e8 ± 0.6e8	2.5e3 ± 0.6e3	2.2e7 ± 0.9e7	2.3e4 ± 0.9e4	6.95e3 ± 1.8e3

The average of the prevalence, the CFU-values per square meter (cfu/sqm) and per gram manure are calculated over all iterations. The CFU values in the intestine as well as the excreted and ingested quantities are obtained by calculating the average over all animals in one iteration at first and then over all iterations.

Table S5b (log_{10} CFU). Calculated numeric values for the scenario when chicks are set positive with 10^2 CFU bacteria per chick on day 3. Setting: 20 chicks, 10 seeder, 10 sentinels, Ross 308, target weight 2 kg, feeding duration 36 days, stocking density 39 kg/m^2, 1000 g litter/m^2

day	prevalence in %	cfu/sqm	cfu/g manure	cfu in intestine	cfu/g feces	cfu ingested per animal
	(% mean ± sd)	(log10 mean ± sd)	(log10 mean ± sd)	(log10 mean ± sd)	(log10 mean ± sd)	(log10 mean ± sd)
1	0 ± 0	-Inf ± -Inf	-Inf ± -Inf	-Inf ± -Inf	-Inf ± -Inf	-Inf ± 0
2	0 ± 0	-Inf ± -Inf	-Inf ± -Inf	-Inf ± -Inf	-Inf ± -Inf	-Inf ± 0
3	0 ± 0	-Inf ± -Inf	-Inf ± -Inf	-Inf ± -Inf	-Inf ± -Inf	1.7 ± 0
4	47.75 ± 2.79	5.32 ± 5.22	1.95 ± 1.85	4.56 ± 4.45	2.56 ± 2.45	-Inf ± 0
5	89.1 ± 15.46	5.74 ± 5.64	2.26 ± 2.15	4.88 ± 4.8	2.81 ± 2.73	1.47 ± 1.37
6	97.75 ± 6.29	6.08 ± 6.11	2.53 ± 2.55	5.2 ± 5.28	3.22 ± 3.3	1.85 ± 1.75
7	99.6 ± 2.09	6.37 ± 6.41	2.72 ± 2.76	5.48 ± 5.54	3.3 ± 3.36	2.18 ± 2.2
8	99.85 ± 1.11	6.68 ± 6.76	2.94 ± 3.02	5.79 ± 5.9	3.56 ± 3.67	2.43 ± 2.46
9	99.95 ± 0.5	6.96 ± 7.1	3.14 ± 3.28	6.05 ± 6.23	3.78 ± 3.96	2.69 ± 2.77
10	99.95 ± 0.5	7.22 ± 7.39	3.32 ± 3.49	6.31 ± 6.51	3.97 ± 4.16	2.94 ± 3.08
11	100 ± 0	7.38 ± 7.51	3.41 ± 3.54	6.43 ± 6.55	4.02 ± 4.15	3.16 ± 3.33
12	100 ± 0	7.57 ± 7.66	3.53 ± 3.62	6.63 ± 6.75	4.19 ± 4.3	3.28 ± 3.41
13	100 ± 0	7.68 ± 7.69	3.57 ± 3.59	6.7 ± 6.71	4.22 ± 4.23	3.44 ± 3.53
14	100 ± 0	7.83 ± 7.8	3.66 ± 3.63	6.88 ± 6.86	4.36 ± 4.34	3.52 ± 3.53
15	100 ± 0	7.96 ± 7.89	3.74 ± 3.66	6.99 ± 6.92	4.44 ± 4.37	3.65 ± 3.62
16	100 ± 0	8.03 ± 7.91	3.75 ± 3.63	7.02 ± 6.9	4.46 ± 4.33	3.75 ± 3.68
17	100 ± 0	8.09 ± 7.91	3.76 ± 3.58	7.08 ± 6.91	4.47 ± 4.31	3.8 ± 3.67
18	100 ± 0	8.12 ± 7.9	3.75 ± 3.52	7.09 ± 6.91	4.45 ± 4.27	3.84 ± 3.65
19	100 ± 0	8.2 ± 7.94	3.78 ± 3.51	7.19 ± 6.97	4.53 ± 4.31	3.85 ± 3.62
20	100 ± 0	8.24 ± 7.94	3.77 ± 3.47	7.2 ± 6.97	4.52 ± 4.3	3.9 ± 3.64
21	100 ± 0	8.29 ± 7.95	3.78 ± 3.44	7.26 ± 6.98	4.56 ± 4.27	3.92 ± 3.63
22	100 ± 0	8.33 ± 7.99	3.78 ± 3.44	7.3 ± 7.04	4.57 ± 4.32	3.96 ± 3.62
23	100 ± 0	8.33 ± 7.95	3.75 ± 3.36	7.27 ± 6.98	4.53 ± 4.24	3.98 ± 3.64
24	100 ± 0	8.35 ± 7.9	3.72 ± 3.28	7.29 ± 6.96	4.51 ± 4.18	3.97 ± 3.59
25	100 ± 0	8.36 ± 7.89	3.7 ± 3.23	7.3 ± 6.94	4.51 ± 4.15	3.97 ± 3.52
26	100 ± 0	8.38 ± 7.88	3.68 ± 3.19	7.33 ± 6.95	4.53 ± 4.14	3.96 ± 3.5
27	100 ± 0	8.39 ± 7.86	3.66 ± 3.13	7.33 ± 6.93	4.5 ± 4.1	3.97 ± 3.47
28	100 ± 0	8.4 ± 7.9	3.64 ± 3.13	7.34 ± 6.98	4.49 ± 4.12	3.96 ± 3.43
29	100 ± 0	8.4 ± 7.86	3.61 ± 3.06	7.34 ± 6.92	4.47 ± 4.05	3.96 ± 3.45
30	100 ± 0	8.42 ± 7.89	3.6 ± 3.07	7.36 ± 6.98	4.48 ± 4.1	3.95 ± 3.4
31	100 ± 0	8.41 ± 7.84	3.56 ± 2.99	7.34 ± 6.9	4.43 ± 3.99	3.95 ± 3.42
32	100 ± 0	8.4 ± 7.85	3.52 ± 2.97	7.33 ± 6.95	4.4 ± 4.02	3.92 ± 3.35
33	100 ± 0	8.42 ± 7.87	3.51 ± 2.96	7.36 ± 7.01	4.42 ± 4.07	3.9 ± 3.35
34	100 ± 0	8.41 ± 7.83	3.47 ± 2.89	7.34 ± 6.94	4.39 ± 4	3.9 ± 3.35
35	100 ± 0	8.4 ± 7.76	3.44 ± 2.79	7.33 ± 6.88	4.36 ± 3.92	3.88 ± 3.29
36	100 ± 0	8.41 ± 7.81	3.42 ± 2.82	7.34 ± 6.95	4.37 ± 3.97	3.85 ± 3.21
37	100 ± 0	8.41 ± 7.81	3.4 ± 2.8	7.35 ± 6.95	4.36 ± 3.96	3.84 ± 3.24

The average of the prevalence, the CFU-values per square meter (cfu/sqm) and per gram manure are calculated over all iterations. The CFU values in the intestine as well as the excreted and ingested quantities are obtained by calculating the average over all animals in one iteration at first and then over all iterations. The decadic logarithm of zero or negative values is negative infinite (-Inf).

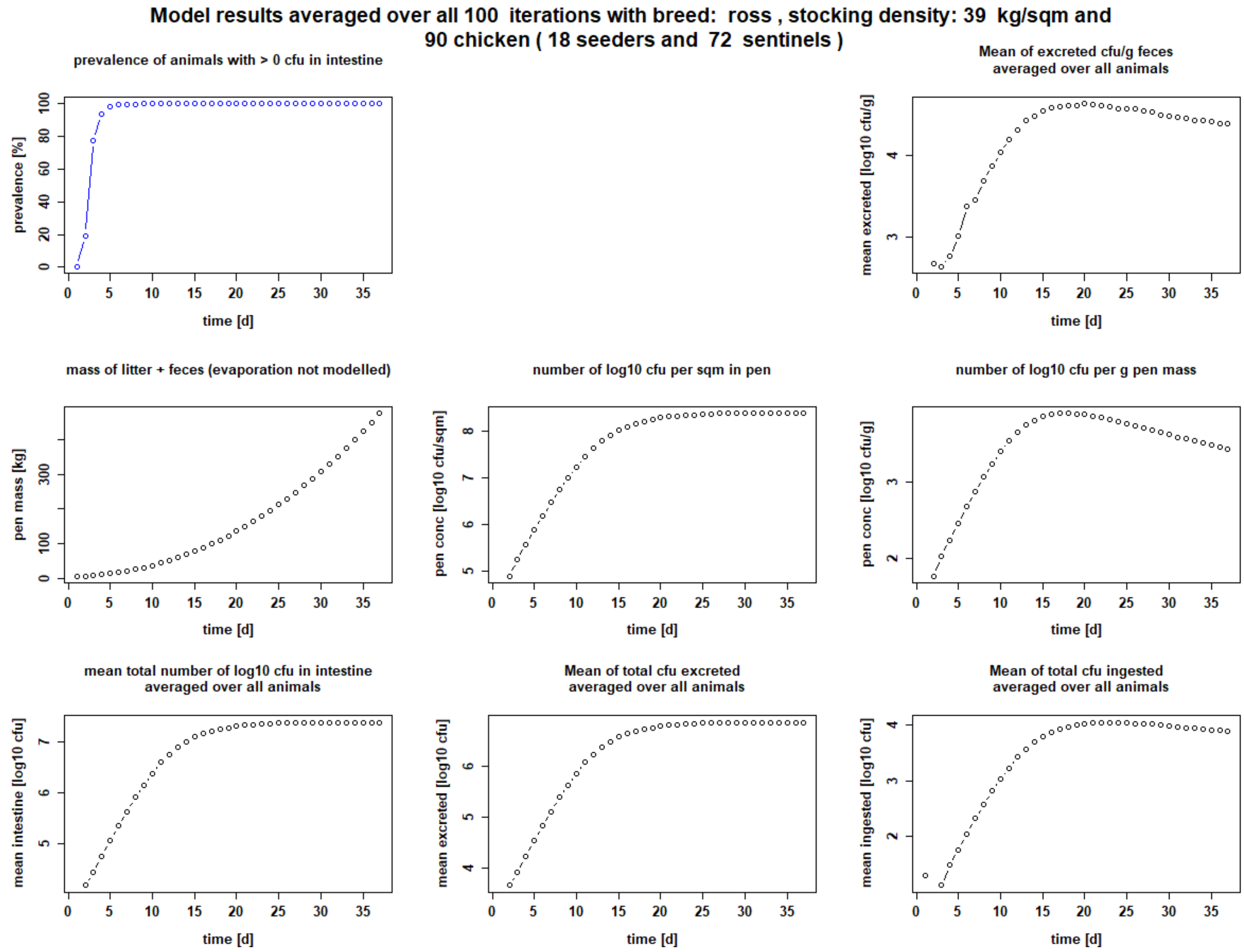

Figure S6. Calculated infection dynamic when day-old chicks are set positive (10^2 CFU bacteria per chick on day 1; reference). Dotplots with results for 90 chicks, 18 seeder, 72 sentinels, Ross 308, feeding duration 36 days, stocking density 39 kg/m^2, litter 1000g/m^2

Table S6a (CFU). Calculated numeric values for scenario when day-old chicks are set positive positive (102 CFU bacteria per chick on day 1; reference). Setting 18 seeder, 72 sentinels, Ross 308, feeding duration 36 days, stocking density 39 kg/m^2, litter 1000 g/m^2

day	prevalence in %	cfu/sqm	cfu/g manure	cfu in intestine	cfu/g feces	cfu ingested per animal
	(mean ± sd)	(mean ± sd)	(mean ± sd)	(mean ± sd)	(mean ± sd)	(mean ± sd)
1	0 ± 0	0e0 ± 0	0e0 ± 0	0e0 ± 0	0e0 ± 0	2e1 ± 0e1
2	19.23 ± 1.03	7.8e4 ± 4.9e4	5.8e1 ± 3.7e1	1.6e4 ± 1e4	4.7e2 ± 3e2	0e0 ± 0
3	77.7 ± 21.9	1.8e5 ± 1e5	1.1e2 ± 0.6e2	2.7e4 ± 1.7e4	4.3e2 ± 2.6e2	1.34e1 ± 0.9e1
4	94.03 ± 9.78	3.8e5 ± 2.3e5	1.7e2 ± 1e2	5.7e4 ± 3.6e4	5.7e2 ± 3.5e2	3.08e1 ± 1.8e1
5	98.07 ± 5.05	7.8e5 ± 4.8e5	2.8e2 ± 1.7e2	1.2e5 ± 0.7e5	1e3 ± 0.6e3	5.8e1 ± 3.5e1
6	99.38 ± 1.98	1.5e6 ± 1e6	4.7e2 ± 3.1e2	2.3e5 ± 1.5e5	2.4e3 ± 1.6e3	1.11e2 ± 0.7e2
7	99.87 ± 0.69	2.9e6 ± 2.2e6	7.4e2 ± 5.5e2	4.3e5 ± 3.5e5	2.9e3 ± 2.3e3	2.12e2 ± 1.4e2
8	99.98 ± 0.16	5.6e6 ± 4.6e6	1.2e3 ± 1e3	8.3e5 ± 7.1e5	4.9e3 ± 4.2e3	3.72e2 ± 2.8e2
9	100 ± 0	9.9e6 ± 8e6	1.7e3 ± 1.4e3	1.4e6 ± 1.2e6	7.5e3 ± 6.3e3	6.54e2 ± 5.3e2
10	100 ± 0	1.7e7 ± 1.3e7	2.5e3 ± 1.9e3	2.4e6 ± 1.9e6	1.1e4 ± 0.9e4	1.08e3 ± 0.9e3
11	100 ± 0	2.9e7 ± 2.3e7	3.5e3 ± 2.8e3	4e6 ± 3.2e6	1.6e4 ± 1.3e4	1.69e3 ± 1.3e3
12	100 ± 0	4.3e7 ± 3.1e7	4.5e3 ± 3.2e3	5.8e6 ± 4.1e6	2.1e4 ± 1.5e4	2.65e3 ± 2.1e3
13	100 ± 0	6.2e7 ± 4.1e7	5.6e3 ± 3.7e3	8e6 ± 5.3e6	2.7e4 ± 1.8e4	3.67e3 ± 2.6e3
14	100 ± 0	8.2e7 ± 4.8e7	6.4e3 ± 3.8e3	1e7 ± 0.6e7	3.1e4 ± 1.8e4	4.93e3 ± 3.2e3
15	100 ± 0	1e8 ± 0.6e8	7.1e3 ± 3.8e3	1.2e7 ± 0.7e7	3.5e4 ± 1.9e4	6.2e3 ± 3.6e3
16	100 ± 0	1.2e8 ± 0.6e8	7.6e3 ± 3.5e3	1.4e7 ± 0.6e7	3.9e4 ± 1.7e4	7.39e3 ± 4e3
17	100 ± 0	1.4e8 ± 0.6e8	7.8e3 ± 3.2e3	1.6e7 ± 0.7e7	4e4 ± 1.6e4	8.4e3 ± 3.9e3
18	100 ± 0	1.6e8 ± 0.6e8	7.9e3 ± 3e3	1.8e7 ± 0.7e7	4.2e4 ± 1.6e4	9.28e3 ± 3.8e3
19	100 ± 0	1.8e8 ± 0.6e8	7.7e3 ± 2.5e3	1.9e7 ± 0.6e7	4.1e4 ± 1.3e4	9.96e3 ± 3.7e3
20	100 ± 0	1.9e8 ± 0.5e8	7.6e3 ± 2e3	2.1e7 ± 0.6e7	4.3e4 ± 1.2e4	1.03e4 ± 0.3e4
21	100 ± 0	2e8 ± 0.5e8	7.3e3 ± 1.8e3	2.1e7 ± 0.5e7	4.2e4 ± 1.1e4	1.08e4 ± 0.3e4
22	100 ± 0	2.1e8 ± 0.5e8	6.9e3 ± 1.5e3	2.2e7 ± 0.5e7	4.2e4 ± 1e4	1.1e4 ± 0.3e4
23	100 ± 0	2.2e8 ± 0.4e8	6.6e3 ± 1.2e3	2.3e7 ± 0.5e7	4e4 ± 0.8e4	1.11e4 ± 0.2e4
24	100 ± 0	2.2e8 ± 0.4e8	6.1e3 ± 1.1e3	2.2e7 ± 0.5e7	3.7e4 ± 0.8e4	1.1e4 ± 0.2e4
25	100 ± 0	2.3e8 ± 0.4e8	5.8e3 ± 1e3	2.3e7 ± 0.5e7	3.7e4 ± 0.8e4	1.07e4 ± 0.2e4
26	100 ± 0	2.3e8 ± 0.4e8	5.5e3 ± 0.9e3	2.4e7 ± 0.5e7	3.7e4 ± 0.8e4	1.06e4 ± 0.2e4
27	100 ± 0	2.4e8 ± 0.4e8	5.1e3 ± 0.8e3	2.4e7 ± 0.4e7	3.5e4 ± 0.6e4	1.05e4 ± 0.2e4
28	100 ± 0	2.4e8 ± 0.3e8	4.8e3 ± 0.7e3	2.4e7 ± 0.4e7	3.4e4 ± 0.6e4	1.03e4 ± 0.2e4
29	100 ± 0	2.4e8 ± 0.3e8	4.5e3 ± 0.6e3	2.4e7 ± 0.4e7	3.2e4 ± 0.6e4	1.01e4 ± 0.1e4
30	100 ± 0	2.4e8 ± 0.3e8	4.1e3 ± 0.6e3	2.3e7 ± 0.4e7	3.1e4 ± 0.6e4	9.67e3 ± 1.3e3
31	100 ± 0	2.4e8 ± 0.3e8	3.9e3 ± 0.5e3	2.3e7 ± 0.5e7	2.9e4 ± 0.6e4	9.29e3 ± 1.2e3
32	100 ± 0	2.4e8 ± 0.3e8	3.6e3 ± 0.5e3	2.4e7 ± 0.4e7	2.9e4 ± 0.5e4	8.92e3 ± 1.2e3
33	100 ± 0	2.4e8 ± 0.3e8	3.4e3 ± 0.4e3	2.4e7 ± 0.4e7	2.7e4 ± 0.5e4	8.67e3 ± 1.1e3
34	100 ± 0	2.4e8 ± 0.3e8	3.2e3 ± 0.4e3	2.4e7 ± 0.4e7	2.7e4 ± 0.5e4	8.35e3 ± 1e3
35	100 ± 0	2.4e8 ± 0.3e8	3.1e3 ± 0.3e3	2.4e7 ± 0.4e7	2.6e4 ± 0.4e4	8.1e3 ± 1e3
36	100 ± 0	2.4e8 ± 0.3e8	2.9e3 ± 0.3e3	2.3e7 ± 0.4e7	2.5e4 ± 0.4e4	7.91e3 ± 0.9e3
37	100 ± 0	2.4e8 ± 0.3e8	2.7e3 ± 0.3e3	2.4e7 ± 0.4e7	2.5e4 ± 0.4e4	7.54e3 ± 0.8e3

The average of the prevalence, the CFU-values per square meter (cfu/sqm) and per gram manure are calculated over all iterations. The CFU values in the intestine as well as the excreted and ingested quantities are obtained by calculating the average over all animals in one iteration at first and then over all iterations.

Table S6b (log_{10} CFU). Calculated numeric values for scenario when day-old chicks are set positive positive (10^2 CFU bacteria per chick on day 1; reference). Setting: 18 seeder, 72 sentinels, Ross 308, feeding duration 36 days, stocking density 39 kg/m^2, litter 1000 g/m^2

day	prevalence in %	cfu/sqm	cfu/g manure	cfu in intestine	cfu/g feces	cfu ingested per animal
	(% mean ± sd)	(log10 mean ± sd)	(log10 mean ± sd)	(log10 mean ± sd)	(log10 mean ± sd)	(log10 mean ± sd)
1	0 ± 0	-Inf ± -Inf	-Inf ± -Inf	-Inf ± -Inf	-Inf ± -Inf	1.3 ± 0
2	19.23 ± 1.03	4.89 ± 4.69	1.77 ± 1.57	4.19 ± 3.99	2.67 ± 2.47	-Inf ± 0
3	77.7 ± 21.9	5.25 ± 5.02	2.03 ± 1.8	4.44 ± 4.22	2.63 ± 2.41	1.13 ± 0.94
4	94.03 ± 9.78	5.58 ± 5.35	2.24 ± 2.02	4.76 ± 4.55	2.76 ± 2.55	1.49 ± 1.27
5	98.07 ± 5.05	5.89 ± 5.68	2.45 ± 2.24	5.07 ± 4.87	3.01 ± 2.8	1.76 ± 1.54
6	99.38 ± 1.98	6.18 ± 5.99	2.68 ± 2.48	5.36 ± 5.19	3.38 ± 3.21	2.05 ± 1.83
7	99.87 ± 0.69	6.47 ± 6.34	2.87 ± 2.74	5.64 ± 5.54	3.46 ± 3.36	2.33 ± 2.14
8	99.98 ± 0.16	6.75 ± 6.66	3.07 ± 2.98	5.92 ± 5.85	3.69 ± 3.62	2.57 ± 2.44
9	100 ± 0	7 ± 6.91	3.23 ± 3.14	6.15 ± 6.07	3.87 ± 3.8	2.82 ± 2.73
10	100 ± 0	7.23 ± 7.12	3.39 ± 3.29	6.38 ± 6.28	4.03 ± 3.93	3.03 ± 2.94
11	100 ± 0	7.46 ± 7.35	3.54 ± 3.44	6.6 ± 6.51	4.2 ± 4.11	3.23 ± 3.12
12	100 ± 0	7.64 ± 7.49	3.65 ± 3.51	6.76 ± 6.61	4.31 ± 4.16	3.42 ± 3.32
13	100 ± 0	7.79 ± 7.61	3.75 ± 3.57	6.9 ± 6.72	4.43 ± 4.24	3.56 ± 3.42
14	100 ± 0	7.91 ± 7.68	3.81 ± 3.57	7.01 ± 6.77	4.49 ± 4.25	3.69 ± 3.51
15	100 ± 0	8.02 ± 7.74	3.85 ± 3.58	7.1 ± 6.82	4.54 ± 4.27	3.79 ± 3.56
16	100 ± 0	8.09 ± 7.76	3.88 ± 3.54	7.16 ± 6.8	4.59 ± 4.23	3.87 ± 3.6
17	100 ± 0	8.16 ± 7.77	3.89 ± 3.51	7.21 ± 6.82	4.6 ± 4.21	3.92 ± 3.59
18	100 ± 0	8.21 ± 7.79	3.9 ± 3.47	7.26 ± 6.84	4.62 ± 4.2	3.97 ± 3.58
19	100 ± 0	8.25 ± 7.75	3.89 ± 3.39	7.28 ± 6.76	4.62 ± 4.1	4 ± 3.57
20	100 ± 0	8.28 ± 7.71	3.88 ± 3.31	7.31 ± 6.76	4.63 ± 4.08	4.01 ± 3.52
21	100 ± 0	8.31 ± 7.69	3.86 ± 3.25	7.33 ± 6.74	4.62 ± 4.03	4.03 ± 3.46
22	100 ± 0	8.33 ± 7.67	3.84 ± 3.18	7.34 ± 6.72	4.62 ± 3.99	4.04 ± 3.43
23	100 ± 0	8.34 ± 7.61	3.82 ± 3.08	7.35 ± 6.65	4.61 ± 3.91	4.04 ± 3.39
24	100 ± 0	8.35 ± 7.6	3.79 ± 3.04	7.35 ± 6.69	4.57 ± 3.9	4.04 ± 3.31
25	100 ± 0	8.36 ± 7.58	3.76 ± 2.98	7.37 ± 6.69	4.57 ± 3.89	4.03 ± 3.28
26	100 ± 0	8.37 ± 7.58	3.74 ± 2.95	7.37 ± 6.69	4.57 ± 3.89	4.03 ± 3.24
27	100 ± 0	8.37 ± 7.54	3.71 ± 2.88	7.38 ± 6.63	4.55 ± 3.8	4.02 ± 3.23
28	100 ± 0	8.38 ± 7.51	3.68 ± 2.81	7.38 ± 6.64	4.53 ± 3.79	4.01 ± 3.18
29	100 ± 0	8.38 ± 7.5	3.65 ± 2.78	7.38 ± 6.64	4.5 ± 3.76	4 ± 3.13
30	100 ± 0	8.38 ± 7.5	3.62 ± 2.74	7.37 ± 6.64	4.49 ± 3.75	3.99 ± 3.11
31	100 ± 0	8.37 ± 7.5	3.59 ± 2.71	7.37 ± 6.67	4.47 ± 3.76	3.97 ± 3.1
32	100 ± 0	8.38 ± 7.47	3.56 ± 2.66	7.38 ± 6.6	4.46 ± 3.68	3.95 ± 3.08
33	100 ± 0	8.38 ± 7.46	3.53 ± 2.62	7.37 ± 6.62	4.44 ± 3.68	3.94 ± 3.03
34	100 ± 0	8.38 ± 7.47	3.51 ± 2.6	7.38 ± 6.64	4.43 ± 3.69	3.92 ± 3
35	100 ± 0	8.38 ± 7.44	3.48 ± 2.54	7.38 ± 6.59	4.42 ± 3.62	3.91 ± 3
36	100 ± 0	8.38 ± 7.41	3.45 ± 2.49	7.37 ± 6.58	4.4 ± 3.6	3.9 ± 2.95
37	100 ± 0	8.38 ± 7.42	3.43 ± 2.47	7.38 ± 6.62	4.4 ± 3.64	3.88 ± 2.91

The average of the prevalence, the CFU-values per square meter (cfu/sqm) and per gram manure are calculated over all iterations. The CFU values in the intestine as well as the excreted and ingested quantities are obtained by calculating the average over all animals in one iteration at first and then over all iterations. The decadic logarithm of zero or negative values is negative infinite (-Inf).

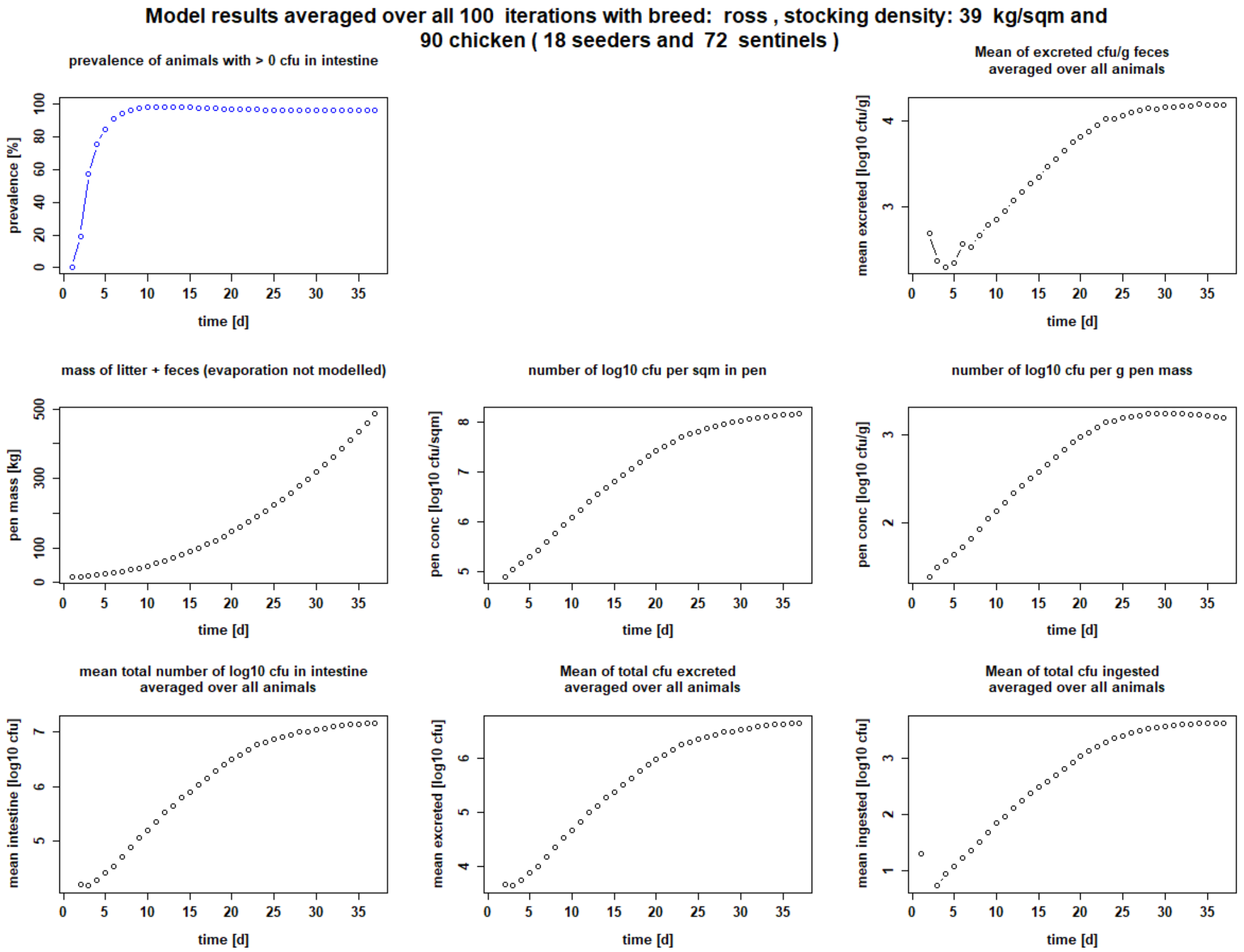

Figure 7. Calculated infection dynamic when day-old chicks are set positive (10^2 CFU bacteria per chick on day 1), measure 3000 g litter/m^2. Dotplots with results for 90 chicks, 18 seeder, 72 sentinels, Ross 308, feeding duration 36 days, stocking density 39 kg/m^2, 3000 g litter/m^2

Table S7a (log CFU). Calculated numeric values for scenario when day-old chicks are set positive (10^2 CFU bacteria per chick on day 1), measure 3000 g litter/m^2. Setting: 18 seeder, 72 sentinels, Ross 308, feeding duration 36 days; stocking density 39 kg/m^2, 3000 g litter/m^2

day	prevalence in %	cfu/sqm	cfu/g manure	cfu in intestine	cfu/g feces	cfu ingested per animal
	(mean ± sd)	(mean ± sd)	(mean ± sd)	(mean ± sd)	(mean ± sd)	(mean ± sd)
1	0 ± 0	0e0 ± 0	0e0 ± 0	0e0 ± 0	0e0 ± 0	2e1 ± 0e1
2	19.23 ± 0.83	8e4 ± 4.4e4	2.4e1 ± 1.3e1	1.6e4 ± 0.9e4	4.8e2 ± 2.6e2	0e0 ± 0
3	57.27 ± 24.07	1.1e5 ± 0.6e5	3.1e1 ± 1.6e1	1.5e4 ± 0.8e4	2.3e2 ± 1.3e2	5.26 ± 3.2
4	75.81 ± 23.09	1.5e5 ± 0.8e5	3.7e1 ± 1.8e1	1.9e4 ± 1e4	1.9e2 ± 1e2	8.68 ± 4.6
5	84.97 ± 18.14	2e5 ± 1.1e5	4.3e1 ± 2.4e1	2.6e4 ± 1.6e4	2.2e2 ± 1.4e2	1.19e1 ± 0.6e1
6	91.1 ± 14.36	2.7e5 ± 1.8e5	5.2e1 ± 3.4e1	3.4e4 ± 2.5e4	3.6e2 ± 2.6e2	1.64e1 ± 0.9e1
7	94.42 ± 10.57	3.9e5 ± 2.7e5	6.6e1 ± 4.5e1	5.1e4 ± 3.7e4	3.4e2 ± 2.4e2	2.3e1 ± 1.5e1
8	96.41 ± 7.74	5.8e5 ± 4.4e5	8.5e1 ± 6.5e1	7.7e4 ± 6.3e4	4.6e2 ± 3.7e2	3.25e1 ± 2.3e1
9	97.54 ± 7.6	8.7e5 ± 7.7e5	1.1e2 ± 1e2	1.2e5 ± 1.1e5	6.1e2 ± 5.9e2	4.73e1 ± 3.7e1
10	98.16 ± 7.27	1.2e6 ± 1.1e6	1.4e2 ± 1.2e2	1.6e5 ± 1.4e5	7e2 ± 6.4e2	7.02e1 ± 6.2e1
11	98.53 ± 6.24	1.7e6 ± 1.6e6	1.7e2 ± 1.5e2	2.3e5 ± 2.1e5	8.9e2 ± 8.3e2	9.33e1 ± 8.4e1
12	98.24 ± 9.52	2.5e6 ± 2.2e6	2.2e2 ± 1.9e2	3.3e5 ± 2.9e5	1.2e3 ± 1.1e3	1.29e2 ± 1.2e2
13	98.26 ± 10.08	3.5e6 ± 3.1e6	2.7e2 ± 2.4e2	4.4e5 ± 4.1e5	1.5e3 ± 1.4e3	1.77e2 ± 1.6e2
14	98.34 ± 9.72	4.8e6 ± 4.3e6	3.3e2 ± 2.9e2	6.1e5 ± 5.6e5	1.9e3 ± 1.7e3	2.35e2 ± 2.1e2
15	98.27 ± 10.06	6.3e6 ± 5.9e6	3.8e2 ± 3.6e2	7.7e5 ± 7.6e5	2.2e3 ± 2.1e3	3.16e2 ± 2.8e2
16	97.68 ± 13	8.5e6 ± 7.7e6	4.6e2 ± 4.2e2	1.1e6 ± 1e6	2.9e3 ± 2.6e3	3.94e2 ± 3.7e2
17	97.61 ± 13.41	1.1e7 ± 1.1e7	5.6e2 ± 5.2e2	1.4e6 ± 1.4e6	3.5e3 ± 3.4e3	5.11e2 ± 4.6e2
18	97.53 ± 13.46	1.5e7 ± 1.5e7	6.8e2 ± 6.6e2	1.9e6 ± 1.9e6	4.5e3 ± 4.4e3	6.64e2 ± 6.2e2
19	97.34 ± 14.93	2.1e7 ± 2.1e7	8.3e2 ± 8.5e2	2.6e6 ± 2.8e6	5.6e3 ± 6.2e3	8.61e2 ± 8.3e2
20	97.2 ± 15.28	2.6e7 ± 2.6e7	9.6e2 ± 9.5e2	3.1e6 ± 3.1e6	6.6e3 ± 6.5e3	1.11e3 ± 1.1e3
21	97.1 ± 15.43	3.2e7 ± 3e7	1.1e3 ± 1e3	3.8e6 ± 3.5e6	7.5e3 ± 6.9e3	1.36e3 ± 1.3e3
22	97.06 ± 15.58	4e7 ± 3.6e7	1.2e3 ± 1.1e3	4.7e6 ± 4.2e6	8.9e3 ± 7.9e3	1.62e3 ± 1.5e3
23	96.77 ± 16.27	4.9e7 ± 4.4e7	1.4e3 ± 1.2e3	5.9e6 ± 5.4e6	1.1e4 ± 1e4	1.95e3 ± 1.7e3
24	96.28 ± 18.33	5.6e7 ± 4.9e7	1.5e3 ± 1.3e3	6.3e6 ± 5.7e6	1.1e4 ± 0.9e4	2.35e3 ± 2.1e3
25	96.39 ± 17.78	6.5e7 ± 5.3e7	1.6e3 ± 1.3e3	7.3e6 ± 5.9e6	1.2e4 ± 1e4	2.58e3 ± 2.2e3
26	96.38 ± 17.84	7.2e7 ± 5.5e7	1.6e3 ± 1.2e3	8e6 ± 5.9e6	1.3e4 ± 0.9e4	2.87e3 ± 2.4e3
27	96.38 ± 17.84	8.1e7 ± 5.9e7	1.7e3 ± 1.2e3	9e6 ± 6.6e6	1.3e4 ± 1e4	3.11e3 ± 2.4e3
28	96.41 ± 17.67	9e7 ± 6.3e7	1.8e3 ± 1.2e3	9.9e6 ± 6.9e6	1.4e4 ± 1e4	3.39e3 ± 2.5e3
29	96.39 ± 17.79	9.7e7 ± 6.4e7	1.7e3 ± 1.2e3	1e7 ± 0.7e7	1.4e4 ± 0.9e4	3.65e3 ± 2.5e3
30	96.3 ± 18.22	1e8 ± 0.7e8	1.8e3 ± 1.1e3	1.1e7 ± 0.7e7	1.5e4 ± 0.9e4	3.77e3 ± 2.5e3
31	96.34 ± 18	1.1e8 ± 0.7e8	1.8e3 ± 1.1e3	1.2e7 ± 0.7e7	1.5e4 ± 0.9e4	3.96e3 ± 2.5e3
32	96.32 ± 18.11	1.2e8 ± 0.7e8	1.8e3 ± 1e3	1.3e7 ± 0.7e7	1.5e4 ± 0.9e4	4.08e3 ± 2.5e3
33	96.24 ± 18.49	1.2e8 ± 0.7e8	1.7e3 ± 0.9e3	1.3e7 ± 0.7e7	1.5e4 ± 0.8e4	4.18e3 ± 2.4e3
34	96.24 ± 18.49	1.3e8 ± 0.7e8	1.7e3 ± 0.9e3	1.4e7 ± 0.7e7	1.6e4 ± 0.8e4	4.25e3 ± 2.3e3
35	96.23 ± 18.55	1.4e8 ± 0.7e8	1.7e3 ± 0.8e3	1.4e7 ± 0.7e7	1.5e4 ± 0.8e4	4.34e3 ± 2.3e3
36	96.24 ± 18.5	1.4e8 ± 0.7e8	1.6e3 ± 0.8e3	1.4e7 ± 0.7e7	1.5e4 ± 0.7e4	4.37e3 ± 2.2e3
37	96.26 ± 18.44	1.4e8 ± 0.7e8	1.6e3 ± 0.7e3	1.5e7 ± 0.7e7	1.5e4 ± 0.7e4	4.35e3 ± 2.1e3

The average of the prevalence, the CFU-values per square meter (cfu/sqm) and per gram manure are calculated over all iterations. The CFU values in the intestine as well as the excreted and ingested quantities are obtained by calculating the average over all animals in one iteration at first and then over all iterations.

Corresponding Table S7b (log_{10} CFU). Calculated numeric values for scenario when day-old chicks are set positive (10^2 CFU bacteria per chick on day 1), measure 3000 g litter/m^2. Setting: 18 seeder, 72 sentinels, Ross 308, feeding duration 36 days, stocking density 39 kg/m^2, 3000 g litter/m^2

day	prevalence in %	cfu/sqm	cfu/g manure	cfu in intestine	cfu/g feces	cfu ingested per animal
	(% mean ± sd)	(log10 mean ± sd)	(log10 mean ± sd)	(log10 mean ± sd)	(log10 mean ± sd)	(log10 mean ± sd)
1	0 ± 0	-Inf ± -Inf	-Inf ± -Inf	-Inf ± -Inf	-Inf ± -Inf	1.3 ± 0
2	19.23 ± 0.83	4.91 ± 4.65	1.38 ± 1.12	4.21 ± 3.95	2.68 ± 2.42	-Inf ± 0
3	57.27 ± 24.07	5.06 ± 4.76	1.5 ± 1.2	4.17 ± 3.92	2.36 ± 2.11	0.72 ± 0.5
4	75.81 ± 23.09	5.19 ± 4.88	1.57 ± 1.26	4.28 ± 4.01	2.28 ± 2.01	0.94 ± 0.67
5	84.97 ± 18.14	5.31 ± 5.06	1.63 ± 1.38	4.41 ± 4.21	2.34 ± 2.14	1.08 ± 0.79
6	91.1 ± 14.36	5.44 ± 5.25	1.72 ± 1.54	4.53 ± 4.4	2.55 ± 2.42	1.21 ± 0.98
7	94.42 ± 10.57	5.59 ± 5.43	1.82 ± 1.66	4.71 ± 4.57	2.53 ± 2.39	1.36 ± 1.19
8	96.41 ± 7.74	5.77 ± 5.65	1.93 ± 1.81	4.89 ± 4.8	2.66 ± 2.57	1.51 ± 1.36
9	97.54 ± 7.6	5.94 ± 5.89	2.05 ± 2	5.06 ± 5.05	2.79 ± 2.77	1.68 ± 1.56
10	98.16 ± 7.27	6.09 ± 6.04	2.14 ± 2.09	5.19 ± 5.15	2.84 ± 2.81	1.85 ± 1.79
11	98.53 ± 6.24	6.24 ± 6.2	2.23 ± 2.19	5.35 ± 5.32	2.95 ± 2.92	1.97 ± 1.92
12	98.24 ± 9.52	6.4 ± 6.35	2.34 ± 2.28	5.52 ± 5.47	3.07 ± 3.02	2.11 ± 2.07
13	98.26 ± 10.08	6.54 ± 6.5	2.43 ± 2.38	5.65 ± 5.62	3.17 ± 3.14	2.25 ± 2.19
14	98.34 ± 9.72	6.68 ± 6.64	2.51 ± 2.47	5.79 ± 5.75	3.27 ± 3.23	2.37 ± 2.32
15	98.27 ± 10.06	6.8 ± 6.77	2.58 ± 2.55	5.89 ± 5.88	3.34 ± 3.33	2.5 ± 2.45
16	97.68 ± 13	6.93 ± 6.88	2.66 ± 2.62	6.03 ± 5.98	3.46 ± 3.42	2.6 ± 2.57
17	97.61 ± 13.41	7.06 ± 7.03	2.75 ± 2.72	6.15 ± 6.14	3.55 ± 3.53	2.71 ± 2.66
18	97.53 ± 13.46	7.19 ± 7.17	2.83 ± 2.82	6.29 ± 6.28	3.65 ± 3.64	2.82 ± 2.79
19	97.34 ± 14.93	7.31 ± 7.33	2.92 ± 2.93	6.41 ± 6.45	3.75 ± 3.79	2.93 ± 2.92
20	97.2 ± 15.28	7.42 ± 7.41	2.98 ± 2.98	6.5 ± 6.49	3.82 ± 3.81	3.05 ± 3.06
21	97.1 ± 15.43	7.51 ± 7.48	3.03 ± 3.01	6.58 ± 6.55	3.87 ± 3.84	3.14 ± 3.13
22	97.06 ± 15.58	7.6 ± 7.55	3.09 ± 3.04	6.67 ± 6.62	3.95 ± 3.9	3.21 ± 3.18
23	96.77 ± 16.27	7.69 ± 7.64	3.15 ± 3.1	6.77 ± 6.73	4.02 ± 3.98	3.29 ± 3.24
24	96.28 ± 18.33	7.75 ± 7.69	3.17 ± 3.11	6.8 ± 6.75	4.02 ± 3.97	3.37 ± 3.32
25	96.39 ± 17.78	7.81 ± 7.73	3.19 ± 3.11	6.86 ± 6.77	4.07 ± 3.98	3.41 ± 3.35
26	96.38 ± 17.84	7.86 ± 7.74	3.21 ± 3.09	6.9 ± 6.77	4.1 ± 3.97	3.46 ± 3.37
27	96.38 ± 17.84	7.91 ± 7.77	3.23 ± 3.09	6.95 ± 6.82	4.12 ± 3.99	3.49 ± 3.38
28	96.41 ± 17.67	7.96 ± 7.8	3.24 ± 3.09	7 ± 6.84	4.14 ± 3.99	3.53 ± 3.39
29	96.39 ± 17.79	7.99 ± 7.8	3.24 ± 3.06	7.01 ± 6.83	4.14 ± 3.95	3.56 ± 3.41
30	96.3 ± 18.22	8.02 ± 7.82	3.25 ± 3.04	7.05 ± 6.85	4.17 ± 3.96	3.58 ± 3.4
31	96.34 ± 18	8.05 ± 7.83	3.25 ± 3.03	7.07 ± 6.86	4.17 ± 3.95	3.6 ± 3.39
32	96.32 ± 18.11	8.07 ± 7.84	3.24 ± 3.01	7.1 ± 6.87	4.17 ± 3.95	3.61 ± 3.39
33	96.24 ± 18.49	8.1 ± 7.83	3.24 ± 2.97	7.11 ± 6.84	4.18 ± 3.9	3.62 ± 3.39
34	96.24 ± 18.49	8.12 ± 7.84	3.24 ± 2.95	7.14 ± 6.86	4.19 ± 3.92	3.63 ± 3.36
35	96.23 ± 18.55	8.14 ± 7.83	3.23 ± 2.92	7.15 ± 6.85	4.19 ± 3.89	3.64 ± 3.36
36	96.24 ± 18.5	8.15 ± 7.82	3.22 ± 2.89	7.16 ± 6.84	4.19 ± 3.87	3.64 ± 3.34
37	96.26 ± 18.44	8.16 ± 7.82	3.2 ± 2.87	7.17 ± 6.85	4.19 ± 3.86	3.64 ± 3.31

The average of the prevalence, the CFU-values per square meter (cfu/sqm) and per gram manure are calculated over all iterations. The CFU values in the intestine as well as the excreted and ingested quantities are obtained by calculating the average over all animals in one iteration at first and then over all iterations. The decadic logarithm of zero or negative values is negative infinite (-Inf).

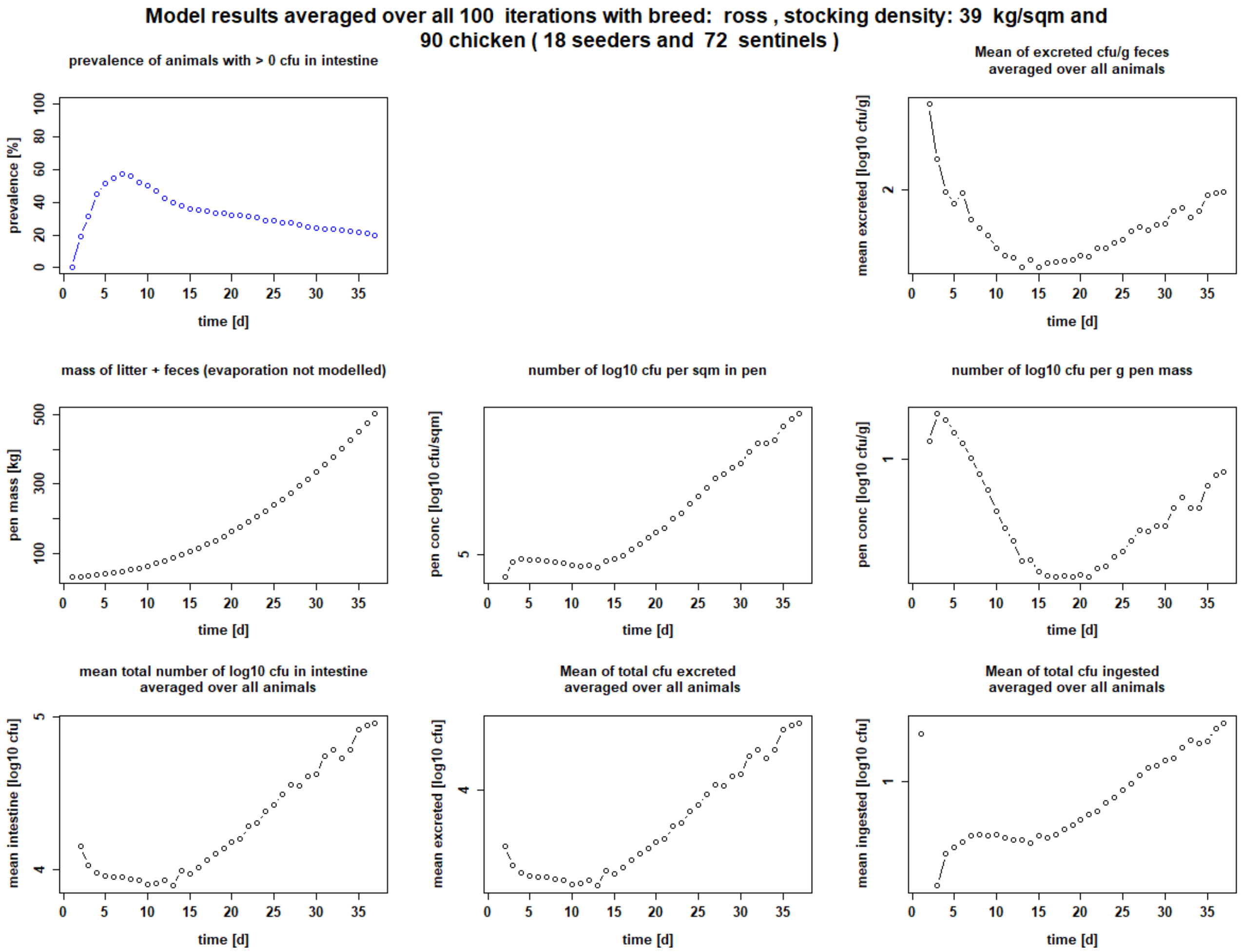

Figure S8. Calculated infection dynamic when day-old chicks are set positive (10^2 CFU bacteria per chick on day 1), measure 6000 g litter/m^2. Dotplots with results for 90 chicks,18 seeder, 72 sentinels, Ross 308, feeding duration 36 days, stocking density 39 kg/m^2; 6000 g litter/m^2

Table S8a (CFU). Calculated numeric values for scenario when day-old chicks are set positive (10^2 CFU bacteria per chick on day 1), measure 6000 g litter/m². Setting: 18 seeder, 72 sentinels, Ross 308, feeding duration 36 days, stocking density 39 kg/m²; 6000 g litter/m²

day	prevalence in %	cfu/sqm	cfu/g manure	cfu in intestine	cfu/g feces	cfu ingested per animal
	(mean ± sd)	(mean ± sd)	(mean ± sd)	(mean ± sd)	(mean ± sd)	(mean ± sd)
1	0 ± 0	0e0 ± 0	0e0 ± 0	0e0 ± 0	0e0 ± 0	2e1 ± 0e1
2	19.19 ± 0.97	7.1e4 ± 3.9e4	1.1e1 ± 0.6e1	1.4e4 ± 0.8e4	4.3e2 ± 2.3e2	0e0 ± 0
3	31.5 ± 18.5	9e4 ± 3.9e4	1.3e1 ± 0.6e1	1.1e4 ± 0.6e4	1.7e2 ± 1e2	2.17 ± 1.5
4	44.99 ± 22.83	9.3e4 ± 3.9e4	1.3e1 ± 0.5e1	9.6e3 ± 5e3	9.5e1 ± 5e1	3.49 ± 1.7
5	51.51 ± 23.57	9.2e4 ± 3.9e4	1.2e1 ± 0.5e1	9.2e3 ± 5.1e3	7.8e1 ± 4.3e1	3.81 ± 1.8
6	54.83 ± 24.12	9.1e4 ± 4.2e4	1.1e1 ± 0.5e1	9e3 ± 5e3	9.5e1 ± 5.3e1	4.12 ± 2
7	57.23 ± 25.11	9.1e4 ± 4.6e4	1e1 ± 0.5e1	8.9e3 ± 5.6e3	5.9e1 ± 3.7e1	4.53 ± 2.3
8	56.13 ± 27.21	8.9e4 ± 5.5e4	9.1 ± 5.5	8.7e3 ± 6.8e3	5.1e1 ± 4e1	4.58 ± 2.6
9	52.33 ± 30.29	8.8e4 ± 6.2e4	8.1 ± 5.8	8.6e3 ± 7.3e3	4.6e1 ± 3.9e1	4.54 ± 3.1
10	50.58 ± 31.81	8.4e4 ± 7.1e4	7.1 ± 6	8.1e3 ± 8.3e3	3.6e1 ± 3.8e1	4.63 ± 3.7
11	46.78 ± 33.48	8.3e4 ± 7.9e4	6.3 ± 6	8.2e3 ± 8.9e3	3.2e1 ± 3.5e1	4.4 ± 4.1
12	42.71 ± 34.97	8.5e4 ± 9.9e4	5.8 ± 6.8	8.6e3 ± 12.2e3	3.1e1 ± 4.4e1	4.28 ± 4.6
13	39.83 ± 36.13	8.2e4 ± 10.3e4	5.1 ± 6.4	7.9e3 ± 11e3	2.6e1 ± 3.7e1	4.24 ± 5.5
14	38.19 ± 35.86	9.1e4 ± 13e4	5.1 ± 7.4	9.9e3 ± 15.9e3	3e1 ± 4.8e1	4.03 ± 5.6
15	36.1 ± 36.16	9.3e4 ± 15.1e4	4.8 ± 7.7	9.5e3 ± 17.2e3	2.7e1 ± 4.8e1	4.5 ± 7.1
16	35.23 ± 36.27	9.9e4 ± 17.7e4	4.6 ± 8.3	1e4 ± 2.1e4	2.8e1 ± 5.6e1	4.39 ± 8
17	34.62 ± 36.39	1.1e5 ± 2.1e5	4.6 ± 8.9	1.2e4 ± 2.4e4	2.9e1 ± 6e1	4.58 ± 9.2
18	33.57 ± 36.35	1.2e5 ± 2.5e5	4.6 ± 9.6	1.3e4 ± 2.9e4	2.9e1 ± 6.6e1	4.94 ± 10.6
19	33.12 ± 36.85	1.3e5 ± 2.8e5	4.6 ± 10.2	1.4e4 ± 3.2e4	3e1 ± 7e1	5.28 ± 12.2
20	32.12 ± 36.79	1.4e5 ± 3.2e5	4.7 ± 10.5	1.5e4 ± 3.6e4	3.2e1 ± 7.5e1	5.71 ± 13.7
21	32.08 ± 37.09	1.5e5 ± 3.5e5	4.6 ± 10.6	1.6e4 ± 3.8e4	3.1e1 ± 7.4e1	6.15 ± 15
22	31.18 ± 36.98	1.7e5 ± 4.5e5	4.9 ± 12.6	1.9e4 ± 5.5e4	3.7e1 ± 10.5e1	6.51 ± 16
23	30.53 ± 36.5	1.9e5 ± 5e5	4.9 ± 13.1	2e4 ± 5.7e4	3.6e1 ± 10.2e1	7.29 ± 20
24	29.12 ± 36.47	2.2e5 ± 6e5	5.2 ± 14.6	2.4e4 ± 7.1e4	4e1 ± 11.8e1	7.85 ± 22
25	28.64 ± 36.43	2.4e5 ± 7e5	5.4 ± 15.8	2.7e4 ± 8e4	4.3e1 ± 12.8e1	8.79 ± 25.5
26	27.74 ± 36.08	2.8e5 ± 8.1e5	5.8 ± 17	3.1e4 ± 9.2e4	4.9e1 ± 14.5e1	9.64 ± 28.8
27	27.31 ± 36.26	3.2e5 ± 9.5e5	6.3 ± 18.6	3.6e4 ± 10.9e4	5.3e1 ± 16e1	1.09e1 ± 3.3e1
28	26.16 ± 35.56	3.4e5 ± 10.2e5	6.2 ± 18.6	3.6e4 ± 10.9e4	5e1 ± 15.2e1	1.22e1 ± 3.7e1
29	25.13 ± 35.39	3.7e5 ± 11.5e5	6.4 ± 19.6	4.1e4 ± 12.9e4	5.4e1 ± 17.1e1	1.27e1 ± 3.9e1
30	24.03 ± 35.71	4e5 ± 11.9e5	6.4 ± 19.1	4.2e4 ± 12.5e4	5.5e1 ± 16.2e1	1.36e1 ± 4.2e1
31	23.91 ± 35.68	4.8e5 ± 15.8e5	7.2 ± 23.8	5.6e4 ± 19.9e4	6.9e1 ± 24.8e1	1.41e1 ± 4.3e1
32	23.46 ± 35.55	5.5e5 ± 18.3e5	7.7 ± 25.9	6.1e4 ± 21e4	7.3e1 ± 25e1	1.65e1 ± 5.5e1
33	23.26 ± 35.12	5.4e5 ± 18.2e5	7.2 ± 24.4	5.4e4 ± 18.2e4	6.2e1 ± 21e1	1.82e1 ± 6.2e1
34	22.13 ± 35.12	5.7e5 ± 20.1e5	7.2 ± 25.3	6.1e4 ± 22.1e4	6.9e1 ± 25.1e1	1.75e1 ± 6e1
35	21.84 ± 35.08	7e5 ± 25.2e5	8.4 ± 30	8.3e4 ± 30.4e4	9e1 ± 33e1	1.81e1 ± 6.4e1
36	21.08 ± 34.92	7.9e5 ± 31.1e5	9 ± 35.1	8.8e4 ± 37.3e4	9.4e1 ± 39.7e1	2.15e1 ± 7.8e1
37	19.57 ± 33.98	8.6e5 ± 33.1e5	9.2 ± 35.4	9.1e4 ± 35.1e4	9.5e1 ± 36.4e1	2.36e1 ± 9.3e1

The average of the prevalence, the CFU-values per square meter (cfu/sqm) and per gram manure are calculated over all iterations. The CFU values in the intestine as well as the excreted and ingested quantities are obtained by calculating the average over all animals in one iteration at first and then over all iterations.

Table S8b (log₁₀ CFU). Calculated numeric values for scenario when day-old chicks are set positive (10^2 CFU bacteria per chick on day 1), measure 6000 g litter/m². Setting: 18 seeder, 72 sentinels, Ross 308, feeding duration 36 days, stocking density 39 kg/m²; 6000 g litter/m²

day	prevalence in %	cfu/sqm	cfu/g manure	cfu in intestine	cfu/g feces	cfu ingested per animal
	(% mean ± sd)	(log10 mean ± sd)	(log10 mean ± sd)	(log10 mean ± sd)	(log10 mean ± sd)	(log10 mean ± sd)
1	0 ± 0	-Inf ± -Inf	-Inf ± -Inf	-Inf ± -Inf	-Inf ± -Inf	1.3 ± 0
2	19.19 ± 0.97	4.85 ± 4.59	1.05 ± 0.79	4.15 ± 3.89	2.63 ± 2.37	-Inf ± 0
3	31.5 ± 18.5	4.95 ± 4.59	1.13 ± 0.77	4.03 ± 3.79	2.22 ± 1.98	0.34 ± 0.18
4	44.99 ± 22.83	4.97 ± 4.59	1.11 ± 0.74	3.98 ± 3.7	1.98 ± 1.7	0.54 ± 0.24
5	51.51 ± 23.57	4.97 ± 4.59	1.08 ± 0.7	3.96 ± 3.7	1.89 ± 1.64	0.58 ± 0.27
6	54.83 ± 24.12	4.96 ± 4.62	1.05 ± 0.71	3.96 ± 3.7	1.98 ± 1.72	0.61 ± 0.3
7	57.23 ± 25.11	4.96 ± 4.67	1 ± 0.71	3.95 ± 3.75	1.77 ± 1.57	0.66 ± 0.36
8	56.13 ± 27.21	4.95 ± 4.74	0.96 ± 0.74	3.94 ± 3.83	1.71 ± 1.6	0.66 ± 0.42
9	52.33 ± 30.29	4.94 ± 4.8	0.91 ± 0.76	3.93 ± 3.87	1.66 ± 1.59	0.66 ± 0.49
10	50.58 ± 31.81	4.93 ± 4.85	0.85 ± 0.78	3.91 ± 3.92	1.56 ± 1.57	0.67 ± 0.56
11	46.78 ± 33.48	4.92 ± 4.9	0.8 ± 0.78	3.91 ± 3.95	1.51 ± 1.55	0.64 ± 0.62
12	42.71 ± 34.97	4.93 ± 5	0.77 ± 0.83	3.94 ± 4.09	1.49 ± 1.64	0.63 ± 0.66
13	39.83 ± 36.13	4.91 ± 5.01	0.71 ± 0.81	3.9 ± 4.04	1.42 ± 1.56	0.63 ± 0.74
14	38.19 ± 35.86	4.96 ± 5.12	0.71 ± 0.87	4 ± 4.2	1.48 ± 1.68	0.61 ± 0.75
15	36.1 ± 36.16	4.97 ± 5.18	0.68 ± 0.89	3.98 ± 4.24	1.42 ± 1.68	0.65 ± 0.85
16	35.23 ± 36.27	4.99 ± 5.25	0.67 ± 0.92	4.02 ± 4.31	1.45 ± 1.75	0.64 ± 0.91
17	34.62 ± 36.39	5.03 ± 5.32	0.66 ± 0.95	4.06 ± 4.39	1.46 ± 1.78	0.66 ± 0.96
18	33.57 ± 36.35	5.07 ± 5.39	0.66 ± 0.98	4.11 ± 4.46	1.47 ± 1.82	0.69 ± 1.03
19	33.12 ± 36.85	5.11 ± 5.45	0.66 ± 1.01	4.14 ± 4.51	1.48 ± 1.85	0.72 ± 1.09
20	32.12 ± 36.79	5.15 ± 5.5	0.67 ± 1.02	4.19 ± 4.55	1.51 ± 1.88	0.76 ± 1.14
21	32.08 ± 37.09	5.18 ± 5.54	0.66 ± 1.02	4.2 ± 4.58	1.5 ± 1.87	0.79 ± 1.18
22	31.18 ± 36.98	5.24 ± 5.65	0.69 ± 1.1	4.28 ± 4.74	1.56 ± 2.02	0.81 ± 1.2
23	30.53 ± 36.5	5.27 ± 5.7	0.69 ± 1.12	4.31 ± 4.75	1.56 ± 2.01	0.86 ± 1.3
24	29.12 ± 36.47	5.33 ± 5.78	0.72 ± 1.17	4.39 ± 4.85	1.61 ± 2.07	0.89 ± 1.34
25	28.64 ± 36.43	5.38 ± 5.85	0.74 ± 1.2	4.43 ± 4.9	1.63 ± 2.11	0.94 ± 1.41
26	27.74 ± 36.08	5.44 ± 5.91	0.77 ± 1.23	4.5 ± 4.97	1.69 ± 2.16	0.98 ± 1.46
27	27.31 ± 36.26	5.5 ± 5.98	0.8 ± 1.27	4.56 ± 5.04	1.72 ± 2.21	1.04 ± 1.51
28	26.16 ± 35.56	5.53 ± 6.01	0.79 ± 1.27	4.55 ± 5.04	1.7 ± 2.18	1.09 ± 1.57
29	25.13 ± 35.39	5.57 ± 6.06	0.81 ± 1.29	4.61 ± 5.11	1.74 ± 2.23	1.1 ± 1.59
30	24.03 ± 35.71	5.6 ± 6.07	0.81 ± 1.28	4.63 ± 5.1	1.74 ± 2.21	1.13 ± 1.63
31	23.91 ± 35.68	5.68 ± 6.2	0.86 ± 1.38	4.74 ± 5.3	1.84 ± 2.39	1.15 ± 1.63
32	23.46 ± 35.55	5.74 ± 6.26	0.89 ± 1.41	4.78 ± 5.32	1.86 ± 2.4	1.22 ± 1.74
33	23.26 ± 35.12	5.73 ± 6.26	0.86 ± 1.39	4.73 ± 5.26	1.79 ± 2.32	1.26 ± 1.79
34	22.13 ± 35.12	5.76 ± 6.3	0.86 ± 1.4	4.78 ± 5.35	1.84 ± 2.4	1.24 ± 1.78
35	21.84 ± 35.08	5.85 ± 6.4	0.92 ± 1.48	4.92 ± 5.48	1.95 ± 2.52	1.26 ± 1.8
36	21.08 ± 34.92	5.9 ± 6.49	0.95 ± 1.55	4.95 ± 5.57	1.97 ± 2.6	1.33 ± 1.89
37	19.57 ± 33.98	5.93 ± 6.52	0.96 ± 1.55	4.96 ± 5.54	1.98 ± 2.56	1.37 ± 1.97

The average of the prevalence, the CFU-values per square meter (cfu/sqm) and per gram manure are calculated over all iterations. The CFU values in the intestine as well as the excreted and ingested quantities are obtained by calculating the average over all animals in one iteration at first and then over all iterations. The decadic logarithm of zero or negative values is negative infinite (-Inf).

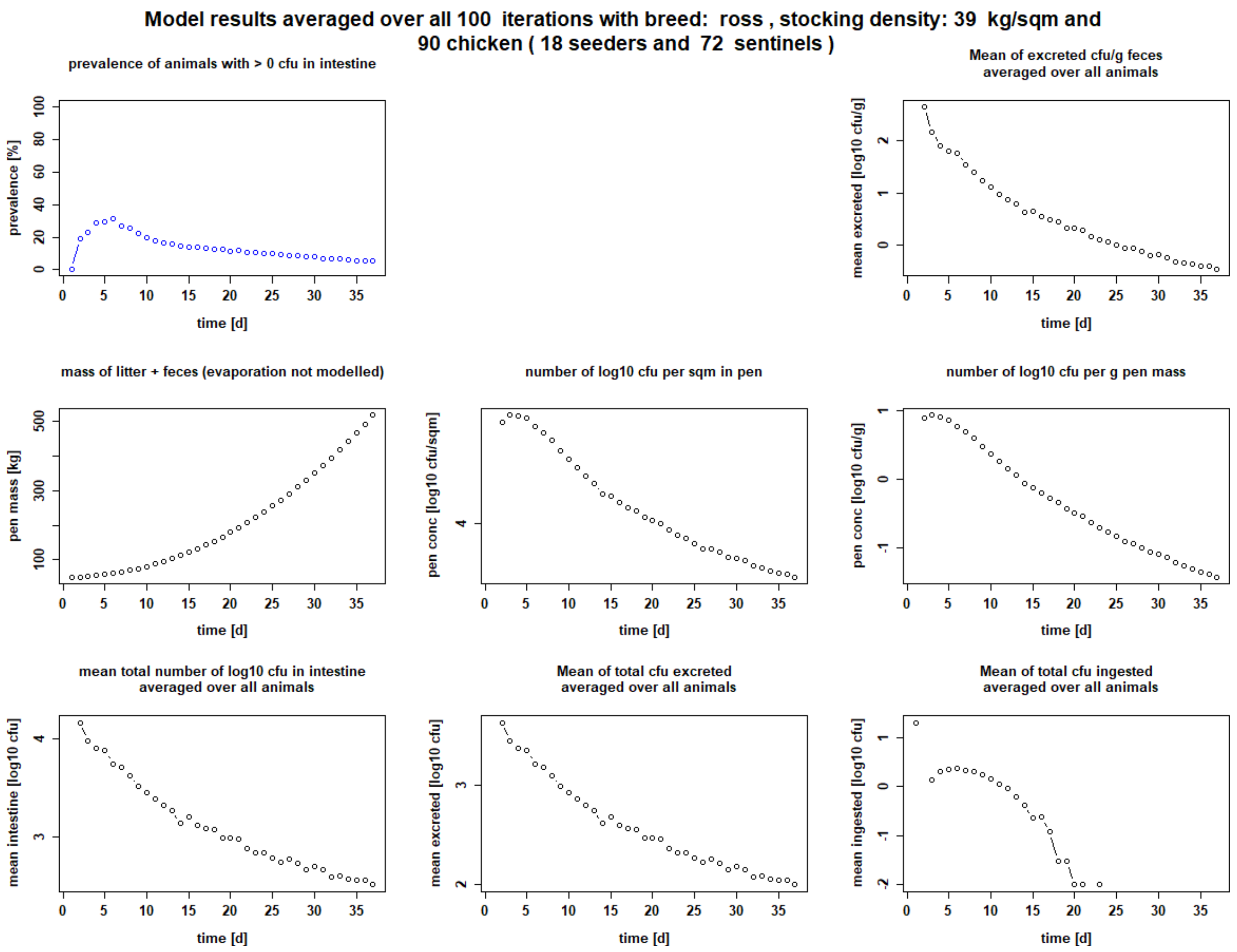

Figure S9. Calculated infection dynamic when day-old chicks are set positive (10^2 CFU bacteria per chick on day 1), measure 9000 g litter/m^2. Dotplots with results for 90 chicks, 18 seeder, 72 sentinels, Ross 308, feeding duration 36 days, stocking density 39 kg/m^2; 9000 g litter/m^2

Table S9a (CFU). Calculated numeric values for scenario when day-old chicks are set positive (10^2 CFU bacteria per chick on day 1), measure 9000 g litter/m^2. Setting: 18 seeder, 72 sentinels, Ross 308, feeding duration 36 days, stocking density 39 kg/m^2; 9000 g litter/m^2

day	prevalence in %	cfu/sqm	cfu/g manure	cfu in intestine	cfu/g feces	cfu ingested per animal
	(mean ± sd)	(mean ± sd)	(mean ± sd)	(mean ± sd)	(mean ± sd)	(mean ± sd)
1	0 ± 0	0e0 ± 0	0e0 ± 0	0e0 ± 0	0e0 ± 0	2e1 ± 0e1
2	19.2 ± 0.85	7.2e4 ± 4.5e4	7.8 ± 4.8	1.4e4 ± 0.9e4	4.3e2 ± 2.7e2	0e0 ± 0
3	22.96 ± 11.57	8.4e4 ± 3.7e4	8.7 ± 3.8	9.5e3 ± 5.8e3	1.5e2 ± 0.9e2	1.35 ± 1.2
4	28.84 ± 16.48	8.2e4 ± 3.6e4	8.1 ± 3.5	8e3 ± 4.8e3	7.9e1 ± 4.8e1	1.99 ± 1.2
5	29.72 ± 16.97	7.8e4 ± 3.3e4	7.3 ± 3	7.5e3 ± 4.4e3	6.4e1 ± 3.8e1	2.25 ± 1.2
6	31.47 ± 18.3	6.7e4 ± 2.9e4	6 ± 2.6	5.5e3 ± 3.5e3	5.8e1 ± 3.6e1	2.33 ± 1.2
7	27.13 ± 17.83	5.9e4 ± 2.6e4	4.9 ± 2.2	5.1e3 ± 3.1e3	3.4e1 ± 2.1e1	2.17 ± 1.2
8	25.84 ± 16.7	5.1e4 ± 2.3e4	3.9 ± 1.8	4.2e3 ± 2.7e3	2.5e1 ± 1.6e1	1.99 ± 1.1
9	22.23 ± 14.03	4.2e4 ± 1.9e4	3 ± 1.4	3.2e3 ± 2.2e3	1.7e1 ± 1.2e1	1.72 ± 1.1
10	19.8 ± 11.28	3.5e4 ± 1.6e4	2.4 ± 1.1	2.8e3 ± 2e3	1.3e1 ± 0.9e1	1.41 ± 0.9
11	17.46 ± 7.97	3e4 ± 1.4e4	1.8 ± 0.9	2.4e3 ± 1.9e3	9.6 ± 7.3	1.12 ± 0.8
12	16.43 ± 8.29	2.5e4 ± 1.2e4	1.4 ± 0.7	2.1e3 ± 1.6e3	7.5 ± 5.7	9.3e-1 ± 7.7e-1
13	15.54 ± 5.61	2.2e4 ± 1e4	1.2 ± 0.5	1.9e3 ± 1.3e3	6.2 ± 4.3	6.1e-1 ± 7.1e-1
14	14.26 ± 3.85	1.8e4 ± 0.7e4	8.6e-1 ± 3.5e-1	1.4e3 ± 1e3	4.2 ± 3.2	4.1e-1 ± 5.7e-1
15	13.84 ± 2.37	1.7e4 ± 0.7e4	7.6e-1 ± 3e-1	1.6e3 ± 1.1e3	4.5 ± 3	2.3e-1 ± 4.5e-1
16	13.58 ± 2.31	1.5e4 ± 0.5e4	6.2e-1 ± 2.2e-1	1.3e3 ± 0.8e3	3.6 ± 2.1	2.4e-1 ± 4.3e-1
17	13.24 ± 2.38	1.4e4 ± 0.5e4	5.2e-1 ± 1.8e-1	1.2e3 ± 0.9e3	3.1 ± 2.1	1.2e-1 ± 3.3e-1
18	12.51 ± 2.27	1.3e4 ± 0.5e4	4.5e-1 ± 1.6e-1	1.2e3 ± 0.8e3	2.8 ± 1.8	3e-2 ± 17.1e-2
19	12.41 ± 2.12	1.1e4 ± 0.4e4	3.7e-1 ± 1.3e-1	9.9e2 ± 7.3e2	2.2 ± 1.6	3e-2 ± 17.1e-2
20	11.51 ± 2.18	1.1e4 ± 0.4e4	3.2e-1 ± 1.3e-1	9.9e2 ± 7.7e2	2.1 ± 1.6	1e-2 ± 10e-2
21	11.62 ± 2.06	1e4 ± 0.4e4	2.8e-1 ± 1e-1	9.6e2 ± 5.9e2	1.9 ± 1.2	1e-2 ± 10e-2
22	10.74 ± 2.4	8.9e3 ± 3.6e3	2.3e-1 ± 0.9e-1	7.7e2 ± 5.7e2	1.5 ± 1.1	0e0 ± 0
23	10.3 ± 2.3	8e3 ± 3.2e3	1.9e-1 ± 0.8e-1	7e2 ± 5e2	1.2 ± 0.9	1e-2 ± 10e-2
24	10.18 ± 2.18	7.5e3 ± 3e3	1.7e-1 ± 0.7e-1	7e2 ± 5.1e2	1.2 ± 0.8	0e0 ± 0
25	9.63 ± 2.19	6.9e3 ± 3e3	1.4e-1 ± 0.6e-1	6.2e2 ± 4.8e2	9.9e-1 ± 7.6e-1	0e0 ± 0
26	9.36 ± 2.06	6.2e3 ± 2.3e3	1.2e-1 ± 0.5e-1	5.5e2 ± 4.6e2	8.7e-1 ± 7.2e-1	0e0 ± 0
27	8.87 ± 2.22	6.1e3 ± 2.5e3	1.1e-1 ± 0.5e-1	6e2 ± 4.5e2	8.8e-1 ± 6.7e-1	0e0 ± 0
28	8.72 ± 2.31	5.8e3 ± 2.3e3	10e-2 ± 4.1e-2	5.4e2 ± 3.9e2	7.6e-1 ± 5.5e-1	0e0 ± 0
29	8.04 ± 2.25	5.3e3 ± 2.3e3	8.6e-2 ± 3.8e-2	4.7e2 ± 4.1e2	6.3e-1 ± 5.4e-1	0e0 ± 0
30	7.83 ± 2.42	5.1e3 ± 2.7e3	7.9e-2 ± 4.1e-2	5e2 ± 4.8e2	6.5e-1 ± 6.2e-1	0e0 ± 0
31	6.99 ± 2.39	4.9e3 ± 2.4e3	7.1e-2 ± 3.4e-2	4.7e2 ± 4.2e2	5.9e-1 ± 5.2e-1	0e0 ± 0
32	7 ± 2.36	4.4e3 ± 2.1e3	6.1e-2 ± 2.8e-2	3.9e2 ± 3.7e2	4.7e-1 ± 4.4e-1	0e0 ± 0
33	6.8 ± 2.17	4.2e3 ± 2.1e3	5.4e-2 ± 2.7e-2	4e2 ± 3.6e2	4.6e-1 ± 4.2e-1	0e0 ± 0
34	6.33 ± 2.23	4e3 ± 2.1e3	4.9e-2 ± 2.6e-2	3.8e2 ± 3.7e2	4.3e-1 ± 4.2e-1	0e0 ± 0
35	5.62 ± 2.35	3.8e3 ± 2e3	4.4e-2 ± 2.3e-2	3.6e2 ± 3.6e2	4e-1 ± 3.9e-1	0e0 ± 0
36	5.56 ± 2.16	3.8e3 ± 2.2e3	4.1e-2 ± 2.4e-2	3.7e2 ± 3.8e2	3.9e-1 ± 4e-1	0e0 ± 0
37	5.29 ± 1.97	3.5e3 ± 1.8e3	3.7e-2 ± 1.9e-2	3.3e2 ± 2.9e2	3.4e-1 ± 3e-1	0e0 ± 0

The average of the prevalence, the CFU-values per square meter (cfu/sqm) and per gram manure are calculated over all iterations. The CFU values in the intestine as well as the excreted and ingested quantities are obtained by calculating the average over all animals in one iteration at first and then over all iterations.

Table S9b (log_{10} CFU). Calculated numeric values for scenario when day-old chicks are set positive (10^2 CFU bacteria per chick on day 1), measure 9000 g litter/m². Setting: 18 seeder, 72 sentinels, Ross 308, feeding duration 36 days, stocking density 39 kg/m²; 9000 g litter/m²

day	prevalence in %	cfu/sqm	cfu/g manure	cfu in intestine	cfu/g feces	cfu ingested per animal
	(% mean ± sd)	(log10 mean ± sd)	(log10 mean ± sd)	(log10 mean ± sd)	(log10 mean ± sd)	(log10 mean ± sd)
1	0 ± 0	-Inf ± -Inf	-Inf ± -Inf	-Inf ± -Inf	-Inf ± -Inf	1.3 ± 0
2	19.2 ± 0.85	4.86 ± 4.65	0.89 ± 0.68	4.16 ± 3.95	2.64 ± 2.43	-Inf ± 0
3	22.96 ± 11.57	4.92 ± 4.57	0.94 ± 0.59	3.98 ± 3.76	2.17 ± 1.95	0.13 ± 0.07
4	28.84 ± 16.48	4.91 ± 4.56	0.91 ± 0.55	3.9 ± 3.68	1.9 ± 1.68	0.3 ± 0.07
5	29.72 ± 16.97	4.89 ± 4.51	0.86 ± 0.48	3.87 ± 3.64	1.81 ± 1.58	0.35 ± 0.09
6	31.47 ± 18.3	4.83 ± 4.46	0.78 ± 0.41	3.74 ± 3.54	1.76 ± 1.56	0.37 ± 0.1
7	27.13 ± 17.83	4.77 ± 4.41	0.69 ± 0.34	3.71 ± 3.49	1.53 ± 1.31	0.34 ± 0.07
8	25.84 ± 16.7	4.7 ± 4.37	0.6 ± 0.26	3.63 ± 3.44	1.4 ± 1.21	0.3 ± 0.03
9	22.23 ± 14.03	4.62 ± 4.28	0.48 ± 0.14	3.51 ± 3.35	1.23 ± 1.07	0.24 ± 0.02
10	19.8 ± 11.28	4.54 ± 4.2	0.37 ± 0.03	3.45 ± 3.3	1.11 ± 0.95	0.15 ± -0.03
11	17.46 ± 7.97	4.47 ± 4.16	0.26 ± -0.05	3.38 ± 3.27	0.98 ± 0.86	0.05 ± -0.11
12	16.43 ± 8.29	4.4 ± 4.09	0.16 ± -0.15	3.32 ± 3.2	0.87 ± 0.76	-0.03 ± -0.11
13	15.54 ± 5.61	4.34 ± 3.98	0.06 ± -0.3	3.27 ± 3.11	0.8 ± 0.63	-0.21 ± -0.15
14	14.26 ± 3.85	4.25 ± 3.87	-0.06 ± -0.45	3.14 ± 3.02	0.62 ± 0.5	-0.39 ± -0.24
15	13.84 ± 2.37	4.23 ± 3.82	-0.12 ± -0.53	3.21 ± 3.03	0.65 ± 0.47	-0.64 ± -0.35
16	13.58 ± 2.31	4.18 ± 3.73	-0.21 ± -0.66	3.12 ± 2.89	0.56 ± 0.33	-0.62 ± -0.37
17	13.24 ± 2.38	4.14 ± 3.67	-0.28 ± -0.75	3.09 ± 2.94	0.49 ± 0.33	-0.92 ± -0.49
18	12.51 ± 2.27	4.11 ± 3.66	-0.34 ± -0.79	3.08 ± 2.9	0.44 ± 0.26	-1.52 ± -0.77
19	12.41 ± 2.12	4.06 ± 3.62	-0.43 ± -0.87	2.99 ± 2.86	0.33 ± 0.2	-1.52 ± -0.77
20	11.51 ± 2.18	4.03 ± 3.63	-0.49 ± -0.89	2.99 ± 2.89	0.32 ± 0.21	-2 ± -1
21	11.62 ± 2.06	4.01 ± 3.56	-0.55 ± -0.99	2.98 ± 2.77	0.28 ± 0.07	-2 ± -1
22	10.74 ± 2.4	3.95 ± 3.56	-0.63 ± -1.02	2.89 ± 2.75	0.17 ± 0.03	-Inf ± 0
23	10.3 ± 2.3	3.9 ± 3.51	-0.71 ± -1.11	2.84 ± 2.7	0.1 ± -0.05	-2 ± -1
24	10.18 ± 2.18	3.87 ± 3.47	-0.77 ± -1.18	2.84 ± 2.7	0.06 ± -0.08	-Inf ± 0
25	9.63 ± 2.19	3.84 ± 3.47	-0.84 ± -1.2	2.79 ± 2.68	0 ± -0.12	-Inf ± 0
26	9.36 ± 2.06	3.79 ± 3.36	-0.91 ± -1.35	2.74 ± 2.66	-0.06 ± -0.14	-Inf ± 0
27	8.87 ± 2.22	3.78 ± 3.4	-0.95 ± -1.33	2.77 ± 2.66	-0.06 ± -0.18	-Inf ± 0
28	8.72 ± 2.31	3.76 ± 3.37	-1 ± -1.39	2.73 ± 2.59	-0.12 ± -0.26	-Inf ± 0
29	8.04 ± 2.25	3.72 ± 3.37	-1.07 ± -1.42	2.67 ± 2.61	-0.2 ± -0.27	-Inf ± 0
30	7.83 ± 2.42	3.71 ± 3.43	-1.1 ± -1.39	2.7 ± 2.68	-0.18 ± -0.21	-Inf ± 0
31	6.99 ± 2.39	3.69 ± 3.37	-1.15 ± -1.47	2.67 ± 2.62	-0.23 ± -0.28	-Inf ± 0
32	7 ± 2.36	3.65 ± 3.32	-1.22 ± -1.55	2.6 ± 2.56	-0.33 ± -0.36	-Inf ± 0
33	6.8 ± 2.17	3.63 ± 3.33	-1.26 ± -1.57	2.6 ± 2.56	-0.34 ± -0.38	-Inf ± 0
34	6.33 ± 2.23	3.6 ± 3.33	-1.31 ± -1.59	2.58 ± 2.57	-0.37 ± -0.38	-Inf ± 0
35	5.62 ± 2.35	3.58 ± 3.3	-1.35 ± -1.64	2.56 ± 2.56	-0.4 ± -0.4	-Inf ± 0
36	5.56 ± 2.16	3.58 ± 3.34	-1.39 ± -1.62	2.57 ± 2.58	-0.41 ± -0.39	-Inf ± 0
37	5.29 ± 1.97	3.55 ± 3.25	-1.44 ± -1.73	2.52 ± 2.46	-0.47 ± -0.52	-Inf ± 0

The average of the prevalence, the CFU-values per square meter (cfu/sqm) and per gram manure are calculated over all iterations. The CFU values in the intestine as well as the excreted and ingested quantities are obtained by calculating the average over all animals in one iteration at first and then over all iterations. The decadic logarithm of zero or negative values is negative infinite (-Inf).

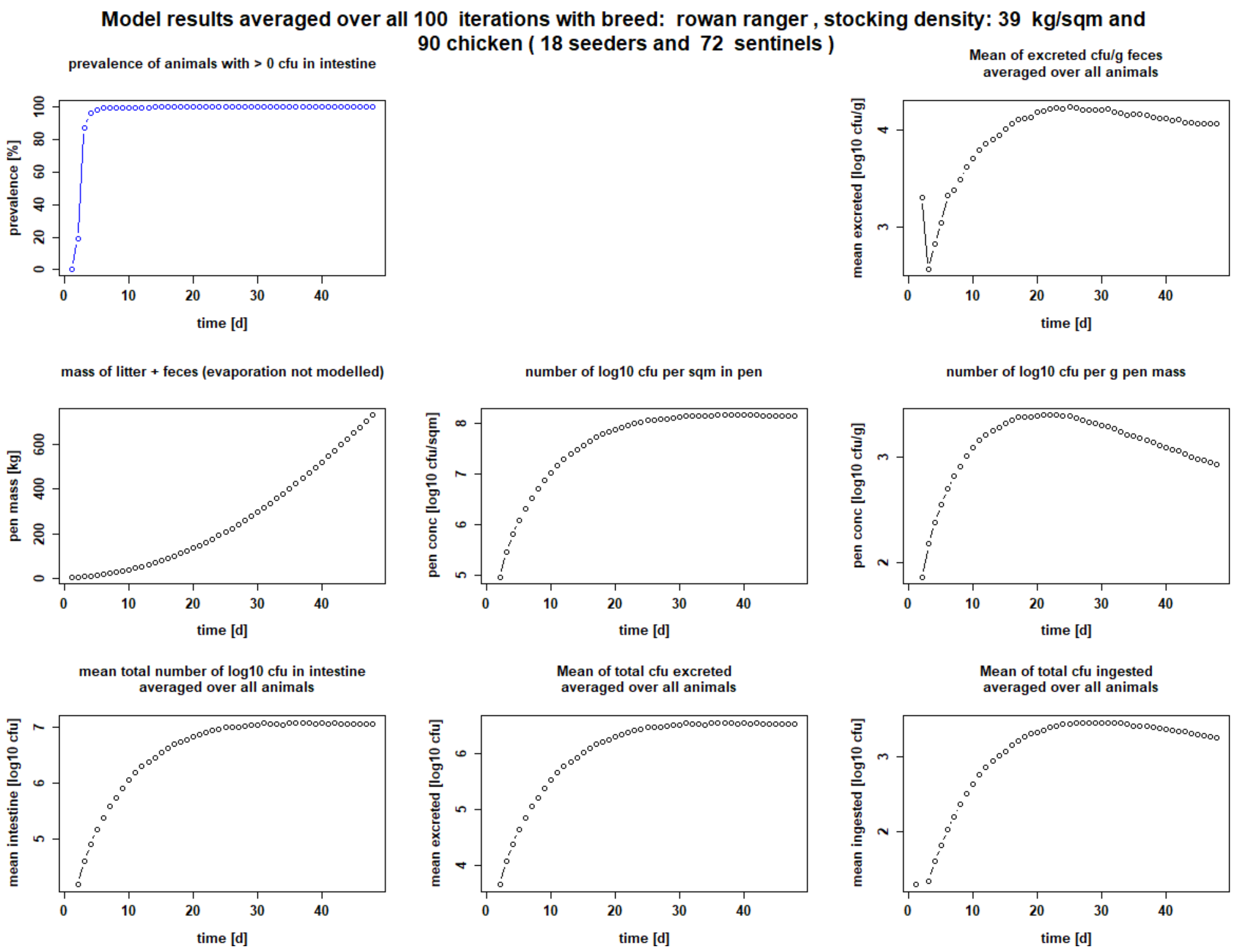

Figure S10. Calculated infection dynamic when day-old chicks are set positive (10^2 CFU bacteria per chick on day 1), measure slow growing breed. Dotplots with results for 90 chicks, 18 seeder, 72 sentinels, Rowan x Ranger, feeding duration 47 days, stocking density 39 kg/m^2; 1000g litter/m^2

Table S10a (CFU). Calculated numeric values for scenario when day-old chicks are set positive (10^2 CFU bacteria per chick on day 1), measure slow growing breed. Setting: 90 chicks, 18 seeder, 72 sentinels, Rowan x Ranger, feeding duration 47 days; stocking density 39 kg/m^2, 1000 g litter/m^2

day	prevalence in %	cfu/sqm	cfu/g manure	cfu in intestine	cfu/g feces	cfu ingested per animal
	(mean ± sd)	(mean ± sd)	(mean ± sd)	(mean ± sd)	(mean ± sd)	(mean ± sd)
1	0 ± 0	0e0 ± 0	0e0 ± 0	0e0 ± 0	0e0 ± 0	2e1 ± 0e1
2	19.12 ± 0.87	9e4 ± 6e4	7.2e1 ± 4.8e1	1.5e4 ± 1e4	2e3 ± 1.4e3	0e0 ± 0
3	87.49 ± 12.77	2.8e5 ± 1.8e5	1.5e2 ± 0.9e2	3.9e4 ± 2.4e4	3.7e2 ± 2.3e2	2.26e1 ± 1.6e1
4	96.36 ± 8.11	6.3e5 ± 4.3e5	2.4e2 ± 1.6e2	8e4 ± 5.7e4	6.7e2 ± 4.7e2	4.11e1 ± 2.6e1
5	98.67 ± 4.02	1.2e6 ± 0.9e6	3.5e2 ± 2.6e2	1.4e5 ± 1.1e5	1.1e3 ± 0.9e3	6.68e1 ± 4.6e1
6	99.59 ± 1.82	2e6 ± 1.6e6	5e2 ± 3.8e2	2.3e5 ± 1.9e5	2.1e3 ± 1.7e3	1.07e2 ± 0.8e2
7	99.71 ± 1.35	3.3e6 ± 2.6e6	6.6e2 ± 5.1e2	3.8e5 ± 3e5	2.4e3 ± 1.9e3	1.59e2 ± 1.2e2
8	99.91 ± 0.54	5e6 ± 4.1e6	8.2e2 ± 6.7e2	5.5e5 ± 4.8e5	3.1e3 ± 2.7e3	2.29e2 ± 1.8e2
9	99.9 ± 0.53	7.4e6 ± 5.8e6	1e3 ± 0.8e3	8.1e5 ± 6.3e5	4.1e3 ± 3.2e3	3.21e2 ± 2.6e2
10	99.92 ± 0.51	1.1e7 ± 0.9e7	1.2e3 ± 1e3	1.1e6 ± 1e6	5.1e3 ± 4.4e3	4.26e2 ± 3.3e2
11	99.98 ± 0.16	1.5e7 ± 1.3e7	1.5e3 ± 1.3e3	1.6e6 ± 1.4e6	6.1e3 ± 5.6e3	5.65e2 ± 4.6e2
12	99.99 ± 0.11	2e7 ± 1.7e7	1.6e3 ± 1.4e3	2e6 ± 1.8e6	7.1e3 ± 6.5e3	7.14e2 ± 6.2e2
13	99.98 ± 0.16	2.4e7 ± 2.1e7	1.8e3 ± 1.5e3	2.4e6 ± 2e6	8e3 ± 6.7e3	8.69e2 ± 7.7e2
14	100 ± 0	3e7 ± 2.5e7	1.9e3 ± 1.6e3	2.9e6 ± 2.5e6	8.9e3 ± 7.7e3	1.02e3 ± 0.9e3
15	100 ± 0	3.7e7 ± 3e7	2.1e3 ± 1.7e3	3.6e6 ± 3e6	1e4 ± 0.8e4	1.17e3 ± 1e3
16	100 ± 0	4.5e7 ± 3.6e7	2.2e3 ± 1.8e3	4.3e6 ± 3.4e6	1.2e4 ± 0.9e4	1.39e3 ± 1.1e3
17	100 ± 0	5.4e7 ± 4.3e7	2.4e3 ± 1.9e3	5.1e6 ± 4.3e6	1.3e4 ± 1.1e4	1.58e3 ± 1.3e3
18	100 ± 0	6.1e7 ± 4.7e7	2.4e3 ± 1.9e3	5.6e6 ± 4.3e6	1.3e4 ± 1e4	1.8e3 ± 1.5e3
19	100 ± 0	6.7e7 ± 5e7	2.4e3 ± 1.8e3	6e6 ± 4.5e6	1.3e4 ± 1e4	1.97e3 ± 1.5e3
20	100 ± 0	7.6e7 ± 5.5e7	2.5e3 ± 1.8e3	6.9e6 ± 5e6	1.5e4 ± 1.1e4	2.06e3 ± 1.5e3
21	100 ± 0	8.4e7 ± 5.6e7	2.5e3 ± 1.7e3	7.6e6 ± 5e6	1.6e4 ± 1e4	2.22e3 ± 1.6e3
22	100 ± 0	9.2e7 ± 6e7	2.5e3 ± 1.6e3	8.2e6 ± 5.4e6	1.6e4 ± 1.1e4	2.38e3 ± 1.6e3
23	100 ± 0	1e8 ± 0.6e8	2.5e3 ± 1.6e3	8.9e6 ± 5.5e6	1.7e4 ± 1e4	2.49e3 ± 1.6e3
24	100 ± 0	1.1e8 ± 0.6e8	2.5e3 ± 1.4e3	9.3e6 ± 5.5e6	1.6e4 ± 1e4	2.64e3 ± 1.6e3
25	100 ± 0	1.1e8 ± 0.7e8	2.4e3 ± 1.4e3	9.9e6 ± 6e6	1.7e4 ± 1e4	2.68e3 ± 1.6e3
26	100 ± 0	1.2e8 ± 0.7e8	2.3e3 ± 1.3e3	1e7 ± 0.6e7	1.7e4 ± 1e4	2.79e3 ± 1.6e3
27	100 ± 0	1.2e8 ± 0.7e8	2.2e3 ± 1.2e3	1e7 ± 0.6e7	1.6e4 ± 0.9e4	2.78e3 ± 1.6e3
28	100 ± 0	1.2e8 ± 0.7e8	2.1e3 ± 1.1e3	1e7 ± 0.6e7	1.6e4 ± 0.8e4	2.76e3 ± 1.5e3
29	100 ± 0	1.3e8 ± 0.7e8	2.1e3 ± 1e3	1.1e7 ± 0.6e7	1.6e4 ± 0.8e4	2.74e3 ± 1.4e3
30	100 ± 0	1.3e8 ± 0.6e8	2e3 ± 1e3	1.1e7 ± 0.6e7	1.6e4 ± 0.8e4	2.77e3 ± 1.4e3
31	100 ± 0	1.4e8 ± 0.7e8	1.9e3 ± 0.9e3	1.2e7 ± 0.6e7	1.6e4 ± 0.8e4	2.76e3 ± 1.3e3
32	100 ± 0	1.4e8 ± 0.7e8	1.8e3 ± 0.9e3	1.2e7 ± 0.6e7	1.5e4 ± 0.7e4	2.78e3 ± 1.3e3
33	100 ± 0	1.4e8 ± 0.6e8	1.8e3 ± 0.8e3	1.2e7 ± 0.5e7	1.5e4 ± 0.7e4	2.72e3 ± 1.3e3
34	100 ± 0	1.4e8 ± 0.6e8	1.6e3 ± 0.7e3	1.1e7 ± 0.5e7	1.4e4 ± 0.7e4	2.65e3 ± 1.2e3
35	100 ± 0	1.4e8 ± 0.6e8	1.6e3 ± 0.7e3	1.2e7 ± 0.6e7	1.4e4 ± 0.7e4	2.54e3 ± 1.1e3
36	100 ± 0	1.5e8 ± 0.6e8	1.5e3 ± 0.7e3	1.2e7 ± 0.6e7	1.4e4 ± 0.7e4	2.51e3 ± 1.1e3
37	100 ± 0	1.5e8 ± 0.6e8	1.4e3 ± 0.6e3	1.2e7 ± 0.6e7	1.4e4 ± 0.7e4	2.48e3 ± 1.1e3
38	100 ± 0	1.5e8 ± 0.6e8	1.4e3 ± 0.6e3	1.2e7 ± 0.6e7	1.3e4 ± 0.6e4	2.43e3 ± 1.1e3
39	100 ± 0	1.5e8 ± 0.6e8	1.3e3 ± 0.5e3	1.2e7 ± 0.5e7	1.3e4 ± 0.6e4	2.34e3 ± 1e3
40	100 ± 0	1.5e8 ± 0.6e8	1.2e3 ± 0.5e3	1.2e7 ± 0.5e7	1.3e4 ± 0.6e4	2.26e3 ± 1e3
41	100 ± 0	1.5e8 ± 0.6e8	1.2e3 ± 0.5e3	1.2e7 ± 0.5e7	1.2e4 ± 0.6e4	2.2e3 ± 0.9e3
42	100 ± 0	1.5e8 ± 0.6e8	1.1e3 ± 0.5e3	1.2e7 ± 0.5e7	1.3e4 ± 0.5e4	2.13e3 ± 0.9e3
43	100 ± 0	1.4e8 ± 0.6e8	1.1e3 ± 0.4e3	1.1e7 ± 0.5e7	1.2e4 ± 0.6e4	2.1e3 ± 0.9e3
44	100 ± 0	1.4e8 ± 0.6e8	1e3 ± 0.4e3	1.2e7 ± 0.5e7	1.2e4 ± 0.5e4	1.99e3 ± 0.8e3
45	100 ± 0	1.4e8 ± 0.6e8	9.5e2 ± 3.8e2	1.1e7 ± 0.5e7	1.2e4 ± 0.5e4	1.92e3 ± 0.8e3
46	100 ± 0	1.4e8 ± 0.6e8	9.2e2 ± 3.7e2	1.2e7 ± 0.5e7	1.2e4 ± 0.5e4	1.84e3 ± 0.7e3
47	100 ± 0	1.4e8 ± 0.6e8	8.8e2 ± 3.6e2	1.1e7 ± 0.5e7	1.1e4 ± 0.5e4	1.81e3 ± 0.7e3
48	100 ± 0	1.4e8 ± 0.6e8	8.5e2 ± 3.5e2	1.2e7 ± 0.5e7	1.2e4 ± 0.5e4	1.75e3 ± 0.7e3

The average of the prevalence, the CFU-values per square meter (cfu/sqm) and per gram manure are calculated over all iterations. The CFU values in the intestine as well as the excreted and ingested quantities are obtained by calculating the average over all animals in one iteration at first and then over all iterations.

Table S10b (log_{10} CFU). Calculated numeric values for scenario when day-old chicks are set positive (10^2 CFU bacteria per chick on day 1), measure slow growing breed. Setting: 90 chicks, 18 seeder, 72 sentinels, Rowan x Ranger, feeding duration 47 days, stocking density 39 kg/m^2; 1000 g litter/m^2

day	prevalence in %	cfu/sqm	cfu/g manure	cfu in intestine	cfu/g feces	cfu ingested per animal
	(% mean ± sd)	(log10 mean ± sd)	(log10 mean ± sd)	(log10 mean ± sd)	(log10 mean ± sd)	(log10 mean ± sd)
1	0 ± 0	-Inf ± -Inf	-Inf ± -Inf	-Inf ± -Inf	-Inf ± -Inf	1.3 ± 0
2	19.12 ± 0.87	4.95 ± 4.78	1.86 ± 1.69	4.17 ± 3.99	3.31 ± 3.13	-Inf ± 0
3	87.49 ± 12.77	5.45 ± 5.24	2.17 ± 1.97	4.59 ± 4.38	2.57 ± 2.36	1.36 ± 1.19
4	96.36 ± 8.11	5.8 ± 5.63	2.38 ± 2.21	4.9 ± 4.76	2.82 ± 2.67	1.61 ± 1.41
5	98.67 ± 4.02	6.08 ± 5.95	2.54 ± 2.41	5.16 ± 5.05	3.05 ± 2.93	1.82 ± 1.66
6	99.59 ± 1.82	6.31 ± 6.2	2.69 ± 2.59	5.37 ± 5.27	3.33 ± 3.24	2.03 ± 1.9
7	99.71 ± 1.35	6.52 ± 6.41	2.82 ± 2.71	5.57 ± 5.48	3.38 ± 3.28	2.2 ± 2.09
8	99.91 ± 0.54	6.7 ± 6.62	2.91 ± 2.83	5.74 ± 5.68	3.49 ± 3.43	2.36 ± 2.25
9	99.9 ± 0.53	6.87 ± 6.76	3.01 ± 2.9	5.91 ± 5.8	3.61 ± 3.51	2.51 ± 2.42
10	99.92 ± 0.51	7.03 ± 6.94	3.09 ± 3	6.05 ± 5.99	3.71 ± 3.64	2.63 ± 2.52
11	99.98 ± 0.16	7.18 ± 7.11	3.16 ± 3.1	6.2 ± 6.16	3.79 ± 3.75	2.75 ± 2.67
12	99.99 ± 0.11	7.29 ± 7.24	3.21 ± 3.16	6.29 ± 6.25	3.85 ± 3.81	2.85 ± 2.79
13	99.98 ± 0.16	7.39 ± 7.31	3.25 ± 3.17	6.38 ± 6.3	3.9 ± 3.83	2.94 ± 2.88
14	100 ± 0	7.48 ± 7.4	3.28 ± 3.21	6.46 ± 6.4	3.95 ± 3.89	3.01 ± 2.93
15	100 ± 0	7.57 ± 7.48	3.32 ± 3.23	6.56 ± 6.47	4.01 ± 3.93	3.07 ± 2.99
16	100 ± 0	7.65 ± 7.55	3.35 ± 3.25	6.63 ± 6.53	4.07 ± 3.97	3.14 ± 3.06
17	100 ± 0	7.73 ± 7.64	3.38 ± 3.28	6.71 ± 6.63	4.11 ± 4.03	3.2 ± 3.1
18	100 ± 0	7.79 ± 7.67	3.38 ± 3.27	6.75 ± 6.63	4.12 ± 4	3.25 ± 3.16
19	100 ± 0	7.83 ± 7.7	3.38 ± 3.25	6.78 ± 6.66	4.13 ± 4	3.29 ± 3.18
20	100 ± 0	7.88 ± 7.74	3.39 ± 3.25	6.84 ± 6.7	4.18 ± 4.04	3.31 ± 3.18
21	100 ± 0	7.93 ± 7.75	3.4 ± 3.22	6.88 ± 6.7	4.19 ± 4.01	3.35 ± 3.2
22	100 ± 0	7.97 ± 7.78	3.4 ± 3.21	6.91 ± 6.73	4.21 ± 4.03	3.38 ± 3.2
23	100 ± 0	8 ± 7.79	3.4 ± 3.19	6.95 ± 6.74	4.22 ± 4.02	3.4 ± 3.21
24	100 ± 0	8.03 ± 7.8	3.39 ± 3.16	6.97 ± 6.74	4.21 ± 3.98	3.42 ± 3.21
25	100 ± 0	8.06 ± 7.83	3.39 ± 3.15	7 ± 6.78	4.23 ± 4.01	3.43 ± 3.2
26	100 ± 0	8.07 ± 7.82	3.37 ± 3.12	7 ± 6.75	4.23 ± 3.98	3.45 ± 3.21
27	100 ± 0	8.08 ± 7.82	3.35 ± 3.09	7 ± 6.76	4.21 ± 3.96	3.44 ± 3.19
28	100 ± 0	8.1 ± 7.81	3.33 ± 3.05	7.02 ± 6.75	4.2 ± 3.93	3.44 ± 3.18
29	100 ± 0	8.12 ± 7.82	3.32 ± 3.02	7.05 ± 6.76	4.21 ± 3.92	3.44 ± 3.16
30	100 ± 0	8.13 ± 7.81	3.3 ± 2.98	7.05 ± 6.75	4.2 ± 3.91	3.44 ± 3.14
31	100 ± 0	8.14 ± 7.83	3.29 ± 2.97	7.07 ± 6.78	4.21 ± 3.92	3.44 ± 3.12
32	100 ± 0	8.15 ± 7.82	3.27 ± 2.94	7.07 ± 6.76	4.18 ± 3.87	3.44 ± 3.13
33	100 ± 0	8.15 ± 7.81	3.24 ± 2.9	7.07 ± 6.74	4.17 ± 3.84	3.43 ± 3.11
34	100 ± 0	8.15 ± 7.79	3.21 ± 2.86	7.05 ± 6.72	4.15 ± 3.81	3.42 ± 3.08
35	100 ± 0	8.16 ± 7.8	3.2 ± 2.84	7.08 ± 6.75	4.16 ± 3.83	3.4 ± 3.05
36	100 ± 0	8.16 ± 7.81	3.18 ± 2.83	7.08 ± 6.77	4.16 ± 3.84	3.4 ± 3.05
37	100 ± 0	8.17 ± 7.81	3.16 ± 2.8	7.08 ± 6.75	4.15 ± 3.82	3.39 ± 3.04
38	100 ± 0	8.17 ± 7.8	3.14 ± 2.77	7.08 ± 6.74	4.13 ± 3.79	3.38 ± 3.03
39	100 ± 0	8.16 ± 7.79	3.11 ± 2.74	7.07 ± 6.73	4.11 ± 3.77	3.37 ± 3.01
40	100 ± 0	8.17 ± 7.79	3.09 ± 2.71	7.08 ± 6.72	4.12 ± 3.76	3.35 ± 2.98
41	100 ± 0	8.16 ± 7.78	3.07 ± 2.69	7.07 ± 6.72	4.09 ± 3.74	3.34 ± 2.96
42	100 ± 0	8.17 ± 7.78	3.06 ± 2.67	7.09 ± 6.72	4.1 ± 3.73	3.33 ± 2.95
43	100 ± 0	8.16 ± 7.78	3.02 ± 2.65	7.06 ± 6.73	4.07 ± 3.75	3.32 ± 2.93
44	100 ± 0	8.15 ± 7.76	3 ± 2.61	7.06 ± 6.7	4.08 ± 3.71	3.3 ± 2.92
45	100 ± 0	8.15 ± 7.75	2.98 ± 2.58	7.06 ± 6.69	4.07 ± 3.69	3.28 ± 2.89
46	100 ± 0	8.15 ± 7.76	2.97 ± 2.57	7.07 ± 6.72	4.07 ± 3.71	3.27 ± 2.86
47	100 ± 0	8.15 ± 7.76	2.95 ± 2.56	7.06 ± 6.7	4.06 ± 3.7	3.26 ± 2.86
48	100 ± 0	8.15 ± 7.77	2.93 ± 2.55	7.07 ± 6.73	4.06 ± 3.72	3.24 ± 2.85

The average of the prevalence, the CFU-values per square meter (cfu/sqm) and per gram manure are calculated over all iterations. The CFU values in the intestine as well as the excreted and ingested quantities are obtained by calculating the average over all animals in one iteration at first and then over all iterations. The decadic logarithm of zero or negative values is negative infinite (-Inf).

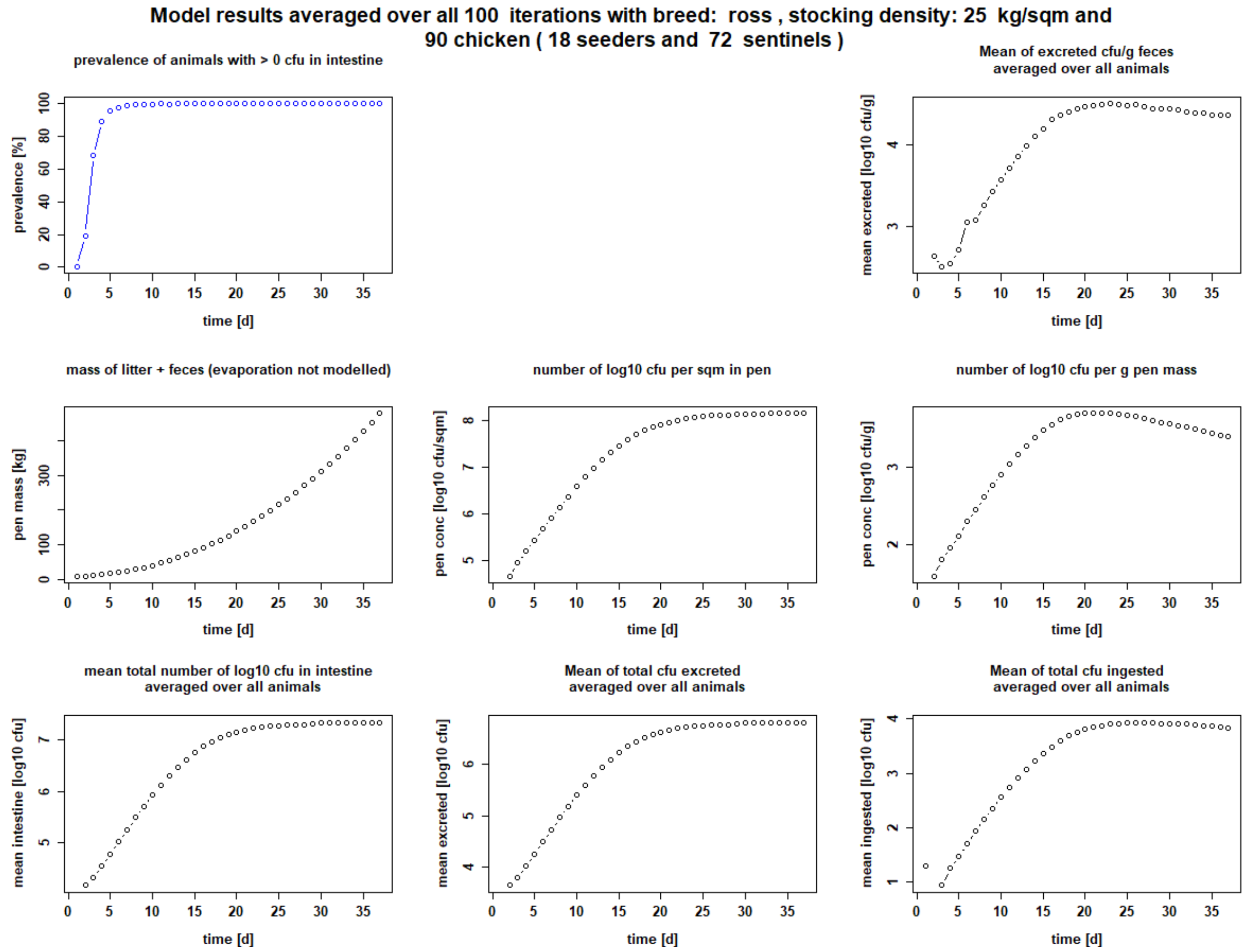

Figure S11. Calculated infection dynamic when day-old chicks are set positive (10^2 CFU bacteria per chick on day 1), measure stocking density 25 kg/m^2. Dotplots with results for 90 chicks, 18 seeder, 72 sentinels, Ross 308, feeding duration 36 days, stocking density 25 kg/m^2, 1000 g litter/m^2

Table S11a (CFU). Calculated numeric values for scenario when day-old chicks are set positive (10^2 CFU bacteria per chick on day 1), measure stocking density 25 kg/m^2. Setting**:** 90 chicks, 18 seeder, 72 sentinels, Ross 308, feeding duration 36 days; stocking density 25 kg/m^2, 1000 g litter/m^2

day	prevalence in %	cfu/sqm	cfu/g manure	cfu in intestine	cfu/g feces	cfu ingested per animal
	(mean ± sd)	(mean ± sd)	(mean ± sd)	(mean ± sd)	(mean ± sd)	(mean ± sd)
1	0 ± 0	0e0 ± 0	0e0 ± 0	0e0 ± 0	0e0 ± 0	2e1 ± 0e1
2	19.24 ± 0.92	4.7e4 ± 2.8e4	3.8e1 ± 2.3e1	1.4e4 ± 0.9e4	4.3e2 ± 2.7e2	0e0 ± 0
3	68.63 ± 24.76	8.9e4 ± 4.5e4	6.2e1 ± 3.2e1	2e4 ± 1.1e4	3.1e2 ± 1.7e2	8.61 ± 5.6
4	89.43 ± 12.88	1.6e5 ± 0.8e5	9.1e1 ± 4.8e1	3.5e4 ± 2e4	3.5e2 ± 2e2	1.79e1 ± 0.9e1
5	95.7 ± 9.4	2.7e5 ± 1.6e5	1.3e2 ± 0.8e2	5.9e4 ± 3.8e4	5.1e2 ± 3.2e2	3e1 ± 1.6e1
6	98.04 ± 6.89	4.7e5 ± 3.1e5	2e2 ± 1.3e2	1.1e5 ± 0.7e5	1.1e3 ± 0.8e3	4.95e1 ± 2.9e1
7	99.16 ± 4.15	8.1e5 ± 5.1e5	2.8e2 ± 1.8e2	1.8e5 ± 1.2e5	1.2e3 ± 0.8e3	8.71e1 ± 5.7e1
8	99.69 ± 1.75	1.4e6 ± 1e6	4e2 ± 2.8e2	3.1e5 ± 2.2e5	1.8e3 ± 1.3e3	1.4e2 ± 0.9e2
9	99.83 ± 1.2	2.3e6 ± 1.6e6	5.8e2 ± 3.9e2	5.1e5 ± 3.6e5	2.7e3 ± 1.9e3	2.25e2 ± 1.6e2
10	99.96 ± 0.31	3.8e6 ± 2.7e6	8e2 ± 5.7e2	8.3e5 ± 6.2e5	3.7e3 ± 2.8e3	3.62e2 ± 2.5e2
11	100 ± 0	6.1e6 ± 4.4e6	1.1e3 ± 0.8e3	1.3e6 ± 1e6	5.1e3 ± 3.8e3	5.51e2 ± 3.9e2
12	99.99 ± 0.11	9.5e6 ± 6.9e6	1.5e3 ± 1.1e3	2e6 ± 1.5e6	7.2e3 ± 5.4e3	8.25e2 ± 6e2
13	100 ± 0	1.4e7 ± 1e7	1.9e3 ± 1.4e3	2.9e6 ± 2.1e6	9.7e3 ± 7.1e3	1.18e3 ± 0.9e3
14	100 ± 0	2.1e7 ± 1.4e7	2.4e3 ± 1.7e3	4.2e6 ± 3e6	1.3e4 ± 0.9e4	1.67e3 ± 1.2e3
15	100 ± 0	2.9e7 ± 1.9e7	3e3 ± 2e3	5.7e6 ± 3.8e6	1.6e4 ± 1.1e4	2.33e3 ± 1.6e3
16	100 ± 0	3.9e7 ± 2.4e7	3.6e3 ± 2.2e3	7.6e6 ± 4.6e6	2.1e4 ± 1.2e4	3.07e3 ± 2e3
17	100 ± 0	5e7 ± 2.9e7	4.1e3 ± 2.4e3	9.4e6 ± 5.5e6	2.3e4 ± 1.4e4	3.95e3 ± 2.4e3
18	100 ± 0	6.1e7 ± 3.1e7	4.5e3 ± 2.3e3	1.1e7 ± 0.6e7	2.6e4 ± 1.3e4	4.85e3 ± 2.8e3
19	100 ± 0	7.2e7 ± 3.3e7	4.8e3 ± 2.2e3	1.3e7 ± 0.6e7	2.8e4 ± 1.3e4	5.66e3 ± 2.9e3
20	100 ± 0	8.2e7 ± 3.5e7	4.9e3 ± 2.1e3	1.4e7 ± 0.6e7	3e4 ± 1.3e4	6.43e3 ± 3e3
21	100 ± 0	9e7 ± 3.5e7	5e3 ± 1.9e3	1.5e7 ± 0.6e7	3e4 ± 1.2e4	7.04e3 ± 3e3
22	100 ± 0	9.9e7 ± 3.4e7	5e3 ± 1.7e3	1.7e7 ± 0.6e7	3.2e4 ± 1.1e4	7.5e3 ± 2.9e3
23	100 ± 0	1.1e8 ± 0.3e8	4.9e3 ± 1.5e3	1.8e7 ± 0.6e7	3.2e4 ± 1e4	7.95e3 ± 2.8e3
24	100 ± 0	1.2e8 ± 0.3e8	4.9e3 ± 1.4e3	1.9e7 ± 0.6e7	3.2e4 ± 1e4	8.29e3 ± 2.5e3
25	100 ± 0	1.2e8 ± 0.3e8	4.7e3 ± 1.2e3	1.9e7 ± 0.5e7	3.1e4 ± 0.9e4	8.54e3 ± 2.5e3
26	100 ± 0	1.2e8 ± 0.3e8	4.5e3 ± 1.1e3	2e7 ± 0.5e7	3.2e4 ± 0.8e4	8.54e3 ± 2.3e3
27	100 ± 0	1.3e8 ± 0.3e8	4.2e3 ± 0.9e3	2e7 ± 0.5e7	2.9e4 ± 0.7e4	8.64e3 ± 2.1e3
28	100 ± 0	1.3e8 ± 0.3e8	4e3 ± 0.8e3	2e7 ± 0.5e7	2.8e4 ± 0.7e4	8.45e3 ± 1.8e3
29	100 ± 0	1.3e8 ± 0.3e8	3.8e3 ± 0.7e3	2.1e7 ± 0.5e7	2.8e4 ± 0.7e4	8.28e3 ± 1.7e3
30	100 ± 0	1.3e8 ± 0.2e8	3.6e3 ± 0.7e3	2.1e7 ± 0.5e7	2.8e4 ± 0.6e4	8.2e3 ± 1.6e3
31	100 ± 0	1.4e8 ± 0.2e8	3.4e3 ± 0.6e3	2.1e7 ± 0.4e7	2.7e4 ± 0.5e4	8.15e3 ± 1.5e3
32	100 ± 0	1.4e8 ± 0.2e8	3.3e3 ± 0.5e3	2.2e7 ± 0.4e7	2.6e4 ± 0.5e4	7.95e3 ± 1.4e3
33	100 ± 0	1.4e8 ± 0.2e8	3.1e3 ± 0.5e3	2.2e7 ± 0.4e7	2.5e4 ± 0.5e4	7.76e3 ± 1.2e3
34	100 ± 0	1.4e8 ± 0.2e8	2.9e3 ± 0.4e3	2.2e7 ± 0.4e7	2.5e4 ± 0.5e4	7.51e3 ± 1.1e3
35	100 ± 0	1.4e8 ± 0.2e8	2.7e3 ± 0.4e3	2.2e7 ± 0.4e7	2.4e4 ± 0.5e4	7.35e3 ± 1e3
36	100 ± 0	1.4e8 ± 0.2e8	2.6e3 ± 0.4e3	2.2e7 ± 0.4e7	2.3e4 ± 0.5e4	7.12e3 ± 1e3
37	100 ± 0	1.4e8 ± 0.2e8	2.5e3 ± 0.3e3	2.2e7 ± 0.4e7	2.3e4 ± 0.5e4	6.89e3 ± 0.9e3

The average of the prevalence, the CFU-values per square meter (cfu/sqm) and per gram manure are calculated over all iterations. The CFU values in the intestine as well as the excreted and ingested quantities are obtained by calculating the average over all animals in one iteration at first and then over all iterations.

Table S11b (log_{10} CFU). Calculated numeric values for scenario when day-old chicks are set positive (10^2 CFU bacteria per chick on day 1), measure stocking density 25 kg/m^2. Setting: 90 chicks, 18 seeder, 72 sentinels, Ross 308, feeding duration 36 days; stocking density 25 kg/m^2, 1000 g litter/m^2

day	prevalence in %	cfu/sqm	cfu/g manure	cfu in intestine	cfu/g feces	cfu ingested per animal
	(% mean ± sd)	(log10 mean ± sd)	(log10 mean ± sd)	(log10 mean ± sd)	(log10 mean ± sd)	(log10 mean ± sd)
1	0 ± 0	-Inf ± -Inf	-Inf ± -Inf	-Inf ± -Inf	-Inf ± -Inf	1.3 ± 0
2	19.24 ± 0.92	4.67 ± 4.45	1.58 ± 1.37	4.16 ± 3.95	2.64 ± 2.42	-Inf ± 0
3	68.63 ± 24.76	4.95 ± 4.66	1.8 ± 1.5	4.31 ± 4.03	2.5 ± 2.22	0.94 ± 0.75
4	89.43 ± 12.88	5.2 ± 4.92	1.96 ± 1.68	4.55 ± 4.29	2.55 ± 2.29	1.25 ± 0.97
5	95.7 ± 9.4	5.43 ± 5.2	2.11 ± 1.88	4.77 ± 4.57	2.71 ± 2.51	1.48 ± 1.2
6	98.04 ± 6.89	5.68 ± 5.49	2.29 ± 2.11	5.02 ± 4.87	3.04 ± 2.89	1.69 ± 1.47
7	99.16 ± 4.15	5.91 ± 5.71	2.45 ± 2.25	5.25 ± 5.06	3.07 ± 2.88	1.94 ± 1.76
8	99.69 ± 1.75	6.14 ± 5.98	2.61 ± 2.44	5.49 ± 5.35	3.26 ± 3.12	2.15 ± 1.95
9	99.83 ± 1.2	6.37 ± 6.2	2.76 ± 2.59	5.71 ± 5.55	3.43 ± 3.28	2.35 ± 2.19
10	99.96 ± 0.31	6.58 ± 6.43	2.91 ± 2.75	5.92 ± 5.79	3.57 ± 3.44	2.56 ± 2.39
11	100 ± 0	6.79 ± 6.64	3.04 ± 2.9	6.12 ± 5.99	3.71 ± 3.58	2.74 ± 2.59
12	99.99 ± 0.11	6.98 ± 6.84	3.16 ± 3.03	6.3 ± 6.18	3.85 ± 3.73	2.92 ± 2.77
13	100 ± 0	7.15 ± 7.01	3.28 ± 3.13	6.47 ± 6.33	3.99 ± 3.85	3.07 ± 2.94
14	100 ± 0	7.31 ± 7.16	3.38 ± 3.23	6.62 ± 6.48	4.1 ± 3.96	3.22 ± 3.08
15	100 ± 0	7.46 ± 7.28	3.47 ± 3.29	6.76 ± 6.58	4.2 ± 4.02	3.37 ± 3.21
16	100 ± 0	7.59 ± 7.38	3.55 ± 3.34	6.88 ± 6.66	4.31 ± 4.1	3.49 ± 3.31
17	100 ± 0	7.69 ± 7.46	3.61 ± 3.38	6.97 ± 6.74	4.37 ± 4.13	3.6 ± 3.38
18	100 ± 0	7.78 ± 7.5	3.65 ± 3.36	7.05 ± 6.74	4.41 ± 4.1	3.69 ± 3.45
19	100 ± 0	7.86 ± 7.52	3.68 ± 3.35	7.11 ± 6.77	4.45 ± 4.11	3.75 ± 3.46
20	100 ± 0	7.91 ± 7.54	3.69 ± 3.32	7.15 ± 6.78	4.47 ± 4.11	3.81 ± 3.48
21	100 ± 0	7.96 ± 7.55	3.7 ± 3.29	7.19 ± 6.79	4.48 ± 4.08	3.85 ± 3.48
22	100 ± 0	8 ± 7.54	3.7 ± 3.24	7.22 ± 6.76	4.5 ± 4.04	3.88 ± 3.47
23	100 ± 0	8.03 ± 7.52	3.69 ± 3.18	7.25 ± 6.75	4.5 ± 4	3.9 ± 3.44
24	100 ± 0	8.06 ± 7.53	3.69 ± 3.15	7.28 ± 6.78	4.5 ± 4	3.92 ± 3.41
25	100 ± 0	8.08 ± 7.5	3.67 ± 3.09	7.28 ± 6.73	4.49 ± 3.93	3.93 ± 3.4
26	100 ± 0	8.1 ± 7.48	3.65 ± 3.04	7.31 ± 6.72	4.5 ± 3.91	3.93 ± 3.35
27	100 ± 0	8.1 ± 7.43	3.63 ± 2.96	7.3 ± 6.66	4.47 ± 3.82	3.94 ± 3.32
28	100 ± 0	8.11 ± 7.43	3.6 ± 2.92	7.3 ± 6.7	4.45 ± 3.85	3.93 ± 3.26
29	100 ± 0	8.12 ± 7.41	3.58 ± 2.87	7.32 ± 6.69	4.45 ± 3.82	3.92 ± 3.24
30	100 ± 0	8.13 ± 7.39	3.56 ± 2.82	7.33 ± 6.68	4.45 ± 3.8	3.91 ± 3.21
31	100 ± 0	8.14 ± 7.37	3.54 ± 2.77	7.33 ± 6.63	4.43 ± 3.73	3.91 ± 3.17
32	100 ± 0	8.14 ± 7.32	3.51 ± 2.7	7.34 ± 6.59	4.41 ± 3.67	3.9 ± 3.13
33	100 ± 0	8.14 ± 7.31	3.49 ± 2.65	7.33 ± 6.64	4.39 ± 3.7	3.89 ± 3.07
34	100 ± 0	8.15 ± 7.3	3.46 ± 2.62	7.34 ± 6.65	4.4 ± 3.7	3.88 ± 3.04
35	100 ± 0	8.15 ± 7.28	3.44 ± 2.58	7.34 ± 6.62	4.37 ± 3.66	3.87 ± 3.02
36	100 ± 0	8.15 ± 7.28	3.42 ± 2.55	7.34 ± 6.64	4.37 ± 3.67	3.85 ± 2.99
37	100 ± 0	8.15 ± 7.29	3.39 ± 2.53	7.35 ± 6.65	4.36 ± 3.67	3.84 ± 2.97

The average of the prevalence, the CFU-values per square meter (cfu/sqm) and per gram manure are calculated over all iterations. The CFU values in the intestine as well as the excreted and ingested quantities are obtained by calculating the average over all animals in one iteration at first and then over all iterations. The decadic logarithm of zero or negative values is negative infinite (-Inf).

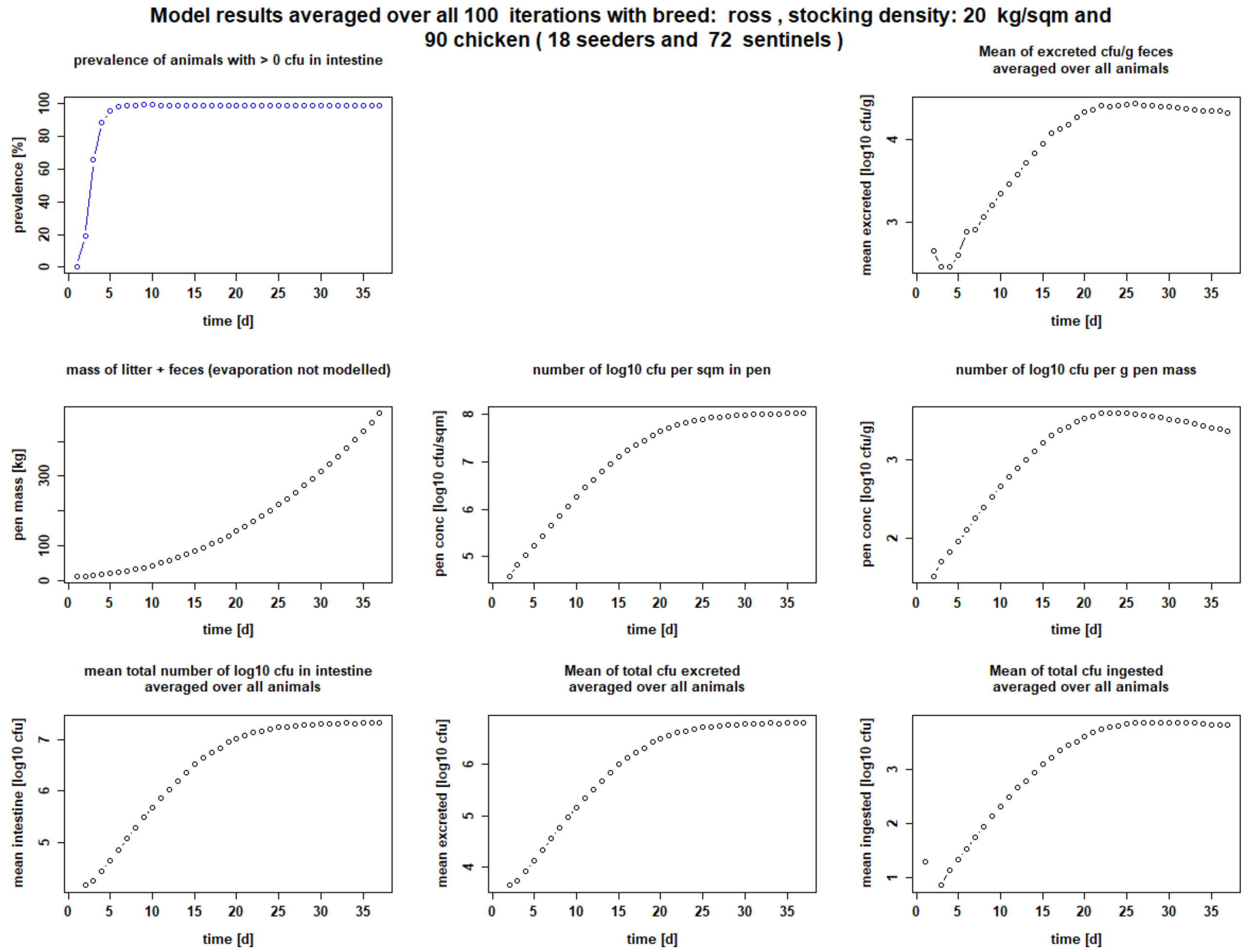

Figure S12. Calculated infection dynamic when day-old chicks are set positive (10^2 CFU bacteria per chick on day 1), measure stocking density 20 kg/m². Dotplots with results for 90 chicks, 18 seeder, 72 sentinels, Ross 308, feeding duration 36 days, stocking density 20 kg/m², 1000 g litter/m²

Table S12a (CFU). Calculated numeric values for scenario when day-old chicks are set positive (10^2 CFU bacteria per chick on day 1), measure stocking density 20 kg/m^2. Setting**:** 90 chicks, 18 seeder, 72 sentinels, Ross 308, feeding duration 36 days; stocking density 20 kg/m^2, 1000 g litter/m^2

day	prevalence in %	cfu/sqm	cfu/g manure	cfu in intestine	cfu/g feces	cfu ingested per animal
	(mean ± sd)	(mean ± sd)	(mean ± sd)	(mean ± sd)	(mean ± sd)	(mean ± sd)
1	0 ± 0	0e0 ± 0	0e0 ± 0	0e0 ± 0	0e0 ± 0	2e1 ± 0e1
2	19.08 ± 1.04	3.8e4 ± 2.3e4	3.3e1 ± 1.9e1	1.5e4 ± 0.9e4	4.5e2 ± 2.6e2	0e0 ± 0
3	65.72 ± 23.58	6.7e4 ± 2.9e4	5e1 ± 2.2e1	1.9e4 ± 0.8e4	2.9e2 ± 1.3e2	7.26 ± 4.6
4	88.5 ± 11.42	1.1e5 ± 0.5e5	6.6e1 ± 2.9e1	2.8e4 ± 1.3e4	2.8e2 ± 1.3e2	1.41e1 ± 0.6e1
5	95.91 ± 7.09	1.7e5 ± 0.9e5	9e1 ± 4.5e1	4.6e4 ± 2.5e4	3.9e2 ± 2.2e2	2.17e1 ± 1e1
6	98.21 ± 6.19	2.7e5 ± 1.4e5	1.3e2 ± 0.7e2	7.2e4 ± 3.9e4	7.5e2 ± 4.1e2	3.46e1 ± 1.8e1
7	99.04 ± 5.68	4.5e5 ± 2.7e5	1.8e2 ± 1.1e2	1.2e5 ± 0.8e5	8e2 ± 5.3e2	5.59e1 ± 2.9e1
8	99.31 ± 6.24	7.3e5 ± 4.4e5	2.5e2 ± 1.5e2	2e5 ± 1.2e5	1.2e3 ± 0.7e3	8.86e1 ± 5.3e1
9	99.42 ± 5.56	1.1e6 ± 0.7e6	3.3e2 ± 1.9e2	3e5 ± 1.8e5	1.6e3 ± 0.9e3	1.37e2 ± 0.8e2
10	99.52 ± 4.78	1.8e6 ± 1.2e6	4.5e2 ± 2.9e2	4.9e5 ± 3.4e5	2.2e3 ± 1.5e3	2.09e2 ± 1.2e2
11	99.34 ± 6.56	2.8e6 ± 1.8e6	6e2 ± 3.9e2	7.4e5 ± 5e5	2.9e3 ± 2e3	3.11e2 ± 2e2
12	99.13 ± 8.56	4.1e6 ± 2.8e6	7.7e2 ± 5.2e2	1.1e6 ± 0.8e6	3.8e3 ± 2.7e3	4.56e2 ± 3e2
13	99.19 ± 8.11	6.1e6 ± 4.1e6	10e2 ± 6.7e2	1.6e6 ± 1.1e6	5.3e3 ± 3.7e3	6.22e2 ± 4.2e2
14	99.16 ± 8.44	8.9e6 ± 6.2e6	1.3e3 ± 0.9e3	2.3e6 ± 1.6e6	6.9e3 ± 5e3	8.78e2 ± 5.9e2
15	99.14 ± 8.56	1.3e7 ± 0.9e7	1.6e3 ± 1.2e3	3.2e6 ± 2.5e6	9.1e3 ± 7e3	1.23e3 ± 0.9e3
16	99.13 ± 8.67	1.8e7 ± 1.3e7	2e3 ± 1.5e3	4.4e6 ± 3.3e6	1.2e4 ± 0.9e4	1.67e3 ± 1.2e3
17	99.11 ± 8.89	2.3e7 ± 1.6e7	2.3e3 ± 1.6e3	5.6e6 ± 3.8e6	1.4e4 ± 0.9e4	2.2e3 ± 1.6e3
18	99.17 ± 8.33	2.9e7 ± 1.8e7	2.6e3 ± 1.6e3	6.7e6 ± 4.1e6	1.5e4 ± 0.9e4	2.78e3 ± 1.9e3
19	99.11 ± 8.89	3.7e7 ± 2.1e7	3e3 ± 1.7e3	8.7e6 ± 4.8e6	1.9e4 ± 1.1e4	3.28e3 ± 2.1e3
20	99.13 ± 8.67	4.5e7 ± 2.3e7	3.3e3 ± 1.7e3	1e7 ± 0.5e7	2.2e4 ± 1.1e4	4.05e3 ± 2.3e3
21	99.09 ± 9.11	5.3e7 ± 2.5e7	3.6e3 ± 1.7e3	1.2e7 ± 0.6e7	2.3e4 ± 1.1e4	4.77e3 ± 2.5e3
22	99.11 ± 8.89	6.2e7 ± 2.6e7	3.8e3 ± 1.6e3	1.4e7 ± 0.6e7	2.6e4 ± 1.1e4	5.44e3 ± 2.6e3
23	99.12 ± 8.78	6.8e7 ± 2.5e7	3.8e3 ± 1.4e3	1.4e7 ± 0.5e7	2.6e4 ± 1e4	6.12e3 ± 2.6e3
24	99.13 ± 8.67	7.4e7 ± 2.4e7	3.9e3 ± 1.3e3	1.6e7 ± 0.5e7	2.6e4 ± 0.9e4	6.47e3 ± 2.4e3
25	99.09 ± 9.11	8e7 ± 2.4e7	3.9e3 ± 1.2e3	1.7e7 ± 0.5e7	2.7e4 ± 0.9e4	6.79e3 ± 2.2e3
26	99.09 ± 9.11	8.5e7 ± 2.4e7	3.8e3 ± 1.1e3	1.7e7 ± 0.5e7	2.7e4 ± 0.8e4	7.12e3 ± 2.2e3
27	99.1 ± 9	8.8e7 ± 2.2e7	3.6e3 ± 0.9e3	1.8e7 ± 0.5e7	2.6e4 ± 0.7e4	7.3e3 ± 2.1e3
28	99.1 ± 9	9.2e7 ± 2.2e7	3.5e3 ± 0.8e3	1.9e7 ± 0.5e7	2.6e4 ± 0.7e4	7.31e3 ± 1.8e3
29	99.08 ± 9.22	9.5e7 ± 2.2e7	3.4e3 ± 0.8e3	1.9e7 ± 0.5e7	2.5e4 ± 0.7e4	7.38e3 ± 1.8e3
30	99.09 ± 9.11	9.8e7 ± 2.2e7	3.3e3 ± 0.7e3	2e7 ± 0.5e7	2.5e4 ± 0.7e4	7.32e3 ± 1.7e3
31	99.04 ± 9.56	1e8 ± 0.2e8	3.1e3 ± 0.7e3	2e7 ± 0.5e7	2.5e4 ± 0.6e4	7.32e3 ± 1.6e3
32	99.07 ± 9.33	1e8 ± 0.2e8	3e3 ± 0.6e3	2e7 ± 0.5e7	2.4e4 ± 0.6e4	7.25e3 ± 1.5e3
33	99.07 ± 9.33	1e8 ± 0.2e8	2.8e3 ± 0.5e3	2e7 ± 0.5e7	2.4e4 ± 0.5e4	7.11e3 ± 1.4e3
34	99.06 ± 9.44	1e8 ± 0.2e8	2.7e3 ± 0.5e3	2e7 ± 0.5e7	2.3e4 ± 0.5e4	6.98e3 ± 1.3e3
35	99.03 ± 9.67	1e8 ± 0.2e8	2.6e3 ± 0.5e3	2.1e7 ± 0.5e7	2.2e4 ± 0.5e4	6.75e3 ± 1.2e3
36	99.03 ± 9.67	1.1e8 ± 0.2e8	2.5e3 ± 0.4e3	2.1e7 ± 0.5e7	2.3e4 ± 0.5e4	6.61e3 ± 1.2e3
37	99.03 ± 9.67	1.1e8 ± 0.2e8	2.3e3 ± 0.4e3	2e7 ± 0.4e7	2.1e4 ± 0.4e4	6.52e3 ± 1.1e3

The average of the prevalence, the CFU-values per square meter (cfu/sqm) and per gram manure are calculated over all iterations. The CFU values in the intestine as well as the excreted and ingested quantities are obtained by calculating the average over all animals in one iteration at first and then over all iterations.

Table S12b (log_{10} CFU). Calculated numeric values for scenario when day-old chicks are set positive (10^2 CFU bacteria per chick on day 1), measure stocking density 20 kg/m². Setting**:** 90 chicks, 18 seeder, 72 sentinels, Ross 308, feeding duration 36 days; stocking density 20 kg/m², 1000 g litter/m²

day	prevalence in %	cfu/sqm	cfu/g manure	cfu in intestine	cfu/g feces	cfu ingested per animal
	(% mean ± sd)	(log10 mean ± sd)	(log10 mean ± sd)	(log10 mean ± sd)	(log10 mean ± sd)	(log10 mean ± sd)
1	0 ± 0	-Inf ± -Inf	-Inf ± -Inf	-Inf ± -Inf	-Inf ± -Inf	1.3 ± 0
2	19.08 ± 1.04	4.58 ± 4.35	1.51 ± 1.28	4.17 ± 3.94	2.65 ± 2.42	-Inf ± 0
3	65.72 ± 23.58	4.82 ± 4.47	1.7 ± 1.34	4.27 ± 3.91	2.46 ± 2.1	0.86 ± 0.66
4	88.5 ± 11.42	5.02 ± 4.67	1.82 ± 1.47	4.45 ± 4.13	2.45 ± 2.13	1.15 ± 0.81
5	95.91 ± 7.09	5.23 ± 4.93	1.95 ± 1.65	4.66 ± 4.4	2.59 ± 2.34	1.34 ± 0.99
6	98.21 ± 6.19	5.43 ± 5.14	2.1 ± 1.81	4.86 ± 4.59	2.88 ± 2.61	1.54 ± 1.25
7	99.04 ± 5.68	5.65 ± 5.43	2.25 ± 2.03	5.08 ± 4.9	2.91 ± 2.72	1.75 ± 1.47
8	99.31 ± 6.24	5.86 ± 5.65	2.39 ± 2.18	5.29 ± 5.1	3.06 ± 2.87	1.95 ± 1.73
9	99.42 ± 5.56	6.06 ± 5.83	2.52 ± 2.29	5.48 ± 5.25	3.21 ± 2.97	2.14 ± 1.92
10	99.52 ± 4.78	6.26 ± 6.07	2.66 ± 2.46	5.69 ± 5.53	3.34 ± 3.18	2.32 ± 2.09
11	99.34 ± 6.56	6.45 ± 6.26	2.78 ± 2.59	5.87 ± 5.7	3.47 ± 3.3	2.49 ± 2.3
12	99.13 ± 8.56	6.62 ± 6.45	2.88 ± 2.72	6.02 ± 5.88	3.58 ± 3.44	2.66 ± 2.47
13	99.19 ± 8.11	6.79 ± 6.62	3 ± 2.83	6.2 ± 6.04	3.72 ± 3.57	2.79 ± 2.63
14	99.16 ± 8.44	6.95 ± 6.79	3.11 ± 2.95	6.36 ± 6.22	3.84 ± 3.7	2.94 ± 2.77
15	99.14 ± 8.56	7.11 ± 6.97	3.21 ± 3.07	6.51 ± 6.4	3.96 ± 3.85	3.09 ± 2.93
16	99.13 ± 8.67	7.25 ± 7.12	3.3 ± 3.17	6.64 ± 6.52	4.08 ± 3.96	3.22 ± 3.09
17	99.11 ± 8.89	7.36 ± 7.21	3.37 ± 3.21	6.75 ± 6.58	4.14 ± 3.98	3.34 ± 3.21
18	99.17 ± 8.33	7.46 ± 7.26	3.42 ± 3.21	6.82 ± 6.62	4.18 ± 3.98	3.44 ± 3.29
19	99.11 ± 8.89	7.57 ± 7.32	3.48 ± 3.23	6.94 ± 6.68	4.28 ± 4.02	3.52 ± 3.31
20	99.13 ± 8.67	7.65 ± 7.37	3.52 ± 3.24	7.01 ± 6.73	4.34 ± 4.05	3.61 ± 3.36
21	99.09 ± 9.11	7.72 ± 7.4	3.56 ± 3.23	7.07 ± 6.75	4.37 ± 4.04	3.68 ± 3.39
22	99.11 ± 8.89	7.79 ± 7.41	3.58 ± 3.21	7.14 ± 6.75	4.41 ± 4.03	3.74 ± 3.41
23	99.12 ± 8.78	7.83 ± 7.4	3.59 ± 3.15	7.15 ± 6.73	4.41 ± 3.98	3.79 ± 3.41
24	99.13 ± 8.67	7.87 ± 7.38	3.59 ± 3.1	7.2 ± 6.72	4.41 ± 3.94	3.81 ± 3.38
25	99.09 ± 9.11	7.9 ± 7.39	3.59 ± 3.07	7.23 ± 6.73	4.43 ± 3.93	3.83 ± 3.35
26	99.09 ± 9.11	7.93 ± 7.38	3.58 ± 3.03	7.24 ± 6.73	4.44 ± 3.92	3.85 ± 3.33
27	99.1 ± 9	7.94 ± 7.35	3.56 ± 2.96	7.25 ± 6.69	4.42 ± 3.86	3.86 ± 3.31
28	99.1 ± 9	7.96 ± 7.34	3.55 ± 2.92	7.27 ± 6.69	4.42 ± 3.83	3.86 ± 3.27
29	99.08 ± 9.22	7.98 ± 7.34	3.53 ± 2.89	7.28 ± 6.69	4.4 ± 3.81	3.87 ± 3.24
30	99.09 ± 9.11	7.99 ± 7.34	3.51 ± 2.86	7.29 ± 6.72	4.41 ± 3.84	3.86 ± 3.23
31	99.04 ± 9.56	8 ± 7.32	3.5 ± 2.81	7.3 ± 6.67	4.39 ± 3.76	3.86 ± 3.21
32	99.07 ± 9.33	8.01 ± 7.31	3.48 ± 2.77	7.3 ± 6.67	4.38 ± 3.74	3.86 ± 3.18
33	99.07 ± 9.33	8.01 ± 7.29	3.45 ± 2.73	7.31 ± 6.67	4.37 ± 3.73	3.85 ± 3.15
34	99.06 ± 9.44	8.01 ± 7.28	3.43 ± 2.69	7.3 ± 6.69	4.36 ± 3.74	3.84 ± 3.12
35	99.03 ± 9.67	8.02 ± 7.28	3.41 ± 2.66	7.31 ± 6.66	4.35 ± 3.7	3.83 ± 3.09
36	99.03 ± 9.67	8.03 ± 7.26	3.39 ± 2.63	7.33 ± 6.66	4.35 ± 3.69	3.82 ± 3.08
37	99.03 ± 9.67	8.03 ± 7.24	3.36 ± 2.58	7.31 ± 6.62	4.33 ± 3.64	3.81 ± 3.05

The average of the prevalence, the CFU-values per square meter (cfu/sqm) and per gram manure are calculated over all iterations. The CFU values in the intestine as well as the excreted and ingested quantities are obtained by calculating the average over all animals in one iteration at first and then over all iterations. The decadic logarithm of zero or negative values is negative infinite (-Inf).

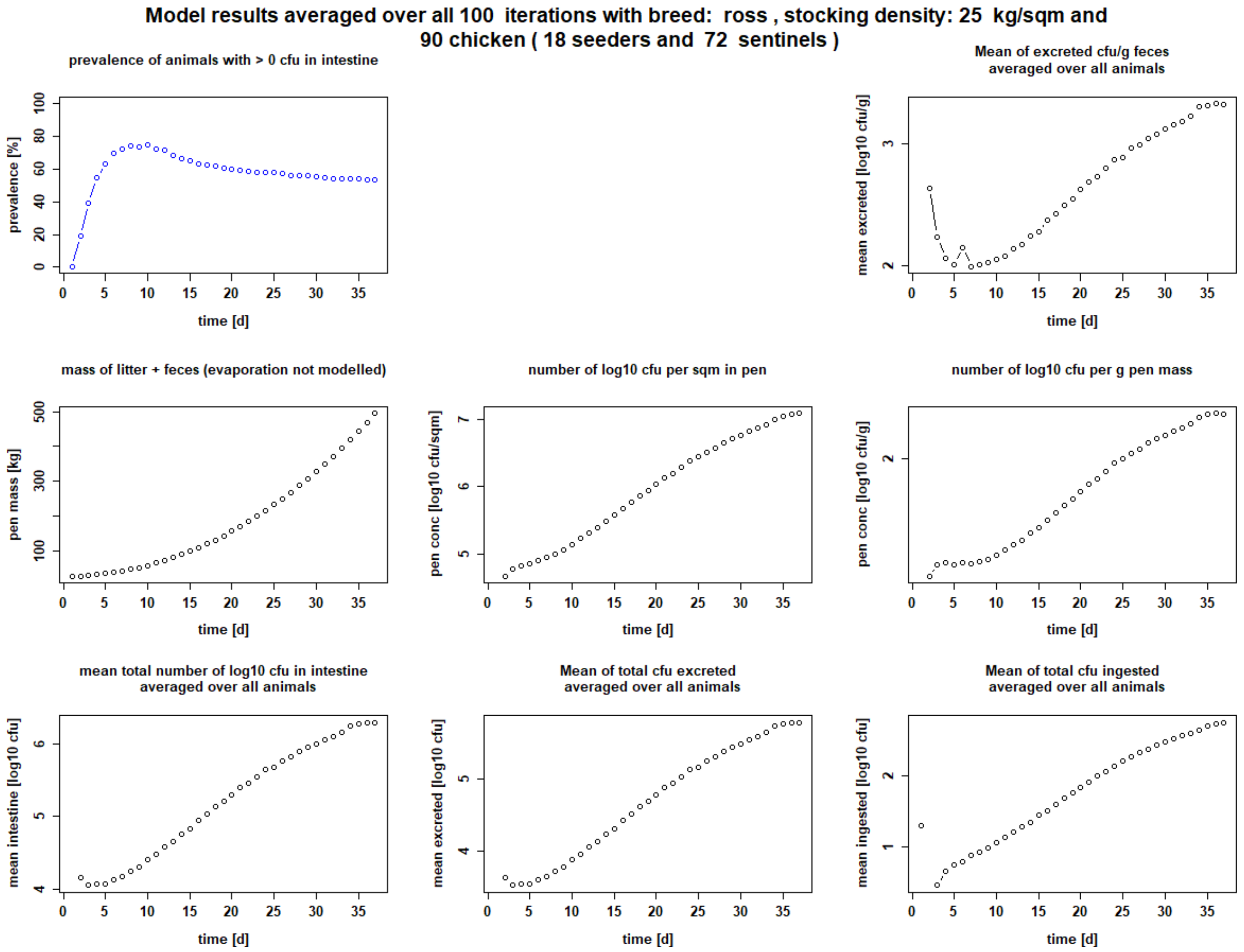

Figure S13. Calculated infection dynamic when day-old chicks are set positive (10^2 CFU bacteria per chick on day 1), combined measures litter 3000 g/m^2 and stocking density 25 kg/m^2. Dotplots with results for 90 chicks, 18 seeder, 72 sentinels, Ross 308, feeding duration 36 days, stocking density 25 kg/m^2, 3000 g litter/m^2

Table S13a (CFU). Calculated numeric values for scenario when day-old chicks are set positive (10^2 CFU bacteria per chick on day 1), combined measures litter 3000 g/m^2 and stocking density 25 kg/m^2. Setting: 90 chicks, 18 seeder, 72 sentinels, Ross 308, feeding duration 36 days, stocking density 20 kg/m^2, 3000 g litter/m^2

day	prevalence in %	cfu/sqm	cfu/g manure	cfu in intestine	cfu/g feces	cfu ingested per animal
	(mean ± sd)	(mean ± sd)	(mean ± sd)	(mean ± sd)	(mean ± sd)	(mean ± sd)
1	0 ± 0	0e0 ± 0	0e0 ± 0	0e0 ± 0	0e0 ± 0	2e1 ± 0e1
2	19.22 ± 0.84	4.6e4 ± 2.9e4	1.4e1 ± 0.9e1	1.4e4 ± 0.9e4	4.3e2 ± 2.7e2	0e0 ± 0
3	39.33 ± 22.23	5.9e4 ± 2.9e4	1.7e1 ± 0.9e1	1.1e4 ± 0.7e4	1.7e2 ± 1e2	2.9 ± 2.1
4	54.74 ± 24.1	6.7e4 ± 3.5e4	1.8e1 ± 0.9e1	1.2e4 ± 0.7e4	1.2e2 ± 0.7e2	4.58 ± 2.6
5	63.43 ± 23.91	7.2e4 ± 3.6e4	1.7e1 ± 0.9e1	1.2e4 ± 0.7e4	1e2 ± 0.6e2	5.54 ± 3.1
6	69.71 ± 22.93	7.9e4 ± 4.8e4	1.8e1 ± 1.1e1	1.4e4 ± 1e4	1.4e2 ± 1e2	6.29 ± 3.5
7	72.12 ± 24.74	8.7e4 ± 6.1e4	1.8e1 ± 1.2e1	1.5e4 ± 1.2e4	9.8e1 ± 7.8e1	7.58 ± 4.9
8	74.42 ± 25.49	10e4 ± 7.7e4	1.8e1 ± 1.4e1	1.7e4 ± 1.5e4	1e2 ± 0.9e2	8.49 ± 6.2
9	73.97 ± 28.29	1.1e5 ± 0.9e5	1.9e1 ± 1.6e1	2e4 ± 1.8e4	1.1e2 ± 0.9e2	9.71 ± 7.9
10	74.8 ± 28.43	1.4e5 ± 1.4e5	2e1 ± 2e1	2.5e4 ± 2.8e4	1.1e2 ± 1.3e2	1.14e1 ± 1e1
11	72.62 ± 31.85	1.7e5 ± 1.8e5	2.2e1 ± 2.4e1	3e4 ± 3.5e4	1.2e2 ± 1.4e2	1.35e1 ± 1.4e1
12	71.64 ± 34	2.1e5 ± 2.4e5	2.4e1 ± 2.8e1	3.8e4 ± 4.8e4	1.4e2 ± 1.7e2	1.61e1 ± 1.8e1
13	68.66 ± 37.24	2.5e5 ± 3.1e5	2.6e1 ± 3.3e1	4.5e4 ± 6e4	1.5e2 ± 2e2	1.92e1 ± 2.3e1
14	66.72 ± 39.1	3.1e5 ± 4e5	2.9e1 ± 3.8e1	5.8e4 ± 7.7e4	1.7e2 ± 2.3e2	2.25e1 ± 2.9e1
15	65.31 ± 40.12	3.7e5 ± 5.1e5	3.2e1 ± 4.4e1	6.8e4 ± 9.9e4	1.9e2 ± 2.8e2	2.78e1 ± 3.7e1
16	63.28 ± 41.74	4.7e5 ± 6.7e5	3.6e1 ± 5.2e1	8.7e4 ± 13.2e4	2.4e2 ± 3.6e2	3.27e1 ± 4.5e1
17	62.59 ± 42.09	5.8e5 ± 8.8e5	4.1e1 ± 6.2e1	1.1e5 ± 1.7e5	2.6e2 ± 4.3e2	3.96e1 ± 5.8e1
18	61.77 ± 42.99	7.2e5 ± 11.5e5	4.6e1 ± 7.4e1	1.3e5 ± 2.2e5	3.1e2 ± 5.1e2	4.79e1 ± 7.4e1
19	60.87 ± 43.53	8.8e5 ± 13.8e5	5.1e1 ± 8.1e1	1.6e5 ± 2.5e5	3.5e2 ± 5.6e2	5.79e1 ± 9.3e1
20	60.29 ± 43.46	1.1e6 ± 1.8e6	5.8e1 ± 9.5e1	2e5 ± 3.4e5	4.2e2 ± 7.1e2	6.86e1 ± 10.9e1
21	59.62 ± 43.96	1.3e6 ± 2.2e6	6.6e1 ± 10.7e1	2.5e5 ± 4.1e5	4.9e2 ± 8e2	8.23e1 ± 13.5e1
22	58.8 ± 44.49	1.6e6 ± 2.6e6	7.2e1 ± 11.7e1	2.8e5 ± 4.7e5	5.4e2 ± 8.8e2	9.92e1 ± 16.2e1
23	58.36 ± 44.75	1.9e6 ± 3.1e6	8.1e1 ± 13.3e1	3.5e5 ± 5.9e5	6.3e2 ± 10.5e2	1.14e2 ± 1.9e2
24	58.03 ± 44.91	2.4e6 ± 4e6	9.3e1 ± 15.6e1	4.4e5 ± 7.6e5	7.3e2 ± 12.6e2	1.36e2 ± 2.2e2
25	57.89 ± 44.78	2.7e6 ± 4.6e6	9.9e1 ± 16.7e1	4.8e5 ± 8.2e5	7.7e2 ± 13.2e2	1.62e2 ± 2.7e2
26	57.19 ± 45.03	3.2e6 ± 5.4e6	1.1e2 ± 1.8e2	5.8e5 ± 9.8e5	9.1e2 ± 15.4e2	1.81e2 ± 3.1e2
27	55.91 ± 45.84	3.7e6 ± 6.4e6	1.2e2 ± 2e2	6.6e5 ± 11.5e5	9.7e2 ± 16.9e2	2.08e2 ± 3.5e2
28	56.22 ± 45.63	4.4e6 ± 7.4e6	1.3e2 ± 2.2e2	7.8e5 ± 13.4e5	1.1e3 ± 1.9e3	2.34e2 ± 4e2
29	56.14 ± 45.7	5.1e6 ± 8.7e6	1.4e2 ± 2.4e2	9e5 ± 15.8e5	1.2e3 ± 2.1e3	2.67e2 ± 4.5e2
30	55.23 ± 46.4	5.7e6 ± 9.8e6	1.5e2 ± 2.5e2	10e5 ± 17e5	1.3e3 ± 2.2e3	2.99e2 ± 5.1e2
31	54.87 ± 46.34	6.6e6 ± 11.1e6	1.6e2 ± 2.7e2	1.1e6 ± 1.9e6	1.4e3 ± 2.4e3	3.29e2 ± 5.6e2
32	54.41 ± 46.77	7.4e6 ± 12.2e6	1.7e2 ± 2.7e2	1.3e6 ± 2.1e6	1.5e3 ± 2.5e3	3.63e2 ± 6.1e2
33	54.47 ± 46.71	8.3e6 ± 13.9e6	1.8e2 ± 2.9e2	1.4e6 ± 2.5e6	1.7e3 ± 2.9e3	3.95e2 ± 6.5e2
34	54.27 ± 46.91	9.8e6 ± 16.7e6	2e2 ± 3.3e2	1.8e6 ± 3.1e6	2e3 ± 3.5e3	4.32e2 ± 7.2e2
35	53.88 ± 47.32	1.1e7 ± 1.9e7	2.1e2 ± 3.6e2	1.9e6 ± 3.4e6	2e3 ± 3.7e3	4.94e2 ± 8.4e2
36	53.62 ± 47.58	1.2e7 ± 2e7	2.1e2 ± 3.6e2	2e6 ± 3.3e6	2.1e3 ± 3.5e3	5.35e2 ± 9.4e2
37	53.32 ± 47.88	1.2e7 ± 2e7	2.1e2 ± 3.4e2	2e6 ± 3.2e6	2.1e3 ± 3.4e3	5.57e2 ± 9.5e2

The average of the prevalence, the CFU-values per square meter (cfu/sqm) and per gram manure are calculated over all iterations. The CFU values in the intestine as well as the excreted and ingested quantities are obtained by calculating the average over all animals in one iteration at first and then over all iterations.

Table S13b (log_{10} CFU). Calculated numeric values for scenario when day-old chicks are set positive (10^2 CFU bacteria per chick on day 1), combined measures litter 3000 g/m^2 and stocking density 25 kg/m^2. Setting: 90 chicks, 18 seeder, 72 sentinels, Ross 308, feeding duration 36 days, stocking density 20 kg/m^2, 3000 g litter/m^2

day	prevalence in %	cfu/sqm	cfu/g manure	cfu in intestine	cfu/g feces	cfu ingested per animal
	(% mean ± sd)	(log10 mean ± sd)	(log10 mean ± sd)	(log10 mean ± sd)	(log10 mean ± sd)	(log10 mean ± sd)
1	0 ± 0	-Inf ± -Inf	-Inf ± -Inf	-Inf ± -Inf	-Inf ± -Inf	1.3 ± 0
2	19.22 ± 0.84	4.66 ± 4.46	1.16 ± 0.95	4.16 ± 3.95	2.63 ± 2.43	-Inf ± 0
3	39.33 ± 22.23	4.77 ± 4.47	1.24 ± 0.93	4.05 ± 3.82	2.24 ± 2.01	0.46 ± 0.33
4	54.74 ± 24.1	4.83 ± 4.54	1.25 ± 0.97	4.07 ± 3.86	2.06 ± 1.86	0.66 ± 0.41
5	63.43 ± 23.91	4.86 ± 4.56	1.24 ± 0.95	4.08 ± 3.82	2.01 ± 1.75	0.74 ± 0.5
6	69.71 ± 22.93	4.9 ± 4.68	1.25 ± 1.04	4.13 ± 3.99	2.15 ± 2.01	0.8 ± 0.55
7	72.12 ± 24.74	4.94 ± 4.78	1.25 ± 1.09	4.17 ± 4.07	1.99 ± 1.89	0.88 ± 0.69
8	74.42 ± 25.49	5 ± 4.89	1.26 ± 1.15	4.24 ± 4.18	2.01 ± 1.95	0.93 ± 0.79
9	73.97 ± 28.29	5.06 ± 4.98	1.27 ± 1.19	4.3 ± 4.25	2.02 ± 1.98	0.99 ± 0.9
10	74.8 ± 28.43	5.14 ± 5.14	1.31 ± 1.31	4.4 ± 4.45	2.06 ± 2.11	1.05 ± 0.99
11	72.62 ± 31.85	5.22 ± 5.25	1.34 ± 1.37	4.48 ± 4.55	2.08 ± 2.14	1.13 ± 1.14
12	71.64 ± 34	5.31 ± 5.38	1.39 ± 1.45	4.58 ± 4.68	2.14 ± 2.23	1.21 ± 1.25
13	68.66 ± 37.24	5.39 ± 5.49	1.42 ± 1.52	4.65 ± 4.78	2.17 ± 2.3	1.28 ± 1.36
14	66.72 ± 39.1	5.49 ± 5.6	1.47 ± 1.58	4.76 ± 4.89	2.24 ± 2.37	1.35 ± 1.46
15	65.31 ± 40.12	5.57 ± 5.71	1.51 ± 1.64	4.83 ± 5	2.28 ± 2.44	1.44 ± 1.57
16	63.28 ± 41.74	5.67 ± 5.83	1.56 ± 1.72	4.94 ± 5.12	2.37 ± 2.55	1.51 ± 1.66
17	62.59 ± 42.09	5.76 ± 5.94	1.61 ± 1.79	5.03 ± 5.24	2.42 ± 2.63	1.6 ± 1.76
18	61.77 ± 42.99	5.86 ± 6.06	1.67 ± 1.87	5.13 ± 5.35	2.49 ± 2.71	1.68 ± 1.87
19	60.87 ± 43.53	5.94 ± 6.14	1.71 ± 1.91	5.2 ± 5.41	2.54 ± 2.75	1.76 ± 1.97
20	60.29 ± 43.46	6.03 ± 6.24	1.76 ± 1.98	5.3 ± 5.53	2.62 ± 2.85	1.84 ± 2.04
21	59.62 ± 43.96	6.12 ± 6.34	1.82 ± 2.03	5.39 ± 5.61	2.69 ± 2.9	1.92 ± 2.13
22	58.8 ± 44.49	6.2 ± 6.41	1.86 ± 2.07	5.45 ± 5.67	2.73 ± 2.95	2 ± 2.21
23	58.36 ± 44.75	6.28 ± 6.5	1.91 ± 2.12	5.55 ± 5.77	2.8 ± 3.02	2.06 ± 2.27
24	58.03 ± 44.91	6.38 ± 6.6	1.97 ± 2.19	5.64 ± 5.88	2.86 ± 3.1	2.13 ± 2.35
25	57.89 ± 44.78	6.44 ± 6.66	2 ± 2.22	5.68 ± 5.92	2.89 ± 3.12	2.21 ± 2.43
26	57.19 ± 45.03	6.51 ± 6.74	2.04 ± 2.26	5.76 ± 5.99	2.96 ± 3.19	2.26 ± 2.49
27	55.91 ± 45.84	6.57 ± 6.8	2.07 ± 2.3	5.82 ± 6.06	2.99 ± 3.23	2.32 ± 2.55
28	56.22 ± 45.63	6.64 ± 6.87	2.11 ± 2.34	5.89 ± 6.13	3.04 ± 3.27	2.37 ± 2.6
29	56.14 ± 45.7	6.71 ± 6.94	2.14 ± 2.38	5.95 ± 6.2	3.07 ± 3.32	2.43 ± 2.66
30	55.23 ± 46.4	6.76 ± 6.99	2.17 ± 2.4	6 ± 6.23	3.11 ± 3.34	2.48 ± 2.71
31	54.87 ± 46.34	6.82 ± 7.04	2.2 ± 2.43	6.06 ± 6.29	3.15 ± 3.38	2.52 ± 2.75
32	54.41 ± 46.77	6.87 ± 7.09	2.22 ± 2.44	6.1 ± 6.32	3.18 ± 3.4	2.56 ± 2.79
33	54.47 ± 46.71	6.92 ± 7.14	2.25 ± 2.47	6.16 ± 6.4	3.22 ± 3.46	2.6 ± 2.82
34	54.27 ± 46.91	6.99 ± 7.22	2.29 ± 2.52	6.25 ± 6.49	3.3 ± 3.54	2.64 ± 2.86
35	53.88 ± 47.32	7.04 ± 7.28	2.32 ± 2.56	6.27 ± 6.53	3.31 ± 3.57	2.69 ± 2.93
36	53.62 ± 47.58	7.07 ± 7.31	2.32 ± 2.56	6.29 ± 6.52	3.32 ± 3.55	2.73 ± 2.97
37	53.32 ± 47.88	7.09 ± 7.31	2.32 ± 2.54	6.3 ± 6.51	3.31 ± 3.53	2.75 ± 2.98

The average of the prevalence, the CFU-values per square meter (cfu/sqm) and per gram manure are calculated over all iterations. The CFU values in the intestine as well as the excreted and ingested quantities are obtained by calculating the average over all animals in one iteration at first and then over all iterations. The decadic logarithm of zero or negative values is negative infinite (-Inf).

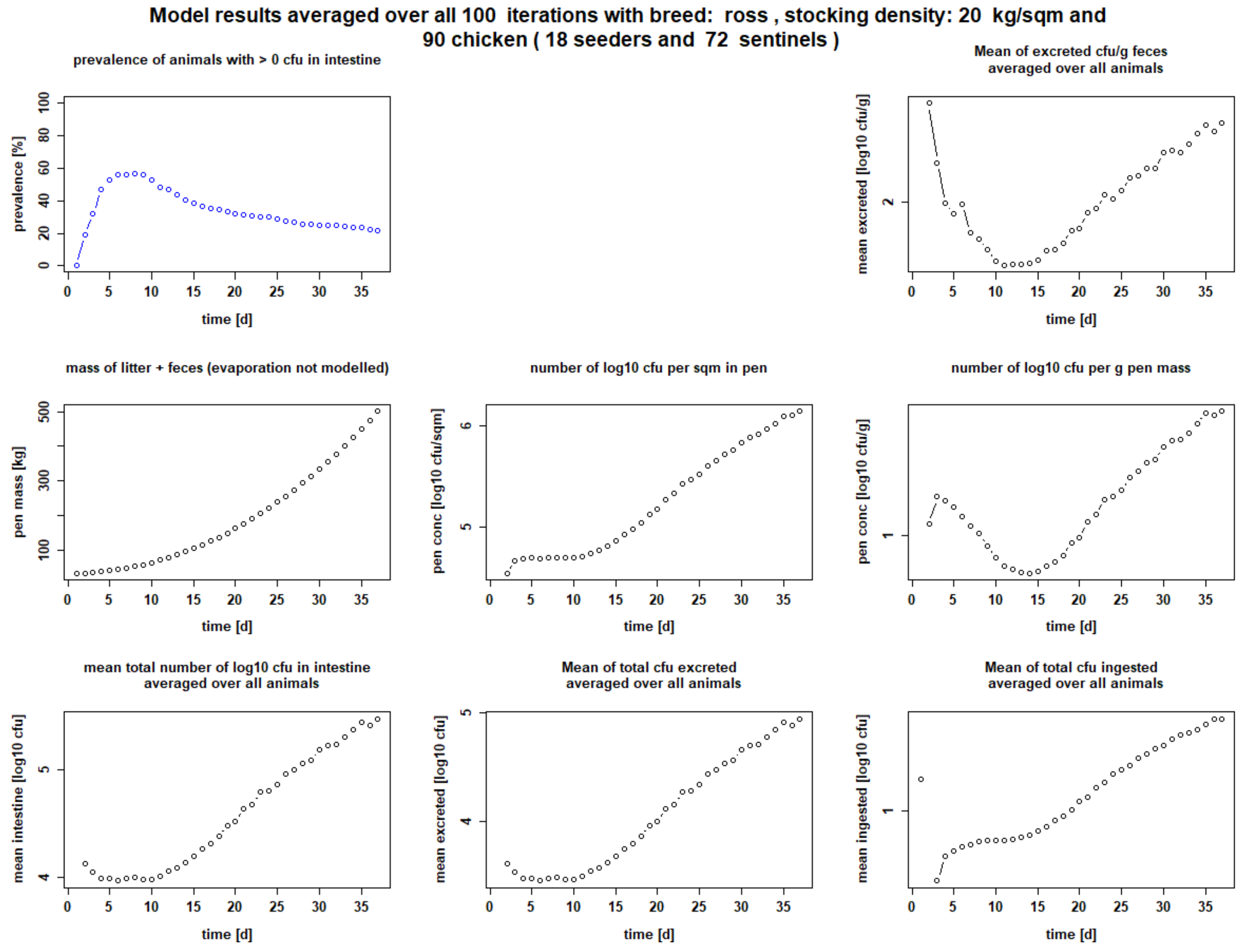

Figure S14. Calculated infection dynamic when day-old chicks are set positive (10^2 CFU bacteria per chick on day 1), combined measures litter 3000 g/m^2 and stocking density 20 kg/m^2. Dotplots with results for 90 chicks, 18 seeder, 72 sentinels, Ross 308, feeding duration 36 days, stocking density 20 kg/m^2, 3000 g litter/m^2

Table S14a (CFU). Calculated numeric values for scenario when day-old chicks are set positive (10^2 CFU bacteria per chick on day 1), combined measures litter 3000 g/m^2 and stocking density 20 kg/m^2. Setting: 90 chicks, 18 seeder, 72 sentinels, Ross 308, feeding duration 36 days, stocking density 20 kg/m^2, 3000 g litter/m^2

day	prevalence in %	cfu/sqm	cfu/g manure	cfu in intestine	cfu/g feces	cfu ingested per animal
	(mean ± sd)	(mean ± sd)	(mean ± sd)	(mean ± sd)	(mean ± sd)	(mean ± sd)
1	0 ± 0	0e0 ± 0	0e0 ± 0	0e0 ± 0	0e0 ± 0	2e1 ± 0e1
2	19.23 ± 0.89	3.5e4 ± 2.1e4	1.1e1 ± 0.6e1	1.4e4 ± 0.8e4	4.1e2 ± 2.4e2	0e0 ± 0
3	31.84 ± 17.64	4.7e4 ± 2.2e4	1.4e1 ± 0.7e1	1.1e4 ± 0.7e4	1.7e2 ± 1e2	2.13 ± 1.5
4	46.89 ± 22.47	4.9e4 ± 2.3e4	1.4e1 ± 0.7e1	9.9e3 ± 5.9e3	9.9e1 ± 5.8e1	3.62 ± 2
5	52.93 ± 24.16	5e4 ± 2.4e4	1.3e1 ± 0.6e1	9.8e3 ± 5.6e3	8.4e1 ± 4.8e1	4.07 ± 2.2
6	56.2 ± 24.71	4.9e4 ± 2.8e4	1.2e1 ± 0.7e1	9.3e3 ± 6.5e3	9.7e1 ± 6.8e1	4.49 ± 2.4
7	56.26 ± 27.06	4.9e4 ± 3.2e4	1.1e1 ± 0.7e1	9.7e3 ± 7.5e3	6.4e1 ± 5e1	4.75 ± 3
8	56.91 ± 28.45	5e4 ± 3.7e4	1e1 ± 0.7e1	10e3 ± 8.4e3	5.9e1 ± 5e1	5.01 ± 3.6
9	55.89 ± 31.21	5e4 ± 4.2e4	9.1 ± 7.6	9.5e3 ± 9.4e3	5e1 ± 5e1	5.15 ± 4.2
10	52.91 ± 32.89	5e4 ± 4.6e4	8.2 ± 7.6	9.6e3 ± 10.3e3	4.3e1 ± 4.6e1	5.22 ± 4.8
11	48.54 ± 34.94	5.1e4 ± 5.5e4	7.7 ± 8.3	1e4 ± 1.3e4	4e1 ± 5.1e1	5.13 ± 5.3
12	47.06 ± 35.37	5.5e4 ± 7.2e4	7.4 ± 9.8	1.1e4 ± 1.8e4	4.1e1 ± 6.3e1	5.26 ± 6.3
13	43.67 ± 36.45	5.9e4 ± 8.8e4	7.2 ± 10.8	1.2e4 ± 2.1e4	4.1e1 ± 6.9e1	5.57 ± 7.9
14	40.46 ± 36.85	6.5e4 ± 10.6e4	7.2 ± 11.7	1.4e4 ± 2.4e4	4.2e1 ± 7.4e1	5.86 ± 9.5
15	38.37 ± 37.01	7.3e4 ± 13.1e4	7.3 ± 13.2	1.6e4 ± 3.1e4	4.4e1 ± 8.6e1	6.45 ± 11.4
16	36.92 ± 36.96	8.4e4 ± 16.2e4	7.7 ± 14.9	1.8e4 ± 3.8e4	5e1 ± 10.3e1	7.07 ± 13.7
17	35.41 ± 37.11	9.5e4 ± 19.8e4	7.9 ± 16.6	2.1e4 ± 4.6e4	5.1e1 ± 11.4e1	7.96 ± 16.5
18	34.57 ± 36.97	1.1e5 ± 2.4e5	8.4 ± 18.7	2.4e4 ± 5.7e4	5.5e1 ± 13.1e1	8.89 ± 19.8
19	33.54 ± 36.85	1.3e5 ± 3.1e5	9.4 ± 21.5	3e4 ± 7.2e4	6.7e1 ± 15.7e1	1.01e1 ± 2.4e1
20	32.36 ± 37.15	1.5e5 ± 3.7e5	9.8 ± 23.8	3.3e4 ± 8.5e4	6.9e1 ± 17.8e1	1.21e1 ± 2.9e1
21	31.47 ± 37.06	1.9e5 ± 4.8e5	1.1e1 ± 2.8e1	4.4e4 ± 11.4e4	8.6e1 ± 22.5e1	1.34e1 ± 3.4e1
22	30.84 ± 37.01	2.2e5 ± 5.7e5	1.2e1 ± 3.1e1	4.8e4 ± 12.9e4	9.1e1 ± 24.4e1	1.65e1 ± 4.3e1
23	30.38 ± 37.23	2.7e5 ± 7.2e5	1.4e1 ± 3.7e1	6.2e4 ± 17.1e4	1.1e2 ± 3.1e2	1.87e1 ± 5e1
24	29.82 ± 36.69	3e5 ± 8e5	1.4e1 ± 3.8e1	6.3e4 ± 17.2e4	1e2 ± 2.8e2	2.25e1 ± 6.2e1
25	29.07 ± 36.02	3.4e5 ± 9e5	1.5e1 ± 4e1	7.4e4 ± 19.7e4	1.2e2 ± 3.2e2	2.42e1 ± 6.6e1
26	27.31 ± 35.82	4e5 ± 10.8e5	1.6e1 ± 4.5e1	9e4 ± 24.9e4	1.4e2 ± 3.9e2	2.69e1 ± 7.3e1
27	26.72 ± 35.69	4.6e5 ± 12.3e5	1.7e1 ± 4.7e1	9.9e4 ± 27.2e4	1.5e2 ± 4e2	3.13e1 ± 8.5e1
28	25.74 ± 36.12	5.2e5 ± 14.4e5	1.9e1 ± 5.1e1	1.2e5 ± 3.3e5	1.6e2 ± 4.6e2	3.47e1 ± 9.4e1
29	25.58 ± 36.24	5.7e5 ± 15.8e5	1.9e1 ± 5.3e1	1.2e5 ± 3.4e5	1.6e2 ± 4.5e2	3.89e1 ± 10.7e1
30	25.12 ± 36.46	6.8e5 ± 18.8e5	2.1e1 ± 5.9e1	1.5e5 ± 4.3e5	2e2 ± 5.6e2	4.12e1 ± 11.4e1
31	24.83 ± 36.56	7.7e5 ± 21.6e5	2.3e1 ± 6.4e1	1.7e5 ± 4.9e5	2.1e2 ± 6.1e2	4.78e1 ± 13.2e1
32	24.72 ± 36.44	8.3e5 ± 22.7e5	2.3e1 ± 6.3e1	1.7e5 ± 4.7e5	2e2 ± 5.6e2	5.26e1 ± 14.7e1
33	24.03 ± 36.78	9.3e5 ± 25.4e5	2.4e1 ± 6.6e1	2e5 ± 5.5e5	2.3e2 ± 6.3e2	5.44e1 ± 15e1
34	23.62 ± 36.47	1.1e6 ± 3e6	2.6e1 ± 7.3e1	2.3e5 ± 6.7e5	2.7e2 ± 7.6e2	5.91e1 ± 16.2e1
35	23.46 ± 36.19	1.2e6 ± 3.5e6	2.9e1 ± 8.2e1	2.8e5 ± 8.1e5	3e2 ± 8.8e2	6.6e1 ± 18.3e1
36	22.48 ± 36.16	1.3e6 ± 3.7e6	2.8e1 ± 8.1e1	2.6e5 ± 7.5e5	2.8e2 ± 8e2	7.47e1 ± 21.3e1
37	21.58 ± 36.05	1.4e6 ± 4e6	2.9e1 ± 8.5e1	3e5 ± 8.7e5	3.1e2 ± 9e2	7.49e1 ± 21.4e1

The average of the prevalence, the CFU-values per square meter (cfu/sqm) and per gram manure are calculated over all iterations. The CFU values in the intestine as well as the excreted and ingested quantities are obtained by calculating the average over all animals in one iteration at first and then over all iterations.

Table S14b (log_{10} CFU). Calculated numeric values for scenario when day-old chicks are set positive (10^2 CFU bacteria per chick on day 1), combined measures litter 3000 g/m^2 and stocking density 20 kg/m^2. Setting: 90 chicks, 18 seeder, 72 sentinels, Ross 308, feeding duration 36 days, stocking density 20 kg/m^2, 3000 g litter/m^2

day	prevalence in %	cfu/sqm	cfu/g manure	cfu in intestine	cfu/g feces	cfu ingested per animal
	(% mean ± sd)	(log10 mean ± sd)	(log10 mean ± sd)	(log10 mean ± sd)	(log10 mean ± sd)	(log10 mean ± sd)
1	0 ± 0	-Inf ± -Inf	-Inf ± -Inf	-Inf ± -Inf	-Inf ± -Inf	1.3 ± 0
2	19.23 ± 0.89	4.54 ± 4.31	1.04 ± 0.81	4.13 ± 3.9	2.61 ± 2.38	-Inf ± 0
3	31.84 ± 17.64	4.67 ± 4.35	1.14 ± 0.82	4.05 ± 3.82	2.24 ± 2.01	0.33 ± 0.19
4	46.89 ± 22.47	4.69 ± 4.37	1.13 ± 0.81	3.99 ± 3.77	1.99 ± 1.77	0.56 ± 0.31
5	52.93 ± 24.16	4.7 ± 4.38	1.11 ± 0.79	3.99 ± 3.75	1.93 ± 1.68	0.61 ± 0.34
6	56.2 ± 24.71	4.69 ± 4.44	1.07 ± 0.82	3.97 ± 3.81	1.99 ± 1.83	0.65 ± 0.38
7	56.26 ± 27.06	4.69 ± 4.51	1.04 ± 0.85	3.99 ± 3.88	1.81 ± 1.7	0.68 ± 0.48
8	56.91 ± 28.45	4.7 ± 4.57	1.01 ± 0.87	4 ± 3.92	1.77 ± 1.7	0.7 ± 0.55
9	55.89 ± 31.21	4.7 ± 4.62	0.96 ± 0.88	3.98 ± 3.97	1.7 ± 1.7	0.71 ± 0.62
10	52.91 ± 32.89	4.7 ± 4.66	0.92 ± 0.88	3.98 ± 4.01	1.64 ± 1.66	0.72 ± 0.68
11	48.54 ± 34.94	4.71 ± 4.74	0.88 ± 0.92	4.01 ± 4.11	1.61 ± 1.71	0.71 ± 0.72
12	47.06 ± 35.37	4.74 ± 4.86	0.87 ± 0.99	4.06 ± 4.25	1.61 ± 1.8	0.72 ± 0.8
13	43.67 ± 36.45	4.77 ± 4.95	0.86 ± 1.03	4.09 ± 4.31	1.61 ± 1.84	0.75 ± 0.9
14	40.46 ± 36.85	4.81 ± 5.02	0.86 ± 1.07	4.14 ± 4.39	1.62 ± 1.87	0.77 ± 0.98
15	38.37 ± 37.01	4.86 ± 5.12	0.87 ± 1.12	4.2 ± 4.49	1.64 ± 1.93	0.81 ± 1.06
16	36.92 ± 36.96	4.92 ± 5.21	0.89 ± 1.17	4.26 ± 4.58	1.7 ± 2.01	0.85 ± 1.14
17	35.41 ± 37.11	4.98 ± 5.3	0.9 ± 1.22	4.31 ± 4.67	1.71 ± 2.06	0.9 ± 1.22
18	34.57 ± 36.97	5.04 ± 5.39	0.92 ± 1.27	4.38 ± 4.76	1.74 ± 2.12	0.95 ± 1.3
19	33.54 ± 36.85	5.12 ± 5.48	0.97 ± 1.33	4.48 ± 4.86	1.82 ± 2.2	1 ± 1.37
20	32.36 ± 37.15	5.18 ± 5.57	0.99 ± 1.38	4.52 ± 4.93	1.84 ± 2.25	1.08 ± 1.46
21	31.47 ± 37.06	5.27 ± 5.68	1.05 ± 1.45	4.64 ± 5.06	1.93 ± 2.35	1.13 ± 1.53
22	30.84 ± 37.01	5.34 ± 5.75	1.08 ± 1.5	4.68 ± 5.11	1.96 ± 2.39	1.22 ± 1.63
23	30.38 ± 37.23	5.43 ± 5.86	1.14 ± 1.57	4.79 ± 5.23	2.04 ± 2.49	1.27 ± 1.7
24	29.82 ± 36.69	5.47 ± 5.9	1.15 ± 1.58	4.8 ± 5.23	2.02 ± 2.45	1.35 ± 1.79
25	29.07 ± 36.02	5.53 ± 5.95	1.17 ± 1.6	4.87 ± 5.29	2.07 ± 2.5	1.38 ± 1.82
26	27.31 ± 35.82	5.6 ± 6.04	1.22 ± 1.65	4.96 ± 5.4	2.15 ± 2.59	1.43 ± 1.86
27	26.72 ± 35.69	5.66 ± 6.09	1.24 ± 1.67	5 ± 5.44	2.17 ± 2.6	1.5 ± 1.93
28	25.74 ± 36.12	5.72 ± 6.16	1.27 ± 1.71	5.06 ± 5.51	2.21 ± 2.66	1.54 ± 1.97
29	25.58 ± 36.24	5.76 ± 6.2	1.28 ± 1.72	5.08 ± 5.53	2.21 ± 2.65	1.59 ± 2.03
30	25.12 ± 36.46	5.83 ± 6.27	1.33 ± 1.77	5.19 ± 5.63	2.3 ± 2.75	1.61 ± 2.06
31	24.83 ± 36.56	5.89 ± 6.34	1.36 ± 1.8	5.23 ± 5.69	2.32 ± 2.78	1.68 ± 2.12
32	24.72 ± 36.44	5.92 ± 6.36	1.36 ± 1.8	5.23 ± 5.67	2.31 ± 2.75	1.72 ± 2.17
33	24.03 ± 36.78	5.97 ± 6.4	1.38 ± 1.82	5.3 ± 5.74	2.36 ± 2.8	1.74 ± 2.18
34	23.62 ± 36.47	6.03 ± 6.47	1.42 ± 1.86	5.37 ± 5.83	2.42 ± 2.88	1.77 ± 2.21
35	23.46 ± 36.19	6.09 ± 6.55	1.46 ± 1.92	5.44 ± 5.91	2.48 ± 2.94	1.82 ± 2.26
36	22.48 ± 36.16	6.11 ± 6.56	1.45 ± 1.91	5.41 ± 5.87	2.44 ± 2.9	1.87 ± 2.33
37	21.58 ± 36.05	6.15 ± 6.61	1.47 ± 1.93	5.47 ± 5.94	2.49 ± 2.95	1.87 ± 2.33

The average of the prevalence, the CFU-values per square meter (cfu/sqm) and per gram manure are calculated over all iterations. The CFU values in the intestine as well as the excreted and ingested quantities are obtained by calculating the average over all animals in one iteration at first and then over all iterations. The decadic logarithm of zero or negative values is negative infinite (-Inf).

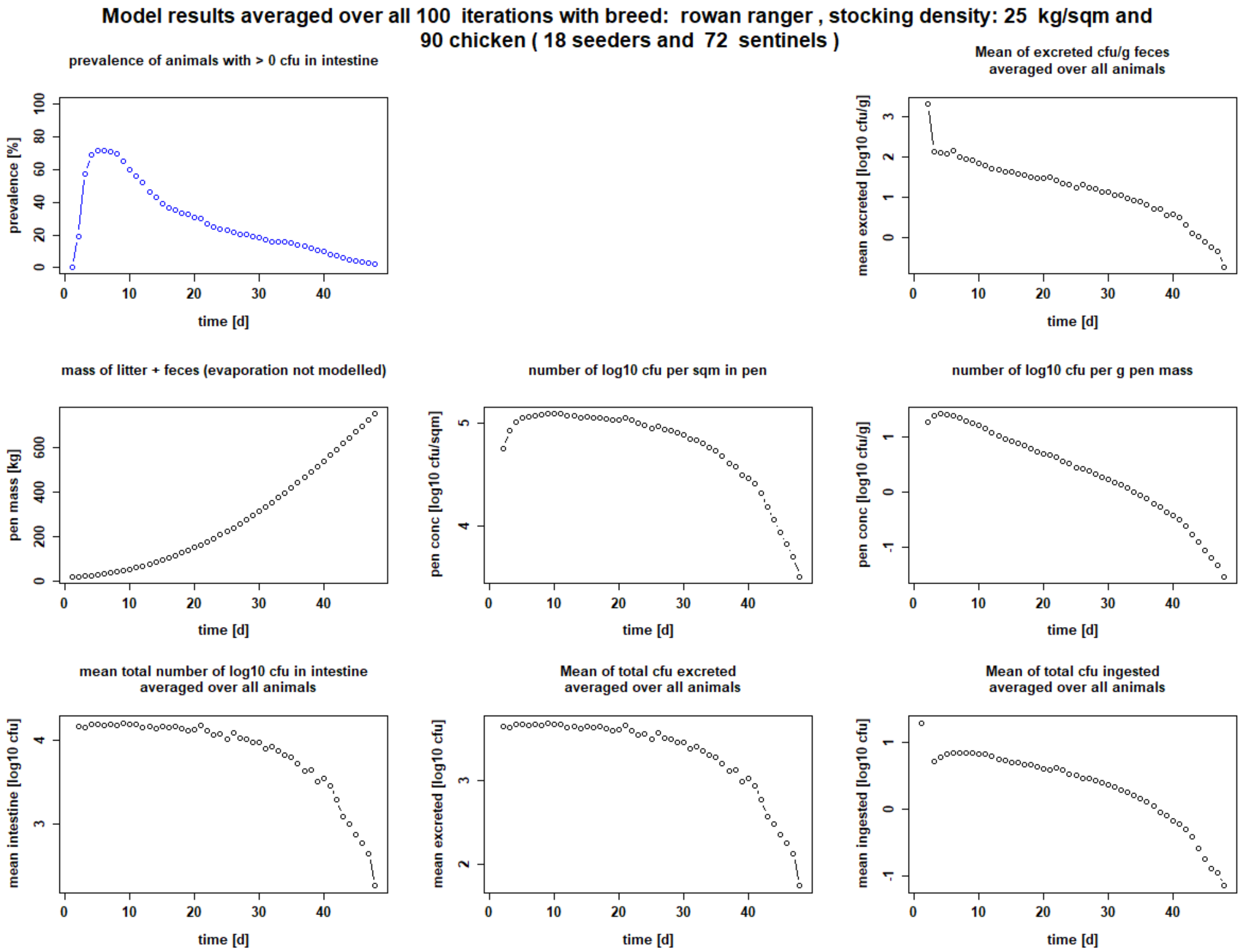

Figure S15. Calculated infection dynamic when day-old chicks are set positive (10^2 CFU bacteria per chick on day 1), combined measures slow growing breed, stocking density 25 kg/m^2 and litter 3000 g/m^2. Dotplots with results for 90 chicks, 18 seeder, 72 sentinels, Rowan x Ranger, feeding duration 47 days, stocking density 25 kg/m^2, 3000 g litter/m^2

Table S15a (CFU). Calculated numeric values for scenario when day-old chicks are set positive (10^2 CFU bacteria per chick on day 1), combined measures slow growing breed, stocking density 25 kg/m^2 and litter 3000 g/m^2 Setting: 90 chicks, 18 seeder, 72 sentinels, Rowan x Ranger, feeding duration 47 days, stocking density 25 kg/m^2, 3000 g litter/m^2

day	prevalence in %	cfu/sqm	cfu/g manure	cfu in intestine	cfu/g feces	cfu ingested per animal
	(mean ± sd)	(mean ± sd)	(mean ± sd)	(mean ± sd)	(mean ± sd)	(mean ± sd)
1	0 ± 0	0e0 ± 0	0e0 ± 0	0e0 ± 0	0e0 ± 0	2e1 ± 0e1
2	19.22 ± 0.91	5.7e4 ± 3.4e4	1.8e1 ± 1.1e1	1.5e4 ± 0.9e4	2e3 ± 1.2e3	0e0 ± 0
3	57.31 ± 24.28	8.5e4 ± 4.2e4	2.4e1 ± 1.2e1	1.4e4 ± 0.8e4	1.4e2 ± 0.7e2	5.33 ± 3.5
4	69.37 ± 23.78	1e5 ± 0.6e5	2.6e1 ± 1.4e1	1.6e4 ± 1e4	1.3e2 ± 0.8e2	6.13 ± 3.3
5	71.79 ± 25.67	1.1e5 ± 0.7e5	2.5e1 ± 1.4e1	1.6e4 ± 1e4	1.2e2 ± 0.8e2	6.72 ± 3.9
6	72.03 ± 28	1.2e5 ± 0.7e5	2.3e1 ± 1.4e1	1.5e4 ± 1e4	1.4e2 ± 0.9e2	7.13 ± 4.5
7	71.16 ± 28.75	1.2e5 ± 0.8e5	2.1e1 ± 1.5e1	1.6e4 ± 1.3e4	10e1 ± 8.1e1	7.01 ± 4.5
8	69.78 ± 29.85	1.2e5 ± 0.9e5	1.9e1 ± 1.5e1	1.5e4 ± 1.3e4	8.7e1 ± 7.7e1	7.04 ± 5.1
9	65.42 ± 33.05	1.2e5 ± 1.1e5	1.8e1 ± 1.6e1	1.6e4 ± 1.7e4	8.3e1 ± 8.9e1	6.93 ± 5.7
10	60.22 ± 35.22	1.2e5 ± 1.3e5	1.6e1 ± 1.6e1	1.6e4 ± 1.9e4	7.1e1 ± 8.4e1	6.89 ± 6.6
11	56.13 ± 36.6	1.2e5 ± 1.4e5	1.4e1 ± 1.6e1	1.6e4 ± 2e4	6.1e1 ± 7.8e1	6.73 ± 7.4
12	52.08 ± 37.69	1.2e5 ± 1.4e5	1.2e1 ± 1.4e1	1.4e4 ± 1.9e4	5.2e1 ± 6.9e1	6.32 ± 7.7
13	46.63 ± 37.58	1.2e5 ± 1.6e5	1e1 ± 1.4e1	1.5e4 ± 2.2e4	5e1 ± 7.4e1	5.66 ± 7.6
14	43 ± 37.43	1.1e5 ± 1.7e5	9 ± 13.4	1.4e4 ± 2.3e4	4.2e1 ± 7.1e1	5.53 ± 8.1
15	39.04 ± 37.98	1.1e5 ± 1.9e5	8.3 ± 13.6	1.5e4 ± 2.7e4	4.2e1 ± 7.7e1	5.01 ± 8.3
16	36.87 ± 37.51	1.1e5 ± 2e5	7.4 ± 13	1.4e4 ± 2.7e4	3.9e1 ± 7.4e1	5.01 ± 9.1
17	35.37 ± 37.09	1.1e5 ± 2.1e5	6.7 ± 12.5	1.5e4 ± 2.9e4	3.7e1 ± 7.2e1	4.78 ± 9.3
18	33.43 ± 36.71	1.1e5 ± 2.2e5	6 ± 11.7	1.4e4 ± 2.9e4	3.2e1 ± 6.8e1	4.64 ± 9.5
19	32.44 ± 36.16	1.1e5 ± 2.2e5	5.3 ± 10.9	1.3e4 ± 2.9e4	3e1 ± 6.5e1	4.4 ± 9.5
20	31.02 ± 35.74	1.1e5 ± 2.3e5	4.8 ± 10.4	1.4e4 ± 3.1e4	3e1 ± 6.7e1	4.12 ± 9.3
21	29.89 ± 34.02	1.1e5 ± 2.6e5	4.7 ± 10.9	1.5e4 ± 3.8e4	3.1e1 ± 7.9e1	3.97 ± 9.3
22	26.92 ± 33.57	1.1e5 ± 2.6e5	4.2 ± 9.8	1.3e4 ± 3.3e4	2.6e1 ± 6.5e1	4.14 ± 10.4
23	24.68 ± 32.52	10e4 ± 24.7e4	3.6 ± 8.8	1.2e4 ± 3.1e4	2.2e1 ± 5.9e1	3.84 ± 9.7
24	23.34 ± 31.78	9.7e4 ± 25e4	3.2 ± 8.3	1.2e4 ± 3.3e4	2.1e1 ± 5.8e1	3.42 ± 9.2
25	22.94 ± 31	8.9e4 ± 23.2e4	2.7 ± 7.1	1e4 ± 2.8e4	1.8e1 ± 4.7e1	3.24 ± 9
26	21.8 ± 31.14	9.2e4 ± 25.5e4	2.7 ± 7.3	1.2e4 ± 3.6e4	2.1e1 ± 6.1e1	2.88 ± 8.1
27	20.5 ± 30.63	8.8e4 ± 24.5e4	2.4 ± 6.6	1.1e4 ± 3.1e4	1.7e1 ± 4.9e1	2.93 ± 8.6
28	20.1 ± 30.34	8.5e4 ± 24.1e4	2.1 ± 6	1e4 ± 3e4	1.6e1 ± 4.6e1	2.74 ± 8.1
29	19.1 ± 29.84	8e4 ± 22.9e4	1.9 ± 5.4	9.7e3 ± 28e3	1.4e1 ± 4e1	2.53 ± 7.7
30	18.19 ± 29.07	7.8e4 ± 23e4	1.7 ± 5.1	9.6e3 ± 30e3	1.4e1 ± 4.3e1	2.32 ± 7.1
31	17.28 ± 28.38	7.1e4 ± 21.2e4	1.5 ± 4.4	8.1e3 ± 24.9e3	1.1e1 ± 3.4e1	2.19 ± 6.9
32	16.02 ± 28.09	6.8e4 ± 20.8e4	1.3 ± 4.1	8.4e3 ± 27.1e3	1.1e1 ± 3.5e1	1.93 ± 6.2
33	15.76 ± 28.05	6.4e4 ± 20.1e4	1.2 ± 3.7	7.6e3 ± 25.1e3	9.6 ± 31.9	1.81 ± 5.9
34	15.53 ± 28.03	5.9e4 ± 18.7e4	1 ± 3.2	6.8e3 ± 22.3e3	8.5 ± 28	1.63 ± 5.5
35	14.98 ± 27.33	5.4e4 ± 17.9e4	8.9e-1 ± 29.4e-1	6.4e3 ± 21.9e3	7.7 ± 26.6	1.44 ± 4.9
36	13.58 ± 26.46	4.8e4 ± 16.3e4	7.5e-1 ± 25.5e-1	5.4e3 ± 18.9e3	6.4 ± 22.5	1.3 ± 4.6
37	13.17 ± 25.79	4.1e4 ± 14.4e4	6.1e-1 ± 21.4e-1	4.4e3 ± 16.3e3	5.1 ± 19	1.12 ± 4.1
38	11.8 ± 25.25	3.8e4 ± 14.2e4	5.4e-1 ± 20e-1	4.5e3 ± 18.1e3	5 ± 20.3	9.1e-1 ± 35e-1
39	10.51 ± 23.9	3.2e4 ± 11.8e4	4.3e-1 ± 15.9e-1	3.2e3 ± 12.5e3	3.5 ± 13.7	8.1e-1 ± 33.6e-1
40	9.8 ± 22.64	3e4 ± 12.4e4	3.8e-1 ± 15.8e-1	3.5e3 ± 17e3	3.8 ± 18.4	6.6e-1 ± 27.3e-1
41	8.24 ± 20.64	2.6e4 ± 11.6e4	3.2e-1 ± 14.2e-1	2.9e3 ± 14e3	3.1 ± 14.8	5.9e-1 ± 27.3e-1
42	7.41 ± 18.9	2.1e4 ± 9.7e4	2.4e-1 ± 11.4e-1	2e3 ± 10.2e3	2 ± 10.5	5e-1 ± 25.4e-1
43	6.08 ± 16.92	1.5e4 ± 7.3e4	1.7e-1 ± 8.1e-1	1.2e3 ± 6.2e3	1.3 ± 6.4	3.8e-1 ± 20.1e-1
44	4.88 ± 16.13	1.2e4 ± 5.9e4	1.2e-1 ± 6.3e-1	1e3 ± 6.1e3	1 ± 6.4	2.6e-1 ± 14.6e-1
45	4.14 ± 14.72	8.8e3 ± 46.4e3	9e-2 ± 47.8e-2	7.6e2 ± 43.8e2	7.7e-1 ± 44.7e-1	1.8e-1 ± 11.1e-1
46	3.72 ± 13.52	6.7e3 ± 37.4e3	6.6e-2 ± 37e-2	5.9e2 ± 36.8e2	5.8e-1 ± 36.6e-1	1.3e-1 ± 8.6e-1
47	2.84 ± 12.39	5.1e3 ± 30.2e3	4.8e-2 ± 28.8e-2	4.5e2 ± 30.4e2	4.5e-1 ± 30.5e-1	1.1e-1 ± 7.2e-1
48	1.83 ± 8.97	3.3e3 ± 19.9e3	3e-2 ± 18.3e-2	1.8e2 ± 13.3e2	1.8e-1 ± 13.1e-1	7e-2 ± 53.7e-2

The average of the prevalence, the CFU-values per square meter (cfu/sqm) and per gram manure are calculated over all iterations. The CFU values in the intestine as well as the excreted and ingested quantities are obtained by calculating the average over all animals in one iteration at first and then over all iterations.

Table S15b (log_{10} CFU). Calculated numeric values for scenario when day-old chicks are set positive (10^2 CFU bacteria per chick on day 1), combined measures slow growing breed, stocking density 25 kg/m^2 and litter 3000 g/m^2 Setting: 90 chicks, 18 seeder, 72 sentinels, Rowan x Ranger, feeding duration 47 days, stocking density 25 kg/m^2, 3000 g litter/m^2

day	prevalence in %	cfu/sqm	cfu/g manure	cfu in intestine	cfu/g feces	cfu ingested per animal
	(% mean ± sd)	(log10 mean ± sd)	(log10 mean ± sd)	(log10 mean ± sd)	(log10 mean ± sd)	(log10 mean ± sd)
1	0 ± 0	-Inf ± -Inf	-Inf ± -Inf	-Inf ± -Inf	-Inf ± -Inf	1.3 ± 0
2	19.22 ± 0.91	4.76 ± 4.53	1.26 ± 1.03	4.16 ± 3.93	3.3 ± 3.07	-Inf ± 0
3	57.31 ± 24.28	4.93 ± 4.62	1.38 ± 1.07	4.16 ± 3.88	2.14 ± 1.86	0.73 ± 0.54
4	69.37 ± 23.78	5.02 ± 4.74	1.41 ± 1.14	4.19 ± 3.98	2.11 ± 1.9	0.79 ± 0.52
5	71.79 ± 25.67	5.05 ± 4.81	1.4 ± 1.16	4.19 ± 4.01	2.08 ± 1.9	0.83 ± 0.59
6	72.03 ± 28	5.07 ± 4.84	1.37 ± 1.14	4.18 ± 4	2.15 ± 1.96	0.85 ± 0.65
7	71.16 ± 28.75	5.08 ± 4.91	1.33 ± 1.17	4.2 ± 4.11	2 ± 1.91	0.85 ± 0.65
8	69.78 ± 29.85	5.08 ± 4.96	1.28 ± 1.16	4.19 ± 4.13	1.94 ± 1.88	0.85 ± 0.71
9	65.42 ± 33.05	5.09 ± 5.05	1.24 ± 1.2	4.21 ± 4.24	1.92 ± 1.95	0.84 ± 0.76
10	60.22 ± 35.22	5.09 ± 5.1	1.19 ± 1.2	4.2 ± 4.27	1.85 ± 1.92	0.84 ± 0.82
11	56.13 ± 36.6	5.09 ± 5.15	1.14 ± 1.2	4.2 ± 4.3	1.79 ± 1.89	0.83 ± 0.87
12	52.08 ± 37.69	5.07 ± 5.16	1.07 ± 1.15	4.15 ± 4.28	1.71 ± 1.84	0.8 ± 0.88
13	46.63 ± 37.58	5.07 ± 5.19	1.02 ± 1.15	4.17 ± 4.35	1.69 ± 1.87	0.75 ± 0.88
14	43 ± 37.43	5.05 ± 5.22	0.96 ± 1.13	4.14 ± 4.36	1.63 ± 1.85	0.74 ± 0.91
15	39.04 ± 37.98	5.06 ± 5.28	0.92 ± 1.13	4.17 ± 4.43	1.63 ± 1.89	0.7 ± 0.92
16	36.87 ± 37.51	5.05 ± 5.3	0.87 ± 1.11	4.15 ± 4.43	1.59 ± 1.87	0.7 ± 0.96
17	35.37 ± 37.09	5.06 ± 5.32	0.83 ± 1.1	4.16 ± 4.46	1.56 ± 1.86	0.68 ± 0.97
18	33.43 ± 36.71	5.05 ± 5.33	0.78 ± 1.07	4.14 ± 4.46	1.51 ± 1.83	0.67 ± 0.98
19	32.44 ± 36.16	5.03 ± 5.34	0.72 ± 1.04	4.12 ± 4.46	1.47 ± 1.81	0.64 ± 0.98
20	31.02 ± 35.74	5.03 ± 5.36	0.68 ± 1.02	4.13 ± 4.49	1.47 ± 1.82	0.61 ± 0.97
21	29.89 ± 34.02	5.05 ± 5.42	0.67 ± 1.04	4.18 ± 4.58	1.5 ± 1.9	0.6 ± 0.97
22	26.92 ± 33.57	5.04 ± 5.41	0.62 ± 0.99	4.12 ± 4.51	1.42 ± 1.81	0.62 ± 1.02
23	24.68 ± 32.52	5 ± 5.39	0.55 ± 0.95	4.07 ± 4.49	1.34 ± 1.77	0.58 ± 0.99
24	23.34 ± 31.78	4.99 ± 5.4	0.5 ± 0.92	4.08 ± 4.52	1.32 ± 1.76	0.53 ± 0.96
25	22.94 ± 31	4.95 ± 5.37	0.43 ± 0.85	4.01 ± 4.44	1.24 ± 1.67	0.51 ± 0.95
26	21.8 ± 31.14	4.97 ± 5.41	0.42 ± 0.86	4.09 ± 4.56	1.32 ± 1.79	0.46 ± 0.91
27	20.5 ± 30.63	4.94 ± 5.39	0.37 ± 0.82	4.03 ± 4.49	1.23 ± 1.69	0.47 ± 0.94
28	20.1 ± 30.34	4.93 ± 5.38	0.33 ± 0.78	4.02 ± 4.48	1.2 ± 1.66	0.44 ± 0.91
29	19.1 ± 29.84	4.9 ± 5.36	0.27 ± 0.73	3.98 ± 4.45	1.14 ± 1.61	0.4 ± 0.88
30	18.19 ± 29.07	4.89 ± 5.36	0.23 ± 0.7	3.98 ± 4.48	1.13 ± 1.63	0.37 ± 0.85
31	17.28 ± 28.38	4.85 ± 5.33	0.17 ± 0.64	3.91 ± 4.4	1.04 ± 1.53	0.34 ± 0.84
32	16.02 ± 28.09	4.83 ± 5.32	0.12 ± 0.61	3.92 ± 4.43	1.04 ± 1.55	0.29 ± 0.79
33	15.76 ± 28.05	4.81 ± 5.3	0.07 ± 0.57	3.88 ± 4.4	0.98 ± 1.5	0.26 ± 0.77
34	15.53 ± 28.03	4.77 ± 5.27	0.01 ± 0.51	3.83 ± 4.35	0.93 ± 1.45	0.21 ± 0.74
35	14.98 ± 27.33	4.73 ± 5.25	-0.05 ± 0.47	3.8 ± 4.34	0.89 ± 1.42	0.16 ± 0.69
36	13.58 ± 26.46	4.68 ± 5.21	-0.12 ± 0.41	3.73 ± 4.28	0.8 ± 1.35	0.11 ± 0.66
37	13.17 ± 25.79	4.61 ± 5.16	-0.21 ± 0.33	3.64 ± 4.21	0.7 ± 1.28	0.05 ± 0.61
38	11.8 ± 25.25	4.58 ± 5.15	-0.27 ± 0.3	3.65 ± 4.26	0.7 ± 1.31	-0.04 ± 0.54
39	10.51 ± 23.9	4.5 ± 5.07	-0.37 ± 0.2	3.51 ± 4.1	0.55 ± 1.14	-0.09 ± 0.53
40	9.8 ± 22.64	4.47 ± 5.09	-0.42 ± 0.2	3.54 ± 4.23	0.58 ± 1.26	-0.18 ± 0.44
41	8.24 ± 20.64	4.42 ± 5.07	-0.49 ± 0.15	3.46 ± 4.15	0.49 ± 1.17	-0.23 ± 0.44
42	7.41 ± 18.9	4.32 ± 4.99	-0.61 ± 0.06	3.29 ± 4.01	0.31 ± 1.02	-0.3 ± 0.4
43	6.08 ± 16.92	4.18 ± 4.86	-0.77 ± -0.09	3.09 ± 3.79	0.11 ± 0.81	-0.42 ± 0.3
44	4.88 ± 16.13	4.06 ± 4.77	-0.91 ± -0.2	3 ± 3.79	0.02 ± 0.8	-0.59 ± 0.16
45	4.14 ± 14.72	3.94 ± 4.67	-1.04 ± -0.32	2.88 ± 3.64	-0.11 ± 0.65	-0.74 ± 0.05
46	3.72 ± 13.52	3.83 ± 4.57	-1.18 ± -0.43	2.77 ± 3.57	-0.24 ± 0.56	-0.89 ± -0.07
47	2.84 ± 12.39	3.71 ± 4.48	-1.31 ± -0.54	2.65 ± 3.48	-0.35 ± 0.48	-0.96 ± -0.14
48	1.83 ± 8.97	3.51 ± 4.3	-1.52 ± -0.74	2.26 ± 3.12	-0.75 ± 0.12	-1.15 ± -0.27

The average of the prevalence, the CFU-values per square meter (cfu/sqm) and per gram manure are calculated over all iterations. The CFU values in the intestine as well as the excreted and ingested quantities are obtained by calculating the average over all animals in one iteration at first and then over all iterations. The decadic logarithm of zero or negative values is negative infinite (-Inf).

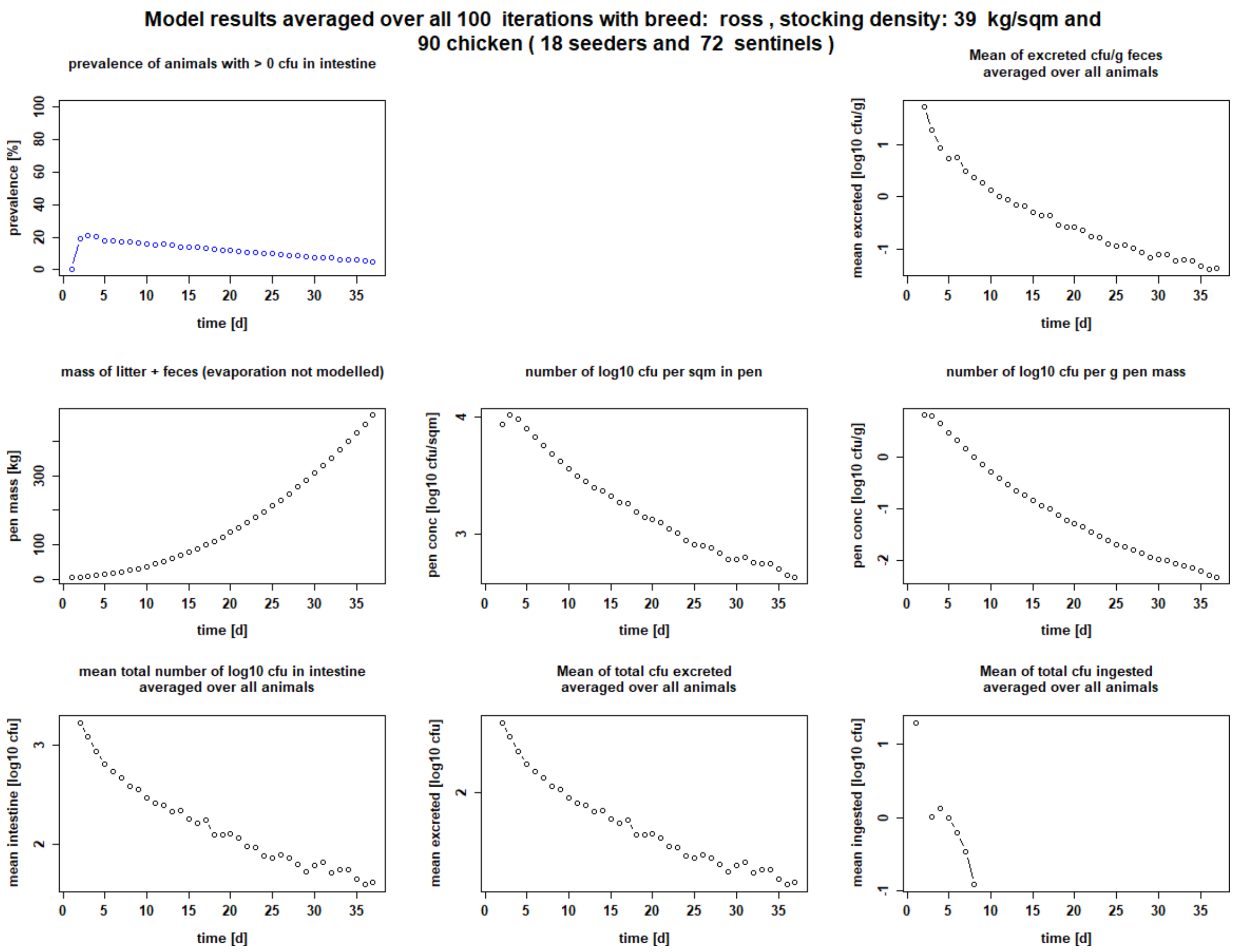

Figure S16. Calculated infection dynamic when day-old chicks are set positive (10^2 CFU bacteria per chick on day 1), measure lower growth rate for ESBLs in the intestines. Dotplots with results for 90 chicks, 18 seeder, 72 sentinels, Ross 308, feeding duration 36 days, stocking density 39 kg/m^2, 1000 g litter/m^2

Table S16a (CFU). Calculated numeric values for scenario when day-old chicks are set positive (10^2 CFU bacteria per chick on day 1), measure lower growth rate for ESBLs in the intestines. Setting: 90 chicks, 18 seeder, 72 sentinels, Ross 308, feeding duration 36 days, stocking density 39 kg/m^2, 1000 g litter/m^2

day	prevalence in %	cfu/sqm	cfu/g manure	cfu in intestine	cfu/g feces	cfu ingested per animal
	(mean ± sd)	(mean ± sd)	(mean ± sd)	(mean ± sd)	(mean ± sd)	(mean ± sd)
1	0 ± 0	0e0 ± 0	0e0 ± 0	0e0 ± 0	0e0 ± 0	2e1 ± 0e1
2	19.22 ± 0.9	8.5e3 ± 4.8e3	6.4 ± 3.6	1.7e3 ± 1e3	5.1e1 ± 2.9e1	0e0 ± 0
3	20.68 ± 6.97	1e4 ± 0.4e4	6.2 ± 2.2	1.2e3 ± 0.5e3	1.9e1 ± 0.8e1	1.02 ± 0.9
4	20.02 ± 6.37	9.5e3 ± 3.3e3	4.4 ± 1.5	8.7e2 ± 4.8e2	8.7 ± 4.8	1.32 ± 0.7
5	18 ± 3.07	8e3 ± 2.3e3	2.9 ± 0.8	6.4e2 ± 3.1e2	5.5 ± 2.7	1 ± 0.6
6	17.46 ± 1.52	6.7e3 ± 1.9e3	2.1 ± 0.6	5.5e2 ± 3.2e2	5.7 ± 3.4	6.2e-1 ± 5.1e-1
7	16.98 ± 1.48	5.7e3 ± 1.7e3	1.4 ± 0.4	4.7e2 ± 2.8e2	3.1 ± 1.8	3.4e-1 ± 4.8e-1
8	16.84 ± 1.7	4.8e3 ± 1.4e3	10e-1 ± 2.8e-1	3.9e2 ± 2.2e2	2.3 ± 1.3	1.2e-1 ± 3.3e-1
9	16.49 ± 1.96	4.2e3 ± 1.2e3	7.3e-1 ± 2.1e-1	3.6e2 ± 2.1e2	1.9 ± 1.1	0e0 ± 0
10	15.8 ± 2.05	3.6e3 ± 1.1e3	5.2e-1 ± 1.6e-1	3e2 ± 1.8e2	1.3 ± 0.8	0e0 ± 0
11	15.13 ± 2.09	3.1e3 ± 1.2e3	3.8e-1 ± 1.4e-1	2.6e2 ± 1.8e2	1 ± 0.7	0e0 ± 0
12	15.53 ± 2.09	2.8e3 ± 1e3	2.9e-1 ± 1e-1	2.5e2 ± 1.6e2	8.9e-1 ± 5.8e-1	0e0 ± 0
13	14.83 ± 2.16	2.5e3 ± 0.9e3	2.2e-1 ± 0.8e-1	2.2e2 ± 1.3e2	7.2e-1 ± 4.3e-1	0e0 ± 0
14	14.14 ± 2.2	2.4e3 ± 0.9e3	1.8e-1 ± 0.7e-1	2.2e2 ± 1.4e2	6.7e-1 ± 4.3e-1	0e0 ± 0
15	13.57 ± 2.62	2.1e3 ± 0.8e3	1.4e-1 ± 0.5e-1	1.8e2 ± 1.2e2	5.1e-1 ± 3.3e-1	0e0 ± 0
16	13.71 ± 2.1	1.9e3 ± 0.6e3	1.1e-1 ± 0.4e-1	1.6e2 ± 1e2	4.4e-1 ± 2.7e-1	0e0 ± 0
17	12.88 ± 2.3	1.8e3 ± 0.6e3	9.9e-2 ± 3.3e-2	1.8e2 ± 1e2	4.4e-1 ± 2.5e-1	0e0 ± 0
18	12.36 ± 2.45	1.5e3 ± 0.5e3	7.5e-2 ± 2.5e-2	1.3e2 ± 0.8e2	2.9e-1 ± 1.9e-1	0e0 ± 0
19	12.11 ± 2.45	1.4e3 ± 0.4e3	6.1e-2 ± 2e-2	1.2e2 ± 0.7e2	2.7e-1 ± 1.6e-1	0e0 ± 0
20	11.57 ± 2.34	1.3e3 ± 0.5e3	5.3e-2 ± 1.9e-2	1.3e2 ± 0.9e2	2.7e-1 ± 1.8e-1	0e0 ± 0
21	11.14 ± 2.36	1.3e3 ± 0.4e3	4.5e-2 ± 1.6e-2	1.2e2 ± 0.7e2	2.3e-1 ± 1.4e-1	0e0 ± 0
22	10.57 ± 2.31	1.1e3 ± 0.4e3	3.6e-2 ± 1.4e-2	9.4e1 ± 7.7e1	1.8e-1 ± 1.5e-1	0e0 ± 0
23	10.39 ± 2.2	1e3 ± 0.4e3	3.1e-2 ± 1.2e-2	9.2e1 ± 6.4e1	1.7e-1 ± 1.2e-1	0e0 ± 0
24	9.7 ± 2.31	8.9e2 ± 3e2	2.5e-2 ± 0.8e-2	7.7e1 ± 4.7e1	1.3e-1 ± 0.8e-1	0e0 ± 0
25	9.62 ± 2.45	8.1e2 ± 2.9e2	2.1e-2 ± 0.7e-2	7.3e1 ± 5e1	1.2e-1 ± 0.8e-1	0e0 ± 0
26	9.1 ± 2.49	8e2 ± 2.9e2	1.9e-2 ± 0.7e-2	7.7e1 ± 5.3e1	1.2e-1 ± 0.8e-1	0e0 ± 0
27	8.81 ± 2.36	7.6e2 ± 3e2	1.7e-2 ± 0.7e-2	7.3e1 ± 5e1	1.1e-1 ± 0.7e-1	0e0 ± 0
28	8.58 ± 2.55	6.9e2 ± 2.8e2	1.4e-2 ± 0.6e-2	6.2e1 ± 5.1e1	8.7e-2 ± 7.2e-2	0e0 ± 0
29	7.68 ± 2.23	6.1e2 ± 2.5e2	1.1e-2 ± 0.5e-2	5.3e1 ± 4.2e1	7e-2 ± 5.5e-2	0e0 ± 0
30	7.59 ± 2.26	6.1e2 ± 2.9e2	1.1e-2 ± 0.5e-2	6.1e1 ± 4.9e1	7.9e-2 ± 6.4e-2	0e0 ± 0
31	7.53 ± 2.13	6.3e2 ± 3e2	1e-2 ± 0.5e-2	6.5e1 ± 5.1e1	8.1e-2 ± 6.3e-2	0e0 ± 0
32	7.12 ± 2.16	5.7e2 ± 2.8e2	8.7e-3 ± 4.3e-3	5.1e1 ± 4.8e1	6.1e-2 ± 5.7e-2	0e0 ± 0
33	6.22 ± 2.51	5.6e2 ± 2.9e2	8.1e-3 ± 4.2e-3	5.5e1 ± 4.8e1	6.3e-2 ± 5.5e-2	0e0 ± 0
34	6.26 ± 2.17	5.6e2 ± 2.9e2	7.5e-3 ± 4e-3	5.5e1 ± 4.8e1	6.2e-2 ± 5.5e-2	0e0 ± 0
35	6.19 ± 2.15	5e2 ± 2.6e2	6.4e-3 ± 3.3e-3	4.5e1 ± 4.1e1	4.8e-2 ± 4.5e-2	0e0 ± 0
36	5.36 ± 2.06	4.5e2 ± 2.2e2	5.3e-3 ± 2.7e-3	3.9e1 ± 3.6e1	4.1e-2 ± 3.8e-2	0e0 ± 0
37	4.63 ± 2.22	4.3e2 ± 2.4e2	4.9e-3 ± 2.7e-3	4.1e1 ± 4.3e1	4.3e-2 ± 4.4e-2	0e0 ± 0

The average of the prevalence, the CFU-values per square meter (cfu/sqm) and per gram manure are calculated over all iterations. The CFU values in the intestine as well as the excreted and ingested quantities are obtained by calculating the average over all animals in one iteration at first and then over all iterations.

Table S16b (log_{10} CFU). Calculated numeric values for scenario when day-old chicks are set positive (10^2 CFU bacteria per chick on day 1), measure lower growth rate for ESBLs in the intestines. Setting: 90 chicks, 18 seeder, 72 sentinels, Ross 308, feeding duration 36 days, stocking density 39 kg/m², 1000 g litter/m²

day	prevalence in %	cfu/sqm	cfu/g manure	cfu in intestine	cfu/g feces	cfu ingested per animal
	(% mean ± sd)	(log10 mean ± sd)	(log10 mean ± sd)	(log10 mean ± sd)	(log10 mean ± sd)	(log10 mean ± sd)
1	0 ± 0	-Inf ± -Inf	-Inf ± -Inf	-Inf ± -Inf	-Inf ± -Inf	1.3 ± 0
2	19.22 ± 0.9	3.93 ± 3.68	0.81 ± 0.56	3.23 ± 2.98	1.71 ± 1.46	-Inf ± 0
3	20.68 ± 6.97	4.02 ± 3.57	0.8 ± 0.35	3.08 ± 2.71	1.27 ± 0.9	0.01 ± -0.05
4	20.02 ± 6.37	3.98 ± 3.52	0.64 ± 0.19	2.94 ± 2.68	0.94 ± 0.68	0.12 ± -0.16
5	18 ± 3.07	3.9 ± 3.35	0.46 ± -0.08	2.81 ± 2.5	0.74 ± 0.43	0 ± -0.25
6	17.46 ± 1.52	3.83 ± 3.28	0.32 ± -0.22	2.74 ± 2.51	0.76 ± 0.53	-0.21 ± -0.29
7	16.98 ± 1.48	3.76 ± 3.24	0.16 ± -0.36	2.67 ± 2.44	0.49 ± 0.27	-0.47 ± -0.32
8	16.84 ± 1.7	3.68 ± 3.14	0 ± -0.55	2.59 ± 2.34	0.36 ± 0.11	-0.92 ± -0.49
9	16.49 ± 1.96	3.63 ± 3.09	-0.14 ± -0.67	2.56 ± 2.32	0.28 ± 0.04	-Inf ± 0
10	15.8 ± 2.05	3.56 ± 3.04	-0.28 ± -0.8	2.47 ± 2.25	0.12 ± -0.1	-Inf ± 0
11	15.13 ± 2.09	3.49 ± 3.07	-0.42 ± -0.84	2.42 ± 2.26	0.02 ± -0.14	-Inf ± 0
12	15.53 ± 2.09	3.45 ± 3	-0.53 ± -0.98	2.4 ± 2.21	-0.05 ± -0.23	-Inf ± 0
13	14.83 ± 2.16	3.4 ± 2.95	-0.65 ± -1.09	2.34 ± 2.11	-0.14 ± -0.37	-Inf ± 0
14	14.14 ± 2.2	3.37 ± 2.93	-0.73 ± -1.17	2.34 ± 2.15	-0.17 ± -0.37	-Inf ± 0
15	13.57 ± 2.62	3.32 ± 2.88	-0.84 ± -1.28	2.26 ± 2.08	-0.29 ± -0.48	-Inf ± 0
16	13.71 ± 2.1	3.27 ± 2.79	-0.94 ± -1.42	2.21 ± 2	-0.35 ± -0.56	-Inf ± 0
17	12.88 ± 2.3	3.26 ± 2.79	-1 ± -1.48	2.25 ± 2	-0.36 ± -0.6	-Inf ± 0
18	12.36 ± 2.45	3.19 ± 2.71	-1.12 ± -1.6	2.1 ± 1.91	-0.54 ± -0.73	-Inf ± 0
19	12.11 ± 2.45	3.15 ± 2.65	-1.21 ± -1.71	2.1 ± 1.86	-0.56 ± -0.8	-Inf ± 0
20	11.57 ± 2.34	3.13 ± 2.68	-1.27 ± -1.72	2.11 ± 1.93	-0.57 ± -0.75	-Inf ± 0
21	11.14 ± 2.36	3.1 ± 2.64	-1.34 ± -1.8	2.07 ± 1.86	-0.64 ± -0.84	-Inf ± 0
22	10.57 ± 2.31	3.04 ± 2.64	-1.44 ± -1.84	1.98 ± 1.88	-0.75 ± -0.84	-Inf ± 0
23	10.39 ± 2.2	3.01 ± 2.62	-1.52 ± -1.91	1.96 ± 1.81	-0.78 ± -0.94	-Inf ± 0
24	9.7 ± 2.31	2.95 ± 2.48	-1.61 ± -2.08	1.89 ± 1.68	-0.89 ± -1.1	-Inf ± 0
25	9.62 ± 2.45	2.91 ± 2.46	-1.69 ± -2.13	1.86 ± 1.7	-0.93 ± -1.1	-Inf ± 0
26	9.1 ± 2.49	2.9 ± 2.46	-1.73 ± -2.17	1.89 ± 1.73	-0.91 ± -1.08	-Inf ± 0
27	8.81 ± 2.36	2.88 ± 2.48	-1.78 ± -2.18	1.86 ± 1.7	-0.97 ± -1.13	-Inf ± 0
28	8.58 ± 2.55	2.84 ± 2.45	-1.85 ± -2.25	1.79 ± 1.71	-1.06 ± -1.14	-Inf ± 0
29	7.68 ± 2.23	2.79 ± 2.4	-1.94 ± -2.32	1.72 ± 1.62	-1.15 ± -1.26	-Inf ± 0
30	7.59 ± 2.26	2.79 ± 2.46	-1.97 ± -2.29	1.78 ± 1.69	-1.1 ± -1.19	-Inf ± 0
31	7.53 ± 2.13	2.8 ± 2.47	-1.99 ± -2.32	1.81 ± 1.71	-1.09 ± -1.2	-Inf ± 0
32	7.12 ± 2.16	2.76 ± 2.45	-2.06 ± -2.37	1.71 ± 1.68	-1.22 ± -1.24	-Inf ± 0
33	6.22 ± 2.51	2.75 ± 2.47	-2.09 ± -2.38	1.74 ± 1.68	-1.2 ± -1.26	-Inf ± 0
34	6.26 ± 2.17	2.75 ± 2.47	-2.12 ± -2.4	1.74 ± 1.68	-1.21 ± -1.26	-Inf ± 0
35	6.19 ± 2.15	2.7 ± 2.41	-2.2 ± -2.48	1.65 ± 1.62	-1.32 ± -1.35	-Inf ± 0
36	5.36 ± 2.06	2.65 ± 2.35	-2.27 ± -2.58	1.59 ± 1.56	-1.38 ± -1.42	-Inf ± 0
37	4.63 ± 2.22	2.63 ± 2.38	-2.31 ± -2.57	1.62 ± 1.63	-1.37 ± -1.35	-Inf ± 0

The average of the prevalence, the CFU-values per square meter (cfu/sqm) and per gram manure are calculated over all iterations. The CFU values in the intestine as well as the excreted and ingested quantities are obtained by calculating the average over all animals in one iteration at first and then over all iterations. The decadic logarithm of zero or negative values is negative infinite (-Inf).

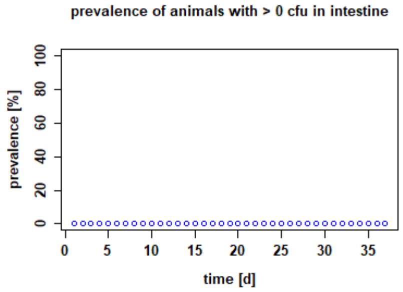

Figure S17. Calculated infection dynamic for the scenario when the pen is contaminated with 10^5 CFU (10^5 CFU in 5.38 kg/litter/m^2; i.e. 1.27 log$_{10}$/g litter) at the beginning of the fattening period (day 1). Dotplots with results for 90 negative birds, Ross 308, feeding duration 36 days, stocking density 39 kg/m^2, 1000 g litter/m^2 (no infection of birds, thus no plots).

Table S17a (CFU). Calculated numeric values for scenario when the pen is contaminated with 10^5 CFU (10^5 CFU in 5.38 kg/litter/m^2; i.e. 1.27 log$_{10}$/g litter) at the beginning of the fattening period (day 1). Setting: 90 negative birds, Ross 308, feeding duration 36 days, stocking density 39 kg/m^2, 1000 g litter/m^2

day	prevalence in %	cfu/sqm	cfu/g manure	cfu in intestine	cfu/g feces	cfu ingested per animal
	(mean ± sd)	(mean ± sd)	(mean ± sd)	(mean ± sd)	(mean ± sd)	(mean ± sd)
1	0 ± 0	9.3e3 ± 0e3	8 ± 0	0e0 ± 0	0e0 ± 0	0e0 ± 0
2	0 ± 0	4.6e3 ± 0e3	3.5 ± 0	0e0 ± 0	0e0 ± 0	0e0 ± 0
3	0 ± 0	2.3e3 ± 0e3	1.4 ± 0	0e0 ± 0	0e0 ± 0	0e0 ± 0
4	0 ± 0	1.2e3 ± 0e3	5.4e-1 ± 0e-1	0e0 ± 0	0e0 ± 0	0e0 ± 0
5	0 ± 0	5.8e2 ± 0e2	2.1e-1 ± 0e-1	0e0 ± 0	0e0 ± 0	0e0 ± 0
6	0 ± 0	2.9e2 ± 0e2	9e-2 ± 0e-2	0e0 ± 0	0e0 ± 0	0e0 ± 0
7	0 ± 0	1.5e2 ± 0e2	3.6e-2 ± 0e-2	0e0 ± 0	0e0 ± 0	0e0 ± 0
8	0 ± 0	7.3e1 ± 0e1	1.5e-2 ± 0e-2	0e0 ± 0	0e0 ± 0	0e0 ± 0
9	0 ± 0	3.6e1 ± 0e1	6.3e-3 ± 0e-3	0e0 ± 0	0e0 ± 0	0e0 ± 0
10	0 ± 0	1.8e1 ± 0e1	2.6e-3 ± 0e-3	0e0 ± 0	0e0 ± 0	0e0 ± 0
11	0 ± 0	9.1 ± 0	1.1e-3 ± 0e-3	0e0 ± 0	0e0 ± 0	0e0 ± 0
12	0 ± 0	4.5 ± 0	4.7e-4 ± 0e-4	0e0 ± 0	0e0 ± 0	0e0 ± 0
13	0 ± 0	2.3 ± 0	2e-4 ± 0e-4	0e0 ± 0	0e0 ± 0	0e0 ± 0
14	0 ± 0	1.1 ± 0	8.9e-5 ± 0e-5	0e0 ± 0	0e0 ± 0	0e0 ± 0
15	0 ± 0	5.7e-1 ± 0e-1	3.9e-5 ± 0e-5	0e0 ± 0	0e0 ± 0	0e0 ± 0
16	0 ± 0	2.8e-1 ± 0e-1	1.7e-5 ± 0e-5	0e0 ± 0	0e0 ± 0	0e0 ± 0
17	0 ± 0	1.4e-1 ± 0e-1	7.7e-6 ± 0e-6	0e0 ± 0	0e0 ± 0	0e0 ± 0
18	0 ± 0	7.1e-2 ± 0e-2	3.4e-6 ± 0e-6	0e0 ± 0	0e0 ± 0	0e0 ± 0
19	0 ± 0	3.5e-2 ± 0e-2	1.5e-6 ± 0e-6	0e0 ± 0	0e0 ± 0	0e0 ± 0
20	0 ± 0	1.8e-2 ± 0e-2	7e-7 ± 0e-7	0e0 ± 0	0e0 ± 0	0e0 ± 0
21	0 ± 0	8.9e-3 ± 0e-3	3.2e-7 ± 0e-7	0e0 ± 0	0e0 ± 0	0e0 ± 0
22	0 ± 0	4.4e-3 ± 0e-3	1.5e-7 ± 0e-7	0e0 ± 0	0e0 ± 0	0e0 ± 0
23	0 ± 0	2.2e-3 ± 0e-3	6.7e-8 ± 0e-8	0e0 ± 0	0e0 ± 0	0e0 ± 0
24	0 ± 0	1.1e-3 ± 0e-3	3.1e-8 ± 0e-8	0e0 ± 0	0e0 ± 0	0e0 ± 0
25	0 ± 0	5.5e-4 ± 0e-4	1.4e-8 ± 0e-8	0e0 ± 0	0e0 ± 0	0e0 ± 0
26	0 ± 0	2.8e-4 ± 0e-4	6.5e-9 ± 0e-9	0e0 ± 0	0e0 ± 0	0e0 ± 0
27	0 ± 0	1.4e-4 ± 0e-4	3e-9 ± 0e-9	0e0 ± 0	0e0 ± 0	0e0 ± 0
28	0 ± 0	6.9e-5 ± 0e-5	1.4e-9 ± 0e-9	0e0 ± 0	0e0 ± 0	0e0 ± 0
29	0 ± 0	3.5e-5 ± 0e-5	6.5e-10 ± 0e-10	0e0 ± 0	0e0 ± 0	0e0 ± 0
30	0 ± 0	1.7e-5 ± 0e-5	3e-10 ± 0e-10	0e0 ± 0	0e0 ± 0	0e0 ± 0
31	0 ± 0	8.7e-6 ± 0e-6	1.4e-10 ± 0e-10	0e0 ± 0	0e0 ± 0	0e0 ± 0
32	0 ± 0	4.3e-6 ± 0e-6	6.6e-11 ± 0e-11	0e0 ± 0	0e0 ± 0	0e0 ± 0
33	0 ± 0	2.2e-6 ± 0e-6	3.1e-11 ± 0e-11	0e0 ± 0	0e0 ± 0	0e0 ± 0
34	0 ± 0	1.1e-6 ± 0e-6	1.5e-11 ± 0e-11	0e0 ± 0	0e0 ± 0	0e0 ± 0
35	0 ± 0	5.4e-7 ± 0e-7	6.9e-12 ± 0e-12	0e0 ± 0	0e0 ± 0	0e0 ± 0
36	0 ± 0	2.7e-7 ± 0e-7	3.2e-12 ± 0e-12	0e0 ± 0	0e0 ± 0	0e0 ± 0
37	0 ± 0	1.4e-7 ± 0e-7	1.5e-12 ± 0e-12	0e0 ± 0	0e0 ± 0	0e0 ± 0

The average of the prevalence, the CFU-values per square meter (cfu/sqm) and per gram manure are calculated over all iterations. The CFU values in the intestine as well as the excreted and ingested quantities are obtained by calculating the average over all animals in one iteration at first and then over all iterations.

Table S17b (log$_{10}$ CFU). Calculated numeric values for scenario when the pen is contaminated with 10^5 CFU (10^5 CFU in 5.38 kg/litter/m^2; i.e. 1.27 log$_{10}$/g litter) at the beginning of the fattening period (day 1). Setting: 90 negative birds, Ross 308, feeding duration 36 days, stocking density 39 kg/m^2, 1000 g litter/m^2

day	prevalence in %	cfu/sqm	cfu/g manure	cfu in intestine	cfu/g feces	cfu ingested per animal
	(% mean ± sd)	(log10 mean ± sd)	(log10 mean ± sd)	(log10 mean ± sd)	(log10 mean ± sd)	(log10 mean ± sd)
1	0 ± 0	3.97 ± -Inf	0.9 ± -Inf	-Inf ± -Inf	-Inf ± -Inf	-Inf ± 0
2	0 ± 0	3.67 ± -Inf	0.54 ± -Inf	-Inf ± -Inf	-Inf ± -Inf	-Inf ± 0
3	0 ± 0	3.37 ± -Inf	0.15 ± -Inf	-Inf ± -Inf	-Inf ± -Inf	-Inf ± 0
4	0 ± 0	3.06 ± -Inf	-0.27 ± -Inf	-Inf ± -Inf	-Inf ± -Inf	-Inf ± 0
5	0 ± 0	2.76 ± -Inf	-0.68 ± -Inf	-Inf ± -Inf	-Inf ± -Inf	-Inf ± 0
6	0 ± 0	2.46 ± -Inf	-1.05 ± -Inf	-Inf ± -Inf	-Inf ± -Inf	-Inf ± 0
7	0 ± 0	2.16 ± -Inf	-1.44 ± -Inf	-Inf ± -Inf	-Inf ± -Inf	-Inf ± 0
8	0 ± 0	1.86 ± -Inf	-1.82 ± -Inf	-Inf ± -Inf	-Inf ± -Inf	-Inf ± 0
9	0 ± 0	1.56 ± -Inf	-2.2 ± -Inf	-Inf ± -Inf	-Inf ± -Inf	-Inf ± 0
10	0 ± 0	1.26 ± -Inf	-2.58 ± -Inf	-Inf ± -Inf	-Inf ± -Inf	-Inf ± 0
11	0 ± 0	0.96 ± -Inf	-2.95 ± -Inf	-Inf ± -Inf	-Inf ± -Inf	-Inf ± 0
12	0 ± 0	0.66 ± -Inf	-3.32 ± -Inf	-Inf ± -Inf	-Inf ± -Inf	-Inf ± 0
13	0 ± 0	0.36 ± -Inf	-3.69 ± -Inf	-Inf ± -Inf	-Inf ± -Inf	-Inf ± 0
14	0 ± 0	0.05 ± -Inf	-4.05 ± -Inf	-Inf ± -Inf	-Inf ± -Inf	-Inf ± 0
15	0 ± 0	-0.25 ± -Inf	-4.41 ± -Inf	-Inf ± -Inf	-Inf ± -Inf	-Inf ± 0
16	0 ± 0	-0.55 ± -Inf	-4.76 ± -Inf	-Inf ± -Inf	-Inf ± -Inf	-Inf ± 0
17	0 ± 0	-0.85 ± -Inf	-5.11 ± -Inf	-Inf ± -Inf	-Inf ± -Inf	-Inf ± 0
18	0 ± 0	-1.15 ± -Inf	-5.46 ± -Inf	-Inf ± -Inf	-Inf ± -Inf	-Inf ± 0
19	0 ± 0	-1.45 ± -Inf	-5.81 ± -Inf	-Inf ± -Inf	-Inf ± -Inf	-Inf ± 0
20	0 ± 0	-1.75 ± -Inf	-6.15 ± -Inf	-Inf ± -Inf	-Inf ± -Inf	-Inf ± 0
21	0 ± 0	-2.05 ± -Inf	-6.5 ± -Inf	-Inf ± -Inf	-Inf ± -Inf	-Inf ± 0
22	0 ± 0	-2.35 ± -Inf	-6.84 ± -Inf	-Inf ± -Inf	-Inf ± -Inf	-Inf ± 0
23	0 ± 0	-2.65 ± -Inf	-7.18 ± -Inf	-Inf ± -Inf	-Inf ± -Inf	-Inf ± 0
24	0 ± 0	-2.96 ± -Inf	-7.52 ± -Inf	-Inf ± -Inf	-Inf ± -Inf	-Inf ± 0
25	0 ± 0	-3.26 ± -Inf	-7.85 ± -Inf	-Inf ± -Inf	-Inf ± -Inf	-Inf ± 0
26	0 ± 0	-3.56 ± -Inf	-8.19 ± -Inf	-Inf ± -Inf	-Inf ± -Inf	-Inf ± 0
27	0 ± 0	-3.86 ± -Inf	-8.52 ± -Inf	-Inf ± -Inf	-Inf ± -Inf	-Inf ± 0
28	0 ± 0	-4.16 ± -Inf	-8.86 ± -Inf	-Inf ± -Inf	-Inf ± -Inf	-Inf ± 0
29	0 ± 0	-4.46 ± -Inf	-9.19 ± -Inf	-Inf ± -Inf	-Inf ± -Inf	-Inf ± 0
30	0 ± 0	-4.76 ± -Inf	-9.52 ± -Inf	-Inf ± -Inf	-Inf ± -Inf	-Inf ± 0
31	0 ± 0	-5.06 ± -Inf	-9.85 ± -Inf	-Inf ± -Inf	-Inf ± -Inf	-Inf ± 0
32	0 ± 0	-5.36 ± -Inf	-10.18 ± -Inf	-Inf ± -Inf	-Inf ± -Inf	-Inf ± 0
33	0 ± 0	-5.66 ± -Inf	-10.51 ± -Inf	-Inf ± -Inf	-Inf ± -Inf	-Inf ± 0
34	0 ± 0	-5.97 ± -Inf	-10.84 ± -Inf	-Inf ± -Inf	-Inf ± -Inf	-Inf ± 0
35	0 ± 0	-6.27 ± -Inf	-11.16 ± -Inf	-Inf ± -Inf	-Inf ± -Inf	-Inf ± 0
36	0 ± 0	-6.57 ± -Inf	-11.49 ± -Inf	-Inf ± -Inf	-Inf ± -Inf	-Inf ± 0
37	0 ± 0	-6.87 ± -Inf	-11.82 ± -Inf	-Inf ± -Inf	-Inf ± -Inf	-Inf ± 0

The average of the prevalence, the CFU-values per square meter (cfu/sqm) and per gram manure are calculated over all iterations. The CFU values in the intestine as well as the excreted and ingested quantities are obtained by calculating the average over all animals in one iteration at first and then over all iterations. The decadic logarithm of zero or negative values is negative infinite (-Inf).

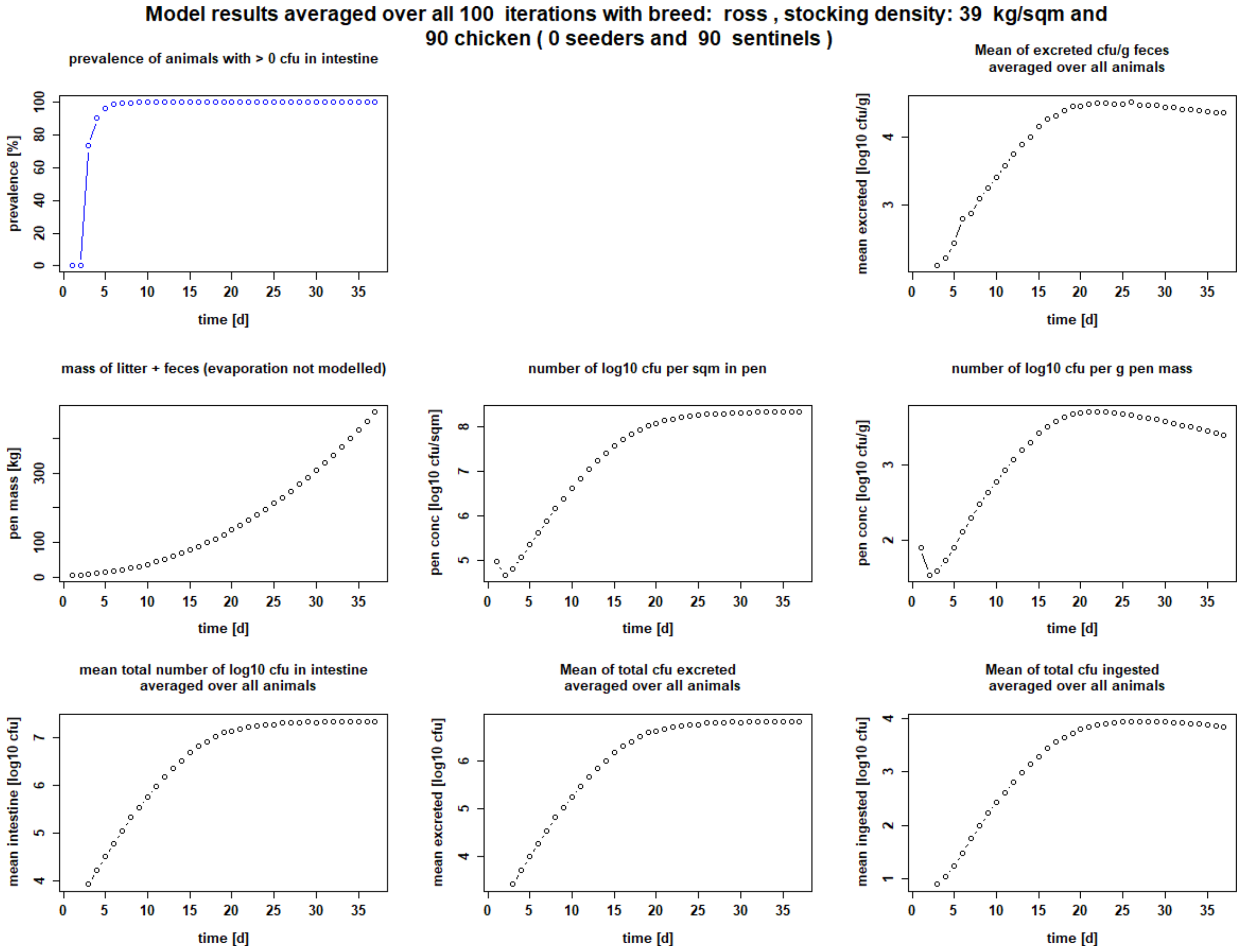

Figure S18. Calculated infection dynamic for the scenario when the pen is contaminated with 10^6 CFU (10^6 CFU in 5.38 kg/litter/m^2; i.e. 2.27 log_{10}/g litter) at the beginning of the fattening period (day 1; reference). Dotplots with results for 90 negative birds, Ross 308, feeding duration 36 days, stocking density 39 kg/m^2, 1000 g litter/m^2

Table S18a (CFU). Calculated numeric values for scenario when the pen is contaminated with 10^6 CFU (10^6 CFU in 5.38 kg/litter/m^2; i.e. 2.27 log_{10}/g litter) at the beginning of the fattening period (day 1; reference). Setting: 90 negative birds, Ross 308, feeding duration 36 days, stocking density 39 kg/m^2, 1000 g litter/m^2

day	prevalence in %	cfu/sqm	cfu/g manure	cfu in intestine	cfu/g feces	cfu ingested per animal
	(mean ± sd)	(mean ± sd)	(mean ± sd)	(mean ± sd)	(mean ± sd)	(mean ± sd)
1	0 ± 0	9.3e4 ± 0e4	8e1 ± 0e1	0e0 ± 0	0e0 ± 0	0e0 ± 0
2	0 ± 0	4.6e4 ± 0e4	3.5e1 ± 0e1	0e0 ± 0	0e0 ± 0	0e0 ± 0
3	73.39 ± 4.87	6.6e4 ± 1.2e4	4e1 ± 0.7e1	8.4e3 ± 2.4e3	1.3e2 ± 0.4e2	8 ± 0
4	90.72 ± 3.28	1.2e5 ± 0.3e5	5.5e1 ± 1.3e1	1.7e4 ± 0.5e4	1.7e2 ± 0.5e2	1.11e1 ± 0.2e1
5	96.74 ± 2.43	2.2e5 ± 0.7e5	8.1e1 ± 2.6e1	3.2e4 ± 1.2e4	2.8e2 ± 1.1e2	1.79e1 ± 0.4e1
6	98.9 ± 1.38	4.2e5 ± 1.7e5	1.3e2 ± 0.5e2	6.1e4 ± 2.8e4	6.4e2 ± 3e2	3.11e1 ± 1e1
7	99.79 ± 0.61	7.8e5 ± 3.5e5	2e2 ± 0.9e2	1.1e5 ± 0.5e5	7.6e2 ± 3.6e2	5.74e1 ± 2.4e1
8	99.94 ± 0.24	1.5e6 ± 0.8e6	3e2 ± 1.6e2	2.1e5 ± 1.3e5	1.3e3 ± 0.7e3	9.83e1 ± 4.4e1
9	100 ± 0	2.5e6 ± 1.5e6	4.3e2 ± 2.5e2	3.5e5 ± 2.2e5	1.8e3 ± 1.2e3	1.68e2 ± 0.9e2
10	100 ± 0	4.1e6 ± 2.3e6	6e2 ± 3.4e2	5.8e5 ± 3.3e5	2.6e3 ± 1.5e3	2.67e2 ± 1.6e2
11	100 ± 0	7e6 ± 4.3e6	8.5e2 ± 5.3e2	9.8e5 ± 6.5e5	3.9e3 ± 2.6e3	4.11e2 ± 2.3e2
12	100 ± 0	1.1e7 ± 0.7e7	1.2e3 ± 0.7e3	1.6e6 ± 0.9e6	5.6e3 ± 3.3e3	6.45e2 ± 4e2
13	100 ± 0	1.7e7 ± 1.1e7	1.6e3 ± 1e3	2.3e6 ± 1.6e6	7.8e3 ± 5.2e3	9.58e2 ± 5.6e2
14	100 ± 0	2.5e7 ± 1.5e7	2e3 ± 1.2e3	3.3e6 ± 2e6	10e3 ± 6.1e3	1.39e3 ± 0.9e3
15	100 ± 0	3.8e7 ± 2.4e7	2.6e3 ± 1.6e3	5e6 ± 3.4e6	1.4e4 ± 0.9e4	1.91e3 ± 1.2e3
16	100 ± 0	5.3e7 ± 3.1e7	3.2e3 ± 1.9e3	6.7e6 ± 3.9e6	1.8e4 ± 1.1e4	2.7e3 ± 1.7e3
17	100 ± 0	6.8e7 ± 3.6e7	3.7e3 ± 2e3	8.3e6 ± 4.4e6	2.1e4 ± 1.1e4	3.55e3 ± 2.1e3
18	100 ± 0	8.7e7 ± 4.1e7	4.2e3 ± 2e3	1.1e7 ± 0.5e7	2.4e4 ± 1.1e4	4.4e3 ± 2.3e3
19	100 ± 0	1.1e8 ± 0.5e8	4.7e3 ± 2e3	1.3e7 ± 0.5e7	2.8e4 ± 1.2e4	5.31e3 ± 2.5e3
20	100 ± 0	1.2e8 ± 0.5e8	4.8e3 ± 1.8e3	1.4e7 ± 0.5e7	2.9e4 ± 1.1e4	6.32e3 ± 2.7e3
21	100 ± 0	1.4e8 ± 0.5e8	5e3 ± 1.6e3	1.6e7 ± 0.5e7	3.1e4 ± 1e4	6.89e3 ± 2.6e3
22	100 ± 0	1.5e8 ± 0.4e8	5e3 ± 1.5e3	1.7e7 ± 0.5e7	3.1e4 ± 1e4	7.56e3 ± 2.5e3
23	100 ± 0	1.7e8 ± 0.4e8	5e3 ± 1.3e3	1.8e7 ± 0.5e7	3.2e4 ± 0.9e4	8e3 ± 2.3e3
24	100 ± 0	1.8e8 ± 0.4e8	4.8e3 ± 1.1e3	1.8e7 ± 0.5e7	3.1e4 ± 0.8e4	8.35e3 ± 2.2e3
25	100 ± 0	1.8e8 ± 0.4e8	4.7e3 ± 1e3	1.9e7 ± 0.5e7	3.1e4 ± 0.8e4	8.45e3 ± 2e3
26	100 ± 0	1.9e8 ± 0.4e8	4.6e3 ± 0.9e3	2e7 ± 0.4e7	3.2e4 ± 0.7e4	8.53e3 ± 1.9e3
27	100 ± 0	2e8 ± 0.4e8	4.3e3 ± 0.8e3	2e7 ± 0.5e7	3e4 ± 0.7e4	8.75e3 ± 1.7e3
28	100 ± 0	2e8 ± 0.3e8	4.1e3 ± 0.7e3	2.1e7 ± 0.4e7	2.9e4 ± 0.6e4	8.64e3 ± 1.7e3
29	100 ± 0	2.1e8 ± 0.3e8	4e3 ± 0.6e3	2.2e7 ± 0.4e7	2.9e4 ± 0.6e4	8.56e3 ± 1.4e3
30	100 ± 0	2.1e8 ± 0.3e8	3.7e3 ± 0.6e3	2.1e7 ± 0.4e7	2.8e4 ± 0.6e4	8.54e3 ± 1.4e3
31	100 ± 0	2.1e8 ± 0.3e8	3.5e3 ± 0.6e3	2.2e7 ± 0.4e7	2.7e4 ± 0.5e4	8.3e3 ± 1.3e3
32	100 ± 0	2.2e8 ± 0.3e8	3.3e3 ± 0.5e3	2.2e7 ± 0.4e7	2.6e4 ± 0.4e4	8.1e3 ± 1.3e3
33	100 ± 0	2.2e8 ± 0.3e8	3.1e3 ± 0.4e3	2.2e7 ± 0.4e7	2.6e4 ± 0.4e4	7.87e3 ± 1.1e3
34	100 ± 0	2.2e8 ± 0.3e8	3e3 ± 0.4e3	2.2e7 ± 0.4e7	2.5e4 ± 0.4e4	7.71e3 ± 1e3
35	100 ± 0	2.2e8 ± 0.3e8	2.8e3 ± 0.3e3	2.2e7 ± 0.4e7	2.4e4 ± 0.4e4	7.49e3 ± 0.9e3
36	100 ± 0	2.2e8 ± 0.3e8	2.6e3 ± 0.3e3	2.2e7 ± 0.4e7	2.3e4 ± 0.5e4	7.21e3 ± 0.8e3
37	100 ± 0	2.2e8 ± 0.3e8	2.5e3 ± 0.3e3	2.2e7 ± 0.4e7	2.2e4 ± 0.4e4	6.93e3 ± 0.8e3

The average of the prevalence, the CFU-values per square meter (cfu/sqm) and per gram manure are calculated over all iterations. The CFU values in the intestine as well as the excreted and ingested quantities are obtained by calculating the average over all animals in one iteration at first and then over all iterations.

Table S18b (log_{10} CFU). Calculated numeric values for scenario when the pen is contaminated with 10^6 CFU (10^6 CFU in 5.38 kg/litter/m^2; i.e. 2.27 log_{10}/g litter) at the beginning of the fattening period (day 1; reference). Setting: 90 negative birds, Ross 308, feeding duration 36 days, stocking density 39 kg/m^2, 1000 g litter/m^2

day	prevalence in %	cfu/sqm	cfu/g manure	cfu in intestine	cfu/g feces	cfu ingested per animal
	(% mean ± sd)	(log10 mean ± sd)	(log10 mean ± sd)	(log10 mean ± sd)	(log10 mean ± sd)	(log10 mean ± sd)
1	0 ± 0	4.97 ± -Inf	1.9 ± -Inf	-Inf ± -Inf	-Inf ± -Inf	-Inf ± 0
2	0 ± 0	4.67 ± -Inf	1.54 ± -Inf	-Inf ± -Inf	-Inf ± -Inf	-Inf ± 0
3	73.39 ± 4.87	4.82 ± 4.07	1.6 ± 0.85	3.93 ± 3.37	2.12 ± 1.56	0.9 ± 0
4	90.72 ± 3.28	5.07 ± 4.45	1.74 ± 1.11	4.23 ± 3.7	2.23 ± 1.7	1.05 ± 0.33
5	96.74 ± 2.43	5.35 ± 4.86	1.91 ± 1.42	4.51 ± 4.1	2.44 ± 2.03	1.25 ± 0.64
6	98.9 ± 1.38	5.62 ± 5.23	2.11 ± 1.72	4.79 ± 4.45	2.81 ± 2.47	1.49 ± 1.01
7	99.79 ± 0.61	5.89 ± 5.54	2.29 ± 1.94	5.06 ± 4.74	2.88 ± 2.56	1.76 ± 1.37
8	99.94 ± 0.24	6.16 ± 5.9	2.48 ± 2.21	5.33 ± 5.1	3.1 ± 2.87	1.99 ± 1.65
9	100 ± 0	6.39 ± 6.17	2.63 ± 2.4	5.54 ± 5.34	3.26 ± 3.06	2.23 ± 1.96
10	100 ± 0	6.62 ± 6.37	2.78 ± 2.53	5.76 ± 5.52	3.42 ± 3.17	2.43 ± 2.2
11	100 ± 0	6.84 ± 6.64	2.93 ± 2.73	5.99 ± 5.81	3.59 ± 3.41	2.61 ± 2.37
12	100 ± 0	7.05 ± 6.82	3.07 ± 2.84	6.19 ± 5.96	3.75 ± 3.52	2.81 ± 2.6
13	100 ± 0	7.24 ± 7.04	3.2 ± 2.99	6.37 ± 6.2	3.89 ± 3.72	2.98 ± 2.75
14	100 ± 0	7.4 ± 7.18	3.3 ± 3.08	6.52 ± 6.3	4 ± 3.79	3.14 ± 2.94
15	100 ± 0	7.58 ± 7.38	3.42 ± 3.22	6.7 ± 6.53	4.15 ± 3.98	3.28 ± 3.06
16	100 ± 0	7.72 ± 7.49	3.51 ± 3.28	6.83 ± 6.59	4.26 ± 4.03	3.43 ± 3.23
17	100 ± 0	7.83 ± 7.56	3.57 ± 3.29	6.92 ± 6.64	4.31 ± 4.03	3.55 ± 3.32
18	100 ± 0	7.94 ± 7.61	3.62 ± 3.3	7.02 ± 6.69	4.38 ± 4.05	3.64 ± 3.37
19	100 ± 0	8.03 ± 7.66	3.67 ± 3.3	7.11 ± 6.74	4.45 ± 4.08	3.73 ± 3.4
20	100 ± 0	8.09 ± 7.66	3.68 ± 3.26	7.13 ± 6.7	4.46 ± 4.02	3.8 ± 3.43
21	100 ± 0	8.14 ± 7.66	3.7 ± 3.21	7.19 ± 6.7	4.49 ± 4	3.84 ± 3.41
22	100 ± 0	8.18 ± 7.65	3.7 ± 3.17	7.22 ± 6.71	4.5 ± 3.99	3.88 ± 3.39
23	100 ± 0	8.22 ± 7.64	3.7 ± 3.11	7.25 ± 6.69	4.5 ± 3.94	3.9 ± 3.37
24	100 ± 0	8.24 ± 7.61	3.68 ± 3.05	7.27 ± 6.67	4.49 ± 3.89	3.92 ± 3.34
25	100 ± 0	8.26 ± 7.61	3.67 ± 3.01	7.28 ± 6.68	4.48 ± 3.88	3.93 ± 3.29
26	100 ± 0	8.29 ± 7.57	3.66 ± 2.94	7.31 ± 6.65	4.51 ± 3.85	3.93 ± 3.27
27	100 ± 0	8.3 ± 7.58	3.64 ± 2.92	7.31 ± 6.68	4.47 ± 3.84	3.94 ± 3.23
28	100 ± 0	8.31 ± 7.52	3.61 ± 2.82	7.32 ± 6.62	4.46 ± 3.77	3.94 ± 3.22
29	100 ± 0	8.33 ± 7.53	3.6 ± 2.8	7.34 ± 6.64	4.46 ± 3.76	3.93 ± 3.14
30	100 ± 0	8.33 ± 7.52	3.57 ± 2.77	7.33 ± 6.64	4.44 ± 3.76	3.93 ± 3.13
31	100 ± 0	8.33 ± 7.53	3.54 ± 2.75	7.34 ± 6.64	4.43 ± 3.73	3.92 ± 3.12
32	100 ± 0	8.34 ± 7.47	3.52 ± 2.66	7.34 ± 6.57	4.41 ± 3.65	3.91 ± 3.11
33	100 ± 0	8.34 ± 7.46	3.5 ± 2.61	7.35 ± 6.57	4.41 ± 3.63	3.9 ± 3.03
34	100 ± 0	8.34 ± 7.44	3.47 ± 2.57	7.34 ± 6.58	4.4 ± 3.64	3.89 ± 3
35	100 ± 0	8.34 ± 7.41	3.44 ± 2.51	7.34 ± 6.61	4.37 ± 3.65	3.87 ± 2.97
36	100 ± 0	8.34 ± 7.42	3.42 ± 2.49	7.34 ± 6.63	4.36 ± 3.66	3.86 ± 2.93
37	100 ± 0	8.34 ± 7.4	3.39 ± 2.45	7.34 ± 6.57	4.35 ± 3.58	3.84 ± 2.92

The average of the prevalence, the CFU-values per square meter (cfu/sqm) and per gram manure are calculated over all iterations. The CFU values in the intestine as well as the excreted and ingested quantities are obtained by calculating the average over all animals in one iteration at first and then over all iterations. The decadic logarithm of zero or negative values is negative infinite (-Inf).

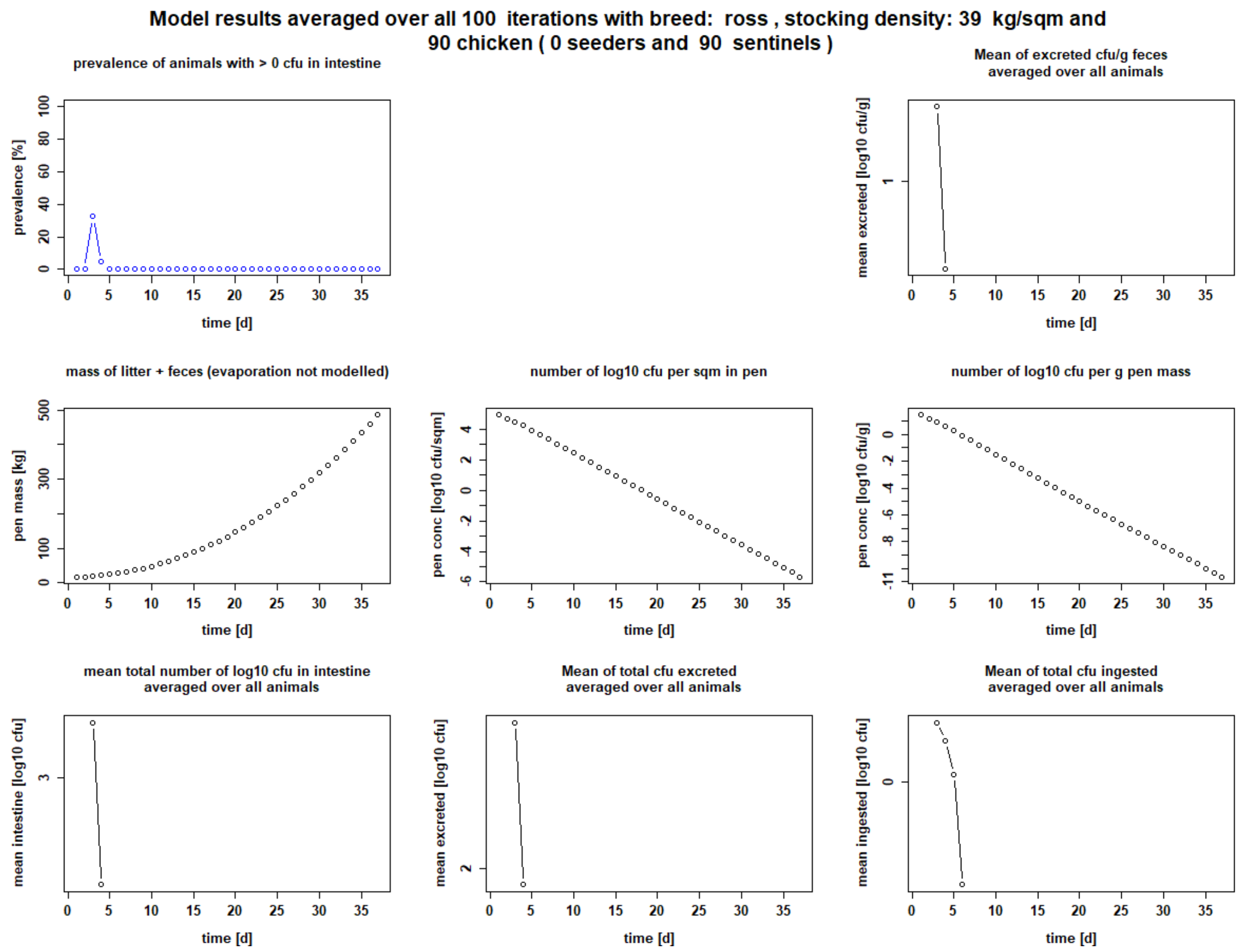

Figure S19. Calculated infection dynamic for the scenario when the pen is contaminated with 10^6 CFU at the beginning of the fattening period, measure 3000 g litter/m^2. Dotplots with results for 90 negative birds, Ross 308, feeding duration 36 days, stocking density 39 kg/m^2, 3000 g litter/m^2

Table S19a (CFU). Calculated numeric values for scenario when the pen is contaminated with 10^6 CFU at the beginning of the fattening period, measure 3000 g litter/m². Setting: 90 negative birds, Ross 308, feeding duration 36 days, stocking density 39 kg/m², 3000 g litter/m²

day	prevalence in %	cfu/sqm	cfu/g manure	cfu in intestine	cfu/g feces	cfu ingested per animal
	(mean ± sd)	(mean ± sd)	(mean ± sd)	(mean ± sd)	(mean ± sd)	(mean ± sd)
1	0 ± 0	9.3e4 ± 0e4	2.9e1 ± 0e1	0e0 ± 0	0e0 ± 0	0e0 ± 0
2	0 ± 0	4.6e4 ± 0e4	1.4e1 ± 0e1	0e0 ± 0	0e0 ± 0	0e0 ± 0
3	32.46 ± 4.42	3.3e4 ± 0.4e4	9 ± 1.2	2e3 ± 0.9e3	3e1 ± 1.4e1	3 ± 0
4	4.77 ± 11.57	1.8e4 ± 0.5e4	4.3 ± 1.2	2.7e2 ± 6.8e2	2.7 ± 6.8	2.15 ± 0.4
5	0 ± 0	8.9e3 ± 2.6e3	1.9 ± 0.5	0e0 ± 0	0e0 ± 0	1.15 ± 0.4
6	0 ± 0	4.5e3 ± 1.3e3	8.6e-1 ± 2.5e-1	0e0 ± 0	0e0 ± 0	1.5e-1 ± 3.6e-1
7	0 ± 0	2.2e3 ± 0.7e3	3.7e-1 ± 1.1e-1	0e0 ± 0	0e0 ± 0	0e0 ± 0
8	0 ± 0	1.1e3 ± 0.3e3	1.6e-1 ± 0.5e-1	0e0 ± 0	0e0 ± 0	0e0 ± 0
9	0 ± 0	5.6e2 ± 1.6e2	7.2e-2 ± 2.1e-2	0e0 ± 0	0e0 ± 0	0e0 ± 0
10	0 ± 0	2.8e2 ± 0.8e2	3.1e-2 ± 0.9e-2	0e0 ± 0	0e0 ± 0	0e0 ± 0
11	0 ± 0	1.4e2 ± 0.4e2	1.4e-2 ± 0.4e-2	0e0 ± 0	0e0 ± 0	0e0 ± 0
12	0 ± 0	7e1 ± 2e1	6e-3 ± 1.8e-3	0e0 ± 0	0e0 ± 0	0e0 ± 0
13	0 ± 0	3.5e1 ± 1e1	2.7e-3 ± 0.8e-3	0e0 ± 0	0e0 ± 0	0e0 ± 0
14	0 ± 0	1.7e1 ± 0.5e1	1.2e-3 ± 0.3e-3	0e0 ± 0	0e0 ± 0	0e0 ± 0
15	0 ± 0	8.7 ± 2.5	5.3e-4 ± 1.5e-4	0e0 ± 0	0e0 ± 0	0e0 ± 0
16	0 ± 0	4.4 ± 1.3	2.4e-4 ± 0.7e-4	0e0 ± 0	0e0 ± 0	0e0 ± 0
17	0 ± 0	2.2 ± 0.6	1.1e-4 ± 0.3e-4	0e0 ± 0	0e0 ± 0	0e0 ± 0
18	0 ± 0	1.1 ± 0.3	4.8e-5 ± 1.4e-5	0e0 ± 0	0e0 ± 0	0e0 ± 0
19	0 ± 0	5.5e-1 ± 1.6e-1	2.2e-5 ± 0.6e-5	0e0 ± 0	0e0 ± 0	0e0 ± 0
20	0 ± 0	2.7e-1 ± 0.8e-1	1e-5 ± 0.3e-5	0e0 ± 0	0e0 ± 0	0e0 ± 0
21	0 ± 0	1.4e-1 ± 0.4e-1	4.6e-6 ± 1.3e-6	0e0 ± 0	0e0 ± 0	0e0 ± 0
22	0 ± 0	6.8e-2 ± 2e-2	2.1e-6 ± 0.6e-6	0e0 ± 0	0e0 ± 0	0e0 ± 0
23	0 ± 0	3.4e-2 ± 1e-2	9.7e-7 ± 2.8e-7	0e0 ± 0	0e0 ± 0	0e0 ± 0
24	0 ± 0	1.7e-2 ± 0.5e-2	4.5e-7 ± 1.3e-7	0e0 ± 0	0e0 ± 0	0e0 ± 0
25	0 ± 0	8.5e-3 ± 2.5e-3	2.1e-7 ± 0.6e-7	0e0 ± 0	0e0 ± 0	0e0 ± 0
26	0 ± 0	4.3e-3 ± 1.2e-3	9.6e-8 ± 2.8e-8	0e0 ± 0	0e0 ± 0	0e0 ± 0
27	0 ± 0	2.1e-3 ± 0.6e-3	4.4e-8 ± 1.3e-8	0e0 ± 0	0e0 ± 0	0e0 ± 0
28	0 ± 0	1.1e-3 ± 0.3e-3	2.1e-8 ± 0.6e-8	0e0 ± 0	0e0 ± 0	0e0 ± 0
29	0 ± 0	5.3e-4 ± 1.5e-4	9.6e-9 ± 2.8e-9	0e0 ± 0	0e0 ± 0	0e0 ± 0
30	0 ± 0	2.7e-4 ± 0.8e-4	4.5e-9 ± 1.3e-9	0e0 ± 0	0e0 ± 0	0e0 ± 0
31	0 ± 0	1.3e-4 ± 0.4e-4	2.1e-9 ± 0.6e-9	0e0 ± 0	0e0 ± 0	0e0 ± 0
32	0 ± 0	6.7e-5 ± 1.9e-5	9.9e-10 ± 2.9e-10	0e0 ± 0	0e0 ± 0	0e0 ± 0
33	0 ± 0	3.3e-5 ± 1e-5	4.6e-10 ± 1.3e-10	0e0 ± 0	0e0 ± 0	0e0 ± 0
34	0 ± 0	1.7e-5 ± 0.5e-5	2.2e-10 ± 0.6e-10	0e0 ± 0	0e0 ± 0	0e0 ± 0
35	0 ± 0	8.3e-6 ± 2.4e-6	1e-10 ± 0.3e-10	0e0 ± 0	0e0 ± 0	0e0 ± 0
36	0 ± 0	4.2e-6 ± 1.2e-6	4.9e-11 ± 1.4e-11	0e0 ± 0	0e0 ± 0	0e0 ± 0
37	0 ± 0	2.1e-6 ± 0.6e-6	2.3e-11 ± 0.7e-11	0e0 ± 0	0e0 ± 0	0e0 ± 0

The average of the prevalence, the CFU-values per square meter (cfu/sqm) and per gram manure are calculated over all iterations. The CFU values in the intestine as well as the excreted and ingested quantities are obtained by calculating the average over all animals in one iteration at first and then over all iterations.

Table S19b (log_{10} CFU).). Calculated numeric values for scenario when the pen is contaminated with 10^6 CFU at the beginning of the fattening period, measure 3000 g litter/m^2.
Setting: 90 negative birds, Ross 308, feeding duration 36 days, stocking density 39 kg/m^2, 3000 g litter/m^2

day	prevalence in %	cfu/sqm	cfu/g manure	cfu in intestine	cfu/g feces	cfu ingested per animal
	(% mean ± sd)	(log10 mean ± sd)	(log10 mean ± sd)	(log10 mean ± sd)	(log10 mean ± sd)	(log10 mean ± sd)
1	0 ± 0	4.97 ± -Inf	1.47 ± -Inf	-Inf ± -Inf	-Inf ± -Inf	-Inf ± 0
2	0 ± 0	4.67 ± -Inf	1.14 ± -Inf	-Inf ± -Inf	-Inf ± -Inf	-Inf ± 0
3	32.46 ± 4.42	4.52 ± 3.65	0.96 ± 0.08	3.29 ± 2.95	1.48 ± 1.14	0.48 ± 0
4	4.77 ± 11.57	4.25 ± 3.72	0.63 ± 0.1	2.44 ± 2.84	0.43 ± 0.83	0.33 ± -0.45
5	0 ± 0	3.95 ± 3.41	0.27 ± -0.26	-Inf ± -Inf	-Inf ± -Inf	0.06 ± -0.45
6	0 ± 0	3.65 ± 3.11	-0.07 ± -0.6	-Inf ± -Inf	-Inf ± -Inf	-0.82 ± -0.45
7	0 ± 0	3.35 ± 2.81	-0.43 ± -0.96	-Inf ± -Inf	-Inf ± -Inf	-Inf ± 0
8	0 ± 0	3.05 ± 2.51	-0.79 ± -1.32	-Inf ± -Inf	-Inf ± -Inf	-Inf ± 0
9	0 ± 0	2.75 ± 2.21	-1.14 ± -1.68	-Inf ± -Inf	-Inf ± -Inf	-Inf ± 0
10	0 ± 0	2.45 ± 1.91	-1.5 ± -2.04	-Inf ± -Inf	-Inf ± -Inf	-Inf ± 0
11	0 ± 0	2.15 ± 1.61	-1.86 ± -2.4	-Inf ± -Inf	-Inf ± -Inf	-Inf ± 0
12	0 ± 0	1.84 ± 1.31	-2.22 ± -2.76	-Inf ± -Inf	-Inf ± -Inf	-Inf ± 0
13	0 ± 0	1.54 ± 1.01	-2.57 ± -3.11	-Inf ± -Inf	-Inf ± -Inf	-Inf ± 0
14	0 ± 0	1.24 ± 0.71	-2.93 ± -3.46	-Inf ± -Inf	-Inf ± -Inf	-Inf ± 0
15	0 ± 0	0.94 ± 0.4	-3.28 ± -3.81	-Inf ± -Inf	-Inf ± -Inf	-Inf ± 0
16	0 ± 0	0.64 ± 0.1	-3.62 ± -4.16	-Inf ± -Inf	-Inf ± -Inf	-Inf ± 0
17	0 ± 0	0.34 ± -0.2	-3.97 ± -4.51	-Inf ± -Inf	-Inf ± -Inf	-Inf ± 0
18	0 ± 0	0.04 ± -0.5	-4.32 ± -4.85	-Inf ± -Inf	-Inf ± -Inf	-Inf ± 0
19	0 ± 0	-0.26 ± -0.8	-4.66 ± -5.2	-Inf ± -Inf	-Inf ± -Inf	-Inf ± 0
20	0 ± 0	-0.56 ± -1.1	-5 ± -5.54	-Inf ± -Inf	-Inf ± -Inf	-Inf ± 0
21	0 ± 0	-0.87 ± -1.4	-5.34 ± -5.88	-Inf ± -Inf	-Inf ± -Inf	-Inf ± 0
22	0 ± 0	-1.17 ± -1.7	-5.68 ± -6.21	-Inf ± -Inf	-Inf ± -Inf	-Inf ± 0
23	0 ± 0	-1.47 ± -2	-6.01 ± -6.55	-Inf ± -Inf	-Inf ± -Inf	-Inf ± 0
24	0 ± 0	-1.77 ± -2.3	-6.35 ± -6.89	-Inf ± -Inf	-Inf ± -Inf	-Inf ± 0
25	0 ± 0	-2.07 ± -2.61	-6.69 ± -7.22	-Inf ± -Inf	-Inf ± -Inf	-Inf ± 0
26	0 ± 0	-2.37 ± -2.91	-7.02 ± -7.56	-Inf ± -Inf	-Inf ± -Inf	-Inf ± 0
27	0 ± 0	-2.67 ± -3.21	-7.35 ± -7.89	-Inf ± -Inf	-Inf ± -Inf	-Inf ± 0
28	0 ± 0	-2.97 ± -3.51	-7.69 ± -8.22	-Inf ± -Inf	-Inf ± -Inf	-Inf ± 0
29	0 ± 0	-3.27 ± -3.81	-8.02 ± -8.55	-Inf ± -Inf	-Inf ± -Inf	-Inf ± 0
30	0 ± 0	-3.57 ± -4.11	-8.35 ± -8.88	-Inf ± -Inf	-Inf ± -Inf	-Inf ± 0
31	0 ± 0	-3.88 ± -4.41	-8.68 ± -9.21	-Inf ± -Inf	-Inf ± -Inf	-Inf ± 0
32	0 ± 0	-4.18 ± -4.71	-9.01 ± -9.54	-Inf ± -Inf	-Inf ± -Inf	-Inf ± 0
33	0 ± 0	-4.48 ± -5.01	-9.33 ± -9.87	-Inf ± -Inf	-Inf ± -Inf	-Inf ± 0
34	0 ± 0	-4.78 ± -5.31	-9.66 ± -10.2	-Inf ± -Inf	-Inf ± -Inf	-Inf ± 0
35	0 ± 0	-5.08 ± -5.62	-9.99 ± -10.52	-Inf ± -Inf	-Inf ± -Inf	-Inf ± 0
36	0 ± 0	-5.38 ± -5.92	-10.31 ± -10.85	-Inf ± -Inf	-Inf ± -Inf	-Inf ± 0
37	0 ± 0	-5.68 ± -6.22	-10.64 ± -11.17	-Inf ± -Inf	-Inf ± -Inf	-Inf ± 0

The average of the prevalence, the CFU-values per square meter (cfu/sqm) and per gram manure are calculated over all iterations. The CFU values in the intestine as well as the excreted and ingested quantities are obtained by calculating the average over all animals in one iteration at first and then over all iterations. The decadic logarithm of zero or negative values is negative infinite (-Inf).

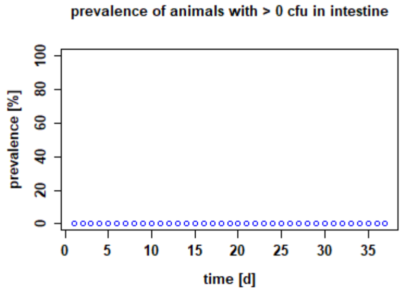

Figure S20. Calculated infection dynamic for the scenario when the pen is contaminated with 10^6 CFU at the beginning of the fattening period, measure 6000 g litter/m^2. Dotplots with results for 90 negative birds, Ross 308, feeding duration 36 days, stocking density 39 kg/m^2, 6000 g litter/m^2 (no infection of birds, thus no plots).

Table S20a (CFU). Calculated numeric values for scenario when the pen is contaminated with 10^6 CFU at the beginning of the fattening period, measure 6000 g litter/m^2. Setting: 90 negative birds, Ross 308, feeding duration 36 days, stocking density 39 kg/m^2, 6000 g litter/m^2

day	prevalence in %	cfu/sqm	cfu/g manure	cfu in intestine	cfu/g feces	cfu ingested per animal
	(mean ± sd)	(mean ± sd)	(mean ± sd)	(mean ± sd)	(mean ± sd)	(mean ± sd)
1	0 ± 0	9.3e4 ± 0e4	1.5e1 ± 0e1	0e0 ± 0	0e0 ± 0	0e0 ± 0
2	0 ± 0	4.6e4 ± 0e4	7.3 ± 0	0e0 ± 0	0e0 ± 0	0e0 ± 0
3	0 ± 0	2.3e4 ± 0e4	3.5 ± 0	0e0 ± 0	0e0 ± 0	1 ± 0
4	0 ± 0	1.2e4 ± 0e4	1.6 ± 0	0e0 ± 0	0e0 ± 0	1 ± 0
5	0 ± 0	5.8e3 ± 0e3	7.5e-1 ± 0e-1	0e0 ± 0	0e0 ± 0	0e0 ± 0
6	0 ± 0	2.9e3 ± 0e3	3.5e-1 ± 0e-1	0e0 ± 0	0e0 ± 0	0e0 ± 0
7	0 ± 0	1.5e3 ± 0e3	1.6e-1 ± 0e-1	0e0 ± 0	0e0 ± 0	0e0 ± 0
8	0 ± 0	7.3e2 ± 0e2	7.4e-2 ± 0e-2	0e0 ± 0	0e0 ± 0	0e0 ± 0
9	0 ± 0	3.6e2 ± 0e2	3.4e-2 ± 0e-2	0e0 ± 0	0e0 ± 0	0e0 ± 0
10	0 ± 0	1.8e2 ± 0e2	1.5e-2 ± 0e-2	0e0 ± 0	0e0 ± 0	0e0 ± 0
11	0 ± 0	9.1e1 ± 0e1	6.9e-3 ± 0e-3	0e0 ± 0	0e0 ± 0	0e0 ± 0
12	0 ± 0	4.5e1 ± 0e1	3.1e-3 ± 0e-3	0e0 ± 0	0e0 ± 0	0e0 ± 0
13	0 ± 0	2.3e1 ± 0e1	1.4e-3 ± 0e-3	0e0 ± 0	0e0 ± 0	0e0 ± 0
14	0 ± 0	1.1e1 ± 0e1	6.4e-4 ± 0e-4	0e0 ± 0	0e0 ± 0	0e0 ± 0
15	0 ± 0	5.7 ± 0	2.9e-4 ± 0e-4	0e0 ± 0	0e0 ± 0	0e0 ± 0
16	0 ± 0	2.8 ± 0	1.3e-4 ± 0e-4	0e0 ± 0	0e0 ± 0	0e0 ± 0
17	0 ± 0	1.4 ± 0	6.1e-5 ± 0e-5	0e0 ± 0	0e0 ± 0	0e0 ± 0
18	0 ± 0	7.1e-1 ± 0e-1	2.8e-5 ± 0e-5	0e0 ± 0	0e0 ± 0	0e0 ± 0
19	0 ± 0	3.5e-1 ± 0e-1	1.3e-5 ± 0e-5	0e0 ± 0	0e0 ± 0	0e0 ± 0
20	0 ± 0	1.8e-1 ± 0e-1	5.9e-6 ± 0e-6	0e0 ± 0	0e0 ± 0	0e0 ± 0
21	0 ± 0	8.9e-2 ± 0e-2	2.7e-6 ± 0e-6	0e0 ± 0	0e0 ± 0	0e0 ± 0
22	0 ± 0	4.4e-2 ± 0e-2	1.2e-6 ± 0e-6	0e0 ± 0	0e0 ± 0	0e0 ± 0
23	0 ± 0	2.2e-2 ± 0e-2	5.8e-7 ± 0e-7	0e0 ± 0	0e0 ± 0	0e0 ± 0
24	0 ± 0	1.1e-2 ± 0e-2	2.7e-7 ± 0e-7	0e0 ± 0	0e0 ± 0	0e0 ± 0
25	0 ± 0	5.5e-3 ± 0e-3	1.2e-7 ± 0e-7	0e0 ± 0	0e0 ± 0	0e0 ± 0
26	0 ± 0	2.8e-3 ± 0e-3	5.8e-8 ± 0e-8	0e0 ± 0	0e0 ± 0	0e0 ± 0
27	0 ± 0	1.4e-3 ± 0e-3	2.7e-8 ± 0e-8	0e0 ± 0	0e0 ± 0	0e0 ± 0
28	0 ± 0	6.9e-4 ± 0e-4	1.3e-8 ± 0e-8	0e0 ± 0	0e0 ± 0	0e0 ± 0
29	0 ± 0	3.5e-4 ± 0e-4	5.9e-9 ± 0e-9	0e0 ± 0	0e0 ± 0	0e0 ± 0
30	0 ± 0	1.7e-4 ± 0e-4	2.8e-9 ± 0e-9	0e0 ± 0	0e0 ± 0	0e0 ± 0
31	0 ± 0	8.7e-5 ± 0e-5	1.3e-9 ± 0e-9	0e0 ± 0	0e0 ± 0	0e0 ± 0
32	0 ± 0	4.3e-5 ± 0e-5	6.1e-10 ± 0e-10	0e0 ± 0	0e0 ± 0	0e0 ± 0
33	0 ± 0	2.2e-5 ± 0e-5	2.9e-10 ± 0e-10	0e0 ± 0	0e0 ± 0	0e0 ± 0
34	0 ± 0	1.1e-5 ± 0e-5	1.4e-10 ± 0e-10	0e0 ± 0	0e0 ± 0	0e0 ± 0
35	0 ± 0	5.4e-6 ± 0e-6	6.4e-11 ± 0e-11	0e0 ± 0	0e0 ± 0	0e0 ± 0
36	0 ± 0	2.7e-6 ± 0e-6	3.1e-11 ± 0e-11	0e0 ± 0	0e0 ± 0	0e0 ± 0
37	0 ± 0	1.4e-6 ± 0e-6	1.4e-11 ± 0e-11	0e0 ± 0	0e0 ± 0	0e0 ± 0

The average of the prevalence, the CFU-values per square meter (cfu/sqm) and per gram manure are calculated over all iterations. The CFU values in the intestine as well as the excreted and ingested quantities are obtained by calculating the average over all animals in one iteration at first and then over all iterations.

Table S20b (log_{10} CFU). Calculated numeric values for scenario when the pen is contaminated with 10^6 CFU at the beginning of the fattening period, measure 6000 g litter/m^2. Setting: 90 negative birds, Ross 308, feeding duration 36 days, stocking density 39 kg/m^2, 6000 g litter/m^2

day	prevalence in %	cfu/sqm	cfu/g manure	cfu in intestine	cfu/g feces	cfu ingested per animal
	(% mean ± sd)	(log10 mean ± sd)	(log10 mean ± sd)	(log10 mean ± sd)	(log10 mean ± sd)	(log10 mean ± sd)
1	0 ± 0	4.97 ± -Inf	1.18 ± -Inf	-Inf ± -Inf	-Inf ± -Inf	-Inf ± 0
2	0 ± 0	4.67 ± -Inf	0.87 ± -Inf	-Inf ± -Inf	-Inf ± -Inf	-Inf ± 0
3	0 ± 0	4.37 ± -Inf	0.54 ± -Inf	-Inf ± -Inf	-Inf ± -Inf	0 ± 0
4	0 ± 0	4.06 ± -Inf	0.21 ± -Inf	-Inf ± -Inf	-Inf ± -Inf	0 ± 0
5	0 ± 0	3.76 ± -Inf	-0.13 ± -Inf	-Inf ± -Inf	-Inf ± -Inf	-Inf ± 0
6	0 ± 0	3.46 ± -Inf	-0.45 ± -Inf	-Inf ± -Inf	-Inf ± -Inf	-Inf ± 0
7	0 ± 0	3.16 ± -Inf	-0.79 ± -Inf	-Inf ± -Inf	-Inf ± -Inf	-Inf ± 0
8	0 ± 0	2.86 ± -Inf	-1.13 ± -Inf	-Inf ± -Inf	-Inf ± -Inf	-Inf ± 0
9	0 ± 0	2.56 ± -Inf	-1.47 ± -Inf	-Inf ± -Inf	-Inf ± -Inf	-Inf ± 0
10	0 ± 0	2.26 ± -Inf	-1.82 ± -Inf	-Inf ± -Inf	-Inf ± -Inf	-Inf ± 0
11	0 ± 0	1.96 ± -Inf	-2.16 ± -Inf	-Inf ± -Inf	-Inf ± -Inf	-Inf ± 0
12	0 ± 0	1.66 ± -Inf	-2.51 ± -Inf	-Inf ± -Inf	-Inf ± -Inf	-Inf ± 0
13	0 ± 0	1.36 ± -Inf	-2.85 ± -Inf	-Inf ± -Inf	-Inf ± -Inf	-Inf ± 0
14	0 ± 0	1.05 ± -Inf	-3.19 ± -Inf	-Inf ± -Inf	-Inf ± -Inf	-Inf ± 0
15	0 ± 0	0.75 ± -Inf	-3.54 ± -Inf	-Inf ± -Inf	-Inf ± -Inf	-Inf ± 0
16	0 ± 0	0.45 ± -Inf	-3.88 ± -Inf	-Inf ± -Inf	-Inf ± -Inf	-Inf ± 0
17	0 ± 0	0.15 ± -Inf	-4.22 ± -Inf	-Inf ± -Inf	-Inf ± -Inf	-Inf ± 0
18	0 ± 0	-0.15 ± -Inf	-4.56 ± -Inf	-Inf ± -Inf	-Inf ± -Inf	-Inf ± 0
19	0 ± 0	-0.45 ± -Inf	-4.9 ± -Inf	-Inf ± -Inf	-Inf ± -Inf	-Inf ± 0
20	0 ± 0	-0.75 ± -Inf	-5.23 ± -Inf	-Inf ± -Inf	-Inf ± -Inf	-Inf ± 0
21	0 ± 0	-1.05 ± -Inf	-5.57 ± -Inf	-Inf ± -Inf	-Inf ± -Inf	-Inf ± 0
22	0 ± 0	-1.35 ± -Inf	-5.9 ± -Inf	-Inf ± -Inf	-Inf ± -Inf	-Inf ± 0
23	0 ± 0	-1.65 ± -Inf	-6.24 ± -Inf	-Inf ± -Inf	-Inf ± -Inf	-Inf ± 0
24	0 ± 0	-1.96 ± -Inf	-6.57 ± -Inf	-Inf ± -Inf	-Inf ± -Inf	-Inf ± 0
25	0 ± 0	-2.26 ± -Inf	-6.9 ± -Inf	-Inf ± -Inf	-Inf ± -Inf	-Inf ± 0
26	0 ± 0	-2.56 ± -Inf	-7.24 ± -Inf	-Inf ± -Inf	-Inf ± -Inf	-Inf ± 0
27	0 ± 0	-2.86 ± -Inf	-7.57 ± -Inf	-Inf ± -Inf	-Inf ± -Inf	-Inf ± 0
28	0 ± 0	-3.16 ± -Inf	-7.9 ± -Inf	-Inf ± -Inf	-Inf ± -Inf	-Inf ± 0
29	0 ± 0	-3.46 ± -Inf	-8.23 ± -Inf	-Inf ± -Inf	-Inf ± -Inf	-Inf ± 0
30	0 ± 0	-3.76 ± -Inf	-8.56 ± -Inf	-Inf ± -Inf	-Inf ± -Inf	-Inf ± 0
31	0 ± 0	-4.06 ± -Inf	-8.88 ± -Inf	-Inf ± -Inf	-Inf ± -Inf	-Inf ± 0
32	0 ± 0	-4.36 ± -Inf	-9.21 ± -Inf	-Inf ± -Inf	-Inf ± -Inf	-Inf ± 0
33	0 ± 0	-4.66 ± -Inf	-9.54 ± -Inf	-Inf ± -Inf	-Inf ± -Inf	-Inf ± 0
34	0 ± 0	-4.97 ± -Inf	-9.87 ± -Inf	-Inf ± -Inf	-Inf ± -Inf	-Inf ± 0
35	0 ± 0	-5.27 ± -Inf	-10.19 ± -Inf	-Inf ± -Inf	-Inf ± -Inf	-Inf ± 0
36	0 ± 0	-5.57 ± -Inf	-10.52 ± -Inf	-Inf ± -Inf	-Inf ± -Inf	-Inf ± 0
37	0 ± 0	-5.87 ± -Inf	-10.84 ± -Inf	-Inf ± -Inf	-Inf ± -Inf	-Inf ± 0

The average of the prevalence, the CFU-values per square meter (cfu/sqm) and per gram manure are calculated over all iterations. The CFU values in the intestine as well as the excreted and ingested quantities are obtained by calculating the average over all animals in one iteration at first and then over all iterations. The decadic logarithm of zero or negative values is negative infinite (-Inf).

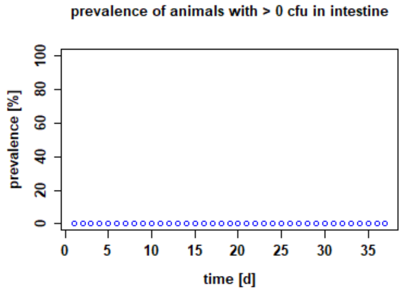

Figure S21. Calculated infection dynamic for the scenario when the pen is contaminated with 10^6 CFU at the beginning of the fattening period, measure 9000 g litter/m^2. Dotplots with results for 90 negative birds, Ross 308, feeding duration 36 days, stocking density 39 kg/m^2, 9000 g litter/m^2 (no infection of birds, thus no plots).

Table S21a (CFU). Calculated numeric values for scenario when the pen is contaminated with 10^6 CFU at the beginning of the fattening period, measure 9000 g litter/m^2. Setting: 90 negative birds, Ross 308, feeding duration 36 days, stocking density 39 kg/m^2, 9000 g litter/m^2

day	prevalence in %	cfu/sqm	cfu/g manure	cfu in intestine	cfu/g feces	cfu ingested per animal
	(mean ± sd)	(mean ± sd)	(mean ± sd)	(mean ± sd)	(mean ± sd)	(mean ± sd)
1	0 ± 0	9.3e4 ± 0e4	1e1 ± 0e1	0e0 ± 0	0e0 ± 0	0e0 ± 0
2	0 ± 0	4.6e4 ± 0e4	5 ± 0	0e0 ± 0	0e0 ± 0	0e0 ± 0
3	0 ± 0	2.3e4 ± 0e4	2.4 ± 0	0e0 ± 0	0e0 ± 0	1 ± 0
4	0 ± 0	1.2e4 ± 0e4	1.1 ± 0	0e0 ± 0	0e0 ± 0	0e0 ± 0
5	0 ± 0	5.8e3 ± 0e3	5.4e-1 ± 0e-1	0e0 ± 0	0e0 ± 0	0e0 ± 0
6	0 ± 0	2.9e3 ± 0e3	2.6e-1 ± 0e-1	0e0 ± 0	0e0 ± 0	0e0 ± 0
7	0 ± 0	1.5e3 ± 0e3	1.2e-1 ± 0e-1	0e0 ± 0	0e0 ± 0	0e0 ± 0
8	0 ± 0	7.3e2 ± 0e2	5.7e-2 ± 0e-2	0e0 ± 0	0e0 ± 0	0e0 ± 0
9	0 ± 0	3.6e2 ± 0e2	2.6e-2 ± 0e-2	0e0 ± 0	0e0 ± 0	0e0 ± 0
10	0 ± 0	1.8e2 ± 0e2	1.2e-2 ± 0e-2	0e0 ± 0	0e0 ± 0	0e0 ± 0
11	0 ± 0	9.1e1 ± 0e1	5.6e-3 ± 0e-3	0e0 ± 0	0e0 ± 0	0e0 ± 0
12	0 ± 0	4.5e1 ± 0e1	2.6e-3 ± 0e-3	0e0 ± 0	0e0 ± 0	0e0 ± 0
13	0 ± 0	2.3e1 ± 0e1	1.2e-3 ± 0e-3	0e0 ± 0	0e0 ± 0	0e0 ± 0
14	0 ± 0	1.1e1 ± 0e1	5.5e-4 ± 0e-4	0e0 ± 0	0e0 ± 0	0e0 ± 0
15	0 ± 0	5.7 ± 0	2.5e-4 ± 0e-4	0e0 ± 0	0e0 ± 0	0e0 ± 0
16	0 ± 0	2.8 ± 0	1.2e-4 ± 0e-4	0e0 ± 0	0e0 ± 0	0e0 ± 0
17	0 ± 0	1.4 ± 0	5.4e-5 ± 0e-5	0e0 ± 0	0e0 ± 0	0e0 ± 0
18	0 ± 0	7.1e-1 ± 0e-1	2.5e-5 ± 0e-5	0e0 ± 0	0e0 ± 0	0e0 ± 0
19	0 ± 0	3.5e-1 ± 0e-1	1.1e-5 ± 0e-5	0e0 ± 0	0e0 ± 0	0e0 ± 0
20	0 ± 0	1.8e-1 ± 0e-1	5.3e-6 ± 0e-6	0e0 ± 0	0e0 ± 0	0e0 ± 0
21	0 ± 0	8.9e-2 ± 0e-2	2.5e-6 ± 0e-6	0e0 ± 0	0e0 ± 0	0e0 ± 0
22	0 ± 0	4.4e-2 ± 0e-2	1.2e-6 ± 0e-6	0e0 ± 0	0e0 ± 0	0e0 ± 0
23	0 ± 0	2.2e-2 ± 0e-2	5.4e-7 ± 0e-7	0e0 ± 0	0e0 ± 0	0e0 ± 0
24	0 ± 0	1.1e-2 ± 0e-2	2.5e-7 ± 0e-7	0e0 ± 0	0e0 ± 0	0e0 ± 0
25	0 ± 0	5.5e-3 ± 0e-3	1.2e-7 ± 0e-7	0e0 ± 0	0e0 ± 0	0e0 ± 0
26	0 ± 0	2.8e-3 ± 0e-3	5.5e-8 ± 0e-8	0e0 ± 0	0e0 ± 0	0e0 ± 0
27	0 ± 0	1.4e-3 ± 0e-3	2.6e-8 ± 0e-8	0e0 ± 0	0e0 ± 0	0e0 ± 0
28	0 ± 0	6.9e-4 ± 0e-4	1.2e-8 ± 0e-8	0e0 ± 0	0e0 ± 0	0e0 ± 0
29	0 ± 0	3.5e-4 ± 0e-4	5.6e-9 ± 0e-9	0e0 ± 0	0e0 ± 0	0e0 ± 0
30	0 ± 0	1.7e-4 ± 0e-4	2.7e-9 ± 0e-9	0e0 ± 0	0e0 ± 0	0e0 ± 0
31	0 ± 0	8.7e-5 ± 0e-5	1.2e-9 ± 0e-9	0e0 ± 0	0e0 ± 0	0e0 ± 0
32	0 ± 0	4.3e-5 ± 0e-5	5.9e-10 ± 0e-10	0e0 ± 0	0e0 ± 0	0e0 ± 0
33	0 ± 0	2.2e-5 ± 0e-5	2.8e-10 ± 0e-10	0e0 ± 0	0e0 ± 0	0e0 ± 0
34	0 ± 0	1.1e-5 ± 0e-5	1.3e-10 ± 0e-10	0e0 ± 0	0e0 ± 0	0e0 ± 0
35	0 ± 0	5.4e-6 ± 0e-6	6.2e-11 ± 0e-11	0e0 ± 0	0e0 ± 0	0e0 ± 0
36	0 ± 0	2.7e-6 ± 0e-6	3e-11 ± 0e-11	0e0 ± 0	0e0 ± 0	0e0 ± 0
37	0 ± 0	1.4e-6 ± 0e-6	1.4e-11 ± 0e-11	0e0 ± 0	0e0 ± 0	0e0 ± 0

The average of the prevalence, the CFU-values per square meter (cfu/sqm) and per gram manure are calculated over all iterations. The CFU values in the intestine as well as the excreted and ingested quantities are obtained by calculating the average over all animals in one iteration at first and then over all iterations.

Table S21b (log_{10} CFU). Calculated numeric values for scenario when the pen is contaminated with 10^6 CFU at the beginning of the fattening period, measure 9000 g litter/m^2. Setting: 90 negative birds, Ross 308, feeding duration 36 days, stocking density 39 kg/m^2, 9000 g litter/m^2

day	prevalence in %	cfu/sqm	cfu/g manure	cfu in intestine	cfu/g feces	cfu ingested per animal
	(% mean ± sd)	(log10 mean ± sd)	(log10 mean ± sd)	(log10 mean ± sd)	(log10 mean ± sd)	(log10 mean ± sd)
1	0 ± 0	4.97 ± -Inf	1.01 ± -Inf	-Inf ± -Inf	-Inf ± -Inf	-Inf ± 0
2	0 ± 0	4.67 ± -Inf	0.7 ± -Inf	-Inf ± -Inf	-Inf ± -Inf	-Inf ± 0
3	0 ± 0	4.37 ± -Inf	0.38 ± -Inf	-Inf ± -Inf	-Inf ± -Inf	0 ± 0
4	0 ± 0	4.06 ± -Inf	0.06 ± -Inf	-Inf ± -Inf	-Inf ± -Inf	-Inf ± 0
5	0 ± 0	3.76 ± -Inf	-0.27 ± -Inf	-Inf ± -Inf	-Inf ± -Inf	-Inf ± 0
6	0 ± 0	3.46 ± -Inf	-0.59 ± -Inf	-Inf ± -Inf	-Inf ± -Inf	-Inf ± 0
7	0 ± 0	3.16 ± -Inf	-0.92 ± -Inf	-Inf ± -Inf	-Inf ± -Inf	-Inf ± 0
8	0 ± 0	2.86 ± -Inf	-1.25 ± -Inf	-Inf ± -Inf	-Inf ± -Inf	-Inf ± 0
9	0 ± 0	2.56 ± -Inf	-1.58 ± -Inf	-Inf ± -Inf	-Inf ± -Inf	-Inf ± 0
10	0 ± 0	2.26 ± -Inf	-1.91 ± -Inf	-Inf ± -Inf	-Inf ± -Inf	-Inf ± 0
11	0 ± 0	1.96 ± -Inf	-2.25 ± -Inf	-Inf ± -Inf	-Inf ± -Inf	-Inf ± 0
12	0 ± 0	1.66 ± -Inf	-2.59 ± -Inf	-Inf ± -Inf	-Inf ± -Inf	-Inf ± 0
13	0 ± 0	1.36 ± -Inf	-2.92 ± -Inf	-Inf ± -Inf	-Inf ± -Inf	-Inf ± 0
14	0 ± 0	1.05 ± -Inf	-3.26 ± -Inf	-Inf ± -Inf	-Inf ± -Inf	-Inf ± 0
15	0 ± 0	0.75 ± -Inf	-3.6 ± -Inf	-Inf ± -Inf	-Inf ± -Inf	-Inf ± 0
16	0 ± 0	0.45 ± -Inf	-3.93 ± -Inf	-Inf ± -Inf	-Inf ± -Inf	-Inf ± 0
17	0 ± 0	0.15 ± -Inf	-4.27 ± -Inf	-Inf ± -Inf	-Inf ± -Inf	-Inf ± 0
18	0 ± 0	-0.15 ± -Inf	-4.61 ± -Inf	-Inf ± -Inf	-Inf ± -Inf	-Inf ± 0
19	0 ± 0	-0.45 ± -Inf	-4.94 ± -Inf	-Inf ± -Inf	-Inf ± -Inf	-Inf ± 0
20	0 ± 0	-0.75 ± -Inf	-5.27 ± -Inf	-Inf ± -Inf	-Inf ± -Inf	-Inf ± 0
21	0 ± 0	-1.05 ± -Inf	-5.61 ± -Inf	-Inf ± -Inf	-Inf ± -Inf	-Inf ± 0
22	0 ± 0	-1.35 ± -Inf	-5.94 ± -Inf	-Inf ± -Inf	-Inf ± -Inf	-Inf ± 0
23	0 ± 0	-1.65 ± -Inf	-6.27 ± -Inf	-Inf ± -Inf	-Inf ± -Inf	-Inf ± 0
24	0 ± 0	-1.96 ± -Inf	-6.6 ± -Inf	-Inf ± -Inf	-Inf ± -Inf	-Inf ± 0
25	0 ± 0	-2.26 ± -Inf	-6.93 ± -Inf	-Inf ± -Inf	-Inf ± -Inf	-Inf ± 0
26	0 ± 0	-2.56 ± -Inf	-7.26 ± -Inf	-Inf ± -Inf	-Inf ± -Inf	-Inf ± 0
27	0 ± 0	-2.86 ± -Inf	-7.59 ± -Inf	-Inf ± -Inf	-Inf ± -Inf	-Inf ± 0
28	0 ± 0	-3.16 ± -Inf	-7.92 ± -Inf	-Inf ± -Inf	-Inf ± -Inf	-Inf ± 0
29	0 ± 0	-3.46 ± -Inf	-8.25 ± -Inf	-Inf ± -Inf	-Inf ± -Inf	-Inf ± 0
30	0 ± 0	-3.76 ± -Inf	-8.58 ± -Inf	-Inf ± -Inf	-Inf ± -Inf	-Inf ± 0
31	0 ± 0	-4.06 ± -Inf	-8.9 ± -Inf	-Inf ± -Inf	-Inf ± -Inf	-Inf ± 0
32	0 ± 0	-4.36 ± -Inf	-9.23 ± -Inf	-Inf ± -Inf	-Inf ± -Inf	-Inf ± 0
33	0 ± 0	-4.66 ± -Inf	-9.56 ± -Inf	-Inf ± -Inf	-Inf ± -Inf	-Inf ± 0
34	0 ± 0	-4.97 ± -Inf	-9.88 ± -Inf	-Inf ± -Inf	-Inf ± -Inf	-Inf ± 0
35	0 ± 0	-5.27 ± -Inf	-10.21 ± -Inf	-Inf ± -Inf	-Inf ± -Inf	-Inf ± 0
36	0 ± 0	-5.57 ± -Inf	-10.53 ± -Inf	-Inf ± -Inf	-Inf ± -Inf	-Inf ± 0
37	0 ± 0	-5.87 ± -Inf	-10.85 ± -Inf	-Inf ± -Inf	-Inf ± -Inf	-Inf ± 0

The average of the prevalence, the CFU-values per square meter (cfu/sqm) and per gram manure are calculated over all iterations. The CFU values in the intestine as well as the excreted and ingested quantities are obtained by calculating the average over all animals in one iteration at first and then over all iterations. The decadic logarithm of zero or negative values is negative infinite (-Inf).

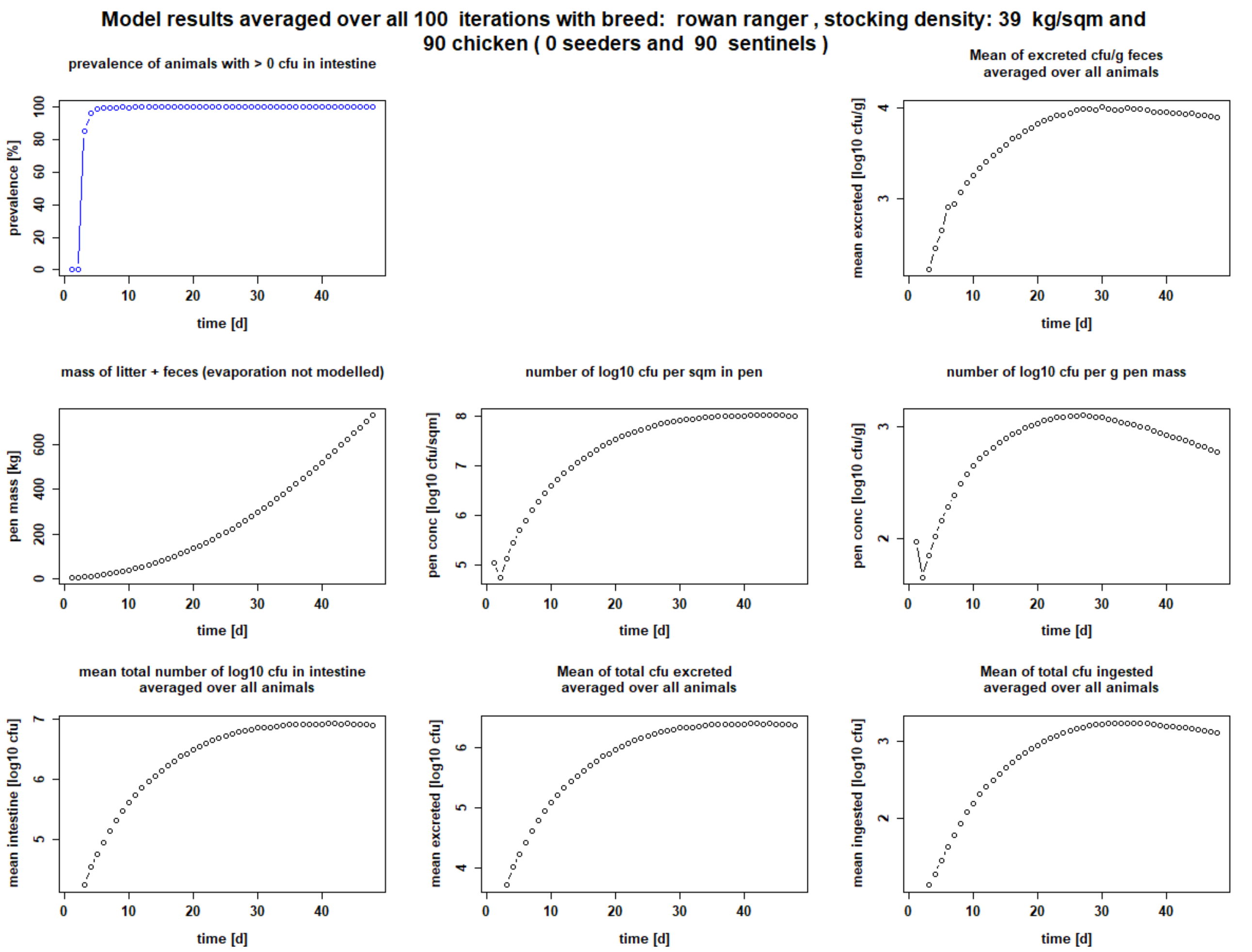

Figure S22. Calculated infection dynamic for the scenario when the pen is contaminated with 10^6 CFU at the beginning of the fattening period, measure slow growing breed. Dotplots with results for 90 negative birds, Rowan x Ranger, feeding duration 47 days, stocking density 39 kg/m^2, 1000 g litter/m^2

Table S22a (CFU). Calculated numeric values for scenario when the pen is contaminated with 106 CFU at the beginning of the fattening period, measure slow growing breed. Setting: 90 negative birds, Rowan x Ranger, feeding duration 47 days, stocking density 39 kg/m^2, 1000 g litter/m^2

day	prevalence in %	cfu/sqm	cfu/g manure	cfu in intestine	cfu/g feces	cfu ingested per animal
	(mean ± sd)	(mean ± sd)	(mean ± sd)	(mean ± sd)	(mean ± sd)	(mean ± sd)
1	0 ± 0	1.1e5 ± 0e5	9.4e1 ± 0e1	0e0 ± 0	0e0 ± 0	0e0 ± 0
2	0 ± 0	5.7e4 ± 0e4	4.5e1 ± 0e1	0e0 ± 0	0e0 ± 0	0e0 ± 0
3	85.04 ± 4.26	1.4e5 ± 0.3e5	7.2e1 ± 1.6e1	1.7e4 ± 0.5e4	1.7e2 ± 0.5e2	1.4e1 ± 0e1
4	96.62 ± 2.15	2.8e5 ± 0.8e5	1.1e2 ± 0.3e2	3.5e4 ± 1.1e4	2.9e2 ± 0.9e2	1.96e1 ± 0.5e1
5	99.19 ± 1	5e5 ± 1.6e5	1.5e2 ± 0.5e2	5.8e4 ± 2.2e4	4.5e2 ± 1.7e2	2.94e1 ± 0.9e1
6	99.89 ± 0.37	8e5 ± 2.8e5	1.9e2 ± 0.7e2	8.9e4 ± 3.6e4	8.2e2 ± 3.3e2	4.42e1 ± 1.5e1
7	99.94 ± 0.33	1.3e6 ± 0.5e6	2.5e2 ± 1e2	1.4e5 ± 0.6e5	8.9e2 ± 3.8e2	6.22e1 ± 2.2e1
8	99.99 ± 0.11	1.9e6 ± 0.8e6	3.1e2 ± 1.3e2	2.1e5 ± 1e5	1.2e3 ± 0.5e3	8.64e1 ± 3.4e1
9	100 ± 0	2.8e6 ± 1.1e6	3.8e2 ± 1.5e2	3e5 ± 1.3e5	1.5e3 ± 0.7e3	1.21e2 ± 0.5e2
10	99.99 ± 0.11	3.9e6 ± 1.7e6	4.5e2 ± 1.9e2	4.1e5 ± 2e5	1.8e3 ± 0.9e3	1.58e2 ± 0.6e2
11	100 ± 0	5.4e6 ± 2.4e6	5.2e2 ± 2.3e2	5.6e5 ± 2.7e5	2.2e3 ± 1e3	2.06e2 ± 0.9e2
12	100 ± 0	7e6 ± 3.1e6	5.9e2 ± 2.6e2	7.1e5 ± 3.4e5	2.6e3 ± 1.2e3	2.55e2 ± 1.1e2
13	100 ± 0	9.1e6 ± 4.4e6	6.6e2 ± 3.2e2	9.1e5 ± 4.9e5	3e3 ± 1.6e3	3.13e2 ± 1.4e2
14	100 ± 0	1.1e7 ± 0.5e7	7.2e2 ± 3.5e2	1.1e6 ± 0.6e6	3.4e3 ± 1.7e3	3.78e2 ± 1.8e2
15	100 ± 0	1.4e7 ± 0.7e7	7.9e2 ± 3.9e2	1.4e6 ± 0.7e6	3.9e3 ± 2.1e3	4.45e2 ± 2.1e2
16	100 ± 0	1.7e7 ± 0.9e7	8.6e2 ± 4.3e2	1.7e6 ± 0.9e6	4.6e3 ± 2.5e3	5.29e2 ± 2.6e2
17	100 ± 0	2.1e7 ± 1.1e7	9.1e2 ± 4.7e2	2e6 ± 1.1e6	4.9e3 ± 2.6e3	6.14e2 ± 3.1e2
18	100 ± 0	2.5e7 ± 1.3e7	9.9e2 ± 5e2	2.4e6 ± 1.3e6	5.6e3 ± 2.9e3	6.9e2 ± 3.5e2
19	100 ± 0	2.9e7 ± 1.5e7	1e3 ± 0.5e3	2.7e6 ± 1.4e6	6e3 ± 3.2e3	8.04e2 ± 4e2
20	100 ± 0	3.3e7 ± 1.7e7	1.1e3 ± 0.5e3	3.1e6 ± 1.6e6	6.7e3 ± 3.5e3	8.84e2 ± 4.5e2
21	100 ± 0	3.8e7 ± 2e7	1.1e3 ± 0.6e3	3.5e6 ± 2.1e6	7.3e3 ± 4.3e3	9.72e2 ± 4.9e2
22	100 ± 0	4.3e7 ± 2.2e7	1.2e3 ± 0.6e3	3.9e6 ± 2.1e6	7.7e3 ± 4.2e3	1.08e3 ± 0.6e3
23	100 ± 0	4.9e7 ± 2.7e7	1.2e3 ± 0.7e3	4.4e6 ± 2.7e6	8.4e3 ± 5.1e3	1.16e3 ± 0.6e3
24	100 ± 0	5.4e7 ± 2.8e7	1.2e3 ± 0.6e3	4.8e6 ± 2.6e6	8.4e3 ± 4.5e3	1.28e3 ± 0.7e3
25	100 ± 0	5.9e7 ± 3e7	1.2e3 ± 0.6e3	5.2e6 ± 2.8e6	8.9e3 ± 4.9e3	1.34e3 ± 0.7e3
26	100 ± 0	6.4e7 ± 3.4e7	1.3e3 ± 0.7e3	5.6e6 ± 3.2e6	9.5e3 ± 5.4e3	1.43e3 ± 0.7e3
27	100 ± 0	7e7 ± 3.8e7	1.3e3 ± 0.7e3	6.2e6 ± 3.6e6	9.9e3 ± 5.8e3	1.49e3 ± 0.8e3
28	100 ± 0	7.4e7 ± 4e7	1.3e3 ± 0.7e3	6.4e6 ± 3.6e6	9.7e3 ± 5.5e3	1.59e3 ± 0.9e3
29	100 ± 0	7.7e7 ± 4.1e7	1.2e3 ± 0.6e3	6.6e6 ± 3.6e6	9.5e3 ± 5.2e3	1.62e3 ± 0.9e3
30	100 ± 0	8.2e7 ± 4e7	1.2e3 ± 0.6e3	7.1e6 ± 3.5e6	1e4 ± 0.5e4	1.63e3 ± 0.9e3
31	100 ± 0	8.5e7 ± 4.2e7	1.2e3 ± 0.6e3	7.1e6 ± 3.8e6	9.7e3 ± 5.2e3	1.7e3 ± 0.8e3
32	100 ± 0	8.7e7 ± 4.4e7	1.1e3 ± 0.6e3	7.3e6 ± 3.9e6	9.6e3 ± 5.1e3	1.69e3 ± 0.8e3
33	100 ± 0	9e7 ± 4.2e7	1.1e3 ± 0.5e3	7.5e6 ± 3.6e6	9.5e3 ± 4.6e3	1.68e3 ± 0.8e3
34	100 ± 0	9.3e7 ± 4.4e7	1.1e3 ± 0.5e3	7.9e6 ± 3.8e6	9.9e3 ± 4.8e3	1.67e3 ± 0.8e3
35	100 ± 0	9.6e7 ± 4.3e7	1.1e3 ± 0.5e3	8e6 ± 3.7e6	9.7e3 ± 4.5e3	1.68e3 ± 0.8e3
36	100 ± 0	9.8e7 ± 4.5e7	1e3 ± 0.5e3	8.1e6 ± 4.2e6	9.7e3 ± 5e3	1.68e3 ± 0.8e3
37	100 ± 0	9.9e7 ± 4.5e7	9.8e2 ± 4.4e2	8.3e6 ± 3.9e6	9.6e3 ± 4.6e3	1.66e3 ± 0.8e3
38	100 ± 0	9.9e7 ± 4.5e7	9.3e2 ± 4.2e2	8.1e6 ± 4e6	9.1e3 ± 4.5e3	1.64e3 ± 0.7e3
39	100 ± 0	9.9e7 ± 4.5e7	8.8e2 ± 4e2	8.1e6 ± 4e6	8.9e3 ± 4.4e3	1.59e3 ± 0.7e3
40	100 ± 0	1e8 ± 0.5e8	8.5e2 ± 3.8e2	8.3e6 ± 3.9e6	9e3 ± 4.3e3	1.55e3 ± 0.7e3
41	100 ± 0	1e8 ± 0.5e8	8.2e2 ± 3.7e2	8.4e6 ± 4.3e6	8.8e3 ± 4.5e3	1.51e3 ± 0.7e3
42	100 ± 0	1e8 ± 0.5e8	8e2 ± 3.7e2	8.6e6 ± 4.3e6	8.9e3 ± 4.4e3	1.49e3 ± 0.7e3
43	100 ± 0	1e8 ± 0.5e8	7.6e2 ± 3.3e2	8.3e6 ± 3.9e6	8.6e3 ± 4.1e3	1.47e3 ± 0.7e3
44	100 ± 0	1e8 ± 0.5e8	7.2e2 ± 3.3e2	8.4e6 ± 4.2e6	8.7e3 ± 4.4e3	1.42e3 ± 0.6e3
45	100 ± 0	1e8 ± 0.5e8	6.9e2 ± 3.1e2	8.2e6 ± 4e6	8.4e3 ± 4.1e3	1.38e3 ± 0.6e3
46	100 ± 0	1e8 ± 0.5e8	6.6e2 ± 3e2	8.3e6 ± 4.3e6	8.3e3 ± 4.2e3	1.33e3 ± 0.6e3
47	100 ± 0	1e8 ± 0.4e8	6.3e2 ± 2.7e2	8.2e6 ± 3.8e6	8.2e3 ± 3.8e3	1.29e3 ± 0.6e3
48	100 ± 0	9.9e7 ± 4.3e7	6e2 ± 2.6e2	7.9e6 ± 3.7e6	7.8e3 ± 3.6e3	1.25e3 ± 0.5e3

The average of the prevalence, the CFU-values per square meter (cfu/sqm) and per gram manure are calculated over all iterations. The CFU values in the intestine as well as the excreted and ingested quantities are obtained by calculating the average over all animals in one iteration at first and then over all iterations.

Table S22b (log$_{10}$ CFU). Calculated numeric values for scenario when the pen is contaminated with 10^6 CFU at the beginning of the fattening period, measure slow growing breed. Setting: 90 negative birds, Rowan x Ranger, feeding duration 47 days, stocking density 39 kg/m^2, 1000 g litter/m^2

day	prevalence in %	cfu/sqm	cfu/g manure	cfu in intestine	cfu/g feces	cfu ingested per animal
	(% mean ± sd)	(log10 mean ± sd)	(log10 mean ± sd)	(log10 mean ± sd)	(log10 mean ± sd)	(log10 mean ± sd)
1	0 ± 0	5.05 ± -Inf	1.97 ± -Inf	-Inf ± -Inf	-Inf ± -Inf	-Inf ± 0
2	0 ± 0	4.75 ± -Inf	1.66 ± -Inf	-Inf ± -Inf	-Inf ± -Inf	-Inf ± 0
3	85.04 ± 4.26	5.13 ± 4.49	1.86 ± 1.22	4.24 ± 3.71	2.22 ± 1.69	1.15 ± 0
4	96.62 ± 2.15	5.45 ± 4.9	2.03 ± 1.48	4.54 ± 4.05	2.46 ± 1.97	1.29 ± 0.67
5	99.19 ± 1	5.7 ± 5.21	2.16 ± 1.68	4.77 ± 4.34	2.65 ± 2.22	1.47 ± 0.93
6	99.89 ± 0.37	5.9 ± 5.45	2.29 ± 1.84	4.95 ± 4.55	2.92 ± 2.51	1.65 ± 1.17
7	99.94 ± 0.33	6.1 ± 5.69	2.39 ± 1.98	5.15 ± 4.78	2.95 ± 2.58	1.79 ± 1.35
8	99.99 ± 0.11	6.28 ± 5.9	2.49 ± 2.12	5.32 ± 4.98	3.07 ± 2.74	1.94 ± 1.53
9	100 ± 0	6.44 ± 6.05	2.58 ± 2.19	5.47 ± 5.11	3.18 ± 2.82	2.08 ± 1.71
10	99.99 ± 0.11	6.59 ± 6.23	2.65 ± 2.29	5.61 ± 5.29	3.27 ± 2.94	2.2 ± 1.81
11	100 ± 0	6.73 ± 6.38	2.72 ± 2.36	5.75 ± 5.43	3.34 ± 3.02	2.31 ± 1.95
12	100 ± 0	6.85 ± 6.5	2.77 ± 2.42	5.85 ± 5.53	3.41 ± 3.09	2.41 ± 2.05
13	100 ± 0	6.96 ± 6.65	2.82 ± 2.51	5.96 ± 5.69	3.48 ± 3.21	2.5 ± 2.15
14	100 ± 0	7.06 ± 6.74	2.86 ± 2.54	6.05 ± 5.75	3.54 ± 3.24	2.58 ± 2.27
15	100 ± 0	7.15 ± 6.84	2.9 ± 2.59	6.14 ± 5.87	3.59 ± 3.33	2.65 ± 2.33
16	100 ± 0	7.24 ± 6.94	2.94 ± 2.64	6.23 ± 5.96	3.66 ± 3.39	2.72 ± 2.42
17	100 ± 0	7.31 ± 7.02	2.96 ± 2.67	6.29 ± 6.02	3.69 ± 3.42	2.79 ± 2.49
18	100 ± 0	7.4 ± 7.1	3 ± 2.7	6.38 ± 6.1	3.75 ± 3.47	2.84 ± 2.55
19	100 ± 0	7.46 ± 7.17	3.02 ± 2.72	6.43 ± 6.16	3.78 ± 3.51	2.91 ± 2.61
20	100 ± 0	7.52 ± 7.22	3.04 ± 2.73	6.49 ± 6.21	3.83 ± 3.55	2.95 ± 2.65
21	100 ± 0	7.58 ± 7.31	3.06 ± 2.78	6.55 ± 6.32	3.86 ± 3.63	2.99 ± 2.69
22	100 ± 0	7.63 ± 7.34	3.07 ± 2.77	6.59 ± 6.32	3.89 ± 3.62	3.03 ± 2.76
23	100 ± 0	7.69 ± 7.43	3.09 ± 2.83	6.65 ± 6.43	3.93 ± 3.71	3.06 ± 2.77
24	100 ± 0	7.73 ± 7.45	3.09 ± 2.81	6.68 ± 6.41	3.92 ± 3.66	3.11 ± 2.85
25	100 ± 0	7.77 ± 7.48	3.09 ± 2.81	6.71 ± 6.45	3.95 ± 3.69	3.13 ± 2.85
26	100 ± 0	7.8 ± 7.53	3.1 ± 2.82	6.75 ± 6.51	3.98 ± 3.73	3.15 ± 2.87
27	100 ± 0	7.84 ± 7.58	3.11 ± 2.84	6.79 ± 6.56	3.99 ± 3.76	3.17 ± 2.9
28	100 ± 0	7.87 ± 7.6	3.1 ± 2.83	6.8 ± 6.56	3.99 ± 3.74	3.2 ± 2.94
29	100 ± 0	7.89 ± 7.61	3.09 ± 2.81	6.82 ± 6.56	3.98 ± 3.71	3.21 ± 2.94
30	100 ± 0	7.92 ± 7.61	3.09 ± 2.78	6.85 ± 6.55	4.01 ± 3.7	3.21 ± 2.93
31	100 ± 0	7.93 ± 7.62	3.07 ± 2.77	6.85 ± 6.58	3.99 ± 3.71	3.23 ± 2.92
32	100 ± 0	7.94 ± 7.64	3.06 ± 2.76	6.87 ± 6.59	3.98 ± 3.71	3.23 ± 2.92
33	100 ± 0	7.95 ± 7.63	3.04 ± 2.72	6.87 ± 6.56	3.98 ± 3.66	3.23 ± 2.92
34	100 ± 0	7.97 ± 7.64	3.03 ± 2.7	6.9 ± 6.59	4 ± 3.68	3.22 ± 2.9
35	100 ± 0	7.98 ± 7.63	3.02 ± 2.67	6.91 ± 6.57	3.99 ± 3.66	3.23 ± 2.9
36	100 ± 0	7.99 ± 7.66	3.01 ± 2.67	6.91 ± 6.62	3.98 ± 3.7	3.22 ± 2.88
37	100 ± 0	8 ± 7.65	2.99 ± 2.65	6.92 ± 6.59	3.98 ± 3.66	3.22 ± 2.89
38	100 ± 0	8 ± 7.66	2.97 ± 2.63	6.91 ± 6.6	3.96 ± 3.65	3.22 ± 2.87
39	100 ± 0	8 ± 7.65	2.95 ± 2.6	6.91 ± 6.6	3.95 ± 3.64	3.2 ± 2.86
40	100 ± 0	8 ± 7.65	2.93 ± 2.58	6.92 ± 6.6	3.95 ± 3.63	3.19 ± 2.85
41	100 ± 0	8.01 ± 7.66	2.91 ± 2.57	6.92 ± 6.63	3.95 ± 3.65	3.18 ± 2.83
42	100 ± 0	8.02 ± 7.68	2.9 ± 2.56	6.94 ± 6.63	3.95 ± 3.64	3.17 ± 2.83
43	100 ± 0	8.01 ± 7.66	2.88 ± 2.52	6.92 ± 6.6	3.93 ± 3.61	3.17 ± 2.83
44	100 ± 0	8.01 ± 7.66	2.86 ± 2.51	6.92 ± 6.62	3.94 ± 3.64	3.15 ± 2.8
45	100 ± 0	8.01 ± 7.66	2.84 ± 2.49	6.91 ± 6.61	3.92 ± 3.61	3.14 ± 2.79
46	100 ± 0	8.01 ± 7.67	2.82 ± 2.48	6.92 ± 6.63	3.92 ± 3.63	3.12 ± 2.78
47	100 ± 0	8 ± 7.64	2.8 ± 2.44	6.91 ± 6.58	3.91 ± 3.58	3.11 ± 2.77
48	100 ± 0	8 ± 7.63	2.78 ± 2.41	6.9 ± 6.56	3.89 ± 3.56	3.1 ± 2.74

The average of the prevalence, the CFU-values per square meter (cfu/sqm) and per gram manure are calculated over all iterations. The CFU values in the intestine as well as the excreted and ingested quantities are obtained by calculating the average over all animals in one iteration at first and then over all iterations. The decadic logarithm of zero or negative values is negative infinite (-Inf).

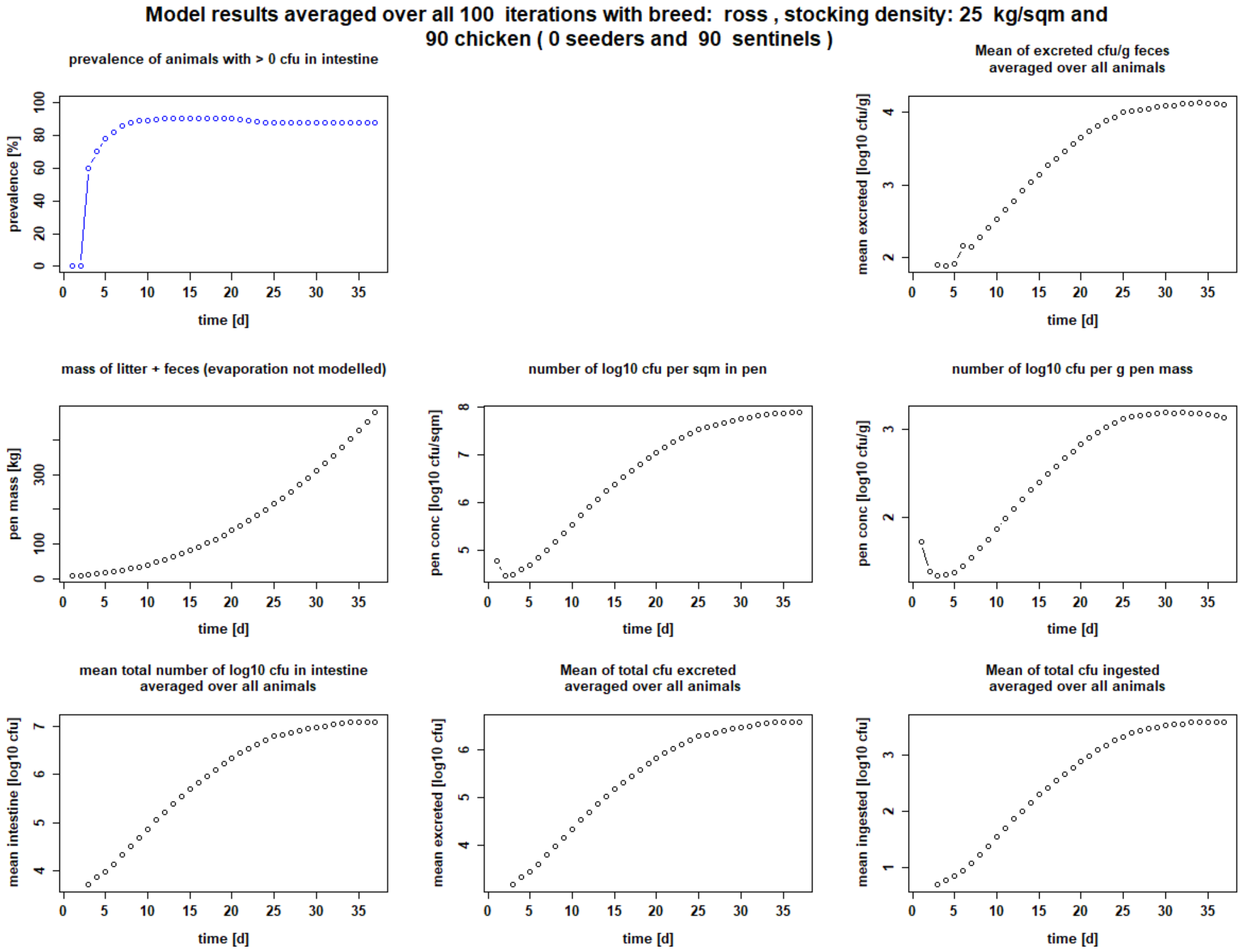

Figure S23. Calculated infection dynamic for the scenario when the pen is contaminated with 10^6 CFU at the beginning of the fattening period, measure stocking density 25 kg/m^2. Dotplots with results for 90 negative birds, Ross 308, feeding duration 36 days, stocking density 25 kg/m^2, 1000 g litter/m^2

Table S23a (CFU). Calculated numeric values for scenario when the pen is contaminated with 10^6 CFU at the beginning of the fattening period, measure stocking density 25 kg/m². Setting: 90 negative birds, Ross 308, feeding duration 36 days, stocking density 25 kg/m², 1000 g litter/m²

day	prevalence in %	cfu/sqm	cfu/g manure	cfu in intestine	cfu/g feces	cfu ingested per animal
	(mean ± sd)	(mean ± sd)	(mean ± sd)	(mean ± sd)	(mean ± sd)	(mean ± sd)
1	0 ± 0	6e4 ± 0e4	5.4e1 ± 0e1	0e0 ± 0	0e0 ± 0	0e0 ± 0
2	0 ± 0	3e4 ± 0e4	2.5e1 ± 0e1	0e0 ± 0	0e0 ± 0	0e0 ± 0
3	60.03 ± 5.12	3.1e4 ± 0.5e4	2.2e1 ± 0.4e1	5.1e3 ± 1.6e3	7.9e1 ± 2.5e1	5 ± 0
4	70.44 ± 7.32	4e4 ± 1.1e4	2.3e1 ± 0.6e1	7.5e3 ± 2.8e3	7.5e1 ± 2.8e1	5.99 ± 1.1
5	78.08 ± 10.54	5e4 ± 1.7e4	2.4e1 ± 0.8e1	9.5e3 ± 4e3	8.1e1 ± 3.4e1	7.2 ± 2
6	82.36 ± 14.53	6.9e4 ± 3.4e4	2.8e1 ± 1.4e1	1.4e4 ± 0.8e4	1.4e2 ± 0.9e2	8.8 ± 3.2
7	85.83 ± 19.88	1e5 ± 0.7e5	3.5e1 ± 2.2e1	2.1e4 ± 1.5e4	1.4e2 ± 1e2	1.22e1 ± 0.6e1
8	87.83 ± 22.43	1.5e5 ± 1.1e5	4.5e1 ± 3.2e1	3.2e4 ± 2.5e4	1.9e2 ± 1.5e2	1.72e1 ± 1.1e1
9	88.96 ± 25.38	2.3e5 ± 1.9e5	5.7e1 ± 4.7e1	4.8e4 ± 4.4e4	2.5e2 ± 2.3e2	2.46e1 ± 1.8e1
10	89.09 ± 28.46	3.5e5 ± 3.1e5	7.4e1 ± 6.5e1	7.4e4 ± 6.8e4	3.3e2 ± 3.1e2	3.53e1 ± 3e1
11	89.86 ± 28.54	5.5e5 ± 5.1e5	9.8e1 ± 9.2e1	1.2e5 ± 1.1e5	4.6e2 ± 4.4e2	5.03e1 ± 4.5e1
12	90.33 ± 28.67	8.2e5 ± 7.5e5	1.3e2 ± 1.2e2	1.7e5 ± 1.6e5	6e2 ± 5.7e2	7.37e1 ± 6.9e1
13	90.4 ± 28.7	1.2e6 ± 1.1e6	1.6e2 ± 1.5e2	2.5e5 ± 2.4e5	8.2e2 ± 8e2	1.01e2 ± 0.9e2
14	90.42 ± 28.71	1.8e6 ± 1.7e6	2.1e2 ± 2e2	3.6e5 ± 3.5e5	1.1e3 ± 1.1e3	1.42e2 ± 1.3e2
15	90.57 ± 28.7	2.5e6 ± 2.5e6	2.5e2 ± 2.6e2	4.9e5 ± 5.2e5	1.4e3 ± 1.5e3	1.98e2 ± 1.9e2
16	90.64 ± 28.71	3.5e6 ± 3.5e6	3.2e2 ± 3.3e2	6.9e5 ± 7.3e5	1.9e3 ± 2e3	2.62e2 ± 2.7e2
17	90.51 ± 28.74	4.7e6 ± 4.5e6	3.9e2 ± 3.7e2	9.2e5 ± 8.8e5	2.3e3 ± 2.2e3	3.52e2 ± 3.6e2
18	90.5 ± 28.76	6.4e6 ± 6.5e6	4.7e2 ± 4.8e2	1.3e6 ± 1.3e6	2.9e3 ± 3.1e3	4.6e2 ± 4.4e2
19	90.46 ± 28.84	8.5e6 ± 8.9e6	5.7e2 ± 5.9e2	1.7e6 ± 1.8e6	3.6e3 ± 3.9e3	5.95e2 ± 6e2
20	90.26 ± 29	1.1e7 ± 1.2e7	6.8e2 ± 7.1e2	2.2e6 ± 2.3e6	4.5e3 ± 4.8e3	7.64e2 ± 7.9e2
21	89.86 ± 29.38	1.5e7 ± 1.5e7	8.1e2 ± 8.4e2	2.8e6 ± 3e6	5.6e3 ± 5.9e3	9.69e2 ± 10.1e2
22	89.39 ± 30.27	1.9e7 ± 1.9e7	9.3e2 ± 9.8e2	3.5e6 ± 3.7e6	6.6e3 ± 7.1e3	1.22e3 ± 1.3e3
23	88.77 ± 31	2.3e7 ± 2.4e7	1.1e3 ± 1.1e3	4.3e6 ± 4.4e6	7.6e3 ± 7.9e3	1.49e3 ± 1.6e3
24	88 ± 32.66	2.8e7 ± 2.7e7	1.2e3 ± 1.2e3	5.1e6 ± 5e6	8.5e3 ± 8.2e3	1.78e3 ± 1.8e3
25	88 ± 32.66	3.4e7 ± 3.2e7	1.3e3 ± 1.3e3	6.3e6 ± 6e6	1e4 ± 1e4	2.07e3 ± 2e3
26	88 ± 32.66	3.8e7 ± 3.5e7	1.4e3 ± 1.3e3	6.7e6 ± 6e6	1e4 ± 0.9e4	2.44e3 ± 2.3e3
27	88 ± 32.66	4.3e7 ± 3.7e7	1.4e3 ± 1.2e3	7.3e6 ± 6.2e6	1.1e4 ± 0.9e4	2.66e3 ± 2.4e3
28	88 ± 32.66	4.7e7 ± 4e7	1.5e3 ± 1.2e3	8.1e6 ± 6.8e6	1.1e4 ± 1e4	2.87e3 ± 2.5e3
29	88 ± 32.66	5.3e7 ± 4.2e7	1.5e3 ± 1.2e3	9.1e6 ± 7.1e6	1.2e4 ± 0.9e4	3.07e3 ± 2.6e3
30	88 ± 32.66	5.8e7 ± 4.4e7	1.6e3 ± 1.2e3	9.7e6 ± 7.4e6	1.3e4 ± 1e4	3.31e3 ± 2.6e3
31	88 ± 32.66	6.1e7 ± 4.3e7	1.5e3 ± 1.1e3	10e6 ± 6.9e6	1.2e4 ± 0.9e4	3.48e3 ± 2.7e3
32	88 ± 32.66	6.7e7 ± 4.6e7	1.6e3 ± 1.1e3	1.1e7 ± 0.8e7	1.3e4 ± 0.9e4	3.55e3 ± 2.5e3
33	88 ± 32.66	7e7 ± 4.6e7	1.5e3 ± 1e3	1.1e7 ± 0.8e7	1.3e4 ± 0.9e4	3.74e3 ± 2.6e3
34	88 ± 32.66	7.3e7 ± 4.7e7	1.5e3 ± 1e3	1.2e7 ± 0.8e7	1.4e4 ± 0.9e4	3.8e3 ± 2.5e3
35	88 ± 32.66	7.6e7 ± 4.7e7	1.5e3 ± 0.9e3	1.2e7 ± 0.8e7	1.3e4 ± 0.8e4	3.86e3 ± 2.5e3
36	88 ± 32.66	7.8e7 ± 4.7e7	1.4e3 ± 0.9e3	1.2e7 ± 0.8e7	1.3e4 ± 0.8e4	3.85e3 ± 2.4e3
37	88 ± 32.66	7.9e7 ± 4.7e7	1.4e3 ± 0.8e3	1.3e7 ± 0.8e7	1.3e4 ± 0.8e4	3.81e3 ± 2.3e3

The average of the prevalence, the CFU-values per square meter (cfu/sqm) and per gram manure are calculated over all iterations. The CFU values in the intestine as well as the excreted and ingested quantities are obtained by calculating the average over all animals in one iteration at first and then over all iterations.

Table S23b (log_{10} CFU). Calculated numeric values for scenario when the pen is contaminated with 10^6 CFU at the beginning of the fattening period, measure stocking density 25 kg/m^2.
Setting: 90 negative birds, Ross 308, feeding duration 36 days, stocking density 25 kg/m^2, 1000 g litter/m^2

day	prevalence in %	cfu/sqm	cfu/g manure	cfu in intestine	cfu/g feces	cfu ingested per animal
	(% mean ± sd)	(log10 mean ± sd)	(log10 mean ± sd)	(log10 mean ± sd)	(log10 mean ± sd)	(log10 mean ± sd)
1	0 ± 0	4.77 ± -Inf	1.73 ± -Inf	-Inf ± -Inf	-Inf ± -Inf	-Inf ± 0
2	0 ± 0	4.47 ± -Inf	1.39 ± -Inf	-Inf ± -Inf	-Inf ± -Inf	-Inf ± 0
3	60.03 ± 5.12	4.5 ± 3.71	1.34 ± 0.56	3.71 ± 3.2	1.9 ± 1.39	0.7 ± 0
4	70.44 ± 7.32	4.6 ± 4.03	1.36 ± 0.78	3.88 ± 3.45	1.87 ± 1.44	0.78 ± 0.04
5	78.08 ± 10.54	4.7 ± 4.23	1.38 ± 0.9	3.98 ± 3.6	1.91 ± 1.53	0.86 ± 0.31
6	82.36 ± 14.53	4.84 ± 4.53	1.45 ± 1.15	4.13 ± 3.91	2.15 ± 1.93	0.94 ± 0.5
7	85.83 ± 19.88	5.01 ± 4.81	1.54 ± 1.35	4.32 ± 4.19	2.14 ± 2.01	1.09 ± 0.8
8	87.83 ± 22.43	5.19 ± 5.04	1.65 ± 1.51	4.51 ± 4.4	2.28 ± 2.17	1.24 ± 1.05
9	88.96 ± 25.38	5.36 ± 5.28	1.75 ± 1.68	4.68 ± 4.64	2.4 ± 2.37	1.39 ± 1.25
10	89.09 ± 28.46	5.55 ± 5.49	1.87 ± 1.81	4.87 ± 4.83	2.52 ± 2.49	1.55 ± 1.48
11	89.86 ± 28.54	5.74 ± 5.71	1.99 ± 1.96	5.06 ± 5.05	2.66 ± 2.65	1.7 ± 1.65
12	90.33 ± 28.67	5.91 ± 5.88	2.1 ± 2.06	5.23 ± 5.2	2.78 ± 2.75	1.87 ± 1.84
13	90.4 ± 28.7	6.08 ± 6.06	2.21 ± 2.18	5.39 ± 5.38	2.92 ± 2.9	2.01 ± 1.97
14	90.42 ± 28.71	6.24 ± 6.23	2.31 ± 2.3	5.55 ± 5.55	3.04 ± 3.03	2.15 ± 2.13
15	90.57 ± 28.7	6.39 ± 6.4	2.4 ± 2.41	5.69 ± 5.72	3.14 ± 3.17	2.3 ± 2.28
16	90.64 ± 28.71	6.54 ± 6.55	2.5 ± 2.51	5.84 ± 5.86	3.27 ± 3.3	2.42 ± 2.43
17	90.51 ± 28.74	6.67 ± 6.65	2.59 ± 2.57	5.97 ± 5.94	3.36 ± 3.34	2.55 ± 2.56
18	90.5 ± 28.76	6.81 ± 6.81	2.67 ± 2.68	6.1 ± 6.13	3.46 ± 3.49	2.66 ± 2.64
19	90.46 ± 28.84	6.93 ± 6.95	2.75 ± 2.77	6.22 ± 6.25	3.56 ± 3.59	2.77 ± 2.78
20	90.26 ± 29	7.05 ± 7.07	2.83 ± 2.85	6.34 ± 6.36	3.66 ± 3.68	2.88 ± 2.9
21	89.86 ± 29.38	7.17 ± 7.19	2.91 ± 2.93	6.45 ± 6.48	3.75 ± 3.77	2.99 ± 3
22	89.39 ± 30.27	7.27 ± 7.29	2.97 ± 2.99	6.54 ± 6.57	3.82 ± 3.85	3.09 ± 3.11
23	88.77 ± 31	7.36 ± 7.37	3.03 ± 3.04	6.63 ± 6.64	3.88 ± 3.9	3.17 ± 3.19
24	88 ± 32.66	7.45 ± 7.44	3.07 ± 3.06	6.71 ± 6.7	3.93 ± 3.91	3.25 ± 3.26
25	88 ± 32.66	7.53 ± 7.51	3.12 ± 3.1	6.8 ± 6.78	4 ± 3.98	3.32 ± 3.31
26	88 ± 32.66	7.59 ± 7.54	3.14 ± 3.1	6.82 ± 6.78	4.02 ± 3.97	3.39 ± 3.37
27	88 ± 32.66	7.63 ± 7.56	3.16 ± 3.09	6.87 ± 6.79	4.03 ± 3.96	3.43 ± 3.38
28	88 ± 32.66	7.68 ± 7.6	3.17 ± 3.09	6.91 ± 6.83	4.05 ± 3.98	3.46 ± 3.39
29	88 ± 32.66	7.72 ± 7.63	3.19 ± 3.09	6.96 ± 6.85	4.08 ± 3.98	3.49 ± 3.41
30	88 ± 32.66	7.76 ± 7.65	3.19 ± 3.08	6.98 ± 6.87	4.1 ± 3.98	3.52 ± 3.42
31	88 ± 32.66	7.78 ± 7.63	3.19 ± 3.04	7 ± 6.84	4.09 ± 3.93	3.54 ± 3.43
32	88 ± 32.66	7.82 ± 7.66	3.2 ± 3.04	7.05 ± 6.9	4.13 ± 3.97	3.55 ± 3.4
33	88 ± 32.66	7.84 ± 7.67	3.19 ± 3.01	7.06 ± 6.88	4.12 ± 3.94	3.57 ± 3.41
34	88 ± 32.66	7.87 ± 7.67	3.19 ± 2.99	7.08 ± 6.89	4.13 ± 3.95	3.58 ± 3.4
35	88 ± 32.66	7.88 ± 7.67	3.17 ± 2.96	7.08 ± 6.88	4.12 ± 3.92	3.59 ± 3.39
36	88 ± 32.66	7.89 ± 7.68	3.16 ± 2.94	7.09 ± 6.89	4.12 ± 3.91	3.59 ± 3.38
37	88 ± 32.66	7.9 ± 7.67	3.14 ± 2.92	7.1 ± 6.88	4.11 ± 3.89	3.58 ± 3.37

The average of the prevalence, the CFU-values per square meter (cfu/sqm) and per gram manure are calculated over all iterations. The CFU values in the intestine as well as the excreted and ingested quantities are obtained by calculating the average over all animals in one iteration at first and then over all iterations. The decadic logarithm of zero or negative values is negative infinite (-Inf).

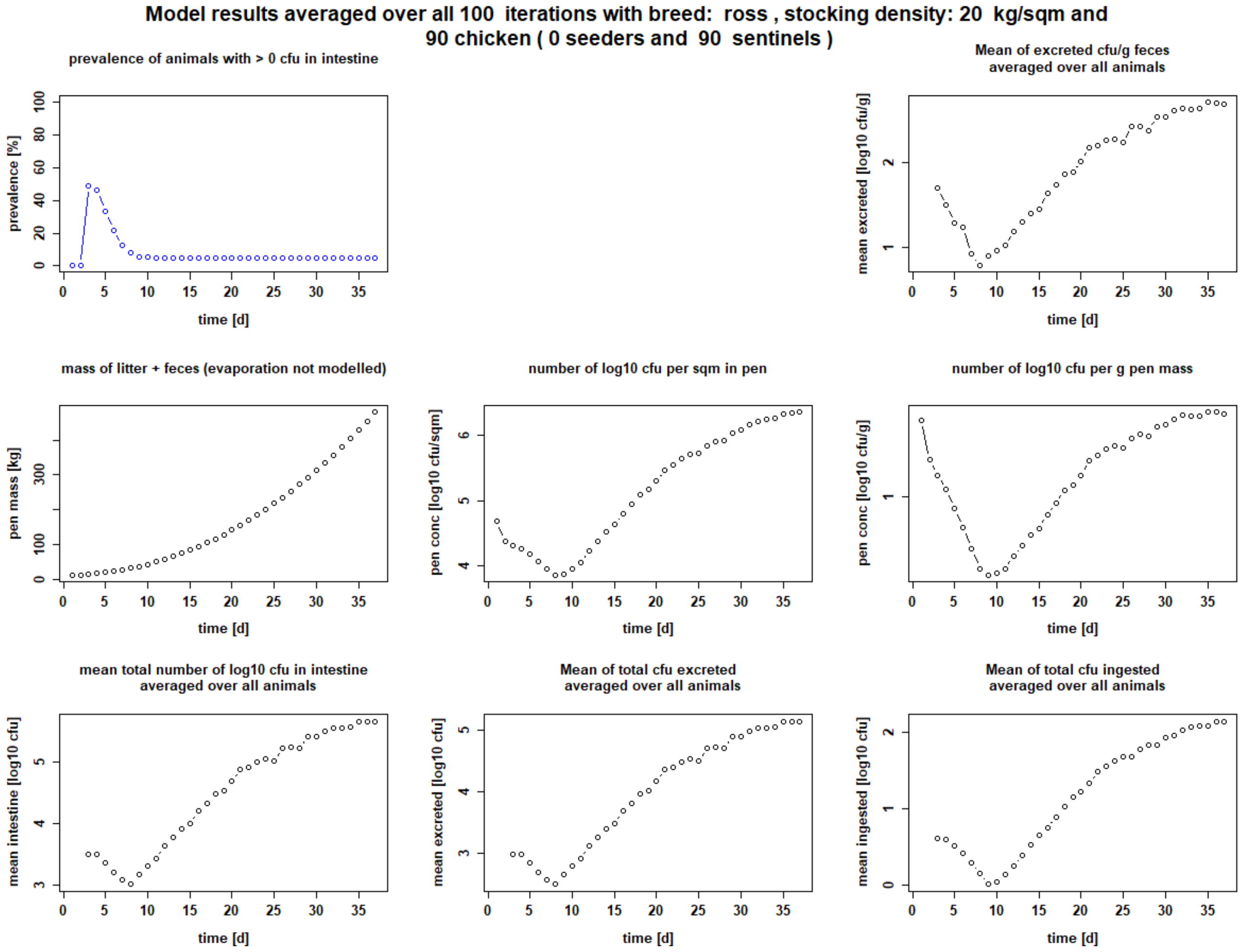

Figure S24. Calculated infection dynamic for the scenario when the pen is contaminated with 10^6 CFU at the beginning of the fattening period, measure stocking density 20 kg/m². Dotplots with results for 90 negative birds, Ross 308, feeding duration 36 days, stocking density 20 kg/m², 1000 g litter/m²

Table S24a (CFU). Calculated numeric values for scenario when the pen is contaminated with 10^6 CFU at the beginning of the fattening period, measure stocking density 20 kg/m². Setting: 90 negative birds, Ross 308, feeding duration 36 days, stocking density 20 kg/m², 1000 g litter/m²

day	prevalence in %	cfu/sqm	cfu/g manure	cfu in intestine	cfu/g feces	cfu ingested per animal
	(mean ± sd)	(mean ± sd)	(mean ± sd)	(mean ± sd)	(mean ± sd)	(mean ± sd)
1	0 ± 0	4.8e4 ± 0e4	4.4e1 ± 0e1	0e0 ± 0	0e0 ± 0	0e0 ± 0
2	0 ± 0	2.4e4 ± 0e4	2e1 ± 0e1	0e0 ± 0	0e0 ± 0	0e0 ± 0
3	49.28 ± 5.21	2e4 ± 0.3e4	1.5e1 ± 0.2e1	3.2e3 ± 1e3	4.9e1 ± 1.5e1	4 ± 0
4	46.44 ± 9.85	1.8e4 ± 0.5e4	1.1e1 ± 0.3e1	3.1e3 ± 1.5e3	3.1e1 ± 1.5e1	3.89 ± 0.6
5	33.54 ± 21.38	1.5e4 ± 0.7e4	7.9 ± 3.7	2.3e3 ± 1.9e3	1.9e1 ± 1.6e1	3.28 ± 1
6	21.68 ± 23.86	1.2e4 ± 1e4	5.4 ± 4.6	1.6e3 ± 2.7e3	1.7e1 ± 2.8e1	2.59 ± 1.4
7	12.39 ± 23.23	9e3 ± 13.4e3	3.6 ± 5.3	1.3e3 ± 3.4e3	8.3 ± 22.5	1.92 ± 2.1
8	8.17 ± 22.24	7.2e3 ± 16.7e3	2.4 ± 5.6	1e3 ± 4e3	6.1 ± 23.6	1.39 ± 2.6
9	5.54 ± 20.99	7.4e3 ± 26.8e3	2.2 ± 7.8	1.5e3 ± 7.3e3	7.9 ± 38.5	1.01 ± 3.1
10	5.24 ± 21.61	8.9e3 ± 39.7e3	2.2 ± 9.9	2e3 ± 10.3e3	9.1 ± 46.1	1.08 ± 4.8
11	4.84 ± 21.23	1.1e4 ± 5.4e4	2.4 ± 11.6	2.7e3 ± 13.4e3	1.1e1 ± 5.3e1	1.36 ± 6.7
12	4.92 ± 21.57	1.7e4 ± 8.4e4	3.1 ± 15.6	4.3e3 ± 22.3e3	1.5e1 ± 8e1	1.74 ± 8.7
13	5 ± 21.9	2.4e4 ± 12.7e4	3.9 ± 20.6	6e3 ± 33.1e3	2e1 ± 11e1	2.45 ± 12.6
14	4.97 ± 21.76	3.3e4 ± 17.8e4	4.7 ± 25.3	8.1e3 ± 44.4e3	2.5e1 ± 13.5e1	3.35 ± 18
15	5 ± 21.9	4.2e4 ± 22.1e4	5.4 ± 27.8	1e4 ± 5.2e4	2.8e1 ± 14.7e1	4.48 ± 24.3
16	5 ± 21.9	6.2e4 ± 33.9e4	7 ± 38.2	1.6e4 ± 9e4	4.3e1 ± 24.5e1	5.52 ± 28.8
17	5 ± 21.9	8.7e4 ± 52.7e4	8.8 ± 53.1	2.2e4 ± 14e4	5.4e1 ± 34.6e1	7.73 ± 42.2
18	5 ± 21.9	1.2e5 ± 7.6e5	1.1e1 ± 6.9e1	3.1e4 ± 19.3e4	7.1e1 ± 44.4e1	1.04e1 ± 6.3e1
19	5 ± 21.9	1.5e5 ± 9.1e5	1.2e1 ± 7.5e1	3.5e4 ± 20.8e4	7.6e1 ± 45.6e1	1.41e1 ± 8.7e1
20	5 ± 21.9	2e5 ± 12.5e5	1.5e1 ± 9.3e1	4.9e4 ± 31.1e4	1e2 ± 6.5e2	1.66e1 ± 10e1
21	5 ± 21.9	3e5 ± 20.5e5	2e1 ± 13.9e1	7.6e4 ± 55.5e4	1.5e2 ± 10.9e2	2.14e1 ± 13.3e1
22	5 ± 21.9	3.6e5 ± 23.9e5	2.2e1 ± 14.8e1	8.2e4 ± 53.2e4	1.6e2 ± 10.1e2	3.04e1 ± 21e1
23	5 ± 21.9	4.4e5 ± 29.7e5	2.5e1 ± 16.9e1	1e5 ± 6.9e5	1.8e2 ± 12.3e2	3.55e1 ± 23.7e1
24	5 ± 21.9	5.1e5 ± 34.4e5	2.7e1 ± 18e1	1.1e5 ± 7.6e5	1.9e2 ± 12.6e2	4.22e1 ± 28.4e1
25	5 ± 21.9	5.3e5 ± 33.5e5	2.5e1 ± 16.2e1	1.1e5 ± 6.4e5	1.7e2 ± 10.2e2	4.69e1 ± 31.6e1
26	5 ± 21.9	7e5 ± 42.4e5	3.1e1 ± 19e1	1.7e5 ± 10e5	2.6e2 ± 15.7e2	4.67e1 ± 29.7e1
27	5 ± 21.9	8.1e5 ± 47.7e5	3.4e1 ± 19.8e1	1.8e5 ± 10.3e5	2.6e2 ± 15.2e2	5.98e1 ± 36.4e1
28	5 ± 21.9	8.3e5 ± 49e5	3.2e1 ± 18.9e1	1.7e5 ± 9.8e5	2.3e2 ± 13.8e2	6.71e1 ± 39.6e1
29	5 ± 21.9	1.1e6 ± 6.3e6	3.9e1 ± 22.5e1	2.6e5 ± 14.9e5	3.4e2 ± 19.7e2	6.72e1 ± 39.4e1
30	5 ± 21.9	1.2e6 ± 6.9e6	4.1e1 ± 23.1e1	2.6e5 ± 14.7e5	3.4e2 ± 19.2e2	8.39e1 ± 48.5e1
31	5 ± 21.9	1.4e6 ± 8.5e6	4.5e1 ± 26.8e1	3.3e5 ± 19.8e5	4.1e2 ± 24.7e2	9.08e1 ± 51.8e1
32	5 ± 21.9	1.6e6 ± 9.5e6	4.8e1 ± 28e1	3.6e5 ± 20.5e5	4.3e2 ± 24.4e2	1.04e2 ± 6.2e2
33	5 ± 21.9	1.7e6 ± 10.2e6	4.8e1 ± 28e1	3.6e5 ± 21.1e5	4.1e2 ± 24.2e2	1.15e2 ± 6.7e2
34	5 ± 21.9	1.8e6 ± 10e6	4.8e1 ± 26e1	3.8e5 ± 19.4e5	4.3e2 ± 21.9e2	1.18e2 ± 6.9e2
35	5 ± 21.9	2.1e6 ± 11.4e6	5.1e1 ± 27.9e1	4.6e5 ± 25.1e5	5e2 ± 27.2e2	1.2e2 ± 6.5e2
36	5 ± 21.9	2.2e6 ± 11.7e6	5.2e1 ± 26.9e1	4.6e5 ± 23.7e5	4.9e2 ± 25.2e2	1.33e2 ± 7.2e2
37	5 ± 21.9	2.3e6 ± 12.2e6	5e1 ± 26.5e1	4.6e5 ± 24.6e5	4.8e2 ± 25.5e2	1.37e2 ± 7.1e2

The average of the prevalence, the CFU-values per square meter (cfu/sqm) and per gram manure are calculated over all iterations. The CFU values in the intestine as well as the excreted and ingested quantities are obtained by calculating the average over all animals in one iteration at first and then over all iterations.

Table S24b (log$_{10}$ CFU). Calculated numeric values for scenario when the pen is contaminated with 10^6 CFU at the beginning of the fattening period, measure stocking density 20 kg/m^2.

Setting: 90 negative birds, Ross 308, feeding duration 36 days, stocking density 20 kg/m^2, 1000 g litter/m^2

day	prevalence in %	cfu/sqm	cfu/g manure	cfu in intestine	cfu/g feces	cfu ingested per animal
	(% mean ± sd)	(log10 mean ± sd)	(log10 mean ± sd)	(log10 mean ± sd)	(log10 mean ± sd)	(log10 mean ± sd)
1	0 ± 0	4.68 ± -Inf	1.64 ± -Inf	-Inf ± -Inf	-Inf ± -Inf	-Inf ± 0
2	0 ± 0	4.38 ± -Inf	1.31 ± -Inf	-Inf ± -Inf	-Inf ± -Inf	-Inf ± 0
3	49.28 ± 5.21	4.3 ± 3.41	1.18 ± 0.28	3.5 ± 3	1.69 ± 1.19	0.6 ± 0
4	46.44 ± 9.85	4.26 ± 3.67	1.05 ± 0.47	3.5 ± 3.17	1.49 ± 1.17	0.59 ± -0.22
5	33.54 ± 21.38	4.17 ± 3.84	0.9 ± 0.56	3.36 ± 3.28	1.29 ± 1.21	0.52 ± 0
6	21.68 ± 23.86	4.07 ± 4	0.74 ± 0.67	3.21 ± 3.43	1.23 ± 1.45	0.41 ± 0.15
7	12.39 ± 23.23	3.96 ± 4.13	0.55 ± 0.72	3.1 ± 3.53	0.92 ± 1.35	0.28 ± 0.32
8	8.17 ± 22.24	3.86 ± 4.22	0.38 ± 0.75	3.01 ± 3.6	0.78 ± 1.37	0.14 ± 0.42
9	5.54 ± 20.99	3.87 ± 4.43	0.33 ± 0.89	3.17 ± 3.86	0.9 ± 1.58	0 ± 0.5
10	5.24 ± 21.61	3.95 ± 4.6	0.35 ± 0.99	3.31 ± 4.01	0.96 ± 1.66	0.03 ± 0.68
11	4.84 ± 21.23	4.06 ± 4.73	0.39 ± 1.06	3.43 ± 4.13	1.02 ± 1.72	0.13 ± 0.83
12	4.92 ± 21.57	4.22 ± 4.93	0.49 ± 1.19	3.63 ± 4.35	1.19 ± 1.9	0.24 ± 0.94
13	5 ± 21.9	4.38 ± 5.1	0.59 ± 1.31	3.78 ± 4.52	1.3 ± 2.04	0.39 ± 1.1
14	4.97 ± 21.76	4.52 ± 5.25	0.67 ± 1.4	3.91 ± 4.65	1.39 ± 2.13	0.53 ± 1.26
15	5 ± 21.9	4.63 ± 5.34	0.73 ± 1.44	4.01 ± 4.72	1.45 ± 2.17	0.65 ± 1.39
16	5 ± 21.9	4.79 ± 5.53	0.85 ± 1.58	4.2 ± 4.96	1.64 ± 2.39	0.74 ± 1.46
17	5 ± 21.9	4.94 ± 5.72	0.94 ± 1.73	4.34 ± 5.15	1.73 ± 2.54	0.89 ± 1.63
18	5 ± 21.9	5.09 ± 5.88	1.05 ± 1.84	4.49 ± 5.29	1.85 ± 2.65	1.02 ± 1.8
19	5 ± 21.9	5.18 ± 5.96	1.09 ± 1.87	4.54 ± 5.32	1.88 ± 2.66	1.15 ± 1.94
20	5 ± 21.9	5.31 ± 6.1	1.18 ± 1.97	4.69 ± 5.49	2.01 ± 2.81	1.22 ± 2
21	5 ± 21.9	5.47 ± 6.31	1.3 ± 2.14	4.88 ± 5.74	2.17 ± 3.04	1.33 ± 2.12
22	5 ± 21.9	5.56 ± 6.38	1.35 ± 2.17	4.91 ± 5.73	2.19 ± 3	1.48 ± 2.32
23	5 ± 21.9	5.64 ± 6.47	1.4 ± 2.23	5.01 ± 5.84	2.26 ± 3.09	1.55 ± 2.37
24	5 ± 21.9	5.71 ± 6.54	1.43 ± 2.26	5.05 ± 5.88	2.27 ± 3.1	1.63 ± 2.45
25	5 ± 21.9	5.72 ± 6.53	1.41 ± 2.21	5.02 ± 5.81	2.23 ± 3.01	1.67 ± 2.5
26	5 ± 21.9	5.84 ± 6.63	1.49 ± 2.28	5.23 ± 6	2.42 ± 3.2	1.67 ± 2.47
27	5 ± 21.9	5.91 ± 6.68	1.53 ± 2.3	5.25 ± 6.01	2.42 ± 3.18	1.78 ± 2.56
28	5 ± 21.9	5.92 ± 6.69	1.51 ± 2.28	5.22 ± 5.99	2.37 ± 3.14	1.83 ± 2.6
29	5 ± 21.9	6.04 ± 6.8	1.59 ± 2.35	5.41 ± 6.17	2.54 ± 3.29	1.83 ± 2.6
30	5 ± 21.9	6.08 ± 6.84	1.61 ± 2.36	5.41 ± 6.17	2.53 ± 3.28	1.92 ± 2.69
31	5 ± 21.9	6.16 ± 6.93	1.66 ± 2.43	5.51 ± 6.3	2.61 ± 3.39	1.96 ± 2.71
32	5 ± 21.9	6.22 ± 6.98	1.68 ± 2.45	5.56 ± 6.31	2.63 ± 3.39	2.02 ± 2.79
33	5 ± 21.9	6.24 ± 7.01	1.68 ± 2.45	5.55 ± 6.32	2.61 ± 3.38	2.06 ± 2.82
34	5 ± 21.9	6.26 ± 7	1.68 ± 2.41	5.58 ± 6.29	2.63 ± 3.34	2.07 ± 2.84
35	5 ± 21.9	6.32 ± 7.06	1.71 ± 2.45	5.66 ± 6.4	2.7 ± 3.43	2.08 ± 2.82
36	5 ± 21.9	6.35 ± 7.07	1.71 ± 2.43	5.67 ± 6.38	2.69 ± 3.4	2.12 ± 2.86
37	5 ± 21.9	6.36 ± 7.08	1.7 ± 2.42	5.66 ± 6.39	2.68 ± 3.41	2.14 ± 2.85

The average of the prevalence, the CFU-values per square meter (cfu/sqm) and per gram manure are calculated over all iterations. The CFU values in the intestine as well as the excreted and ingested quantities are obtained by calculating the average over all animals in one iteration at first and then over all iterations. The decadic logarithm of zero or negative values is negative infinite (-Inf).

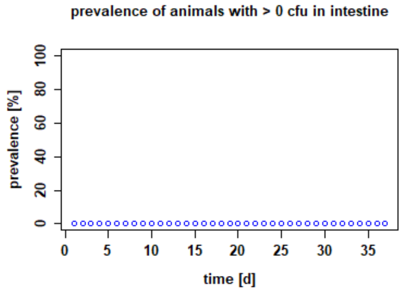

Figure S25. Calculated infection dynamic for the scenario when the pen is contaminated with 10^6 CFU at the beginning of the fattening period, combined measure stocking density 25 kg/m^2 and 3000 g litter/m^2. Dotplots with results for 90 negative birds, Ross 308, feeding duration 36 days, stocking density 25 kg/m^2, 3000 g litter/m^2 (no infection of birds, thus no plots).

Table S25a (CFU). Calculated numeric values for scenario when the pen is contaminated with 10^6 CFU at the beginning of the fattening period, combined measure stocking density 25 kg/m^2 and 3000 g litter/m^2. Setting: 90 negative birds, Ross 308, feeding duration 36 days, stocking density 25 kg/m^2, 3000 g litter/m^2

day	prevalence in %	cfu/sqm	cfu/g manure	cfu in intestine	cfu/g feces	cfu ingested per animal
	(mean ± sd)	(mean ± sd)	(mean ± sd)	(mean ± sd)	(mean ± sd)	(mean ± sd)
1	0 ± 0	6e4 ± 0e4	1.9e1 ± 0e1	0e0 ± 0	0e0 ± 0	0e0 ± 0
2	0 ± 0	3e4 ± 0e4	9.3 ± 0	0e0 ± 0	0e0 ± 0	0e0 ± 0
3	0 ± 0	1.5e4 ± 0e4	4.4 ± 0	0e0 ± 0	0e0 ± 0	2 ± 0
4	0 ± 0	7.4e3 ± 0e3	2 ± 0	0e0 ± 0	0e0 ± 0	1 ± 0
5	0 ± 0	3.7e3 ± 0e3	9e-1 ± 0e-1	0e0 ± 0	0e0 ± 0	0e0 ± 0
6	0 ± 0	1.9e3 ± 0e3	4.2e-1 ± 0e-1	0e0 ± 0	0e0 ± 0	0e0 ± 0
7	0 ± 0	9.3e2 ± 0e2	1.9e-1 ± 0e-1	0e0 ± 0	0e0 ± 0	0e0 ± 0
8	0 ± 0	4.7e2 ± 0e2	8.5e-2 ± 0e-2	0e0 ± 0	0e0 ± 0	0e0 ± 0
9	0 ± 0	2.3e2 ± 0e2	3.8e-2 ± 0e-2	0e0 ± 0	0e0 ± 0	0e0 ± 0
10	0 ± 0	1.2e2 ± 0e2	1.7e-2 ± 0e-2	0e0 ± 0	0e0 ± 0	0e0 ± 0
11	0 ± 0	5.8e1 ± 0e1	7.7e-3 ± 0e-3	0e0 ± 0	0e0 ± 0	0e0 ± 0
12	0 ± 0	2.9e1 ± 0e1	3.4e-3 ± 0e-3	0e0 ± 0	0e0 ± 0	0e0 ± 0
13	0 ± 0	1.5e1 ± 0e1	1.5e-3 ± 0e-3	0e0 ± 0	0e0 ± 0	0e0 ± 0
14	0 ± 0	7.3 ± 0	6.9e-4 ± 0e-4	0e0 ± 0	0e0 ± 0	0e0 ± 0
15	0 ± 0	3.6 ± 0	3.1e-4 ± 0e-4	0e0 ± 0	0e0 ± 0	0e0 ± 0
16	0 ± 0	1.8 ± 0	1.4e-4 ± 0e-4	0e0 ± 0	0e0 ± 0	0e0 ± 0
17	0 ± 0	9.1e-1 ± 0e-1	6.4e-5 ± 0e-5	0e0 ± 0	0e0 ± 0	0e0 ± 0
18	0 ± 0	4.5e-1 ± 0e-1	2.9e-5 ± 0e-5	0e0 ± 0	0e0 ± 0	0e0 ± 0
19	0 ± 0	2.3e-1 ± 0e-1	1.3e-5 ± 0e-5	0e0 ± 0	0e0 ± 0	0e0 ± 0
20	0 ± 0	1.1e-1 ± 0e-1	6.1e-6 ± 0e-6	0e0 ± 0	0e0 ± 0	0e0 ± 0
21	0 ± 0	5.7e-2 ± 0e-2	2.8e-6 ± 0e-6	0e0 ± 0	0e0 ± 0	0e0 ± 0
22	0 ± 0	2.8e-2 ± 0e-2	1.3e-6 ± 0e-6	0e0 ± 0	0e0 ± 0	0e0 ± 0
23	0 ± 0	1.4e-2 ± 0e-2	6e-7 ± 0e-7	0e0 ± 0	0e0 ± 0	0e0 ± 0
24	0 ± 0	7.1e-3 ± 0e-3	2.8e-7 ± 0e-7	0e0 ± 0	0e0 ± 0	0e0 ± 0
25	0 ± 0	3.5e-3 ± 0e-3	1.3e-7 ± 0e-7	0e0 ± 0	0e0 ± 0	0e0 ± 0
26	0 ± 0	1.8e-3 ± 0e-3	6e-8 ± 0e-8	0e0 ± 0	0e0 ± 0	0e0 ± 0
27	0 ± 0	8.9e-4 ± 0e-4	2.8e-8 ± 0e-8	0e0 ± 0	0e0 ± 0	0e0 ± 0
28	0 ± 0	4.4e-4 ± 0e-4	1.3e-8 ± 0e-8	0e0 ± 0	0e0 ± 0	0e0 ± 0
29	0 ± 0	2.2e-4 ± 0e-4	6.1e-9 ± 0e-9	0e0 ± 0	0e0 ± 0	0e0 ± 0
30	0 ± 0	1.1e-4 ± 0e-4	2.8e-9 ± 0e-9	0e0 ± 0	0e0 ± 0	0e0 ± 0
31	0 ± 0	5.5e-5 ± 0e-5	1.3e-9 ± 0e-9	0e0 ± 0	0e0 ± 0	0e0 ± 0
32	0 ± 0	2.8e-5 ± 0e-5	6.3e-10 ± 0e-10	0e0 ± 0	0e0 ± 0	0e0 ± 0
33	0 ± 0	1.4e-5 ± 0e-5	2.9e-10 ± 0e-10	0e0 ± 0	0e0 ± 0	0e0 ± 0
34	0 ± 0	6.9e-6 ± 0e-6	1.4e-10 ± 0e-10	0e0 ± 0	0e0 ± 0	0e0 ± 0
35	0 ± 0	3.5e-6 ± 0e-6	6.5e-11 ± 0e-11	0e0 ± 0	0e0 ± 0	0e0 ± 0
36	0 ± 0	1.7e-6 ± 0e-6	3.1e-11 ± 0e-11	0e0 ± 0	0e0 ± 0	0e0 ± 0
37	0 ± 0	8.7e-7 ± 0e-7	1.5e-11 ± 0e-11	0e0 ± 0	0e0 ± 0	0e0 ± 0

The average of the prevalence, the CFU-values per square meter (cfu/sqm) and per gram manure are calculated over all iterations. The CFU values in the intestine as well as the excreted and ingested quantities are obtained by calculating the average over all animals in one iteration at first and then over all iterations.

Table S25b (log_{10} CFU). Calculated numeric values for scenario when the pen is contaminated with 10^6 CFU at the beginning of the fattening period, combined measure stocking density 25 kg/m^2 and 3000 g litter/m^2. Setting: 90 negative birds, Ross 308, feeding duration 36 days, stocking density 25 kg/m^2, 3000 g litter/m^2

day	prevalence in %	cfu/sqm	cfu/g manure	cfu in intestine	cfu/g feces	cfu ingested per animal
	(% mean ± sd)	(log10 mean ± sd)	(log10 mean ± sd)	(log10 mean ± sd)	(log10 mean ± sd)	(log10 mean ± sd)
1	0 ± 0	4.77 ± -Inf	1.28 ± -Inf	-Inf ± -Inf	-Inf ± -Inf	-Inf ± 0
2	0 ± 0	4.47 ± -Inf	0.97 ± -Inf	-Inf ± -Inf	-Inf ± -Inf	-Inf ± 0
3	0 ± 0	4.17 ± -Inf	0.64 ± -Inf	-Inf ± -Inf	-Inf ± -Inf	0.3 ± 0
4	0 ± 0	3.87 ± -Inf	0.3 ± -Inf	-Inf ± -Inf	-Inf ± -Inf	0 ± 0
5	0 ± 0	3.57 ± -Inf	-0.04 ± -Inf	-Inf ± -Inf	-Inf ± -Inf	-Inf ± 0
6	0 ± 0	3.27 ± -Inf	-0.38 ± -Inf	-Inf ± -Inf	-Inf ± -Inf	-Inf ± 0
7	0 ± 0	2.97 ± -Inf	-0.72 ± -Inf	-Inf ± -Inf	-Inf ± -Inf	-Inf ± 0
8	0 ± 0	2.67 ± -Inf	-1.07 ± -Inf	-Inf ± -Inf	-Inf ± -Inf	-Inf ± 0
9	0 ± 0	2.37 ± -Inf	-1.42 ± -Inf	-Inf ± -Inf	-Inf ± -Inf	-Inf ± 0
10	0 ± 0	2.07 ± -Inf	-1.77 ± -Inf	-Inf ± -Inf	-Inf ± -Inf	-Inf ± 0
11	0 ± 0	1.76 ± -Inf	-2.12 ± -Inf	-Inf ± -Inf	-Inf ± -Inf	-Inf ± 0
12	0 ± 0	1.46 ± -Inf	-2.47 ± -Inf	-Inf ± -Inf	-Inf ± -Inf	-Inf ± 0
13	0 ± 0	1.16 ± -Inf	-2.81 ± -Inf	-Inf ± -Inf	-Inf ± -Inf	-Inf ± 0
14	0 ± 0	0.86 ± -Inf	-3.16 ± -Inf	-Inf ± -Inf	-Inf ± -Inf	-Inf ± 0
15	0 ± 0	0.56 ± -Inf	-3.51 ± -Inf	-Inf ± -Inf	-Inf ± -Inf	-Inf ± 0
16	0 ± 0	0.26 ± -Inf	-3.85 ± -Inf	-Inf ± -Inf	-Inf ± -Inf	-Inf ± 0
17	0 ± 0	-0.04 ± -Inf	-4.19 ± -Inf	-Inf ± -Inf	-Inf ± -Inf	-Inf ± 0
18	0 ± 0	-0.34 ± -Inf	-4.53 ± -Inf	-Inf ± -Inf	-Inf ± -Inf	-Inf ± 0
19	0 ± 0	-0.64 ± -Inf	-4.87 ± -Inf	-Inf ± -Inf	-Inf ± -Inf	-Inf ± 0
20	0 ± 0	-0.94 ± -Inf	-5.21 ± -Inf	-Inf ± -Inf	-Inf ± -Inf	-Inf ± 0
21	0 ± 0	-1.25 ± -Inf	-5.55 ± -Inf	-Inf ± -Inf	-Inf ± -Inf	-Inf ± 0
22	0 ± 0	-1.55 ± -Inf	-5.89 ± -Inf	-Inf ± -Inf	-Inf ± -Inf	-Inf ± 0
23	0 ± 0	-1.85 ± -Inf	-6.22 ± -Inf	-Inf ± -Inf	-Inf ± -Inf	-Inf ± 0
24	0 ± 0	-2.15 ± -Inf	-6.56 ± -Inf	-Inf ± -Inf	-Inf ± -Inf	-Inf ± 0
25	0 ± 0	-2.45 ± -Inf	-6.89 ± -Inf	-Inf ± -Inf	-Inf ± -Inf	-Inf ± 0
26	0 ± 0	-2.75 ± -Inf	-7.22 ± -Inf	-Inf ± -Inf	-Inf ± -Inf	-Inf ± 0
27	0 ± 0	-3.05 ± -Inf	-7.56 ± -Inf	-Inf ± -Inf	-Inf ± -Inf	-Inf ± 0
28	0 ± 0	-3.35 ± -Inf	-7.89 ± -Inf	-Inf ± -Inf	-Inf ± -Inf	-Inf ± 0
29	0 ± 0	-3.65 ± -Inf	-8.22 ± -Inf	-Inf ± -Inf	-Inf ± -Inf	-Inf ± 0
30	0 ± 0	-3.95 ± -Inf	-8.55 ± -Inf	-Inf ± -Inf	-Inf ± -Inf	-Inf ± 0
31	0 ± 0	-4.26 ± -Inf	-8.88 ± -Inf	-Inf ± -Inf	-Inf ± -Inf	-Inf ± 0
32	0 ± 0	-4.56 ± -Inf	-9.2 ± -Inf	-Inf ± -Inf	-Inf ± -Inf	-Inf ± 0
33	0 ± 0	-4.86 ± -Inf	-9.53 ± -Inf	-Inf ± -Inf	-Inf ± -Inf	-Inf ± 0
34	0 ± 0	-5.16 ± -Inf	-9.86 ± -Inf	-Inf ± -Inf	-Inf ± -Inf	-Inf ± 0
35	0 ± 0	-5.46 ± -Inf	-10.18 ± -Inf	-Inf ± -Inf	-Inf ± -Inf	-Inf ± 0
36	0 ± 0	-5.76 ± -Inf	-10.51 ± -Inf	-Inf ± -Inf	-Inf ± -Inf	-Inf ± 0
37	0 ± 0	-6.06 ± -Inf	-10.83 ± -Inf	-Inf ± -Inf	-Inf ± -Inf	-Inf ± 0

The average of the prevalence, the CFU-values per square meter (cfu/sqm) and per gram manure are calculated over all iterations. The CFU values in the intestine as well as the excreted and ingested quantities are obtained by calculating the average over all animals in one iteration at first and then over all iterations. The decadic logarithm of zero or negative values is negative infinite (-Inf).

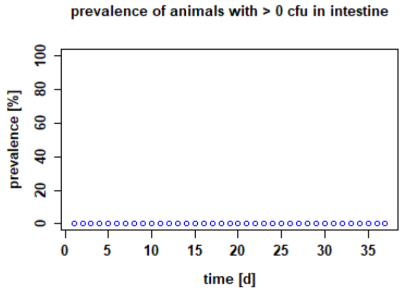

Figure S26. Calculated infection dynamic for the scenario when the pen is contaminated with 10^6 CFU at the beginning of the fattening period, combined measure stocking density 20 kg/m^2 and 3000 g litter/m^2. Dotplots with results for 90 negative birds, Ross 308, feeding duration 36 days, stocking density 20 kg/m^2, 3000 g litter/m^2 (no infection of birds, thus no plots).

Table S26a (CFU). Calculated numeric values for scenario when the pen is contaminated with 10^6 CFU at the beginning of the fattening period, combined measure stocking density 20 kg/m^2 and 3000 g litter/m^2. Setting: 90 negative birds, Ross 308, feeding duration 36 days, stocking density 20 kg/m^2, 3000 g litter/m^2

day	prevalence in %	cfu/sqm	cfu/g manure	cfu in intestine	cfu/g feces	cfu ingested per animal
	(mean ± sd)	(mean ± sd)	(mean ± sd)	(mean ± sd)	(mean ± sd)	(mean ± sd)
1	0 ± 0	4.8e4 ± 0e4	1.5e1 ± 0e1	0e0 ± 0	0e0 ± 0	0e0 ± 0
2	0 ± 0	2.4e4 ± 0e4	7.5 ± 0	0e0 ± 0	0e0 ± 0	0e0 ± 0
3	0 ± 0	1.2e4 ± 0e4	3.6 ± 0	0e0 ± 0	0e0 ± 0	1 ± 0
4	0 ± 0	6e3 ± 0e3	1.7 ± 0	0e0 ± 0	0e0 ± 0	1 ± 0
5	0 ± 0	3e3 ± 0e3	7.6e-1 ± 0e-1	0e0 ± 0	0e0 ± 0	0e0 ± 0
6	0 ± 0	1.5e3 ± 0e3	3.6e-1 ± 0e-1	0e0 ± 0	0e0 ± 0	0e0 ± 0
7	0 ± 0	7.4e2 ± 0e2	1.6e-1 ± 0e-1	0e0 ± 0	0e0 ± 0	0e0 ± 0
8	0 ± 0	3.7e2 ± 0e2	7.5e-2 ± 0e-2	0e0 ± 0	0e0 ± 0	0e0 ± 0
9	0 ± 0	1.9e2 ± 0e2	3.4e-2 ± 0e-2	0e0 ± 0	0e0 ± 0	0e0 ± 0
10	0 ± 0	9.3e1 ± 0e1	1.5e-2 ± 0e-2	0e0 ± 0	0e0 ± 0	0e0 ± 0
11	0 ± 0	4.7e1 ± 0e1	7e-3 ± 0e-3	0e0 ± 0	0e0 ± 0	0e0 ± 0
12	0 ± 0	2.3e1 ± 0e1	3.1e-3 ± 0e-3	0e0 ± 0	0e0 ± 0	0e0 ± 0
13	0 ± 0	1.2e1 ± 0e1	1.4e-3 ± 0e-3	0e0 ± 0	0e0 ± 0	0e0 ± 0
14	0 ± 0	5.8 ± 0	6.5e-4 ± 0e-4	0e0 ± 0	0e0 ± 0	0e0 ± 0
15	0 ± 0	2.9 ± 0	2.9e-4 ± 0e-4	0e0 ± 0	0e0 ± 0	0e0 ± 0
16	0 ± 0	1.5 ± 0	1.3e-4 ± 0e-4	0e0 ± 0	0e0 ± 0	0e0 ± 0
17	0 ± 0	7.3e-1 ± 0e-1	6.1e-5 ± 0e-5	0e0 ± 0	0e0 ± 0	0e0 ± 0
18	0 ± 0	3.6e-1 ± 0e-1	2.8e-5 ± 0e-5	0e0 ± 0	0e0 ± 0	0e0 ± 0
19	0 ± 0	1.8e-1 ± 0e-1	1.3e-5 ± 0e-5	0e0 ± 0	0e0 ± 0	0e0 ± 0
20	0 ± 0	9.1e-2 ± 0e-2	5.9e-6 ± 0e-6	0e0 ± 0	0e0 ± 0	0e0 ± 0
21	0 ± 0	4.5e-2 ± 0e-2	2.7e-6 ± 0e-6	0e0 ± 0	0e0 ± 0	0e0 ± 0
22	0 ± 0	2.3e-2 ± 0e-2	1.3e-6 ± 0e-6	0e0 ± 0	0e0 ± 0	0e0 ± 0
23	0 ± 0	1.1e-2 ± 0e-2	5.8e-7 ± 0e-7	0e0 ± 0	0e0 ± 0	0e0 ± 0
24	0 ± 0	5.7e-3 ± 0e-3	2.7e-7 ± 0e-7	0e0 ± 0	0e0 ± 0	0e0 ± 0
25	0 ± 0	2.8e-3 ± 0e-3	1.3e-7 ± 0e-7	0e0 ± 0	0e0 ± 0	0e0 ± 0
26	0 ± 0	1.4e-3 ± 0e-3	5.8e-8 ± 0e-8	0e0 ± 0	0e0 ± 0	0e0 ± 0
27	0 ± 0	7.1e-4 ± 0e-4	2.7e-8 ± 0e-8	0e0 ± 0	0e0 ± 0	0e0 ± 0
28	0 ± 0	3.5e-4 ± 0e-4	1.3e-8 ± 0e-8	0e0 ± 0	0e0 ± 0	0e0 ± 0
29	0 ± 0	1.8e-4 ± 0e-4	5.9e-9 ± 0e-9	0e0 ± 0	0e0 ± 0	0e0 ± 0
30	0 ± 0	8.9e-5 ± 0e-5	2.8e-9 ± 0e-9	0e0 ± 0	0e0 ± 0	0e0 ± 0
31	0 ± 0	4.4e-5 ± 0e-5	1.3e-9 ± 0e-9	0e0 ± 0	0e0 ± 0	0e0 ± 0
32	0 ± 0	2.2e-5 ± 0e-5	6.2e-10 ± 0e-10	0e0 ± 0	0e0 ± 0	0e0 ± 0
33	0 ± 0	1.1e-5 ± 0e-5	2.9e-10 ± 0e-10	0e0 ± 0	0e0 ± 0	0e0 ± 0
34	0 ± 0	5.5e-6 ± 0e-6	1.4e-10 ± 0e-10	0e0 ± 0	0e0 ± 0	0e0 ± 0
35	0 ± 0	2.8e-6 ± 0e-6	6.5e-11 ± 0e-11	0e0 ± 0	0e0 ± 0	0e0 ± 0
36	0 ± 0	1.4e-6 ± 0e-6	3.1e-11 ± 0e-11	0e0 ± 0	0e0 ± 0	0e0 ± 0
37	0 ± 0	6.9e-7 ± 0e-7	1.4e-11 ± 0e-11	0e0 ± 0	0e0 ± 0	0e0 ± 0

The average of the prevalence, the CFU-values per square meter (cfu/sqm) and per gram manure are calculated over all iterations. The CFU values in the intestine as well as the excreted and ingested quantities are obtained by calculating the average over all animals in one iteration at first and then over all iterations.

Table S26b (log_{10} CFU). Calculated numeric values for scenario when the pen is contaminated with 10^6 CFU at the beginning of the fattening period, combined measure stocking density 20 kg/m^2 and 3000 g litter/m^2. Setting: 90 negative birds, Ross 308, feeding duration 36 days, stocking density 20 kg/m^2, 3000 g litter/m^2

day	prevalence in %	cfu/sqm	cfu/g manure	cfu in intestine	cfu/g feces	cfu ingested per animal
	(% mean ± sd)	(log10 mean ± sd)	(log10 mean ± sd)	(log10 mean ± sd)	(log10 mean ± sd)	(log10 mean ± sd)
1	0 ± 0	4.68 ± -Inf	1.19 ± -Inf	-Inf ± -Inf	-Inf ± -Inf	-Inf ± 0
2	0 ± 0	4.38 ± -Inf	0.88 ± -Inf	-Inf ± -Inf	-Inf ± -Inf	-Inf ± 0
3	0 ± 0	4.08 ± -Inf	0.55 ± -Inf	-Inf ± -Inf	-Inf ± -Inf	0 ± 0
4	0 ± 0	3.77 ± -Inf	0.22 ± -Inf	-Inf ± -Inf	-Inf ± -Inf	0 ± 0
5	0 ± 0	3.47 ± -Inf	-0.12 ± -Inf	-Inf ± -Inf	-Inf ± -Inf	-Inf ± 0
6	0 ± 0	3.17 ± -Inf	-0.44 ± -Inf	-Inf ± -Inf	-Inf ± -Inf	-Inf ± 0
7	0 ± 0	2.87 ± -Inf	-0.78 ± -Inf	-Inf ± -Inf	-Inf ± -Inf	-Inf ± 0
8	0 ± 0	2.57 ± -Inf	-1.13 ± -Inf	-Inf ± -Inf	-Inf ± -Inf	-Inf ± 0
9	0 ± 0	2.27 ± -Inf	-1.47 ± -Inf	-Inf ± -Inf	-Inf ± -Inf	-Inf ± 0
10	0 ± 0	1.97 ± -Inf	-1.81 ± -Inf	-Inf ± -Inf	-Inf ± -Inf	-Inf ± 0
11	0 ± 0	1.67 ± -Inf	-2.16 ± -Inf	-Inf ± -Inf	-Inf ± -Inf	-Inf ± 0
12	0 ± 0	1.37 ± -Inf	-2.5 ± -Inf	-Inf ± -Inf	-Inf ± -Inf	-Inf ± 0
13	0 ± 0	1.07 ± -Inf	-2.85 ± -Inf	-Inf ± -Inf	-Inf ± -Inf	-Inf ± 0
14	0 ± 0	0.76 ± -Inf	-3.19 ± -Inf	-Inf ± -Inf	-Inf ± -Inf	-Inf ± 0
15	0 ± 0	0.46 ± -Inf	-3.53 ± -Inf	-Inf ± -Inf	-Inf ± -Inf	-Inf ± 0
16	0 ± 0	0.16 ± -Inf	-3.87 ± -Inf	-Inf ± -Inf	-Inf ± -Inf	-Inf ± 0
17	0 ± 0	-0.14 ± -Inf	-4.21 ± -Inf	-Inf ± -Inf	-Inf ± -Inf	-Inf ± 0
18	0 ± 0	-0.44 ± -Inf	-4.55 ± -Inf	-Inf ± -Inf	-Inf ± -Inf	-Inf ± 0
19	0 ± 0	-0.74 ± -Inf	-4.89 ± -Inf	-Inf ± -Inf	-Inf ± -Inf	-Inf ± 0
20	0 ± 0	-1.04 ± -Inf	-5.23 ± -Inf	-Inf ± -Inf	-Inf ± -Inf	-Inf ± 0
21	0 ± 0	-1.34 ± -Inf	-5.57 ± -Inf	-Inf ± -Inf	-Inf ± -Inf	-Inf ± 0
22	0 ± 0	-1.64 ± -Inf	-5.9 ± -Inf	-Inf ± -Inf	-Inf ± -Inf	-Inf ± 0
23	0 ± 0	-1.94 ± -Inf	-6.24 ± -Inf	-Inf ± -Inf	-Inf ± -Inf	-Inf ± 0
24	0 ± 0	-2.25 ± -Inf	-6.57 ± -Inf	-Inf ± -Inf	-Inf ± -Inf	-Inf ± 0
25	0 ± 0	-2.55 ± -Inf	-6.9 ± -Inf	-Inf ± -Inf	-Inf ± -Inf	-Inf ± 0
26	0 ± 0	-2.85 ± -Inf	-7.23 ± -Inf	-Inf ± -Inf	-Inf ± -Inf	-Inf ± 0
27	0 ± 0	-3.15 ± -Inf	-7.57 ± -Inf	-Inf ± -Inf	-Inf ± -Inf	-Inf ± 0
28	0 ± 0	-3.45 ± -Inf	-7.9 ± -Inf	-Inf ± -Inf	-Inf ± -Inf	-Inf ± 0
29	0 ± 0	-3.75 ± -Inf	-8.23 ± -Inf	-Inf ± -Inf	-Inf ± -Inf	-Inf ± 0
30	0 ± 0	-4.05 ± -Inf	-8.55 ± -Inf	-Inf ± -Inf	-Inf ± -Inf	-Inf ± 0
31	0 ± 0	-4.35 ± -Inf	-8.88 ± -Inf	-Inf ± -Inf	-Inf ± -Inf	-Inf ± 0
32	0 ± 0	-4.65 ± -Inf	-9.21 ± -Inf	-Inf ± -Inf	-Inf ± -Inf	-Inf ± 0
33	0 ± 0	-4.95 ± -Inf	-9.54 ± -Inf	-Inf ± -Inf	-Inf ± -Inf	-Inf ± 0
34	0 ± 0	-5.26 ± -Inf	-9.86 ± -Inf	-Inf ± -Inf	-Inf ± -Inf	-Inf ± 0
35	0 ± 0	-5.56 ± -Inf	-10.19 ± -Inf	-Inf ± -Inf	-Inf ± -Inf	-Inf ± 0
36	0 ± 0	-5.86 ± -Inf	-10.51 ± -Inf	-Inf ± -Inf	-Inf ± -Inf	-Inf ± 0
37	0 ± 0	-6.16 ± -Inf	-10.84 ± -Inf	-Inf ± -Inf	-Inf ± -Inf	-Inf ± 0

The average of the prevalence, the CFU-values per square meter (cfu/sqm) and per gram manure are calculated over all iterations. The CFU values in the intestine as well as the excreted and ingested quantities are obtained by calculating the average over all animals in one iteration at first and then over all iterations. The decadic logarithm of zero or negative values is negative infinite (-Inf).

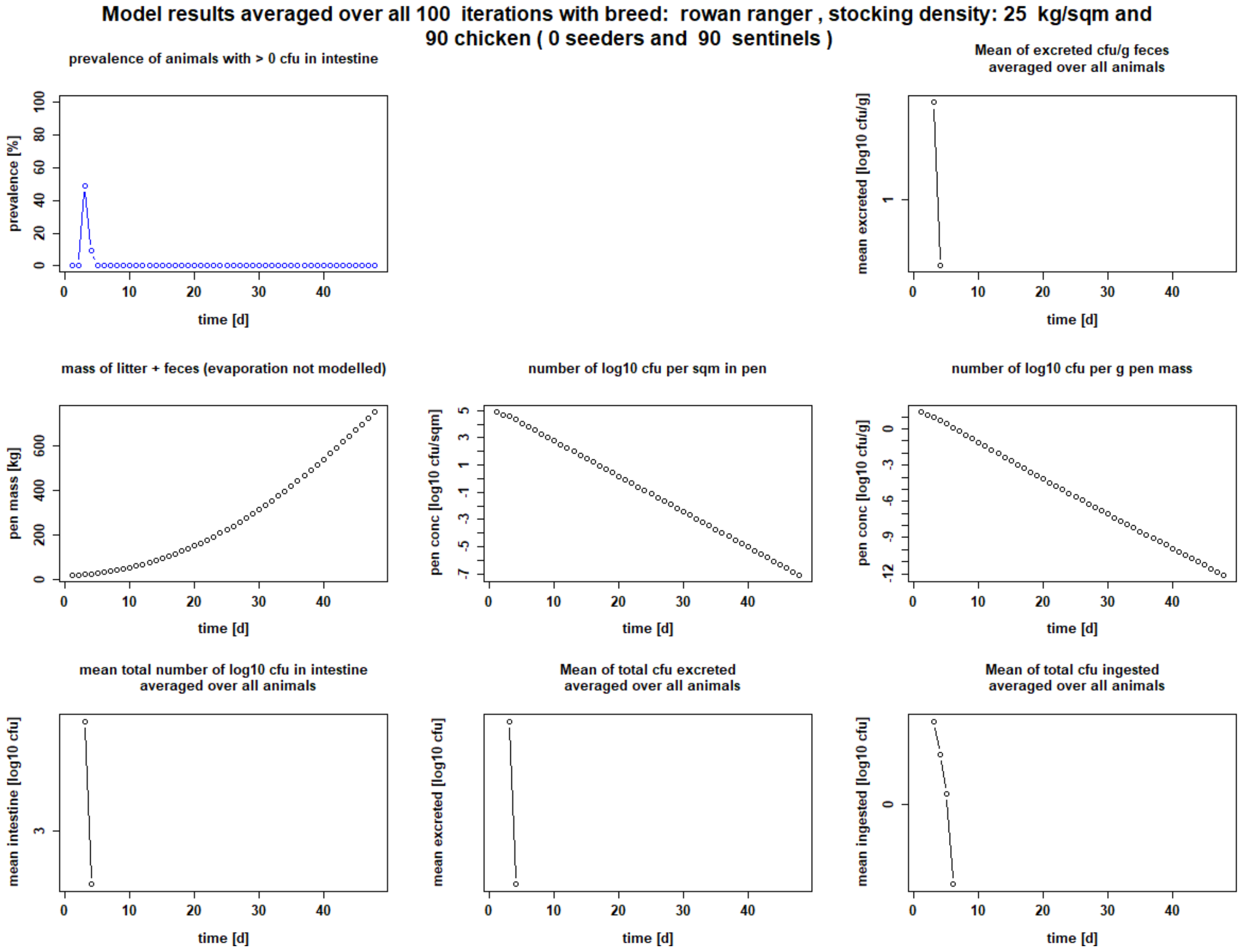

Figure S27. Calculated infection dynamic for the scenario when the pen is contaminated with 10^6 CFU at the beginning of the fattening period, combined measure slow growing breed, stocking density 25 kg/m^2 and 3000 g litter/m^2. Dotplots with results for 90 negative birds, Rowan x Ranger, feeding duration 47 days, stocking density 25 kg/m^2, 3000 g litter/m^2

Table S27a (CFU). Calculated numeric values for scenario when the pen is contaminated with 10^6 CFU at the beginning of the fattening period, combined measure slow growing breed, stocking density 25 kg/m^2 and 3000 g litter/m^2. Setting: 90 negative birds, Rowan x Ranger, feeding duration 47 days, stocking density 25 kg/m^2, 3000 g litter/m^2

day	prevalence in %	cfu/sqm	cfu/g manure	cfu in intestine	cfu/g feces	cfu ingested per animal
	(mean ± sd)	(mean ± sd)	(mean ± sd)	(mean ± sd)	(mean ± sd)	(mean ± sd)
1	0 ± 0	7.3e4 ± 0e4	2.3e1 ± 0e1	0e0 ± 0	0e0 ± 0	0e0 ± 0
2	0 ± 0	3.6e4 ± 0e4	1.2e1 ± 0e1	0e0 ± 0	0e0 ± 0	0e0 ± 0
3	32.12 ± 5.09	2.6e4 ± 0.4e4	7.3 ± 1	2e3 ± 0.9e3	1.9e1 ± 0.9e1	3 ± 0
4	0 ± 0	1.3e4 ± 0.2e4	3.2 ± 0.5	0e0 ± 0	0e0 ± 0	1.5 ± 0.5
5	0 ± 0	6.5e3 ± 0.9e3	1.4 ± 0.2	0e0 ± 0	0e0 ± 0	2.6e-1 ± 4.4e-1
6	0 ± 0	3.3e3 ± 0.5e3	6.6e-1 ± 0.9e-1	0e0 ± 0	0e0 ± 0	0e0 ± 0
7	0 ± 0	1.6e3 ± 0.2e3	2.9e-1 ± 0.4e-1	0e0 ± 0	0e0 ± 0	0e0 ± 0
8	0 ± 0	8.2e2 ± 1.1e2	1.3e-1 ± 0.2e-1	0e0 ± 0	0e0 ± 0	0e0 ± 0
9	0 ± 0	4.1e2 ± 0.6e2	5.8e-2 ± 0.8e-2	0e0 ± 0	0e0 ± 0	0e0 ± 0
10	0 ± 0	2e2 ± 0.3e2	2.6e-2 ± 0.4e-2	0e0 ± 0	0e0 ± 0	0e0 ± 0
11	0 ± 0	1e2 ± 0.1e2	1.1e-2 ± 0.2e-2	0e0 ± 0	0e0 ± 0	0e0 ± 0
12	0 ± 0	5.1e1 ± 0.7e1	5.1e-3 ± 0.7e-3	0e0 ± 0	0e0 ± 0	0e0 ± 0
13	0 ± 0	2.6e1 ± 0.4e1	2.3e-3 ± 0.3e-3	0e0 ± 0	0e0 ± 0	0e0 ± 0
14	0 ± 0	1.3e1 ± 0.2e1	1e-3 ± 0.1e-3	0e0 ± 0	0e0 ± 0	0e0 ± 0
15	0 ± 0	6.4 ± 0.9	4.6e-4 ± 0.6e-4	0e0 ± 0	0e0 ± 0	0e0 ± 0
16	0 ± 0	3.2 ± 0.4	2.1e-4 ± 0.3e-4	0e0 ± 0	0e0 ± 0	0e0 ± 0
17	0 ± 0	1.6 ± 0.2	9.5e-5 ± 1.3e-5	0e0 ± 0	0e0 ± 0	0e0 ± 0
18	0 ± 0	8e-1 ± 1.1e-1	4.3e-5 ± 0.6e-5	0e0 ± 0	0e0 ± 0	0e0 ± 0
19	0 ± 0	4e-1 ± 0.6e-1	2e-5 ± 0.3e-5	0e0 ± 0	0e0 ± 0	0e0 ± 0
20	0 ± 0	2e-1 ± 0.3e-1	9e-6 ± 1.3e-6	0e0 ± 0	0e0 ± 0	0e0 ± 0
21	0 ± 0	10e-2 ± 1.4e-2	4.2e-6 ± 0.6e-6	0e0 ± 0	0e0 ± 0	0e0 ± 0
22	0 ± 0	5e-2 ± 0.7e-2	1.9e-6 ± 0.3e-6	0e0 ± 0	0e0 ± 0	0e0 ± 0
23	0 ± 0	2.5e-2 ± 0.3e-2	8.9e-7 ± 1.2e-7	0e0 ± 0	0e0 ± 0	0e0 ± 0
24	0 ± 0	1.2e-2 ± 0.2e-2	4.1e-7 ± 0.6e-7	0e0 ± 0	0e0 ± 0	0e0 ± 0
25	0 ± 0	6.2e-3 ± 0.9e-3	1.9e-7 ± 0.3e-7	0e0 ± 0	0e0 ± 0	0e0 ± 0
26	0 ± 0	3.1e-3 ± 0.4e-3	8.9e-8 ± 1.2e-8	0e0 ± 0	0e0 ± 0	0e0 ± 0
27	0 ± 0	1.6e-3 ± 0.2e-3	4.2e-8 ± 0.6e-8	0e0 ± 0	0e0 ± 0	0e0 ± 0
28	0 ± 0	7.8e-4 ± 1.1e-4	2e-8 ± 0.3e-8	0e0 ± 0	0e0 ± 0	0e0 ± 0
29	0 ± 0	3.9e-4 ± 0.5e-4	9.1e-9 ± 1.3e-9	0e0 ± 0	0e0 ± 0	0e0 ± 0
30	0 ± 0	1.9e-4 ± 0.3e-4	4.3e-9 ± 0.6e-9	0e0 ± 0	0e0 ± 0	0e0 ± 0
31	0 ± 0	9.7e-5 ± 1.4e-5	2e-9 ± 0.3e-9	0e0 ± 0	0e0 ± 0	0e0 ± 0
32	0 ± 0	4.9e-5 ± 0.7e-5	9.5e-10 ± 1.3e-10	0e0 ± 0	0e0 ± 0	0e0 ± 0
33	0 ± 0	2.4e-5 ± 0.3e-5	4.5e-10 ± 0.6e-10	0e0 ± 0	0e0 ± 0	0e0 ± 0
34	0 ± 0	1.2e-5 ± 0.2e-5	2.1e-10 ± 0.3e-10	0e0 ± 0	0e0 ± 0	0e0 ± 0
35	0 ± 0	6.1e-6 ± 0.8e-6	1e-10 ± 0.1e-10	0e0 ± 0	0e0 ± 0	0e0 ± 0
36	0 ± 0	3e-6 ± 0.4e-6	4.8e-11 ± 0.7e-11	0e0 ± 0	0e0 ± 0	0e0 ± 0
37	0 ± 0	1.5e-6 ± 0.2e-6	2.3e-11 ± 0.3e-11	0e0 ± 0	0e0 ± 0	0e0 ± 0
38	0 ± 0	7.6e-7 ± 1.1e-7	1.1e-11 ± 0.1e-11	0e0 ± 0	0e0 ± 0	0e0 ± 0
39	0 ± 0	3.8e-7 ± 0.5e-7	5.1e-12 ± 0.7e-12	0e0 ± 0	0e0 ± 0	0e0 ± 0
40	0 ± 0	1.9e-7 ± 0.3e-7	2.4e-12 ± 0.3e-12	0e0 ± 0	0e0 ± 0	0e0 ± 0
41	0 ± 0	9.5e-8 ± 1.3e-8	1.2e-12 ± 0.2e-12	0e0 ± 0	0e0 ± 0	0e0 ± 0
42	0 ± 0	4.8e-8 ± 0.7e-8	5.6e-13 ± 0.8e-13	0e0 ± 0	0e0 ± 0	0e0 ± 0
43	0 ± 0	2.4e-8 ± 0.3e-8	2.7e-13 ± 0.4e-13	0e0 ± 0	0e0 ± 0	0e0 ± 0
44	0 ± 0	1.2e-8 ± 0.2e-8	1.3e-13 ± 0.2e-13	0e0 ± 0	0e0 ± 0	0e0 ± 0
45	0 ± 0	5.9e-9 ± 0.8e-9	6.1e-14 ± 0.9e-14	0e0 ± 0	0e0 ± 0	0e0 ± 0
46	0 ± 0	3e-9 ± 0.4e-9	2.9e-14 ± 0.4e-14	0e0 ± 0	0e0 ± 0	0e0 ± 0
47	0 ± 0	1.5e-9 ± 0.2e-9	1.4e-14 ± 0.2e-14	0e0 ± 0	0e0 ± 0	0e0 ± 0
48	0 ± 0	7.4e-10 ± 1e-10	6.8e-15 ± 0.9e-15	0e0 ± 0	0e0 ± 0	0e0 ± 0

The average of the prevalence, the CFU-values per square meter (cfu/sqm) and per gram manure are calculated over all iterations. The CFU values in the intestine as well as the excreted and ingested quantities are obtained by calculating the average over all animals in one iteration at first and then over all iterations.

Table S27b (log_{10} CFU). Calculated numeric values for scenario when the pen is contaminated with 10^6 CFU at the beginning of the fattening period, combined measure slow growing breed, stocking density 25 kg/m^2 and 3000 g litter/m^2. Setting: 90 negative birds, Rowan x Ranger, feeding duration 47 days, stocking density 25 kg/m^2, 3000 g litter/m^2

Model results averaged of all birds and iterations with breed: rowan ranger , stocking density: 25 kg/sqm and 90 chicken (0 seeders and 90 sentinels)

day	prevalence in %	cfu/sqm	cfu/g manure	cfu in intestine	cfu/g feces	cfu ingested per animal
	(% mean ± sd)	(log10 mean ± sd)	(log10 mean ± sd)	(log10 mean ± sd)	(log10 mean ± sd)	(log10 mean ± sd)
1	0 ± 0	4.86 ± -Inf	1.37 ± -Inf	-Inf ± -Inf	-Inf ± -Inf	-Inf ± 0
2	0 ± 0	4.56 ± -Inf	1.06 ± -Inf	-Inf ± -Inf	-Inf ± -Inf	-Inf ± 0
3	32.12 ± 5.09	4.42 ± 3.56	0.86 ± 0.01	3.31 ± 2.97	1.29 ± 0.95	0.48 ± 0
4	0 ± 0	4.12 ± 3.26	0.51 ± -0.35	-Inf ± -Inf	-Inf ± -Inf	0.18 ± -0.3
5	0 ± 0	3.82 ± 2.96	0.16 ± -0.7	-Inf ± -Inf	-Inf ± -Inf	-0.59 ± -0.36
6	0 ± 0	3.51 ± 2.66	-0.18 ± -1.04	-Inf ± -Inf	-Inf ± -Inf	-Inf ± 0
7	0 ± 0	3.21 ± 2.36	-0.53 ± -1.39	-Inf ± -Inf	-Inf ± -Inf	-Inf ± 0
8	0 ± 0	2.91 ± 2.06	-0.89 ± -1.74	-Inf ± -Inf	-Inf ± -Inf	-Inf ± 0
9	0 ± 0	2.61 ± 1.75	-1.24 ± -2.09	-Inf ± -Inf	-Inf ± -Inf	-Inf ± 0
10	0 ± 0	2.31 ± 1.45	-1.59 ± -2.45	-Inf ± -Inf	-Inf ± -Inf	-Inf ± 0
11	0 ± 0	2.01 ± 1.15	-1.94 ± -2.8	-Inf ± -Inf	-Inf ± -Inf	-Inf ± 0
12	0 ± 0	1.71 ± 0.85	-2.29 ± -3.15	-Inf ± -Inf	-Inf ± -Inf	-Inf ± 0
13	0 ± 0	1.41 ± 0.55	-2.64 ± -3.5	-Inf ± -Inf	-Inf ± -Inf	-Inf ± 0
14	0 ± 0	1.11 ± 0.25	-2.99 ± -3.85	-Inf ± -Inf	-Inf ± -Inf	-Inf ± 0
15	0 ± 0	0.8 ± -0.05	-3.34 ± -4.19	-Inf ± -Inf	-Inf ± -Inf	-Inf ± 0
16	0 ± 0	0.5 ± -0.35	-3.68 ± -4.54	-Inf ± -Inf	-Inf ± -Inf	-Inf ± 0
17	0 ± 0	0.2 ± -0.65	-4.02 ± -4.88	-Inf ± -Inf	-Inf ± -Inf	-Inf ± 0
18	0 ± 0	-0.1 ± -0.95	-4.37 ± -5.22	-Inf ± -Inf	-Inf ± -Inf	-Inf ± 0
19	0 ± 0	-0.4 ± -1.26	-4.71 ± -5.56	-Inf ± -Inf	-Inf ± -Inf	-Inf ± 0
20	0 ± 0	-0.7 ± -1.56	-5.04 ± -5.9	-Inf ± -Inf	-Inf ± -Inf	-Inf ± 0
21	0 ± 0	-1 ± -1.86	-5.38 ± -6.24	-Inf ± -Inf	-Inf ± -Inf	-Inf ± 0
22	0 ± 0	-1.3 ± -2.16	-5.72 ± -6.57	-Inf ± -Inf	-Inf ± -Inf	-Inf ± 0
23	0 ± 0	-1.6 ± -2.46	-6.05 ± -6.91	-Inf ± -Inf	-Inf ± -Inf	-Inf ± 0
24	0 ± 0	-1.9 ± -2.76	-6.39 ± -7.24	-Inf ± -Inf	-Inf ± -Inf	-Inf ± 0
25	0 ± 0	-2.21 ± -3.06	-6.72 ± -7.57	-Inf ± -Inf	-Inf ± -Inf	-Inf ± 0
26	0 ± 0	-2.51 ± -3.36	-7.05 ± -7.91	-Inf ± -Inf	-Inf ± -Inf	-Inf ± 0
27	0 ± 0	-2.81 ± -3.66	-7.38 ± -8.24	-Inf ± -Inf	-Inf ± -Inf	-Inf ± 0
28	0 ± 0	-3.11 ± -3.96	-7.71 ± -8.57	-Inf ± -Inf	-Inf ± -Inf	-Inf ± 0
29	0 ± 0	-3.41 ± -4.27	-8.04 ± -8.9	-Inf ± -Inf	-Inf ± -Inf	-Inf ± 0
30	0 ± 0	-3.71 ± -4.57	-8.37 ± -9.22	-Inf ± -Inf	-Inf ± -Inf	-Inf ± 0
31	0 ± 0	-4.01 ± -4.87	-8.7 ± -9.55	-Inf ± -Inf	-Inf ± -Inf	-Inf ± 0
32	0 ± 0	-4.31 ± -5.17	-9.02 ± -9.88	-Inf ± -Inf	-Inf ± -Inf	-Inf ± 0
33	0 ± 0	-4.61 ± -5.47	-9.35 ± -10.21	-Inf ± -Inf	-Inf ± -Inf	-Inf ± 0
34	0 ± 0	-4.91 ± -5.77	-9.67 ± -10.53	-Inf ± -Inf	-Inf ± -Inf	-Inf ± 0
35	0 ± 0	-5.22 ± -6.07	-10 ± -10.86	-Inf ± -Inf	-Inf ± -Inf	-Inf ± 0
36	0 ± 0	-5.52 ± -6.37	-10.32 ± -11.18	-Inf ± -Inf	-Inf ± -Inf	-Inf ± 0
37	0 ± 0	-5.82 ± -6.67	-10.65 ± -11.5	-Inf ± -Inf	-Inf ± -Inf	-Inf ± 0
38	0 ± 0	-6.12 ± -6.98	-10.97 ± -11.83	-Inf ± -Inf	-Inf ± -Inf	-Inf ± 0
39	0 ± 0	-6.42 ± -7.28	-11.29 ± -12.15	-Inf ± -Inf	-Inf ± -Inf	-Inf ± 0
40	0 ± 0	-6.72 ± -7.58	-11.61 ± -12.47	-Inf ± -Inf	-Inf ± -Inf	-Inf ± 0
41	0 ± 0	-7.02 ± -7.88	-11.93 ± -12.79	-Inf ± -Inf	-Inf ± -Inf	-Inf ± 0
42	0 ± 0	-7.32 ± -8.18	-12.26 ± -13.11	-Inf ± -Inf	-Inf ± -Inf	-Inf ± 0
43	0 ± 0	-7.62 ± -8.48	-12.58 ± -13.43	-Inf ± -Inf	-Inf ± -Inf	-Inf ± 0
44	0 ± 0	-7.92 ± -8.78	-12.89 ± -13.75	-Inf ± -Inf	-Inf ± -Inf	-Inf ± 0
45	0 ± 0	-8.23 ± -9.08	-13.21 ± -14.07	-Inf ± -Inf	-Inf ± -Inf	-Inf ± 0
46	0 ± 0	-8.53 ± -9.38	-13.53 ± -14.39	-Inf ± -Inf	-Inf ± -Inf	-Inf ± 0
47	0 ± 0	-8.83 ± -9.68	-13.85 ± -14.71	-Inf ± -Inf	-Inf ± -Inf	-Inf ± 0
48	0 ± 0	-9.13 ± -9.99	-14.17 ± -15.02	-Inf ± -Inf	-Inf ± -Inf	-Inf ± 0

The average of the prevalence, the CFU-values per square meter (cfu/sqm) and per gram manure are calculated over all iterations. The CFU values in the intestine as well as the excreted and ingested quantities are obtained by calculating the average over all animals in one iteration at first and then over all iterations. The decadic logarithm of zero or negative values is negative infinite (-Inf).

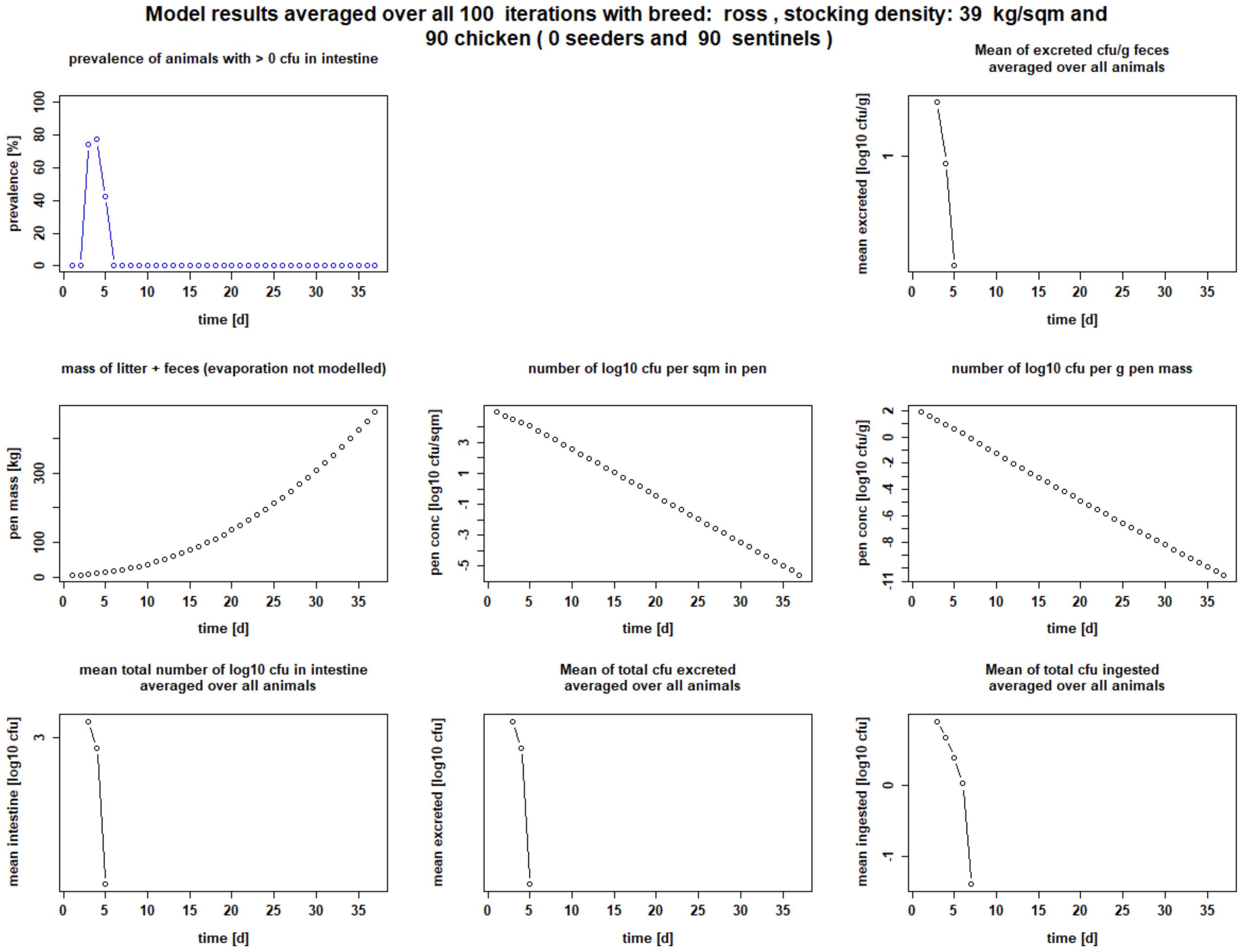

Figure S28. Calculated infection dynamic for the scenario when the pen is contaminated with 10^6 CFU at the beginning of the fattening period, measure reduced growth rate of ESBLs in the intestines. Dotplots with results for 90 negative birds, Ross 308, feeding duration 36 days, stocking density 39 kg/m^2, 1000 g litter/m^2, growth rate 10^4

Table S28a (CFU). Calculated numeric values for scenario when the pen is contaminated with 10^6 CFU at the beginning of the fattening period, measure reduced growth rate of ESBLs in the intestines. Setting: 90 negative birds, Ross 308, feeding duration 36 days, stocking density 39 kg/m^2, 1000 g litter/m^2, growth rate 10^4

day	prevalence in %	cfu/sqm	cfu/g manure	cfu in intestine	cfu/g feces	cfu ingested per animal
	(mean ± sd)	(mean ± sd)	(mean ± sd)	(mean ± sd)	(mean ± sd)	(mean ± sd)
1	0 ± 0	9.3e4 ± 0e4	8e1 ± 0e1	0e0 ± 0	0e0 ± 0	0e0 ± 0
2	0 ± 0	4.6e4 ± 0e4	3.5e1 ± 0e1	0e0 ± 0	0e0 ± 0	0e0 ± 0
3	73.54 ± 4.64	2.9e4 ± 0.1e4	1.7e1 ± 0.1e1	1.2e3 ± 0.3e3	1.8e1 ± 0.4e1	8 ± 0
4	77.04 ± 4.79	1.9e4 ± 0.2e4	8.9 ± 0.8	9.3e2 ± 2.8e2	9.3 ± 2.8	4.72 ± 0.5
5	43.66 ± 15.02	1.2e4 ± 0.2e4	4.2 ± 0.7	4.1e2 ± 2.3e2	3.5 ± 2	2.44 ± 0.5
6	0 ± 0	5.8e3 ± 0.9e3	1.8 ± 0.3	0e0 ± 0	0e0 ± 0	1.08 ± 0.3
7	0 ± 0	2.9e3 ± 0.5e3	7.3e-1 ± 1.1e-1	0e0 ± 0	0e0 ± 0	3e-2 ± 17.1e-2
8	0 ± 0	1.5e3 ± 0.2e3	3e-1 ± 0.5e-1	0e0 ± 0	0e0 ± 0	0e0 ± 0
9	0 ± 0	7.3e2 ± 1.1e2	1.3e-1 ± 0.2e-1	0e0 ± 0	0e0 ± 0	0e0 ± 0
10	0 ± 0	3.6e2 ± 0.6e2	5.3e-2 ± 0.8e-2	0e0 ± 0	0e0 ± 0	0e0 ± 0
11	0 ± 0	1.8e2 ± 0.3e2	2.2e-2 ± 0.3e-2	0e0 ± 0	0e0 ± 0	0e0 ± 0
12	0 ± 0	9.1e1 ± 1.4e1	9.5e-3 ± 1.5e-3	0e0 ± 0	0e0 ± 0	0e0 ± 0
13	0 ± 0	4.6e1 ± 0.7e1	4.1e-3 ± 0.6e-3	0e0 ± 0	0e0 ± 0	0e0 ± 0
14	0 ± 0	2.3e1 ± 0.4e1	1.8e-3 ± 0.3e-3	0e0 ± 0	0e0 ± 0	0e0 ± 0
15	0 ± 0	1.1e1 ± 0.2e1	7.8e-4 ± 1.2e-4	0e0 ± 0	0e0 ± 0	0e0 ± 0
16	0 ± 0	5.7 ± 0.9	3.5e-4 ± 0.5e-4	0e0 ± 0	0e0 ± 0	0e0 ± 0
17	0 ± 0	2.8 ± 0.4	1.5e-4 ± 0.2e-4	0e0 ± 0	0e0 ± 0	0e0 ± 0
18	0 ± 0	1.4 ± 0.2	6.9e-5 ± 1.1e-5	0e0 ± 0	0e0 ± 0	0e0 ± 0
19	0 ± 0	7.1e-1 ± 1.1e-1	3.1e-5 ± 0.5e-5	0e0 ± 0	0e0 ± 0	0e0 ± 0
20	0 ± 0	3.6e-1 ± 0.6e-1	1.4e-5 ± 0.2e-5	0e0 ± 0	0e0 ± 0	0e0 ± 0
21	0 ± 0	1.8e-1 ± 0.3e-1	6.4e-6 ± 1e-6	0e0 ± 0	0e0 ± 0	0e0 ± 0
22	0 ± 0	8.9e-2 ± 1.4e-2	2.9e-6 ± 0.5e-6	0e0 ± 0	0e0 ± 0	0e0 ± 0
23	0 ± 0	4.4e-2 ± 0.7e-2	1.3e-6 ± 0.2e-6	0e0 ± 0	0e0 ± 0	0e0 ± 0
24	0 ± 0	2.2e-2 ± 0.3e-2	6.1e-7 ± 0.9e-7	0e0 ± 0	0e0 ± 0	0e0 ± 0
25	0 ± 0	1.1e-2 ± 0.2e-2	2.8e-7 ± 0.4e-7	0e0 ± 0	0e0 ± 0	0e0 ± 0
26	0 ± 0	5.6e-3 ± 0.9e-3	1.3e-7 ± 0.2e-7	0e0 ± 0	0e0 ± 0	0e0 ± 0
27	0 ± 0	2.8e-3 ± 0.4e-3	6e-8 ± 0.9e-8	0e0 ± 0	0e0 ± 0	0e0 ± 0
28	0 ± 0	1.4e-3 ± 0.2e-3	2.8e-8 ± 0.4e-8	0e0 ± 0	0e0 ± 0	0e0 ± 0
29	0 ± 0	7e-4 ± 1.1e-4	1.3e-8 ± 0.2e-8	0e0 ± 0	0e0 ± 0	0e0 ± 0
30	0 ± 0	3.5e-4 ± 0.5e-4	6.1e-9 ± 0.9e-9	0e0 ± 0	0e0 ± 0	0e0 ± 0
31	0 ± 0	1.7e-4 ± 0.3e-4	2.8e-9 ± 0.4e-9	0e0 ± 0	0e0 ± 0	0e0 ± 0
32	0 ± 0	8.7e-5 ± 1.3e-5	1.3e-9 ± 0.2e-9	0e0 ± 0	0e0 ± 0	0e0 ± 0
33	0 ± 0	4.3e-5 ± 0.7e-5	6.2e-10 ± 1e-10	0e0 ± 0	0e0 ± 0	0e0 ± 0
34	0 ± 0	2.2e-5 ± 0.3e-5	2.9e-10 ± 0.5e-10	0e0 ± 0	0e0 ± 0	0e0 ± 0
35	0 ± 0	1.1e-5 ± 0.2e-5	1.4e-10 ± 0.2e-10	0e0 ± 0	0e0 ± 0	0e0 ± 0
36	0 ± 0	5.4e-6 ± 0.8e-6	6.5e-11 ± 1e-11	0e0 ± 0	0e0 ± 0	0e0 ± 0
37	0 ± 0	2.7e-6 ± 0.4e-6	3.1e-11 ± 0.5e-11	0e0 ± 0	0e0 ± 0	0e0 ± 0

The average of the prevalence, the CFU-values per square meter (cfu/sqm) and per gram manure are calculated over all iterations. The CFU values in the intestine as well as the excreted and ingested quantities are obtained by calculating the average over all animals in one iteration at first and then over all iterations.

Table S28b (log_{10} CFU). Calculated numeric values for scenario when the pen is contaminated with 10^6 CFU at the beginning of the fattening period, measure reduced growth rate of ESBLs in the intestines. Setting: 90 negative birds, Ross 308, feeding duration 36 days, stocking density 39 kg/m^2, 1000 g litter/m^2, growth rate 10^4

day	prevalence in %	cfu/sqm	cfu/g manure	cfu in intestine	cfu/g feces	cfu ingested per animal
	(% mean ± sd)	(log10 mean ± sd)	(log10 mean ± sd)	(log10 mean ± sd)	(log10 mean ± sd)	(log10 mean ± sd)
1	0 ± 0	4.97 ± -Inf	1.9 ± -Inf	-Inf ± -Inf	-Inf ± -Inf	-Inf ± 0
2	0 ± 0	4.67 ± -Inf	1.54 ± -Inf	-Inf ± -Inf	-Inf ± -Inf	-Inf ± 0
3	73.54 ± 4.64	4.46 ± 3.14	1.24 ± -0.08	3.06 ± 2.44	1.25 ± 0.63	0.9 ± 0
4	77.04 ± 4.79	4.28 ± 3.23	0.95 ± -0.11	2.97 ± 2.44	0.97 ± 0.44	0.67 ± -0.35
5	43.66 ± 15.02	4.07 ± 3.26	0.63 ± -0.18	2.62 ± 2.37	0.55 ± 0.3	0.39 ± -0.3
6	0 ± 0	3.77 ± 2.96	0.26 ± -0.55	-Inf ± -Inf	-Inf ± -Inf	0.03 ± -0.56
7	0 ± 0	3.46 ± 2.65	-0.14 ± -0.95	-Inf ± -Inf	-Inf ± -Inf	-1.52 ± -0.77
8	0 ± 0	3.16 ± 2.35	-0.52 ± -1.33	-Inf ± -Inf	-Inf ± -Inf	-Inf ± 0
9	0 ± 0	2.86 ± 2.05	-0.9 ± -1.71	-Inf ± -Inf	-Inf ± -Inf	-Inf ± 0
10	0 ± 0	2.56 ± 1.75	-1.28 ± -2.09	-Inf ± -Inf	-Inf ± -Inf	-Inf ± 0
11	0 ± 0	2.26 ± 1.45	-1.65 ± -2.46	-Inf ± -Inf	-Inf ± -Inf	-Inf ± 0
12	0 ± 0	1.96 ± 1.15	-2.02 ± -2.83	-Inf ± -Inf	-Inf ± -Inf	-Inf ± 0
13	0 ± 0	1.66 ± 0.85	-2.39 ± -3.2	-Inf ± -Inf	-Inf ± -Inf	-Inf ± 0
14	0 ± 0	1.36 ± 0.55	-2.75 ± -3.56	-Inf ± -Inf	-Inf ± -Inf	-Inf ± 0
15	0 ± 0	1.06 ± 0.25	-3.11 ± -3.92	-Inf ± -Inf	-Inf ± -Inf	-Inf ± 0
16	0 ± 0	0.76 ± -0.05	-3.46 ± -4.27	-Inf ± -Inf	-Inf ± -Inf	-Inf ± 0
17	0 ± 0	0.45 ± -0.36	-3.81 ± -4.62	-Inf ± -Inf	-Inf ± -Inf	-Inf ± 0
18	0 ± 0	0.15 ± -0.66	-4.16 ± -4.97	-Inf ± -Inf	-Inf ± -Inf	-Inf ± 0
19	0 ± 0	-0.15 ± -0.96	-4.51 ± -5.32	-Inf ± -Inf	-Inf ± -Inf	-Inf ± 0
20	0 ± 0	-0.45 ± -1.26	-4.85 ± -5.66	-Inf ± -Inf	-Inf ± -Inf	-Inf ± 0
21	0 ± 0	-0.75 ± -1.56	-5.19 ± -6	-Inf ± -Inf	-Inf ± -Inf	-Inf ± 0
22	0 ± 0	-1.05 ± -1.86	-5.53 ± -6.34	-Inf ± -Inf	-Inf ± -Inf	-Inf ± 0
23	0 ± 0	-1.35 ± -2.16	-5.87 ± -6.68	-Inf ± -Inf	-Inf ± -Inf	-Inf ± 0
24	0 ± 0	-1.65 ± -2.46	-6.21 ± -7.02	-Inf ± -Inf	-Inf ± -Inf	-Inf ± 0
25	0 ± 0	-1.95 ± -2.76	-6.55 ± -7.36	-Inf ± -Inf	-Inf ± -Inf	-Inf ± 0
26	0 ± 0	-2.25 ± -3.07	-6.88 ± -7.69	-Inf ± -Inf	-Inf ± -Inf	-Inf ± 0
27	0 ± 0	-2.56 ± -3.37	-7.22 ± -8.03	-Inf ± -Inf	-Inf ± -Inf	-Inf ± 0
28	0 ± 0	-2.86 ± -3.67	-7.55 ± -8.36	-Inf ± -Inf	-Inf ± -Inf	-Inf ± 0
29	0 ± 0	-3.16 ± -3.97	-7.89 ± -8.7	-Inf ± -Inf	-Inf ± -Inf	-Inf ± 0
30	0 ± 0	-3.46 ± -4.27	-8.22 ± -9.03	-Inf ± -Inf	-Inf ± -Inf	-Inf ± 0
31	0 ± 0	-3.76 ± -4.57	-8.55 ± -9.36	-Inf ± -Inf	-Inf ± -Inf	-Inf ± 0
32	0 ± 0	-4.06 ± -4.87	-8.88 ± -9.69	-Inf ± -Inf	-Inf ± -Inf	-Inf ± 0
33	0 ± 0	-4.36 ± -5.17	-9.21 ± -10.02	-Inf ± -Inf	-Inf ± -Inf	-Inf ± 0
34	0 ± 0	-4.66 ± -5.47	-9.53 ± -10.34	-Inf ± -Inf	-Inf ± -Inf	-Inf ± 0
35	0 ± 0	-4.96 ± -5.77	-9.86 ± -10.67	-Inf ± -Inf	-Inf ± -Inf	-Inf ± 0
36	0 ± 0	-5.27 ± -6.08	-10.19 ± -11	-Inf ± -Inf	-Inf ± -Inf	-Inf ± 0
37	0 ± 0	-5.57 ± -6.38	-10.51 ± -11.32	-Inf ± -Inf	-Inf ± -Inf	-Inf ± 0

The average of the prevalence, the CFU-values per square meter (cfu/sqm) and per gram manure are calculated over all iterations. The CFU values in the intestine as well as the excreted and ingested quantities are obtained by calculating the average over all animals in one iteration at first and then over all iterations. The decadic logarithm of zero or negative values is negative infinite (-Inf).
